# Dynamics of Whole-Genome Contacts of Nucleoli in *Drosophila* Cells Suggests a Role for rDNA Genes in Global Epigenetic Regulation

**DOI:** 10.3390/cells9122587

**Published:** 2020-12-03

**Authors:** Nickolai A. Tchurikov, Elena S. Klushevskaya, Daria M. Fedoseeva, Ildar R. Alembekov, Galina I. Kravatskaya, Vladimir R. Chechetkin, Yuri V. Kravatsky, Olga V. Kretova

**Affiliations:** Department of Epigenetic Mechanisms of Gene Expression Regulation, Engelhardt Institute of Molecular Biology Russian Academy of Sciences, 119334 Moscow, Russia; giedre@inbox.ru (E.S.K.); dfedoseeva86@yandex.ru (D.M.F.); alembeki@gmail.com (I.R.A.); gk@eimb.ru (G.I.K.); vladimir_chechet@mail.ru (V.R.C.); jiri@eimb.ru (Y.V.K.); ovkretova@mail.ru (O.V.K.)

**Keywords:** rDNA clusters, 4C, gene ontology, chromatin marks, epigenetics, development, differentiation, heat shock

## Abstract

Chromosomes are organized into 3D structures that are important for the regulation of gene expression and differentiation. Important role in formation of inter-chromosome contacts play rDNA clusters that make up nucleoli. In the course of differentiation, heterochromatization of rDNA units in mouse cells is coupled with the repression or activation of different genes. Furthermore, the nucleoli of human cells shape the direct contacts with genes that are involved in differentiation and cancer. Here, we identified and categorized the genes located in the regions where rDNA clusters make frequent contacts. Using a 4C approach, we demonstrate that in *Drosophila* S2 cells, rDNA clusters form contacts with genes that are involved in chromosome organization and differentiation. Heat shock treatment induces changes in the contacts between nucleoli and hundreds of genes controlling morphogenesis. We show that nucleoli form contacts with regions that are enriched with active or repressive histone marks and where small non-coding RNAs are mapped. These data indicate that rDNA contacts are involved in the repression and activation of gene expression and that rDNA clusters orchestrate large groups of *Drosophila* genes involved in differentiation.

## 1. Introduction

The spatial organization of chromosomes is important for the epigenetic regulation of gene expression. Current research in this area mostly refers to intra-chromosomal contacts that are organized into topologically associating domains (TADs) and into smaller sub-domains that reflect promoter–enhancer interactions within loops [1,2]. It was suggested that there are also interactions between TADs (TAD–TAD interactions) that shape large-scale intra-chromosomal structures that are modulated both in response to external stimuli and during differentiation [3]. Some of these TAD-TAD sets are constitutive while other sets are variable [3]. Chromosome conformation capture techniques provide evidence for such inter-chromosomal interactions. This type of interaction is estimated to be much rarer than intra-chromosomal contacts and occurs between chromosomal regions possessing highly expressed genes, while intra-chromosomal contact regions are often enriched with epigenomically similar domains [2,4].

Inter-chromosomal contacts in *Drosophila* have been known for many years because it is possible to observe them microscopically between polytene chromosomes. The frequent contacts of nucleoli with specific regions in different chromosomes were described by Ananiev et al. in 1981 [5]. Pairing between alleles of the homologous chromosomes—i.e., transvection, which leads to the epigenetic activation or repression of genes—was discovered by Edward Lewis at the *Bithorax* complex in *Drosophila* in 1954 [6].

Currently, Hi-C or 4C techniques have revealed numerous contacts of nucleoli with different chromosomes in human cells [7,8]. In 4C-rDNA experiments, rDNA clusters form frequent contacts with numerous genes involved in differentiation [8,9,10,11]. However, the mechanisms behind this type of orchestration of gene expression are not yet clear. The nucleoli are membraneless organelles that can diffuse in the nucleoplasm and, currently, liquid–liquid phase-separation mechanisms are thought to be involved in the assembly and functioning of nucleoli [12,13]. Therefore, it is of interest to study the genome-wide contacts of rDNA clusters in the *Drosophila* genome using a 4C-rDNA approach.

Here, we show that nucleoli in S2 cells shape the frequent contacts with genes involved in chromosome organization, system development, neuron differentiation, and other biological functions, as well as with mobile elements. Heat shock treatment leads to reorganization of these contacts and induces upregulation of one set of rDNA-contacting genes and downregulation of another set of such genes. These data suggest the functional significance of rDNA contacts and a role for nucleoli in the global regulation of gene expression.

## 2. Materials and Methods

### 2.1. 4C Procedure

DNA samples were prepared as described previously [14,15]. To a suspension containing about 34 × 10^6^ S2 cells in 40 mL of Gibco medium, formaldehyde solution was added for a final concentration of 1.5%. After mixing, incubation of cell suspension was performed for 10 min at room temperature with mixing. Quenching with 2.75 mL of 2 M glycine (final concentration 0.125 M) was performed. After incubation at room temperature for 5 min the suspension was cooled for 15 min in an ice bath and then cells were collected by centrifugation for 15 min at 3500 rpm at 2 °C. The pellet of cells was resuspended at 0 °C in 1 mL of buffer containing 10 mM Tris-HCl buffer, pH 8, 10 mM NaCl, 0.2% NP-40, and freshly added protease inhibitors (0.1 mM PMSF (phenylmethylsulfonyl fluoride) and 1:500 protease inhibitor cocktail (Sigma Aldrich, Beijing, China)). After incubation for 15 min, cells were homogenized by passing through a syringe about 50 times into an Eppendorf. Then nuclei were spun down by centrifugation for 5 min at 5000 rpm in an Eppendorf centrifuge 5415R (Eppendorf Centrifuge 5415 R, Hamburg, Germany) at 2 °C.

The nuclei pellet was resuspended in 756 μL of solution containing 40 mM Tris-HCl buffer, pH 7.4, 50 mM NaCl, 10 mM MgCl_2_ and 10 mM 2-mercaptoethanol. Then 20% SDS (Sodium Dodecyl Sulfate, Calbiochem, CA, USA) was added to final concentration 0.3% and incubation with shaking was performed for 1 h at 37 °C. To sequester SDS, 180 μL of 10% Triton X-100 was added and the solution was incubated for 1 h at 37 °C. Before digestion, 1 μL of BSA (Calbiochem, CA, USA) (5 mg/mL) was added with mixing. Then 50 μL of EcoRI (10 u/μL) was added and after mixing digestion was performed overnight at 37 °C.

To inactivate the restriction enzyme, 35 μL of 20% SDS was added (final concentration 0.7%) and the probe was heated to 65 °C for 30 min. Then the mixture was transferred into a 15 mL Nunc tube and consequently 375 μL of 20% Triton X-100 (to final concentration 1%), 750 μL of 10× ligase buffer, 7.5 μL of BSA (5 mg/mL), 80 μL of 100 mM ATP (Promega, WI, USA), and 5241 μL of milliQ water were added and the final 7.5 mL solution was well mixed. Then 10 μL of T4 DNA ligase (200 u/μL) was added and after mixing incubation was performed for 5 h at 16 °C and then for 30 min at room temperature. During ligation, DNA concentration was equal to 2 ng/μL.

For isolation of DNA, 50 μL of proteinase K (10 mg/mL) was added (final concentration 50 μg/mL) and, after mixing, incubation was performed at 55 °C overnight. For RNA digestion, 40 μL of RNase A (10 mg/mL) was added (final concentration 0.5 μg/mL) and, after mixing, incubation was performed for 30 min at 37 °C. After extensive extraction with phenol-chloroform extraction (three times with 7 mL each) DNA was precipitated by 2.5 vol. of ethanol after addition of 40 μL 10 mg/glycogen and 175 μL 4 M NaCl. The final DNA pellet was washed twice with 70% ethanol and then dissolved in 0.1 × TE.

About 15 μg of DNA was digested in 250 μL solution with 75 u of FaeI overnight at 37 °C. Then the enzyme was inactivated by heating at 65 °C for 30 min. DNA was isolated after phenol-chloroform extraction, precipitated by ethanol, and dissolved in 100 μL of 0.1 × TE.

For circularization, 15 μg of DNA was incubated in 8 mL of T4 DNA ligase buffer containing 400 u of T4 DNA ligase for 5 h at 16 °C. DNA was isolated after phenol-chloroform extraction, precipitated by ethanol, and dissolved in 50 μL of 0.1 × TE.

About 30 ng of DNA was used for PCR with primers: primer (+) 5′ ACTGTTCACGATGAACTTG 3′and primer (−) 5′ CTTGGGATTGTGAACTGAA 3′. The libraries were prepared using TruSeq RNA Sample Preparation Kit v2 (Illumina). The samples were sequenced using Illumina HiSeq 1500. The length of reads was set at 150 nucleotides.

### 2.2. 4C-rDNA Data Processing

4C-rDNA data were processed in the following way. The sequencing was performed using the Illumina system and decoded to FASTQ format using native Illumina Casava software, v. 1.8 (Illumina, CA, USA). In the first step, we checked the read quality by FastQC (https://www.bioinformatics.babraham.ac.uk/projects/fastqc/) and qualified it as very good (for almost all nucleotides at lengths below 200, the quality parameter Q was above 30). The next step was to remove the PCR primers (primer (+) 5′ ACTGTTCACGATGAACTTG 3′ and primer (-) 5′ CTTGGGATTGTGAACTGAA 3′) using Cutadapt v. 2.7 [16] with the following options: --trim-n –times = 5 --minimum-length 20 -q 26 -e 0.15 -g ACTGTTCACGATGAACTTG -g TTCAGTTCACAATCCCAAG -a CTTGGGATTGTGAACTGAA -a CAAGTTCATCGTGAACAGT. The sequences from the viewpoint region of the rDNA attached to *Eco*RI and *Fae*I sites were trimmed. As in our previous work [8], the best results for genome-wide 4C read mapping were produced by the BWA method mem so we applied BWA mem v. 0.7.17 for genome-wide alignments. Samtools v. 1.6 [17] was used to remove unaligned reads from the SAM file, to convert it to BAM format, to sort, and to call variants depending on possible differences (SNPs/INDELs) between the sequenced reads and the reference genome sequence used (dm6/BDGP.6.22.96). At the end of the processing, we obtained a table with the genome-wide coordinates of 4C-rDNA contacts, the number of reads per contact, and their sequences. The table was converted to the bedGraph format that could be further processed and analyzed in any common genome browser.

### 2.3. 4C-rDNA Sequencing Reproducibility Control

**Figure 1:**
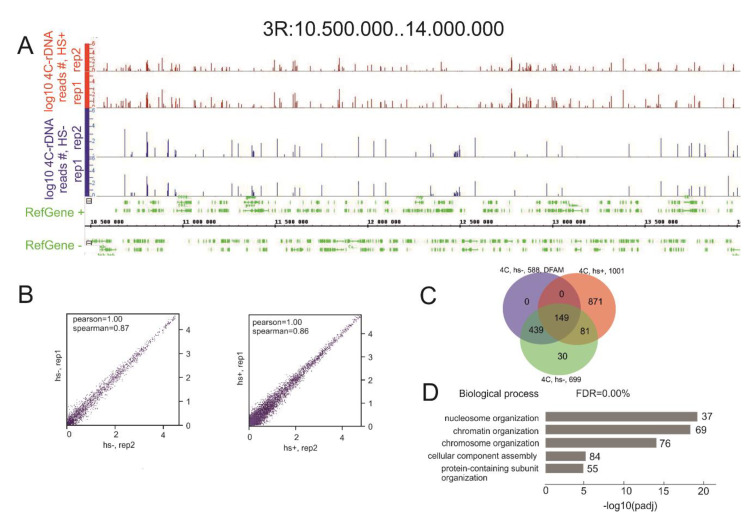
General characteristics of the mapped 4C-rDNA reads. (**A**) The position of rDNA contacts around the 500-kb region of 3R. The mapped reads without a threshold are shown for two replicates corresponding to the normal and heat-shock-treated S2 cells. The data for normal cells are shown in blue and for heat-treated cells in red. (**B**) Scatterplots show the consistency between the 4C-rDNA replicates for the normal (HS-) and heat-shock-treated S2 cells (HS+). (**C**) A Venn diagram shows the intersections between rDNA-contacting genes selected by the threshold of 100 4C-rDNA reads corresponding to a gene without Dfam filtering (699 genes), after filtering to remove the reads entirely corresponding to repetitive sequences (588 genes), and to rDNA-contacting genes detected after heat shock treatment (1001 genes). The list of the genes corresponding to the Venn diagram is shown in Table S1. GO associations of 149 rDNA-contacting genes indicated in the Venn diagram are shown in Table S2. GO associations of 588 rDNA-contacting genes are shown in Table S3. GO associations of 871 genes indicated in the Venn diagram are shown in Table S4. (**D**) The top five detected terms of biological processes associated with the list of 588 rDNA-contacting genes. The values to the right of the bars show the number of rDNA-contacting genes associated with a term.

To assess the reproducibility of the 4C-rDNA sequencing, all experiments were repeated twice (biological replicates). The BAM files with aligned reads for each replicate were analyzed by deepTools2 [18]. All files were RPKM normalized using the bamCoverage program with the options --effectiveGenomeSize 142,573,017--normalizeUsing RPKM --exactScaling --ignoreForNormalization X. The X chromosome was excluded from the normalization to eliminate the influence of 4C bait/anchor mapping on the calculations. Then, the plotCorrelation program was used to calculate the Pearson and Spearman correlation coefficients and to build scatterplot images (the options were --removeOutliers --skipZeros --log1p). The Pearson correlation coefficient was 1.00 both for 4C-rDNA untreated replicates and for 4C-rDNA heat shock replicates. The Spearman correlation coefficients were 0.87 and 0.86, respectively. The scatterplots are shown in the main text (Figure 1B).

### 2.4. 4C-rDNA Contact Detection and Gene Contact List Construction

We selected the most frequent 4C-rDNA contacts for further analysis as follows. First, we created the contact intersection list between replicates. The reads were assigned to the same 4C-rDNA contact if they intersected by at least one nucleotide. This intersection list was made from bedGraph files obtained previously using the BEDTools [19]: bedtools intersect -a 4C_dm0.rep1.bedGraph -b 4C_dm0.rep2.bedGraph -wb. An ad hoc Perl script was used to convert the BEDTools output file back to a bedGraph file with averaging in the overlapped regions. This bedGraph file was also filtered for low complexity, SINEs, LINEs, and other repetitive genomic elements by genomic coordinates subtraction with the Dfam database [20] by BEDTools with the following options: intersect -sorted -v -f 1.0 -a 4C_intersected.bedGraph -b dm6_dfam.hits.bed > 4C_nodfam.bedGraph. Only reads that completely mapped inside Dfam database entries were removed.

To select the most prominent 4C-rDNA contacts, a threshold for the mean reads with the value 100 was applied. In our 4C-rDNA experiments, we applied the six-cutter *Eco*RI enzyme and, thus, we assumed that the contacts were mapped at a resolution of ±2.5 kb. Therefore, the final filtered dataset was mapped onto the dm6 list of genes with each contact size extended to 5 kb using our own mapping pipeline that was created in Perl.

The required threshold for the selection of the mapped 4C-rDNA reads is described below (Statistical Calculations). We selected the genes possessing 100 or more corresponding reads and obtained a list of 588 genes (after Dfam filtering) that was used for the further studies (GO searches, ChIP-Seq profile plotting, 4C, and RNA-Seq differential analysis). Figure S1 shows that all chromosomes are involved in the contacts with the selected rDNA-contacting genes. 

### 2.5. Estimation of the Viewpoint Proximity Effect on 4C-rDNA Cis-Interactions

We examined the possible influence of the viewpoint (4C “anchor” or “bait”) proximity to our results. In the *Drosophila melanogaster* genome, rDNA clusters are located on the X chromosome (from 23.2 Mbp to the chromosome end) and the Y chromosome. The corresponding region in the Y chromosome is not yet included in the dm6 version of the genome. We applied the previously created script [8] to detect the number of the top 4C-rDNA-contacting genes (that mapped with a threshold of above 100 reads) in the vicinity of rDNA locations on the X chromosome and found that nearest 4C-rDNA-contacted gene to the rDNA cluster is more than 1.5 Mbp away (21.7 Mbp lncRNA:flam) and the next 4C-rDNA-contacting gene (20.6 Mbp RunxA) is 2.6 Mbp away. The result indicates that near-bait cis-interactions did not affect our results.

### 2.6. Differential 4C Analysis

As mentioned, 4C-rDNA contacts in our experiments were mapped at a resolution of ±2.5 kb. Therefore, we extended each 4C-rDNA mapped contact fragment to the length of 5 kb for the annotation of 4C contacts to the genes in the dm6 genome list. The dm6 genome annotation was obtained in the GTF format from Ensembl, release 96 for the dm6/BDGP6.22 genome build. Quantification of genes associated with 4C contacts was performed by featureCounts [21] with the following options: -a dm6.22.96.gtf -t gene -g gene_id -M --readExtension5 2500 --readExtension3 2500. The quantified genes list was analyzed by the DESeq2 R package [22] using two 4C replicates as the “control” and two 4C heat shock replicates as the “experiment”. We additionally assessed the replicate consistency at the gene level by creating scatterplot images in the built-in DESeq2 quality check system. We applied the parameter fitType = “local” for differential analysis to achieve more precise results for 4C data. Volcano plots were created by the EnhancedVolcano R package (https://www.bioconductor.org/packages/release/bioc/vignettes/EnhancedVolcano/inst/doc/EnhancedVolcano.html).

### 2.7. qRT-PCR

The relative expression of pre-rRNA was tested using total RNA samples isolated from S2 cells before and after heat shock treatment followed by recovery for 1 h. The expression data were normalized to the amount of 18S RNA. Primers corresponding to 18S RNA (5′ AGTTCATCGTGAACAGTTTCAGTTCACAA 3′) or the external transcribed spacer (5′ ATAAATTTAAAATTTATCTCTTTTCATATAA 3′) were used for the synthesis of single-stranded cDNAs. The qRT-PCR experiments were performed in triplicate using primers corresponding to the 18S RNA gene (5′ AGTGGAGCCGTACCTGTTGG 3′ and 5′ CGGTTTACCCGGACCTCTCG 3′) or to the external transcribed spacer sequence that is only present in the pre-rRNA (5′ GAACACGGGACTTGGCTCCG 3′ and 5′ ACATAAAACCGAGCGCACATGA 3′).

### 2.8. DNA-FISH Experiments

The fragments of *Drosophila melanogaster* rDNA and histone genes were amplified using total DNA isolated from S2 cells and LongAmp Taq DNA Polymerase (NEB). The following primers were used for synthesis of a 5.9-kb fragment of rDNA: 5′ TTCTGGTTGATCCTGCCAGTAGTT 3′ and 5′ ACCCGCGCTTACTTGAATTTCTT 3′. The following primers were used for amplification of a 1.36-kb fragment of histone repeats: 5′ CATCGGTTTCTGCTACCGCCA 3′ and 5′ CACGAATGCTCGCCTAACCCA 3′. The amplified DNAs were cloned, and sequenced clones containing the corresponding DNA fragments were used for the second amplification with the same primers. Finally, amplified rDNA was labeled with Alexa 555 and histone DNA with Alexa 650 using the BioPrime Total Labeling System (Invitrogen). S2 cells were seeded onto slides, rinsed with PBS, and then fixed for 15 min with buffer containing 10 mM PIPES, pH 6.8, 0.1 M NaCl, 1.5 mM MgCl_2_, 300 mM sucrose, 1.2 mM PMSF, 1% PFA, 2.5% Triton X-100, and 1× PBS. Then the cells were rinsed with PBS and treated for 15 min with hypotonic solution (30 mM KCl) followed by dehydration with 70% to 96% ethanol solutions and by RNase treatment (0.2 mg/mL). Chromosomal DNA was then denatured in 50% formamide in 2× SSC at 72 °C for 3 min. Hybridization was performed in a solution containing 50% (*v*/*v*) formamide, 2× SSC, 10% dextran sulfate, and 2 ng/μL salmon DNA at 42 °C for 24 h. After washing with 2× SSC at 42 °C, slides were mounted with Vectashield containing DAPI and imaged on a Leica TCS SP5 broadband confocal microscope and multiphoton system at 63X magnification under oil immersion. A collection of images was used for analysis of the results of hybridization of the rDNA and the histone probes and for quantitative population analysis of close localization of hybridization foci in individual cells.

### 2.9. Genome-Wide Profiles

Small RNA dm6 genome-wide profiles were downloaded as processed signals for two replicates from the GEO database: GSM2975537 and GSM2975538. The mean profile was calculated by wiggleTools [23] and then converted to bigWig format by UCSC genome tools [24]. Small RNA signals isolated from AGO1 and AGO2 immunoprecipitates of S2 cells were computed in the following way: raw data reads were downloaded from GEO GSM280087 (AGO2) and GSM280088 (AGO1) and converted from FQINT to standard FASTQ format by the fq_all2std.pl converter [25]. Then, adapters were removed by Cutadapt [16] with the following options: --trim-n –times = 6 --minimum-length 20 -q 22 -e 0.15 -g CGACTGGAGCACGAGGACACTGA -g GCTTTGCAGAGTCGAAGCTGATTG -g GGACACTGACATGGACTGAAGGAGTA -g CCGCTAGCTCTACCAAACTGGTGAT -g GTAATACGACTCACTATAGGGC –g AATTAACCCTCACTAAAGGG. Filtered reads were aligned to the *D. melanogaster* genome dm3/r5.32 by bowtie2 2.2.6 [26] with the preset –local, then SAMtools 1.6 [17] was used to remove unaligned reads (-F 4) and sort the resulting BAM file. Finally, the BAM file was converted to a bedGraph genome-wide profile by BEDTools 2.29.1 [19] and converted from bedGraph to bigWig using UCSC genome tools [24]).

Almost all available *D. melanogaster* epigenetic data were obtained by the ChIP-chip methods and processed data were available only for genome build dm3/r5.32 at http://www.modencode.org. We downloaded the following ChIP-chip-processed 500-bp-smoothed M values genome-wide profiles for S2 cells: GAF Kc11_GAF.1868 + kc12 GAF.1779, H3K27ac Kc11_H3K27Ac.1874 + Kc12 H3K27AC-AR-0105-50.1783, H3K27me3 S11_H3K27Me3.147 + S14 H3K27me3.377, dMi-2 A21_dMi-2(Q4443).1242 + A20_dMi-2(Q4443).1241, H3K4me2 S15_H3K4me2.641 + A12_H3K4me2.574, H3K4me3 Kc4 H3K4me3-ab8580.1759 + Kc11 H3K4Me3-ab8580.1772, H3K9ac B2_H3K9ac.249 + B5_H3K9ac.557 + B1_H3K9ac.247, H3K9me3 A18_H3K9me3(millipore).737 + A14_H3K9me3.678, H3K18ac H3K18ac (ART3 Elgin) + S12_H3K18ac.80. All downloaded files in the wiggle format were converted to the bigWig format using UCSC genome tools [24]. To compare our data with genome-wide profiles from ModEncode, we reprocessed all 4C-rDNA-contacts data with *D. melanogaster* genome dm3/r5.32 to exclude any possible discrepancies between genome annotations/builds.

Genome-wide profiles graphs were created by SeqPlots [27]. Venn diagrams were created by the website http://bioinformatics.psb.ugent.be/webtools/Venn.

### 2.10. Differential RNA-Seq Analysis

All RNA-Seq data analyzed in this study (accession number GSE145320) were processed uniformly by the following scheme. Trimmomatic [28] was used to filter low-quality reads with the following options: LEADING:18 TRAILING:18 SLIDINGWINDOW:4:22 MINLEN:20. Filtered reads were aligned to the dm6/BDGP.6.22.96 genome by the STAR RNA-Seq aligner [29] in two-pass mode. The package featureCounts [21] was used to quantify alignments to the dm6 Ensembl v.22.96 list of genes with the options: -a dm6.22.96.gtf -t exon -g gene_id *.bam. After that, the list of quantified genes was filtered using the threshold of more than 100 reads associated with 4C contacts (see above), which provided the list of 588 genes. Finally, this list of genes was used for differential RNA-Seq analysis using the DESeq2 R package [22] with heat shock treatment. The consistency of RNA-Seq replicates was assessed by built-in DESeq2 scatterplots. Volcano RNA-Seq plots were created using the EnhancedVolcano R package (https://www.bioconductor.org/packages/release/bioc/vignettes/EnhancedVolcano/inst/doc/EnhancedVolcano.html).

### 2.11. 4C-rDNA Contacts Annotations

4C-rDNA-contacts genetic annotation statistics for color pie plots were calculated by the Homer software package [30] using instrument annotatePeaks with the following Homer databases: fly-o v6.3 (*D. melanogaster* accession and ontology information), fly-p v5.5 (promoters), dm6 v6.4 (genome and annotation for UCSC dm6).

4C-rDNA-contacts epigenetic annotation statistics for color pie plots (Figure 8B) were calculated in the following way. Nine-state chromatin annotation for S2 cells was downloaded from ModEncode in GFF3 format for genome dm3/r5.54, accession modENCODE_3363. The in-house Perl script was used to split the integrated genome track into 9 tracks, each corresponding to the appropriate chromatin state, and to create genome tracks files in the BED format. Then, BEDTools [19] was used to calculate the number of intersections between the 4C-rDNA-contacts list (reprocessed for genome dm3/r5.32) and each of the chromatin states’ genome tracks (command lines like: BEDTools intersect -f 0.5 -a 4C_dm5_nodfam.intersect.bed -b dm5_3state.bed -wb|wc –l).

We assessed the difference between 4C-rDNA-contacts data per chromosome state and the randomized 9-state data in the following way. To preserve the intrinsic 9-state data structure, we performed random data shuffling using the following approaches: (a) leave chromosome coordinates as is and shuffle only states (this method leaves the genome-wide 9-state pattern intact as only states are shuffled while their proportions are intact); (b) shuffle segments of each state across the entire chromosome (this method leaves the order and amount of states intact); (c) shuffle both chromosome coordinates and states (this method retains the proportions of chromatin states and segment lengths genome-wide). Shuffling was performed using an ad hoc Perl script by the Fisher–Yates algorithm 10,000 times and, after each step, the annotation statistics with 4C-rDNA data were calculated. In addition, a statistical test of difference between proportions ([31], 8.7.2) was used to assess the difference between the calculated and shuffled data. We obtained a *p*-value << 10^−6^, which indicates that our results were not obtained by chance.

### 2.12. Statistical Calculations

The following procedure was used to estimate the standard deviation of randomly intersecting gene lists. The *D. melanogaster* dm6 gene list was obtained from the Ensembl dm6.22.96 annotation. Only unique names were left in the list by using the in-house Perl script. The size of the unique gene list was 17,748 gene names. The list was shuffled randomly once by the Fisher–Yates algorithm and the first 588 elements were chosen to be the reference list. At each step, we shuffled the list again randomly using the Fisher–Yates algorithm and chose the first 588 elements and then calculated the number of coinciding elements with the reference list. The procedure was performed 100,000 times. The basic statistics calculation was performed to estimate the mean value and normal deviation of the distribution and the following results were obtained. For the randomly generated list of 588 genes, the overlapping part = 0.03941 ± 0.007, min = 0.01860, max = 0.06295. In the most extreme case, we would have an overlap of 0.06295 (i.e., 6.3%). The observed replicate overlap among the 4C-rDNA gene lists without heat shock treatment: 83.56% for all data, 91.24% at threshold 40, and 92.48% at threshold 100. The observed replicate overlap among the 4C-rDNA gene lists with heat shock: 84.65% for all data, 94.32% at the threshold 40, and 94.61% at the threshold 100. These data indicate that the *p*-values were << 10^−6^ for the random selection of genes generated during the 4C-rDNA procedure thus ensuring that the analyzed gene lists did not intersect randomly at any level of intersection. 

## 3. Results

**rDNA clusters contact genes involved in chromatin organization.** The 4C-rDNA reads after trimming of sequences from the viewpoint region of the rDNA were mapped in the dm6 genome as described in the Materials and Methods. We used Illumina read lengths of 150 nucleotides to ensure the robustness of the mapping in the *Drosophila* genome. As expected, the processed reads possessed *Eco*RI and/or *Fae*I sites at their ends. To visualize the mapped 4C-rDNA reads, we used the IGB Browser.

Figure 1A,B shows that here is a good correlation between the replicates for experiments without heat shock treatment or after it. Heat shock treatment was used to test whether nucleoli contacts with different chromosome sites are physiological and could change upon stimulation. Nucleoli are key sensors of cellular stress and are involved in the response to different types of physiological challenges including heat shock treatment, hypoxia, pH fluctuation, and redox stress [12]. Heat shock leads to the disassembly of the nucleolus and affects rRNA transcription [12] and, thus, could affect the inter-chromosomal contacts of nucleoli. The 3.5-Mb region from the 3R chromosome possesses many contacts, and changes in the rDNA-contact pattern were observed after the treatment. The *Drosophila* genome is enriched with mobile and repetitive elements of different classes. Although the true rDNA contacts could correspond to these repetitive sequences, we filtered the 4C-rDNA reads using the Dfam database of repetitive DNA families [20] in order to remove the reads entirely corresponding to *Drosophila* repeats without any adjacent sequences coming from unique DNA stretches. 

Previously, microscopic analysis of nucleoli contacts with polytene chromosomes revealed the wide distribution of contact frequencies [5]. In this study, we characterized the most frequent rDNA contacts in the genome, as previously performed in the human genome [8]. That is why the mapped reads exceeding 100 contacts were selected and genes located within ±2.5 kb around such contact sites in the normal cells and in cells after heat shock treatment were identified. In this way, we selected 588 genes near rDNA-contacting sites. Without the Dfam filtering, 699 genes were detected in this way from S2 cells that were not subjected to heat shock treatment and 1001 genes after treatment. The overlap between these three groups of genes is shown in Figure 1C and Table S1. The groups of 81 and 30 genes in the Venn diagram correspond to rDNA contacts with repetitive sequences because they were subtracted by Dfam filtering and, thus, are not present in the group of 588 genes. The heat shock treatment leads to redistribution of the rDNA contacts. However, the group of 149 genes that do not correspond to genomic repeats retained their contacts with rDNA after heat shock treatment. Table S2 shows that these genes revealed extremely high associations (up to 3.6 × 10^−48^) with a number of Gene Ontology (GO) items relating to the nucleosome level of chromatin organization, including histone genes. High correlations with particular GO groups were observed for the selected 588 genes (Table S3). The top five groups of these genes are associated with nucleosome organization, chromatin organization, chromosome organization, cellular component assembly, and protein-containing subunit organization (Figure 1D).

Interestingly, the group of 871 genes shown in Figure 1C includes 36 genes corresponding to another set of particular histone genes that do not overlap either with histone genes in the group of 588 genes lacking repeats or with the group of 699 genes with repeats (Table S4). These data suggest that heat shock treatment induces a shift of rDNA-contacting sites within the cluster of histone genes in chromosome 2L or changes the frequencies of these contacts. Among this group of 871 genes, there are also 12 *Ste* genes (involved in the regulation of protein serine/threonine phosphatase activity), 76 *lncRNA* (long non-coding RNA) genes, *Df31* (encodes a histone-binding protein, which is involved in nucleosome assembly), the *mod(mdg4)* gene (encodes a nuclear protein that specifically interacts with various DNA-binding proteins), and several genes specifying co-regulators of transcription (*Taf11*, *kis*, *tara*, and others). These data show that heat shock stress causes changes in the pattern of nucleoli contacts in the genomic regions where genes controlling chromatin organization reside.

**Figure 2:**
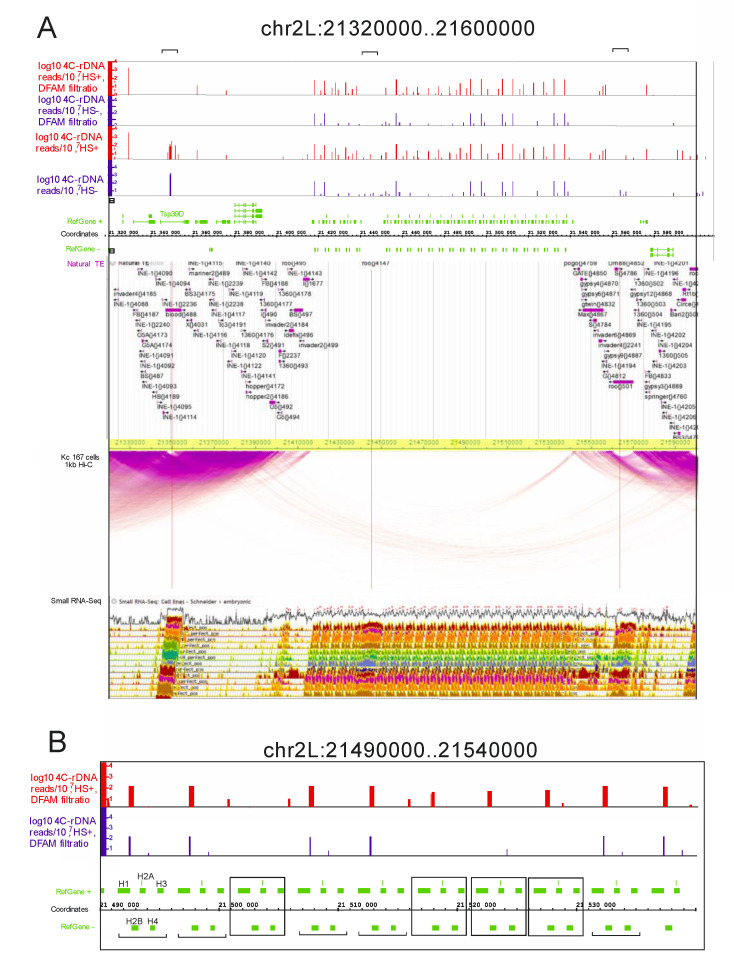
rDNA-contacting sites inside histone gene clusters. (**A**) rDNA contacts are shown in the IGB Browser for the mapped reads without Dfam filtering and after the filtering. The contacts in the normal and heat-treated cells are shown in blue and red, respectively. The distribution of natural transposable elements and small RNAs are indicated as in the FlyBase Browser. The intra-chromosomal contacts detected in Kc 167 cells are shown as in the WashU EpiGenome Browser (https://epgg-test.wustl.edu/browser/). (**B**) The rDNA contacts inside a 50-kb region of the histone gene cluster are shown in more detail. Two sets of histone repeats are indicated by brackets (the contacts detected in both HS− and HS+ cells) or by frames (the contacts detected only in HS+ cells).

**Heat shock treatment intensifies the nucleoli contacts within histone gene clusters.** Next, we studied the rDNA contacts inside histone gene clusters in more detail. Figure 2 shows the 280-kb region from chromosome 2L that possesses a histone gene cluster, which is located in the center of the region.

There are many transposable elements of different classes that are found mainly on the flanks of the region. At the top of Figure 2A, the Dfam-filtered results corresponding to the rDNA contacts in the heat-shock-treated and untreated cells are shown. There are many rDNA-contacting sites in the region corresponding to a histone gene cluster. The number of individual contacts varies and does not always coincide between the normal and heat-shock-treated cells.

The results are shown in more detail in Figure 2B. Surprisingly, better resolution revealed that heat shock treatment induces the appearance of new contacts. The distribution of the contact sites varies between different histone repeats. One set of repeats has a similar distribution of rDNA-contacting sites before and after the treatment; these contact sites are located after the *H1* gene. However, other copies of the histone repeat acquire the contacts only after the heat shock treatment. These data confirm our conclusion drawn from the analysis of the Venn diagram shown in Figure 1 that new rDNA contacts appear after heat shock treatment. The result is partially due to the selection of sites possessing 100 or more mapped reads. Nevertheless, the data indicate that within the histone gene cluster, which spans about 130 kb, there are changes in the rDNA contacts inside rather small regions. Surprisingly, this indicates that the area of contacts of large organelles, such as nucleoli, with a particular chromosome region may be localized.

The Hi-C data, with 1-kb resolution and detected in Kc 167 cells, are shown in Figure 2A. There is a gap in the intra-chromosomal contacts corresponding to a histone gene cluster. It is not clear whether this is because the mapping of Hi-C reads is hampered by the presence of histone repeats or if the histone gene cluster that is enriched with rDNA contacts is free of intra-chromosomal contacts. An argument in favor of the latter follows from the fact that the dense intra-chromosomal contacts were mapped by the same procedure on the flanks of the histone gene cluster, which are enriched with transposable elements. In these dense regions, two “bubbles” are observed that correspond to *Blood* and *Roo* LTR-elements on the left and right flanks, respectively.

There is one more *Roo* element that resides inside the histone gene cluster. This element, as well the *Blood* and *Roo* elements located on the flanks of the cluster, exhibits rDNA contacts that disappear after Dfam filtering. This result again demonstrates that mobile elements are the targets in rDNA contacts. Three LTR-elements and the region of the histone gene cluster itself are the sites where small RNAs are detected in S2 and embryonic cells (Figure 2). The *H1* gene in S2 cells is transcribed throughout the S phase, while the core histone genes are only transcribed in a short pulse during early S phase [32]. It follows that the histone cluster is silenced and small RNAs and rDNA contacts could be involved in the mechanism of this silencing. Our data on the dynamics of rDNA contacts within the histone gene cluster upon heat shock treatment support this view.

**DNA-FISH experiments confirm the contacts between rDNA units and histone genes.** 4C-rDNA data demonstrate the existence of contacts between nucleoli and histone genes. In these experiments, we used a bulk of S2 cells, which is why we next determined whether these contacts are stable and could occur either in every S2 cell or in some portion of the unsynchronized cells. To address this question, we used two-color DNA-FISH.

**Figure 3:**
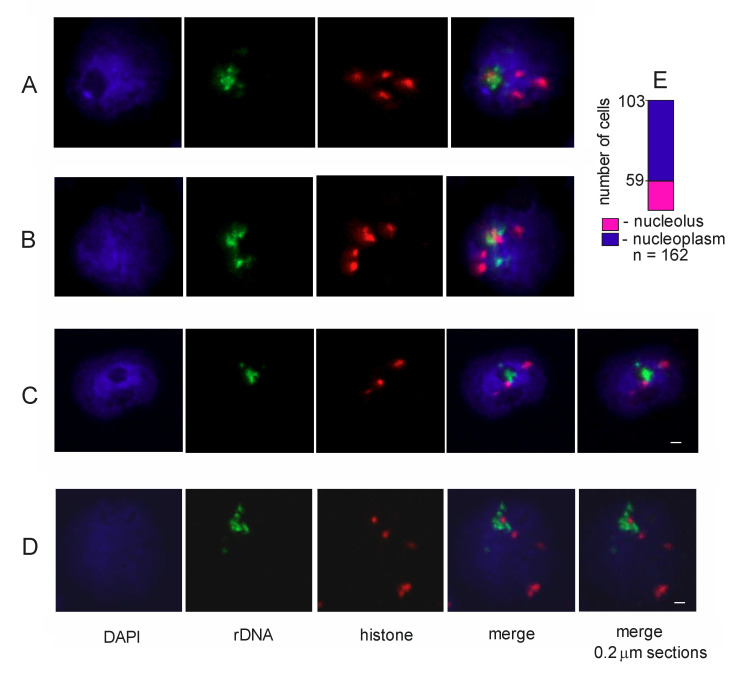
Two-color DNA-FISH with rDNA and histone gene probes. (**A**–**D**) Nucleoli demonstrating colocalization of rDNA units (Alexa 555) and histone genes (Alexa 650). The images shown here were cropped from populations of cells to highlight the localization of foci in a single cell. (**C**,**D**) Nucleoli analyzed with 0.2-μm z-step sections. Scale bar: 1 μm. (**E**) The results of population analysis of the histone foci localized either in the nucleoplasm or at the nucleoli in 162 cells are shown by the bar.

Figure 3D shows that only one-third of the cells possess the inter-chromosomal contacts between rDNA units and histone genes. S2 cells are generally tetraploid [33], which is why we observed several foci for the histone probe. Interestingly, only one or two histone foci reveal contacts with the nucleoli (Figure 3). These data independently confirm the presence of contacts between histone genes and rDNA units and suggest that the contacts are dynamic and may be formed in a particular stage of the cell cycle. Currently, we are studying these contacts in more detail by DNA-FISH using synchronized S2 cells and normal diploid *Drosophila* cells.

**Figure 4:**
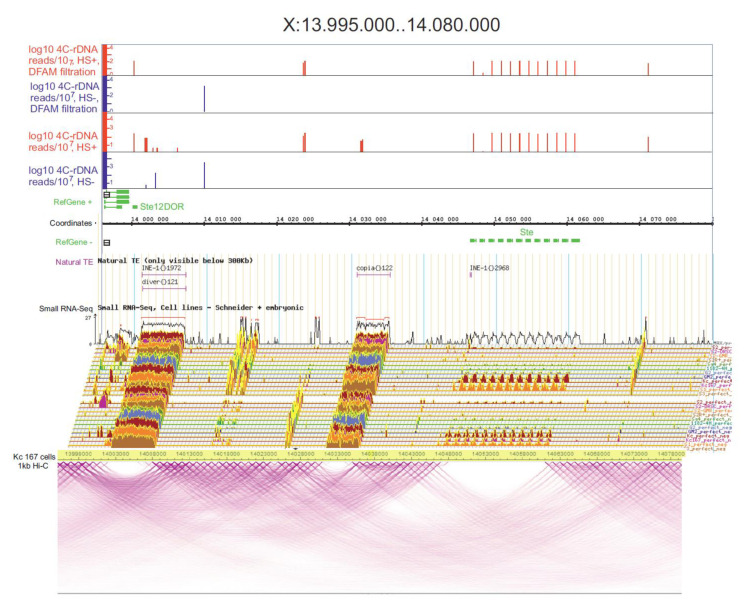
rDNA-contacting sites inside the *Ste* locus. At the top, the rDNA contacts are shown in the IGB Browser for the mapped reads without Dfam filtering and after the filtering. The contacts in Table 167. cells are shown as in the WashU EpiGenome Browser (https://epgg-test.wustl.edu/browser/).

**Heat shock treatment induces nucleoli contacts with *Ste* genes.** Histone clusters demonstrated a shift in the rDNA contacts following heat shock treatment. The data shown in Figure 1C and Table S4 indicate that 12 *Ste* genes that did not shape the contacts with rDNA in the normal state are moved to the nucleoli after heat shock treatment. Euchromatin *Ste* genes, together with heterochromatin *Su*(*Ste*) genes, are involved in the maintenance of male fertility, reproduction, and reproductive isolation of *Drosophila melanogaster.* The cluster of *Ste* genes inside an 85-kb segment of the X chromosome is shown in Figure 4. 

No contacts of rDNA genes with *Ste* genes were detected in the normal cells even without Dfam filtering and the contacts were detected only after heat shock treatment. Again, similar to the results obtained in the histone gene cluster, there was a lack of *Ste* transcripts in both the normal and heat-shock-treated cells, and small RNAs exactly corresponding to the region of the *Ste* genes were detected. A “bubble” in the pattern of intra-chromosomal contacts was observed in the area where the *Ste* genes reside and coinciding with the region containing rDNA contacts. We suggest that nucleoli contacts with this gene cluster are required to ensure the silent state of *Ste* genes.

**Figure 5:**
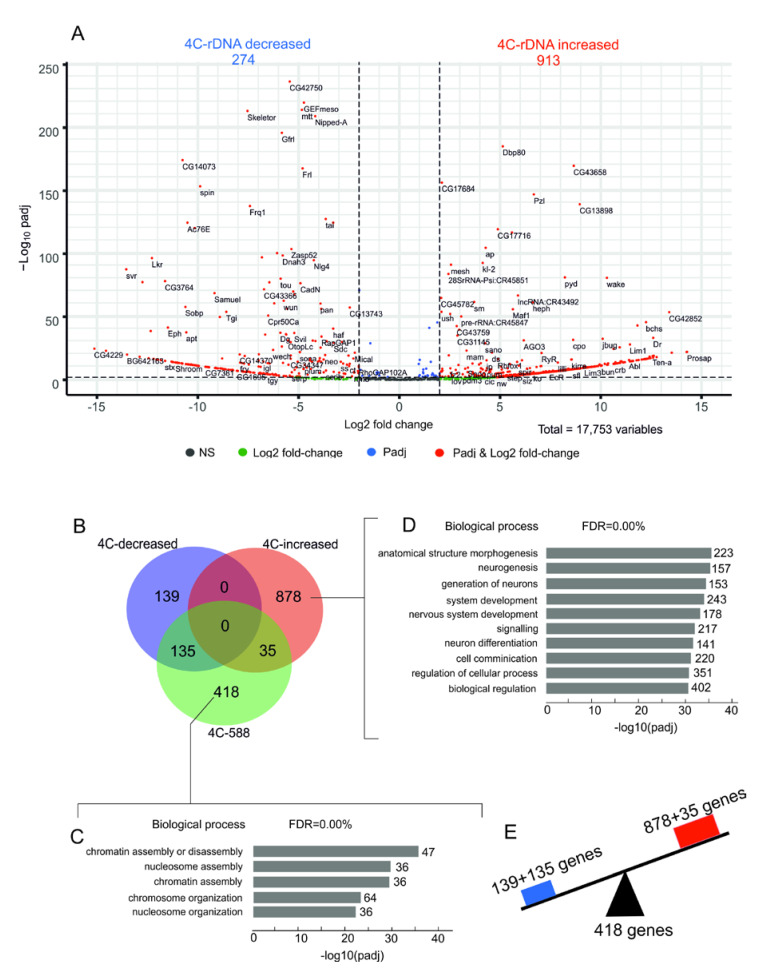
Analysis of changes in the contacts of genes with rDNA clusters after heat shock treatment. (**A**) The volcano plot presents the statistically significant log2-fold changes in contacts of genes with rDNA clusters determined in 4C-rDNA experiments. The list of 17,753 genes from the gene annotation for the dm6/BDGP6.22 genome build was used. Of these, 274 genes demonstrated a decrease in the number of contacts, while 913 genes revealed an increase in the number of contacts. The list of the corresponding genes ranked by padj is shown in Table S5. (**B**) The Venn diagram shows the intersections of these two groups of genes with the list of selected 588 rDNA-contacting genes detected in untreated S2 cells. GO associations of 418 genes indicated in the Venn diagram are shown in Table S6. GO associations of 135 and 139 genes indicated in the Venn diagram are shown in Tables S7 and S8, respectively. (**C**) The top five GO terms associated with 418 rDNA-contacting genes that retained their contacts with rDNA after the treatment correspond to genes that are involved in chromatin assembly. The values to the right of the bars indicate the number of corresponding genes. (**D**) The top ten GO terms associated with 878 rDNA-contacting genes that increased their contacts with rDNA after heat shock treatment and are associated with morphogenesis and development. The values to the right of the bars indicate the number of corresponding genes. The list of these genes is shown in Table S9. (**E**) The scheme illustrates that heat shock treatment induces an alteration of contacts between nucleoli and genes controlling morphogenesis (increase or decrease).

The genes involved in chromosome organization remain in contact with nucleoli while the genes that control morphogenesis show altered contacts after heat stress. The data on rDNA contacts within the histone and *Ste* gene clusters prompted us to perform a whole-genome analysis of changes in the nucleoli contacts induced by heat shock treatment using a volcano presentation. This type of visualization allows us to observe changes in the contact frequencies of nucleoli with different genes. Figure 5B shows the statistically significant results of the differential 4C-rDNA analysis for all detected rDNA contacts in the genome without a threshold of 100 reads that were used before (Figure 1C). Dfam filtering of the 4C-rDNA reads was used to remove the contacts with transposable elements.

We observed that 274 rDNA-contacting genes decreased the number of contacts with rDNA while 913 genes increased the number of contacts. Among these latter genes are those that did not have contacts with nucleoli before the heat shock treatment, e.g., *Ste* genes. The complete list of the genes is shown in Table S5. The list of genes from the 4C differentiation analysis was compared with the previously selected list of 588 genes (Table S1) that revealed 100 or more contacts with nucleoli. The Venn diagram in Figure 5B shows that the majority of genes (418 out of 588 rDNA-contacting genes) retained their contacts with rDNA upon heat shock treatment and corresponds to genes involved in chromatin assembly (Figure 5C, Table S6). Simultaneously, two groups of non-overlapping genes (135 and 139 genes) shown in the Venn diagram in Figure 5B decreased their number of contacts with nucleoli. Both groups are highly associated with morphogenesis, organ development, and differentiation (Tables S7 and S8). Among the genes that decreased the number of contacts are the homeotic *ct* and *Abd-B* genes. It is of interest that the largest group of genes (878 genes in the Venn diagram in Figure 5B) is also associated with morphogenesis and development (Figure 5D and Table S9). These data indicate that heat shock treatment induces alterations (increase or decrease) in the contacts of genes controlling development with nucleoli (Figure 5E), while the genes responsible for chromosome organization retain the contacts.

**Figure 6:**
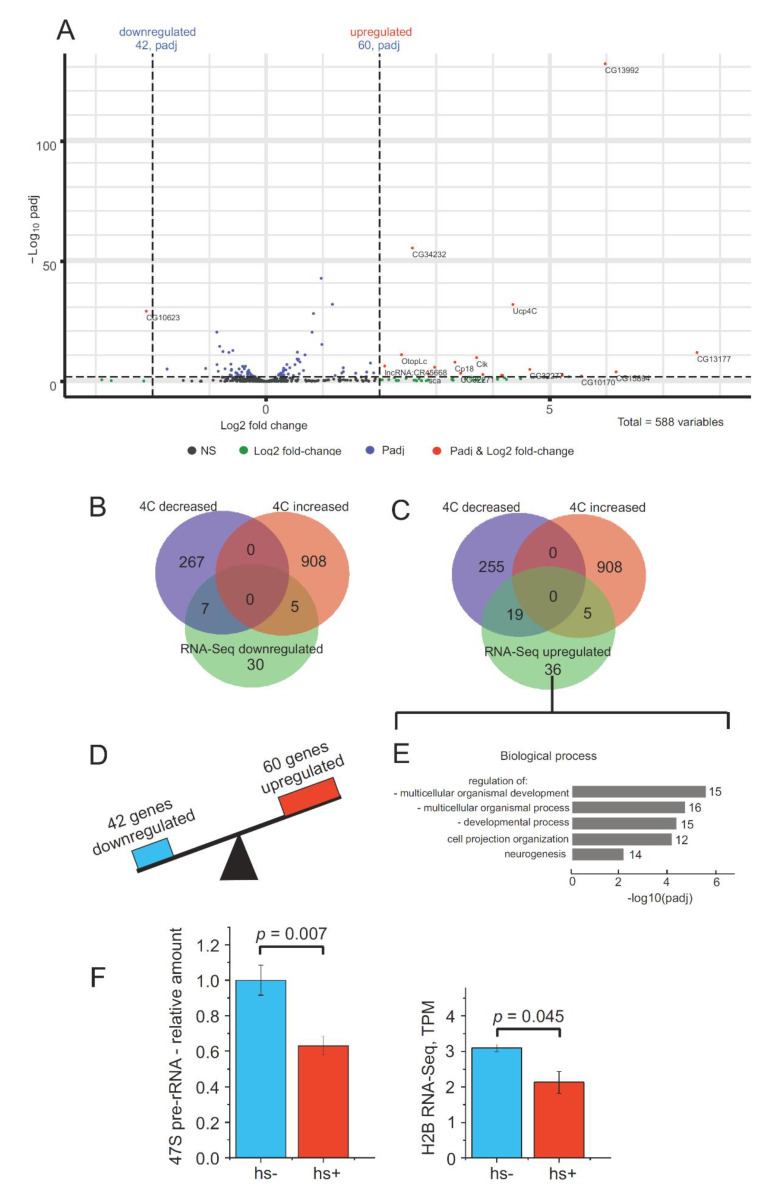
Differential expression of rDNA-contacting genes and rDNA units after heat shock treatment. (**A**) The volcano plot presents the statistically significant log2-fold changes in the expression of rDNA-contacting genes determined in RNA-Seq experiments after heat shock treatment. The expression of 588 rDNA-contacting genes was analyzed. Of these, 42 genes were downregulated while 60 were upregulated. The list of the corresponding rDNA-contacting genes ranked by padj is shown in Table S10. (**B**) The Venn diagram shows the intersections of downregulated rDNA-contacting genes with the lists of genes that revealed a decrease (274 genes) or increase (913 genes) in rDNA contacts as shown in Figure 4A. The list of the corresponding genes is shown in Table S11. GO associations of 42 genes indicated in the Venn diagram are shown in Table S12. (**C**) The Venn diagram shows the intersections of upregulated rDNA-contacting genes with the lists of genes that revealed a decrease (274 genes) or increase (913 genes) in rDNA contacts as shown in Figure 4A. The list of corresponding genes is shown in Table S13. (**D**) A scheme illustrating that heat shock treatment induces changes in the contacts with nucleoli of about 100 genes coupled with the downregulation or upregulation of these genes. (**E**) The top five GO terms associated with 60 upregulated rDNA-contacting genes and the regulation of development. The values to the right of the bars indicate the number of corresponding genes. The list of these genes is shown in Table S14. (**F**) Expression of rDNA and the histone H2B gene (CG33868) in S2 cells before and after heat shock treatment. qRT-PCR experiments showing the relative expression of pre-rRNA and RNA-Seq data for H2B gene expression are shown.

**A set of rDNA-contacting genes controlling development is upregulated after heat shock treatment.** To test the hypothesis that changes in contacts with nucleoli correlate with the changes in expression of rDNA-contacting genes, we analyzed the differential expression of rDNA-contacting genes after heat shock treatment (Table S10). Our data indicate that contacts with nucleoli do not correlate significantly with changes in expression and that most differentially expressed genes do not change their contact frequency. It is likely that a reversible change in 3D chromatin architecture upon heat shock treatment affects only a subset of rDNA-contacting genes. We found that 42 of 588 rDNA-contacting genes were downregulated while 60 genes were upregulated (Figure 6A). 

Seven downregulated genes revealed a decrease in the number of contacts with rDNA genes (Figure 6B, Table S11). Among these, two genes (*Sema1a* and *PlexA*) are involved in the semaphorin-plexin signaling pathway (Table S12). Only five of the downregulated genes showed an increase in the number of contacts and the remaining 30 downregulated genes did not alter their contacts with rDNA after the treatment.

In the group of upregulated genes, there are only five that increased the number of contacts with rDNA (Figure 6C, Table S13). Among them are the *cnc* (cap-n-collar) gene, which encodes a transcription factor that regulates the activation of genes by oxidative stress and is involved in dendrite morphogenesis. Another gene in this group is *chic*, which is involved in cell division and cellular morphogenesis.

Nineteen upregulated genes decreased the number of contacts with rDNA. The group includes several genes encoding transcription factors (*tai*, *Tgi*, *Glut4EF*, *slou*, and *cwo*) and the *Syp* gene that specifies an RNA-binding protein that is required for synapse morphology and synaptic transmission (Figure 6C and Figure S2, Table S13). The GO associations of the 60 upregulated genes that regulate development and contact the rDNA are shown in Figure 6E and Table S14. These genes regulate development and are involved in cell projection organization and neurogenesis. These data indicate that heat shock treatment induces an epigenetic switch that orchestrates gene expression by decreasing the expression of one set of rDNA-contacting genes while at the same time increasing the expression of another set of genes that regulate development (Figure 6D). These results demonstrate that the changes in the contacts with nucleoli of about 100 *Drosophila* genes are coupled with the downregulation or upregulation of these genes. However, only 36 of these genes change their contacts with nucleoli. It should be mentioned that other mechanisms induced by heat shock could regulate rDNA-contacting genes.

After heat shock treatment of S2 cells and recovery for 1 h, rDNA genes are downregulated (Figure 6F) to about 60% of the 47S pre-rRNA level. Inhibition of rDNA gene expression is likely connected to the observed changes in the contacts of rDNA genes (Figure 5A). Downregulation of the expression of rDNA units is coupled with a downregulation of the H2B histone gene, which specifies the core histone, by about 30% (Figure 6F). We suppose that the phase separation mechanism may occur via this coordinated inhibition of rDNA and histone genes and we are currently addressing this question.

**Heat shock treatment may induce transition of the nucleoli to epigenetically different regions.** Because there are changes in the rDNA contacts in response to heat shock treatment, we studied the profiles of histone marks, small RNAs, Ago1 and Ago2 complexes, and other factors within the genomic regions where the contacts were detected. We used the available data for the normal S2 cells and compared the profiles around rDNA-contacting sites in normal and heat-shock-treated S2 cells. As cells easily recover after a rather short heat shock procedure (1 h incubation at 37 °C followed by recovery for 1 h at 27 °C in our experiments), we assumed that the majority of epigenetic marks would not change. However, this assumption should be experimentally proved, because, for example, brief exposure of human cultured cells to hypoxia leads to an induction of H3K4me3 within hours [34]. Therefore, we studied the epigenetic states of the regions where rDNA contacts moved to after heat shock treatment in our experiments. Figure 1C indicates that among 1001 rDNA-contacting sites detected after heat treatment, 230 did not change. However, it is possible to check the putative difference in the contact regions of the remaining 871 sites.

**Figure 7:**
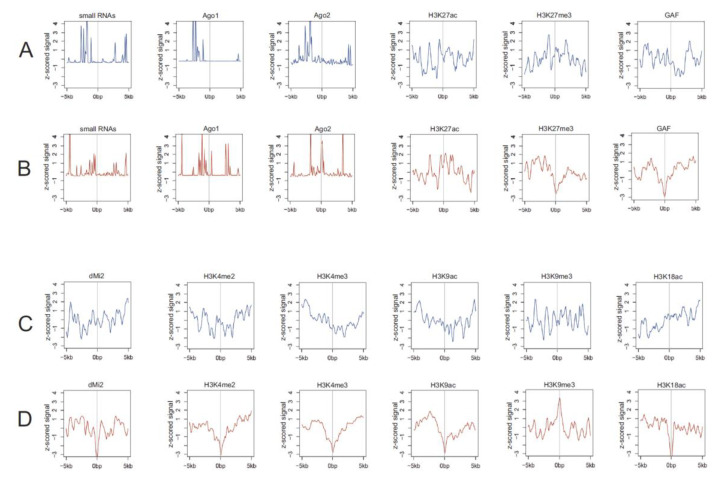
Profilers of small RNAs, Ago complexes, transcription factors, and histone marks around rDNA-contacting sites. The profiles shown in blue (**A**,**C**) and red (**B**,**D**) correspond to nucleoli contacts in normal and heat-shock-treated S2 cells, respectively.

We observed minor differences in the profiles for small RNAs and Ago1 and Ago2 complexes (Figure 7). Nevertheless, this suggests that the rDNA-contacting sites are near the target sites of Ago1 and Ago2 complexes, which are involved in heterochromatin formation [35]. No differences were observed for H3K27ac active chromatin marks. These data suggest that there is a prominent set of rDNA contacts within active chromatin regions. In contrast, clear differences were detected for the repressive mark, H3K27me3. The nucleoli after heat shock treatment move from *Polycomb* repressive regions to regions that are depleted of this mark and are probably active. This result confirms our previous data suggesting that nucleoli change their pattern of contacts after heat shock treatment (Figure 1, Figure 2 and Figure 4).

Another example that supports our view of dynamic rDNA contacts induced by stress comes from the comparison of profiles of the chromatic modifier GAF and the nucleosome remodeler dMi-2. After heat shock treatment, nucleoli shift from the sites enriched with these factors to other sites that lack them. The same is true for profiles of H3K4me2, which marks *Drosophila* PREs and maintains the developmental expression pattern of nearby genes [36], and the active histone mark H3K4me3, which promotes transcription initiation.

Interestingly, the active H3K9ac mark, which switches from transcription initiation to elongation [37], and the heterochromatin marker H3K9me3 have contrasting profiles (Figure 6). After heat shock treatment, rDNA escapes the subset of transcribed regions and moves to inactive or heterochromatin regions. A similar transfer of nucleoli occurs from the active regions marked by H3K18ac to inactive regions.

Taken together, these results independently confirm the above-described data that heat shock treatment leads to changes in nucleoli contacts. Although not all contacts were changed, the data shown in Figure 7 support this conclusion. Moreover, the search of profiles indicates that the epigenetic features of new contact sites induced by heat stress often correspond to repressed chromatin. The only exception is the transfer of rDNA clusters from the regions marked by HK27me3 to the regions depleted of this mark.

**Figure 8:**
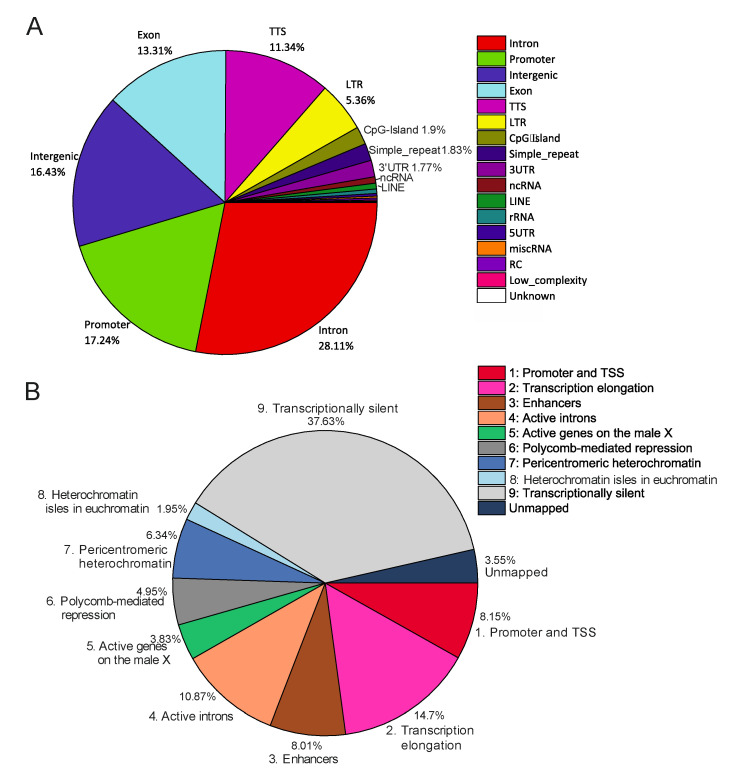
Genomic and epigenomic features at the nucleoli-contacting sites. (**A**) Distribution of rDNA-contacting sites in different portions of the *Drosophila melanogaster* genome. (**B**) Distribution of rDNA-contacting sites inside the different 9 major chromatin states (combinatorial patterns of 18 histone modifications) in S2 cells.

Alterations in rDNA-contacting sites induced by heat shock treatment do not change their proportions of genetic and epigenetic features. To better understand the properties of rDNA-contacting sites, we analyzed the distribution of rDNA-contacting sites in different portions of the *Drosophila* genome. Figure 8A shows that these sites correspond mainly to genes themselves. In reality, 71% of the rDNA-contacting sites correspond to promoters, exons, introns, transcription termination sites (TTS), and untranslated transcribed regions (UTRs).

Intergenic regions and LTR-elements comprise about 16% and 5% of the genome, respectively. It should be mentioned that the values were calculated for Dfam-filtered reads without the threshold of 100 reads per site, which means that at least 5% of 4C-rDNA reads were mapped at the very ends of the LTR-elements because they contained the attached unique sequences from the insertion sites. This class of transposable elements (TEs) comprises about 3.29% of all TEs [38]. In *Drosophila* 20% of the genome consists of TEs [39] and LTR-elements should make up about 0.7% of the genome. Therefore, these elements are about seven times or more enriched with rDNA contacts than expected from a random distribution of contacts throughout the genome.

A similar distribution of rDNA-contacting sites in different portions of the genome was observed after heat shock treatment (not shown), which was unexpected because the essential changes in the rDNA-contact sites were observed after the treatment (see Figure 1, Figure 3 and Figure 4). Therefore, the data suggest that the shift in the contacts takes place in the same proportions between the promoters, exons, introns, TTS, UTRs, and LTR-elements.

This conclusion is independently supported by the distribution of rDNA contacts among regions with different chromatin states (Figure 8B). To elucidate the epigenetic states at the nucleoli contacts, we used the search of determined the nine states of chromatin in S2 cells [40]. The results were similar for the contacts in normal cells (Figure 8B) and in the cells after heat shock treatment (not shown). It follows that changes in nucleoli contacts after heat shock treatment take place between regions possessing similar epigenetic states. About 44% of the contacts take place with transcriptionally silent or repressed chromatin. This value includes about 5% of the contacts corresponding to Polycomb-mediated repressed regions and heterochromatin isles in euchromatin regions. The regions corresponding to active chromatin comprise about 51% in total (promoters, transcription elongation regions, enhancers, active introns, and active genes on the male X chromosome). A comparison with the genome-wide distribution of the nine chromatin states for S2 cells suggests a non-random distribution of rDNA-contact sites (*p*-value < 10^−6^). These data suggest a role for nucleoli in both the epigenetic silencing and activation of genes.

**Figure 9:**
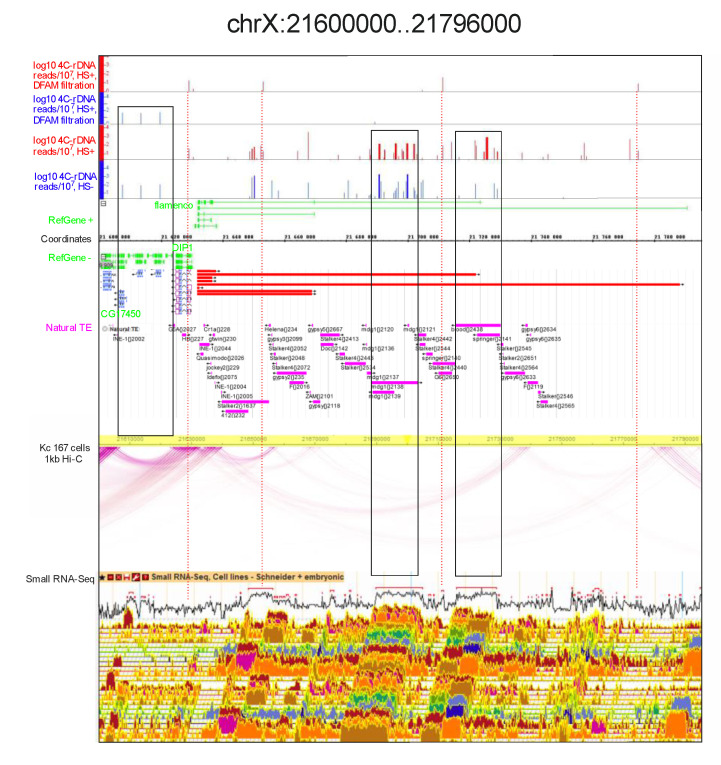
rDNA-contacting sites inside the *flamenco* locus. At the top, the rDNA contacts are shown in the IGB Browser for the mapped reads without Dfam filtering and after the filtering. The contacts in normal and heat-treated cells are shown in blue and red, respectively. The distribution of natural transposable elements and small RNAs are indicated as in the FlyBase Browser. The intra-chromosomal contacts detected in Kc 167 cells are shown as in the WashU EpiGenome Browser (https://epgg-test.wustl.edu/browser/). Long frames indicate two regions of rDNA contacts (without Dfam filtering) in the locus that correspond to *mdg1* and *blood* LTR-elements.

**rDNA contacts may correspond to gaps in the intra-chromosomal loops.**Figure 9 shows the detected rDNA contacts inside the *flamenco* locus. Dfam filtering removed almost all the mapped contacts corresponding to the locus. Nevertheless, three contacts occur in the locus only after heat shock treatment and these correspond to the sites targeted by small RNAs. 

Again, similar to that observed inside the histone gene cluster, there is a gap in the intra-chromosomal contacts corresponding to the entire *flamenco* gene and containing a cluster of LTR-elements (i.e., *mdg1*, *blood*, *Stalker*, *Doc*, and others). The corresponding Hi-C reads were either excluded from the analysis or it was impossible to map them. However, we observed a small gap in the intra-chromosomal loops in the region that does not possess any repeated sequences. There are several protein-coding genes (*CG32500*, *CG32819*, *CG32857*, *CG32820*, and *CG33502*) in this area, which are shown on the left flank in Figure 9. In normal S2 cells, this region has three sites of rDNA contacts, which disappear after heat shock treatment. The result suggests that the contacts of rDNA could affect the formation of intra-chromosomal contacts and, as a result, a gap is formed. Gaps in the intra-chromosomal loops were also observed inside the histone gene cluster and in the region corresponding to *Ste* genes (Figure 2 and Figure 4). In both cases, the gaps coincide with the regions where many rDNA contacts were detected. Taken together with the leftmost gap shown in Figure 9, these results support our supposition that contacts with nucleoli may prevent the formation of intra-chromosomal loops.

## 4. Discussion

The nature and mechanisms of inter-chromosome interactions are still unclear. In principle, there should be no differences between inter-chromosomal and long-range intra-chromosomal regulatory interactions. In both cases, interacting chromatin regions could share or shape some epigenetic regulatory elements. However, the main characteristic of intra-chromosomal interactions is that they are predominantly structural and are formed for the compaction of long chromosomal DNA into the limited space of the nucleus. This is why the number of detected inter-chromosomal interactions is two orders of magnitude weaker than the number of intra-chromosomal contacts [2].

In this study, we examined the most frequent contact sites of rDNA clusters in S2 cells and show that nucleoli form the most stable contacts with genes involved in chromosome organization. In contrast, the association of genes that control morphogenesis and development with nucleoli are altered after heat shock treatment (Figure 5). This mobility of the contacts argues in favor of their physiological significance. One set of genes controlling differentiation decreases the number of contacts while another non-overlapping set of genes increases them. There is an extremely high association (up to 10^−39^) of the latter set with morphogenesis and the generation of neurons (Figure 5D). S2 cells are derived from late-stage embryos [33] and after embryonic stages 16–17, there is a global downregulation of genes important for early neuronal development together with a global upregulation of genes necessary for the final differentiation of neurons [41]. We assume that the upregulation of rDNA-contacting genes involved in neurogenesis may reflect the origin of S2 cells from embryonic neural cells.

Heterochromatin condensation of rDNA clusters initiates the formation of repressed chromatin structures outside of the nucleolus coupled with the transcriptional activation of a set of differentiation genes [42]. The mechanisms of such global changes in gene expression triggered by rDNA clusters are not known. Nevertheless, the data suggest that nucleoli are involved in the epigenetic regulation of different genomic loci. One possible mechanism of this type of regulation is the formation of direct contacts between rDNA genes and different chromosomal regions. We observed that, upon heat shock treatment, a set of *Drosophila* genes involved in differentiation and morphogenesis moved close to nucleoli (Figure 5) and 60 of these rDNA-contacting genes controlling development were upregulated (Figure 6). This increase in contacts with nucleoli coupled with upregulation suggests a role for rDNA in the epigenetic regulation of a specific set of genes in S2 cells.

We speculate that the direct contact of active or silenced rDNA units with different genomic regions leads to the activation or silencing of the corresponding genes. Nucleoli, as membraneless organelles, could shape close interactions with particular genomic regions. Our data demonstrate that rDNA contacts are stable with some sets of genes (e.g., histone genes) and dynamic with other sets of genes (e.g., *Ste* genes). Putative trans-activation or trans-silencing produced by nucleoli is reminiscent of the mechanisms of transvection [6] and Y-linked genetic variation in *Drosophila* that modulate the expression of hundreds of genes in the genome [43].

We observed gaps in the intra-chromosomal contacts within the regions of histone gene repeats and the *Ste* locus contacting the rDNA. One possible explanation is that the mapping of Hi-C data is hampered in areas of genomic repeats. An alternative explanation is that the regions that are involved in inter-chromosomal contacts are excluded from the intra-chromosomal folding. The gaps corresponding to histone genes, the *Ste* locus, and to mobile elements (Figure 2 and Figure 4) are decorated by the mapped small RNAs that probably correspond to siRNAs and piRNAs. We found that LTR-elements comprise about 5% of rDNA-contacting regions (Figure 8) but at present, we cannot map the corresponding rDNA contacts. It cannot be excluded that mobile elements play some role in the inter-chromosomal contacts with nucleoli.

The nucleoli of human cells shape contacts with genomic regions that often possess strong and wide (up to 50-kb) H3K27ac marks, which are characteristic of super-enhancers [44,45]. In S2 cells, we detected an enrichment of this histone mark at the rDNA-contacting regions (Figure 7 and Figure S2). At present, the details of the functional anatomy of nucleoli are unknown and we do not understand mechanistically how chromatin loops of rDNA units could expand silenced or active states on the genes at the contact sites. Recently, it was suggested that super-enhancers are involved in phase-separation mechanisms [46,47]. Additionally, it was demonstrated recently that RNA promotes the nucleation and sizing of the phase-separated condensates [12,48]. We detected that the rDNA-contacting sites are enriched with both small RNAs and the binding sites of Ago1 and Ago2 complexes (Figure 7). Analysis of Ago2 profiles around rDNA-contacting sites at 42 downregulated or 60 upregulated genes (see Figure 6 and Tables S13 and S14) revealed distinct differences in the profiles of these two subgroups of genes. The data suggest that rDNA contacts and heat shock may regulate the activation or repression of the corresponding genes by modulating Ago2 complexes. This conclusion is supported by the recent observation of the involvement of Ago2 in developmental processes by controlling the 3D configuration of the chromatin regions [49]. Currently, we are analyzing individual rDNA-contacting genes from these lists.

We suppose that RNA-mediated mechanisms could be involved in the targeting of nucleoli to specific genomic regions. Taken together, these data argue in favor of the formation of micro-condensates and phase-separation mechanisms being involved in the targeted epigenetic trans-regulation of different *Drosophila* genes that contact the nucleoli. It is unclear how several nucleoli could shape contacts with hundreds of genes. It has been shown previously that eight *Drosophila* nucleoli formed contacts with eight different regions in polytene chromosomes [5]. We suppose that, after heat shock treatment, the disassembled nucleoli rapidly change the pattern of contacts with different chromosomes and that particular sites may differ in individual cells. Our 4C-rDNA procedure, which uses about 34 million cells, could lead to the detection of multiple sites coming from different cells. The current data on cell-to-cell variability support this possibility [50] and, thus, single-cell analysis of rDNA contacts and the study of phase-separation mechanisms could answer the questions raised by this study.

## Data Availability

For data availability, deep-sequencing data have been deposited in the Gene Expression Omnibus (GEO) repository with accession numbers GSE145321 and GSE145322 for 4C-rDNA and RNA-Seq data, respectively.

## Supplementary Materials

The following are available online at https://www.mdpi.com/2073-4409/9/12/2587/s1. Figure S1: The distribution of 588 selected rDNA-contacting genes on Drosophila chromosomes. The search was performed using the http://bioinformatics.sdstate.edu/go/ resource. All chromosomes possess rDNA-contacting genes. Figure S2: The rDNA-contacting sites often possess H3K27ac marks. Two examples are shown: a region of Tai genes in 2L (top) and a region of Tgi gene in 3L (bottom). Also, see H3K27ac profiles in Figure 6. Both genes specify transcription factors, are upregulated, and decrease the number of contacts with rDNA after heat shock treatment (see Figure 6C). Figure S3: Profiles of Ago2 complexes around rDNA-contacting sites at 42 downregulated and 60 upregulated genes (see Figure 6 and Tables S13 and S14). The profiles shown in blue and red correspond to nucleoli contacts in normal and heat-shock-treated S2 cells, respectively. Table S1: The overlap between the selected groups of 4C-contacting genes. Table S2: GO associations with Biological Process. Table S3: GO associations with Biological Process (GENERIC GENE ONTOLOGY (GO) TERM FINDER) of 588 rDNA-contacting genes shown in Venn Diagram in Figure 1C. Table S4: GO associations with Biological Process (GENERIC GENE ONTOLOGY (GO) TERM FINDER) of 871 rDNA-contacting genes shown in the Venn diagram in Figure 1C. Table S5: Changes in contact frequencies of DNA-contacting genes with rDNA clusters after heat shock treatment. Table S6: GO associations with Biological Process (GENERIC GENE ONTOLOGY (GO) TERM FINDER) of 418 rDNA-contacting genes shown in the Venn diagram in Figure 5B and in the diagram in Figure 5C. Table S7: GO associations with Biological Process (GENERIC GENE ONTOLOGY (GO) TERM FINDER) of 135 rDNA-contacting genes shown in the Venn diagram in Figure 5B. The genes decreased their number of contacts with rDNA. Table S8: GO associations with Biological Process (GENERIC GENE ONTOLOGY (GO) TERM FINDER) of 139 rDNA-contacting genes shown in the Venn diagram in Figure 5B. The genes decreased their number of contacts with rDNA. Table S9: GO associations with Biological Process (GENERIC GENE ONTOLOGY (GO) TERM FINDER) of 878 rDNA-contacting genes shown in the Venn diagram in Figure 5B and in the diagram in Figure 5D. The genes increased their number of contacts with rDNA. Table S10: The results of RNA-Seq before (HS-) and after (HS+) heat shock treatment of S2 cells for the selected 588 rDNA-contacting genes. Excel file attached separately. Table S11: The overlap between 274 4C-decreased genes, 913 4C-increased genes, and 42 downregulated genes. Related to the Venn diagram in Figure 6B. Table S12: GO associations with Biological Process (GO Profiler) of 42 rDNA-contacting genes shown in the Venn diagram in Figure 6B. The genes were downregulated after heat shock treatment. Table S13: The overlapping between 274 4C-decreased genes, 913 4C-increased genes, and 60 upregulated genes. Related to the Venn diagram in Figure 6*C*. Table S14: GO associations with Biological Process (GO Profiler) of 60 rDNA-contacting genes shown in the Venn diagram in Figure 6C. The genes were upregulated after heat shock treatment.
