# Supplementary material for: Dynamics of Whole-Genome Contacts of Nucleoli in Drosophila Cells Suggests a Role for rDNA Genes in Global Epigenetic Regulation
Source: Cells. 2020 Dec 3;9(12):2587. doi: 10.3390/cells9122587 (PMC7761670; doi:10.3390/cells9122587)
Supplement: Supplementary file 1 [file cells-09-02587-s001.pdf]

Supplementary Materials for

Dynamics of whole-genome contacts of nucleoli in *Drosophila* cells suggests a role for rDNA genes in global epigenetic regulation

Nickolai A. Tchurikov, Daria M. Fedoseeva, Elena S. Klushevskaya, Indar R. Alembekov, Galina I. Kravatskaya, Vladimir R. Chechetkin, Yuri V. Kravatsky, Olga V. Kretova

Nickolai A. Tchurikov

tchurikov@eimb.ru

This PDF file includes:

Tables S1 to S14

Figures S1 to S3

Table S1. The overlap between the selected groups of 4C-contacting genes: 699 genes that were selected without Dfam filtering, 588 genes that were selected after Dfam filtering, and 1001 genes that were selected after heat shock treatment (without Dfam filtering). All three groups exhibited 100 or more mapped 4C-rDNA reads at a gene. Related to the Venn diagram in Fig. 1*C*.

Names	total	Elements
1001 hs+ 699 hs- Dfam 588	149	28SrRNA:CR45844 CG33932 18SrRNA:CR41548 His1:CG33837 Hers His1:CG33846 pre-rRNA:CR45846 lncRNA:CR44999 CG4374 His1:CG33840 nAChRalpha3 CG45782 18SrRNA-Psi:CR41602 CG32277 CG33159 His4:CG33899 Myo81F His4:CG33885 Glut1 His1:CG33801 CG17716 Rpp20 His4:CG33877 Shaw His2B:CG33870 His2B:CG33910 SmydA-8 His3:CG33842 2SrRNA:CR45836 CR15821 28SrRNA-Psi:CR45851 His1:CG33849 His2A:CG33838 ckd CG13739 Ddr pre-rRNA:CR45845 Nipped-A His2A:CG31618 His3:CG33839 bchs His1:CG33861 18SrRNA-Psi:CR45861 CG43784 2SrRNA:CR45840 His2A:CG33841 CG18809 CG17684 mir-10404-1 His3:CG33818 sona snmRNA:430n snmRNA:430s COX6B sm Glut4EF His1:CG33816 Cp15 CG45493 His2A:CG33850 His2B:CG33888 JMJD5 His2B:CG33900 His4:CG33887 His4:CG33883 snmRNA:430l Gr61a His3:CG33836 snmRNA:430j 2SrRNA-Psi:CR45850 His2B:CG33880 CG15286 CR43383 CG32271 CR45495 28SrRNA:CR45837 CG12655 gry pre-rRNA:CR45847 His4:CG31611 asRNA:CR45501 CG5704 His3:CG33848 mir-10404-2 His4:CG33869 lncRNA:CR44874 snmRNA:430a lncRNA:CR43334 parvin His4:CG33909 His3:CG33803 mesh 18SrRNA:CR45841 His2A:CG33835 CR43377 snmRNA:430o fry 28SrRNA-Psi:CR45859 His3:CG33851 Mid1 His2A:CG33847 Frl His3:CG33845 His3:CG33863 lncRNA:CR43423 cnc His1:CG33834 His3:CG33815 Piezo sti CG33301 His2B:CG33878 CG12535 28SrRNA-Psi:CR40596 His1:CG33843 His2A:CG33862 Mct1 28SrRNA-Psi:CR45862 chic His2B:CG17949 His4:CG33879 snmRNA:430d His1:CG33819 His3:CG31613 His2A:CG33817 snmRNA:430k His-Psi:CR33802 CG15820 CG45487 18SrRNA:CR45838 CG33090 ban His2B:CG33884 2SrRNA:CR45843 CG42389 CG10019 CG13954 His3:CG33860 Mical GEFmeso cep290 His2B:CG33886 2SrRNA-Psi:CR45858 CG43366 CG6051 His1:CG31617 cpo pre-rRNA:CR45856 MESR3
699 hs- Dfam 588	439	Sry-delta CG42445 CG13344 Sry-alpha CR18166 sca CG34429 tou CG15237 CG5787 CG32459 corn CG31636 asRNA:CR43908 CG42340 Drep4 DNaseII His2A:CG33865 CG13203 prim CG34166 CG43965 ReepB CG30485 cdc14 CG8236 BG642163 CG42231 CG9005 Cyp309a2 c(3)G CG30484 CG3764 Cnot4 CG31633 HERC2 lncRNA:CR44544 Cerk lncRNA:CR44917 CG14115 lncRNA:CR45363 haf CG17768 CG9926 CG10654 His2B:CG33868 CG10621 CG15741 BtbVII ERp60 His-Psi:CR33867 CG9664 vih CG31021 mgl CG32373 Toll-7 Pvf2 His4:CG33901 CG31517 Calx CG31751 CG7289 CG6180 TTLL6B E(spl)mbeta-HLH CG1888 His2B:CG33902 CG14151 CG5984 CG9171 Syx18 tea CG3597 Kap-alpha1 asRNA:CR43465 fru THADA CG31019 Glt CG16986 eg CG17739 CG13983 Pbgs Gen CG5151 CG31211 CR43382 Strn-Mlck Mst36Fa CG10646 Tango4 CG32817 lncRNA:CR45222 ens Ucp4C CG3176 caps BBS1 CG17121 Sema1a insv Liprin-alpha CG42837 CG9016 Send1 lncRNA:CR44741 lncRNA:CR45735 TTLL3B bs Tgi Cp18 His1:CG33864 CG10413 CG33635 kek3 ste24a CR45033 CG13887 koi x16 CG10623 snoRNA:nop5-x16-a Tg CG43175 CG13685 CG10011 Pkn CG9986 inaE lncRNA:CR45039 CG13698 CG45105 Tom CG15258 red CG44243 CG30430 CG5756 CG31223 Frq1 Atpalpha scra apt hay Sms LanB1 CG8323 CR18275 MED25 CG15484 CG6220 Dap160 CG18273 bon CG8629 asRNA:CR45137 nsl1 Papss CG18327 CG14488 CG13992 stx CG44812 BTBD9 CG11103 fred Samuel CG9068 lncRNA:CR44184 CG4229 CG12923 snmRNA:430c kto CG44815 lncRNA:CR45037 CG42564 CR45498 AlaRS-m CG46310 mtt

		Nca CG6928 TfIIA-L lncRNA:CR44787 asRNA:CR44083 CG32369 CG42322 CG15211 CRAT Lkr asRNA:CR44416 smal Osi8 His3:CG33866 Nlg4 Npc2a CG14073 CG7943 mir-3643 wech Tango1 mir-2500 TTLL6A CG18635 CG4836 CG11319 Gabat CG13085 CG7381 CG42750 CG12173 CG10257 CG5098 Tom20 Sws1 E(spl)m6-BFM OtopLc OstDelta bnl CG34188 lncRNA:CR45580 CG34135 lncRNA:CR45460 betaTub97EF CG30008 nop5 spin CG10170 Itp-r83A Lsm12 Oda tRNA:Leu-TAA-1-1 CG14370 E(spl)m7-HLH Octbeta2R Shroom His1:CG33813 Pxn CG2082 CG31703 CG16762 pll CR45458 tai igl CG9663 eIF3g2 pico CG3436 CG34212 Rad1 trv CG15458 Zasp52 CG12121 CG1513 Hph Pop2 CG2186 RtcB Axud1 CG2157 Dic2 FoxL1 CG43886 Skeletor Nedd4 Acyp2 Irk1 His2A:CG33814 CG15705 CtsB1 CG42681 CG6362 Hrb27C CG12971 CG6767 Cad87A cuff CG13177 CG17190 CG4335 Atg8a CG18135 CG17068 CG15370 Obp19a Fbxl7 CG13837 Chd64 CG18324 CG3823 hb Rab9 slou CG31773 wun Cdk2 Eph Sry-beta Slob CG13164 toy CG15219 Nak snRNA:U3 PRAS40 RunxA CG46301 CG11560 CG43729 CG3609 shot CG30472 CG6511 CG32436 Srp54k chif sle Or43a Myo28B1 mir-2282 RapGAP1 CG17234 snmRNA:430t CG14005 CG15894 Nckx30C CG42536 CG10834 ubl cwo Aps CG34428 CG42587 AsnRS-m mrj lncRNA:CR44938 CadN dpr17 PlexA Sobp CG4074 lncRNA:CR45306 Gr8a fipi Shark lncRNA:CR45660 CG34001 lncRNA:CR44833 Prp8 CG45492 Ucp4B lncRNA:CR44811 CG15459 CG32152 CG1161 TpnC41C Got2 Galphai Clk CG16732 CG15829 Cpsf160 asRNA:CR44047 Dnah3 CG11768 Peritrophin-A CG10063 CG44085 sty lncRNA:CR44602 nAChRalpha7 lncRNA:CR45038 Traf4 lncRNA:CR32385 Drsl2 Sdc side CG7031 aPKC CG7755 CG1358 IA-2 atl spen CG13743 lov svr asRNA:CR44995 Prosbeta7 CCHa2-R MtnB Usp7 CG10286 CG17267 CG8628 RpL32 CG15186 asRNA:CR46256 Cyp28c1 AdSS mld CG9316 Gfrl pan CG42535 Tim17a2 asRNA:CR44368 Rab5 LSm-4 lncRNA:CR45668 Cpr50Ca CG10168 CG11453 lncRNA:CR44730 CG8366 chico Dg CG2246 tos ssp CG12592 CG10827 CG16771 Syp Ac76E vers ZnT63C CG13185 Sfmbt CG34232 Cyp305a1 Wsck NiPp1 Svil lncRNA:CR45459
1001 hs+ 699 hs-	81	Obp51a CG42329 His1:CG33810 sqa CG34393 His2A:CG33808 Teh1 cue CG14692 lncRNA:CR45347 CG14082 CG44153 CG40178 chinmo Maf1 plh Psa CG14693 His3:CG33812 dpy Ir10a 2SrRNA:CR45864 CR46152 PMCA hbs lncRNA:CR46123 CG6052 lncRNA:CR44452 CG43101 lncRNA:CR45336 CG41520 Ccn kirre asRNA:CR45139 Tsp39D CG43163 Plc21C CG42335 CG14459 pyd uex His4:CG33903 Hr4 Nmdar2 Parp lncRNA:CR45228 28SrRNA-Psi:CR45860 His4:CG33905 spir CG45781 Tlk lncRNA:CR46258 His-Psi:CR33811 WDY CG17839 His1:CG33807 asRNA:CR45367 Pzl Snap25 CG11883 dnt alpha-Man-Ia CG34354 CG14669 Gprk1 Ent1 His2B:CG33906 dpr6 sisA lncRNA:flam CG30116 His3:CG33809 tRNA:Ile-AAT-1-9 heph snmRNA:430b inc CG32850 His2B:CG33904 ush CG43254 lncRNA:CR44935
1001 hs+	871	CG15879 Pex19 lncRNA:CR46197 Dis3l2 eIF3f2 zfh1 CG10470 snmRNA:430g mir-932 Cp36 CG15824 lncRNA:CR45168 CG9003 28SrRNA-Psi:CR40741 CG7101 Tace zf30C His1:CG33852 CG43645 pre-mod(mdg4)-Z CG43221 asRNA:CR46109 CG13197 Cyp12c1 Cdc27 vtd lncRNA:CR42696 l(2)37Bb tRNA:Met-CAT-1-2 CG8950 Obp56e His3:CG33857 casp kl-2 CG5561 CG30268 Shab ft lncRNA:CR44997 Tao ACXB CG11617 cno CG4744 Gr85a CG13299 CG43200 GatA CR43378 CG6959 CG6901 kl-3 swm tRNA:Arg-TCT-1-1 bi CG43759 Cpr31A lncRNA:CR34047 lncRNA:CR44530 Ste:CG33242 His2A:CG33832 Mvl asRNA:CR45397 lncRNA:CR43492 CG42809 CG14892 Ptr 28SrRNA-Psi:CR45853 CG6621 egh His3:CG33806 His2A:CG33856 CG6967 Grip71 ninaA Top3alpha robo2 CG4839 dpr12 Naa30A Pdi Droj2 CG14044 His2B:CG33882 CG43054 Ste:CG33236 His2B:CG33898 CG12902 CG34105 CG34298 Orc3 CG18563 schuy CG32270 CG43088 Cypl lncRNA:CR45519 CG7029 Eip63E Ste:CG33244 lncRNA:CR44710 CG34432 beat-IIa CG5532 Gale CG7460 CG13877 Adgf-A2 nemy bab2 Pgcl lncRNA:CR32657 Prosap ex CG1273 snoRNA:Or-CD12 CG33160 CG1724 kis CG8870 Nox BG642312 hth CG14967 Csas CG3292 CG34200 iPLA2-VIA Ir48a CG13285 CG34426 Myo31DF CG32023 His1:CG33822 Dbp80 RpL28 CG6005 Cp38 His4:CG33875 lncRNA:CR46215 CG1407 Chmp1 CG43850 mim CG6144 GNBP1 Smurf CG33764 lncRNA:CR33938 Hs6st Nph CR43080 asRNA:CR45189 Cp19 CG34427 lncRNA:CR46249 CG42450 CG31145 jbug PIP82 Ccp84Ac asRNA:CR44592 fs(1)h snRNA:U2:38ABb Ste:CG33247 CG31050 rempA ppk12 His3:CG33854 CG43897 CG45486 TppII CG12773 CG5810 CG7058 His2A:CG33853 lncRNA:CR45374 CG3529 CG13810 CG45488 CG5969 bys lncRNA:CR46080 Jon65Aiv Sesn CG34402 His2A:CG33844 CG13297 CG40228 dpr8 Ste:CG33238 snmRNA:430p Tbc1d15-17 CG3987 NKCC mRpL24 CG15546 Gp210 GABA-B-R2 asRNA:CR43470 ap snmRNA:430q CG14340 EMRE Syx1A eRF3 tRNA:Ser-AGA-2-1 CR41320 CG14947 ValRS lncRNA:CR46106 CG42397 CG13405 NetB wake ds CG11123 sano CG34278 RluA-1 CG7692 Paf-AHalpha per CG8500 tRNA:Asn-GTT-1-3 CG42339 Df31 lncRNA:CR43485 pre-mod(mdg4)-L beat-Vc BicD loco CG33337 CG10189 CG43920 CCHa1-R CG42704 EcR Su(Ste):CR42414 CG10182 ND-MWFE Sgs1 Ten-a lncRNA:CR45971 His2B:CG33896 CG14285 His4:CG33889 CR32821 Eip75B Obp56f His2B:CG33894 CG7456 Su(Ste):CR42427 lncRNA:CR45321 lncRNA:CR46024 asRNA:CR44992 Act42A mdy Ac3 Ste:CG33246 pre-mod(mdg4)-G lncRNA:CRG CG8852 frm SPH93 His1:CG33831 CG4467 CG10096 RpL5 CG12945 Ady43A CG5045 His1:CG33804 lncRNA:CR43705 lilli CG15356 snmRNA:430e CG14339 CG8517 His4:CG33881 side-VIII His2A:CG33859 CG5664 CG13560 Nlg2 pre-mod(mdg4)-P DNAlig3 28SrRNA-Psi:CR45855 CG42810 IntS11 CG17180 CG2608 His4:CG33897 CG43110 His-Psi:CR33805 CG4631 CG1677 CG45064 SA-2 Ir48b Aef1 ben His3:CG33824 lncRNA:CR45808 GlyS His3:CG33830 Flo2 mir-4965 CG15570 CG42684 CG17279 mam CG34433 CG31659 Lcp65Aa lncRNA:CR45878 CG18735 mRpS21 Nup214 CG15147 Tsp2A CG31663 crb CG3842 pre-mod(mdg4)-C CG43171 Dhx15 snoRNA:Psi18S-1347b CG34051 asRNA:CR45396 CG30275 Cyp4s3 CG12541 CG15611 Cortactin His2A:CG33829 snama CG3909 lncRNA:CR44470 kay lncRNA:CR44844 asRNA:CR45161 ATPsynD Ccp84Ab Nup153 lncRNA:CR45256 His2A:CG33820 mRpL55 CG17490 pre-mod(mdg4)-AA IMPPP shakB CG15728 mira CG32202 lncRNA:CR44685 Kaz1-ORFB Tif-IA CG43672 His2B:CG33874 CG46305 lncRNA:CR45755 ko cta puf CG5050 CG30432 CG42705 pre-mod(mdg4)-J side-II lncRNA:CR45421 lncRNA:CR44860 lncRNA:CR44802 His4:CG33895 His3:CG33821 lncRNA:CR44143 Mur2B CG11210 PH4alphaPV DIP-zeta CR40450 amd CG8173 egl CG1468 sick CG34228 foxo CG33158 CG12491 Cpr65Ax2 CG13540 His2B:CG33892 CG17929 milt CG42733 tRNA:Ser-AGA-3-1 CG18368 CG4133 CG30060 Spindly CG42319 CG7142 AnxB9 lncRNA:CR43484 lncRNA:CR44594 PDZ-GEF ics CG17571 Cdk8 klar Uhg3 eIF1A viaf Corin Acp65Aa Grip75 sisRNA:CR46360 CG2652 MED30 Spn85F shv mir-2489 tRNA:Arg-ACG-1-2 dimm CG43178 CG40470 resilin CPT2 dpr14 CG43396 qin lncRNA:CR44528 Obp56d Rev1 pip CG13280 MTPAP Camta CheB42c CG11300 CG5246 mir-8 crq Alp2 Sp1

		ash2 28SrRNA-Psi:CR41609 CG42649 2SrRNA-Psi:CR40677 CG10348 CG34204 SNF4Agamma mAChR-C bbg Trxr-2 CG43201 Oatp33Eb Nhe2 Ste:CG33241 snmRNA:430r Atg5 lncRNA:CR43498 cher lncRNA:CR44909 sff Zpr1 lncRNA:CR44568 His1:CG33825 br lncRNA:CR43654 CG5367 CG9034 alpha-Cat Mbs snoRNA:Me28S-C2645a CG13397 Pde8 hale CG10344 CR40801 His4:CG33893 CG12147 Gr28b His4:CG33873 ord side-III wal CG42524 CR41423 His2B:CG33872 MED4 CG42779 Usp12-46 lncRNA:CR43904 btsz lncRNA:CR32773 CG33127 mei-P26 Prosbeta2R2 lncRNA:CR44494 NLaz kermit CG10803 lncRNA:CR46075 CG15255 Burs pre-mod(mdg4)-I lectin-24A dmrt93B udd nAChRbeta3 mRpL10 PNUTS CG42784 lncRNA:CR46268 Dlip1 CG30059 CG42269 lncRNA:CR44931 l(3)psg2 Rbp6 CG1116 Mdr65 lncRNA:CR45810 CG2225 mod(mdg4) CG16786 Grip163 LRP1 CG10809 smog ghi Cyp9f3 Ste12DOR CG32147 CG14234 CG17683 dpn Fs(2)Ket Hnf4 Grip CG7295 veil snoRNA:Me28S-C3351 CG31693 lncRNA:CR44991 CG32085 CG43095 ZnT77C CG13898 lncRNA:CR45232 snmRNA:430h lncRNA:CR45373 snoRNA:MeU6-A47 lncRNA:CR45750 His3:CG33827 Ptp99A adp CG32816 pre-mod(mdg4)-T CG34106 SK CG34315 lncRNA:CR43706 CG31266 His4:CG33891 Rpn2 CG43658 CG18179 kuz ps Ste:CG33237 tRNA:Lys-CTT-1-7 Gnpnat bora pre-mod(mdg4)-E lncRNA:CR45262 lncRNA:CR43838 lncRNA:CR44352 CG5421 28SrRNA-Psi:CR45848 Ste:CG33243 mnd mir-967 CG3402 lncRNA:CR44779 CG7548 tRNA:Asn-GTT-1-2 sIgA CG13135 tRNA:Met-CAT-1-1 Abl Dr Sulf1 lncRNA:CR46212 pre-mod(mdg4)-N CG46308 Tim17b lncRNA:CR45740 lncRNA:CR45809 lncRNA:CR45883 fat-spondin lncRNA:CR44593 pyx Cyp310a1 snRNA:U2:14B His1:CG33855 pre-mod(mdg4)-K CG15734 tara Dop1R2 CG14830 unc-13-4A aos CG34254 Mef2 CG32507 mir-4953 Lrrk lncRNA:CR44349 Leash 5-HT1A CG8399 CR45470 CG44838 CG9582 zormin ACXA firl Hipk tzn CG5131 His2B:CG33908 CG32024 CG8108 CG31125 pAbp snmRNA:430m CG33946 CG9173 Gbs-70E CG43896 CG13272 lncRNA:CR43650 RasGAP1 CG31206 snoRNA:Me28S-C2645c Adat1 snmRNA:430f CG42246 His4:CG33871 Gie asRNA:CR45600 lncRNA:CR46042 His2B:CG33890 r-l Pex3 CG4629 CG34351 CG3984 CG31013 CG15571 CG15545 cdi CG5565 CG7791 CG30460 lncRNA:CR46103 lncRNA:CR44075 ced-6 CR45496 Taf11 GLS Adgf-A I-2 CG12438 CG43172 eIF3c snoRNA:Psi18S-1347c beat-VI Roc2 betaggt-I His1:CG33828 lncRNA:CR44022 CG5522 CG18278 CG42852 tRNA:Asn-GTT-1-1 lncRNA:CR44443 rdog CG44532 CG5009 CG8329 CG42663 tRNA:Lys-CTT-1-6 Alp1 S-Lap4 CG3264 CG32944 CG32695 nonC beat-Ic CG11722 tRNA:Val-AAC-1-1 Kaz1-ORFA CngI X11Lbeta lncRNA:CR44621 lncRNA:CR44905 CG42326 bun mtRNApol pre-mod(mdg4)-AB Su(Ste):CR42428 CG33470 CG13110 CG45494 ReepA CG34353 Lpin CG6931 CG18810 CG12034 His2A:CG33826 AttC CG12128 CG15117 lncRNA:CR44241 Tsp96F CG14286 CG2818 Ste:CG33245 RluA-2 Ggamma30A CG32269 emb Src42A CG31267 CG8539 Ets21C beat-IIIa CG14125 Lim3 CG31224 CG45065 CG12994 CG13144 CG10226 lncRNA:CR44865 CG2233 CG43350 isoQC CG42321 cora Rbfox1 CG3655 Ste:CG33239 CG13312 Sox100B ND-51L1 COX4 His4:CG33907 CG15247 mtg snoRNA:Psi18S-1347a lncRNA:CR44875 tRNA:Phe-GAA-1-8 CheB42b His2B:CG33876 Cul4 lncRNA:CR44357 CG12426 His1:CG33858 Jupiter tral CG10178 Pino eIF4EHP CG43397 CR43384 Atf6 lncRNA:CR44413 Der-1 mAChR-B pre-mod(mdg4)-B lncRNA:CR45632 Acn phr6-4 MYPT-75D CG43673 Lim1 Adk3 CG31924 Fife CG33223 Oatp33Ea lncRNA:CR45267 hang Sgf11 CG14427 CG44044 CG34367 IP3K2 Wdr24 larp HLH54F Cht10 Taf12L Ste:CG33240 Trpm snoRNA:Me28S-C2645b Irk3 CG6912 qua CASK mir-9371 CG3448 Hlc snRNA:U5:38ABb SLO2 CR41508 pre-mod(mdg4)-H Asap CG3698 lncRNA:CR45754 HLH3B CG10097 lncRNA:CR43960 Pi3K92E CG32428 ACXC His3:CG33833 Cp16 Ser His2A:CG33823 CG1077 asRNA:CR44509 Sox21a drl Nrg CG7509 CG30431 olf413 lncRNA:CR42735 CG12898 CG11360 CG2611 CG31760 lncRNA:CR44993 CG43220 disco-r Prm CG16723 AGO3 Ptp61F Spc105R pre-mod(mdg4)-O GCS2beta Nlp rad50 phol snmRNA:430i cos RyR fln CAH1 CG5742 mir-4978 CG46307 CG12516
699 hs-	30	cic Drak CG30345 lncRNA:CR45615 CG13704 Ir41a RpL15 CG30383 Madm CG40813 CG43106 CG9766 CG12682 nolo CG15731 CG1882 lncRNA:CR45704 ORY CG8861 CG32365 pigs CG15927 CG43291 cathD ND-AGGG aqrs lncRNA:CR45703 scrib lncRNA:CR44674 lncRNA:bxd

Table S2. GO associations with Biological Process (GENERIC GENE ONTOLOGY (GO) TERM FINDER) of 149 rDNA-contacting genes shown in the Venn diagram in Fig. 1*C*.

Gene Ontology term	Cluster frequency	Genome frequency	Corrected P-value	FDR	False Positives	Genes annotated to the term
chromatin assembly or disassembly	41 of 146 genes, 28.1%	147 of 16085 genes, 0.9%	3.58e-48	0.00%	0.00	His3:CG33815, His1:CG33840, His4:CG33885, His3:CG33818, His2B:CG33884, His2B:CG33900, His3:CG33860, His1:CG33837, His3:CG31613, His3:CG33842, His4:CG33909, His1:CG33801, His3:CG33803, His4:CG33883, His2B:CG33886, His4:CG33877, His4:CG33869, His1:CG33834, His2B:CG17949, His1:CG33861, His1:CG33843, His1:CG33849, His2B:CG33870, His1:CG31617, His4:CG33879, His2B:CG33878, His2B:CG33888, His2B:CG33910, His2B:CG33880, His3:CG33845, His3:CG33839, His3:CG33848, His3:CG33851, His4:CG33899, His4:CG31611, His1:CG33819,

						His1:CG33816, His3:CG33836, His1:CG33846, His4:CG33887, His3:CG33863
chromatin organization	54 of 146 genes, 37.0%	516 of 16085 genes, 3.2%	1.55e-40	0. 00%	0.00	His3:CG33815, His2A:CG33850, SmydA-8, His1:CG33840, His2A:CG31618, His4:CG33885, His3:CG33818, His2B:CG33884, His2B:CG33900, His2A:CG33817, His3:CG33860, His2A:CG33841, His1:CG33837, His3:CG31613, His3:CG33842, His4:CG33909, His2A:CG33838, His1:CG33801, His3:CG33803, His4:CG33883, His2B:CG33886, His2A:CG33862, His4:CG33877, His4:CG33869, JMJD5, His1:CG33834, His2B:CG17949, sti, His1:CG33861, His1:CG33843, His1:CG33849, His2B:CG33870, His1:CG31617, His4:CG33879, His2B:CG33878, His2A:CG33835, His2B:CG33910, His2B:CG33888, His2B:CG33880, His3:CG33845, His3:CG33839, Nipped-A, Hers, His2A:CG33847, His3:CG33851, His3:CG33848, His4:CG33899, His4:CG31611, His1:CG33819, His1:CG33816, His3:CG33836, His3:CG33863, His1:CG33846, His4:CG33887
nucleosome assembly	32 of 146 genes, 21.9%	99 of 16085 genes, 0.6%	2.89e-39	0. 00%	0.00	His3:CG33815, His1:CG33840, His4:CG33885, His3:CG33818, His3:CG33860, His1:CG33837, His3:CG31613, His3:CG33842, His4:CG33909, His1:CG33801, His3:CG33803, His4:CG33883, His4:CG33877, His4:CG33869, His1:CG33834, His1:CG33861, His1:CG33849, His1:CG33843, His4:CG33879, His1:CG31617, His3:CG33845, His3:CG33839, His3:CG33848, His3:CG33851, His4:CG33899, His4:CG31611, His1:CG33819, His3:CG33836, His1:CG33816, His4:CG33887, His1:CG33846, His3:CG33863
chromatin assembly	32 of 146 genes, 21.9%	116 of 16085 genes, 0.7%	1.02e-36	0. 00%	0.00	His3:CG33815, His1:CG33840, His4:CG33885, His3:CG33818, His3:CG33860, His1:CG33837, His3:CG31613, His3:CG33842, His4:CG33909, His1:CG33801, His3:CG33803, His4:CG33883, His4:CG33877, His4:CG33869, His1:CG33834, His1:CG33861, His1:CG33849, His1:CG33843, His4:CG33879, His1:CG31617, His3:CG33845, His3:CG33839, His3:CG33848, His3:CG33851, His4:CG33899, His4:CG31611, His1:CG33819, His3:CG33836, His1:CG33816, His4:CG33887, His1:CG33846, His3:CG33863
nucleosome organization	33 of 146 genes, 22.6%	143 of 16085 genes, 0.9%	4.40e-35	0. 00%	0.00	His3:CG33815, His1:CG33840, His4:CG33885, His3:CG33818, His3:CG33860, His1:CG33837, His3:CG31613, His3:CG33842, His4:CG33909, His1:CG33801, His3:CG33803, His4:CG33883, His4:CG33877, His4:CG33869, His1:CG33834, His1:CG33861, His1:CG33849, His1:CG33843, His1:CG31617, His4:CG33879, His3:CG33845, His3:CG33839, Nipped-A, His3:CG33848, His3:CG33851, His4:CG33899, His4:CG31611, His1:CG33819, His1:CG33816, His3:CG33836, His4:CG33887, His1:CG33846, His3:CG33863
chromosome organization	55 of 146 genes, 37.7%	762 of 16085 genes, 4.7%	1.02e-32	0. 00%	0.00	His3:CG33815, chic, His2A:CG33850, SmydA-8, His1:CG33840, His2A:CG31618, His4:CG33885, His3:CG33818, His2B:CG33884, His2B:CG33900, His2A:CG33817, His3:CG33860, His2A:CG33841, His1:CG33837, His3:CG31613, His3:CG33842, His4:CG33909, His2A:CG33838, His1:CG33801, His3:CG33803, His4:CG33883, His2B:CG33886, His2A:CG33862, His4:CG33877, His4:CG33869, JMJD5, His1:CG33834, His2B:CG17949, sti, His1:CG33861, His1:CG33843, His1:CG33849, His2B:CG33870, His1:CG31617, His4:CG33879, His2B:CG33878, His2A:CG33835, His2B:CG33910, His2B:CG33888, His2B:CG33880, His3:CG33845, His3:CG33839, Nipped-A, Hers, His2A:CG33847, His3:CG33851, His3:CG33848, His4:CG33899, His4:CG31611, His1:CG33819, His1:CG33816, His3:CG33836, His3:CG33863, His1:CG33846, His4:CG33887
protein-DNA	32 of 146	155 of 16085	3.01e-32	0.	0.00	His3:CG33815, His1:CG33840, His4:CG33885,

complex assembly	genes, 21.9%	genes, 1.0%		00%		His3:CG33818, His3:CG33860, His1:CG33837, His3:CG31613, His3:CG33842, His4:CG33909, His1:CG33801, His3:CG33803, His4:CG33883, His4:CG33877, His4:CG33869, His1:CG33834, His1:CG33861, His1:CG33849, His1:CG33843, His4:CG33879, His1:CG31617, His3:CG33845, His3:CG33839, His3:CG33848, His3:CG33851, His4:CG33899, His4:CG31611, His1:CG33819, His3:CG33836, His1:CG33816, His4:CG33887, His1:CG33846, His3:CG33863
DNA packaging	32 of 146 genes, 21.9%	184 of 16085 genes, 1.1%	1.06e-29	0. 00%	0.00	His3:CG33815, His1:CG33840, His4:CG33885, His3:CG33818, His3:CG33860, His1:CG33837, His3:CG31613, His3:CG33842, His4:CG33909, His1:CG33801, His3:CG33803, His4:CG33883, His4:CG33877, His4:CG33869, His1:CG33834, His1:CG33861, His1:CG33849, His1:CG33843, His4:CG33879, His1:CG31617, His3:CG33845, His3:CG33839, His3:CG33848, His3:CG33851, His4:CG33899, His4:CG31611, His1:CG33819, His3:CG33836, His1:CG33816, His4:CG33887, His1:CG33846, His3:CG33863
protein-DNA complex subunit organization	33 of 146 genes, 22.6%	205 of 16085 genes, 1.3%	1.50e-29	0. 00%	0.00	His3:CG33815, His1:CG33840, His4:CG33885, His3:CG33818, His3:CG33860, His1:CG33837, His3:CG31613, His3:CG33842, His4:CG33909, His1:CG33801, His3:CG33803, His4:CG33883, His4:CG33877, His4:CG33869, His1:CG33834, His1:CG33861, His1:CG33849, His1:CG33843, His1:CG31617, His4:CG33879, His3:CG33845, His3:CG33839, Nipped-A, His3:CG33848, His3:CG33851, His4:CG33899, His4:CG31611, His1:CG33819, His1:CG33816, His3:CG33836, His4:CG33887, His1:CG33846, His3:CG33863
DNA conformation change	32 of 146 genes, 21.9%	209 of 16085 genes, 1.3%	7.56e-28	0. 00%	0.00	His3:CG33815, His1:CG33840, His4:CG33885, His3:CG33818, His3:CG33860, His1:CG33837, His3:CG31613, His3:CG33842, His4:CG33909, His1:CG33801, His3:CG33803, His4:CG33883, His4:CG33877, His4:CG33869, His1:CG33834, His1:CG33861, His1:CG33849, His1:CG33843, His4:CG33879, His1:CG31617, His3:CG33845, His3:CG33839, His3:CG33848, His3:CG33851, His4:CG33899, His4:CG31611, His1:CG33819, His3:CG33836, His1:CG33816, His4:CG33887, His1:CG33846, His3:CG33863
organelle organization	63 of 146 genes, 43.2%	2093 of 16085 genes, 13.0%	6.60e-17	0. 00%	0.00	His3:CG33815, chic, His2A:CG33850, SmydA-8, His1:CG33840, His2A:CG31618, His4:CG33885, His3:CG33818, His2B:CG33884, His2B:CG33900, His2A:CG33817, His3:CG33860, His2A:CG33841, His1:CG33837, cep290, His3:CG31613, His3:CG33842, Mical, His4:CG33909, parvin, His2A:CG33838, His1:CG33801, His3:CG33803, His4:CG33883, His2B:CG33886, His2A:CG33862, His4:CG33877, His4:CG33869, JMJD5, His1:CG33834, His2B:CG17949, sti, His1:CG33861, His1:CG33843, His1:CG33849, His2B:CG33870, His1:CG31617, His4:CG33879, His2B:CG33878, His2A:CG33835, bchs, cnc, His2B:CG33910, His2B:CG33888, His2B:CG33880, His3:CG33845, His3:CG33839, Nipped-A, COX6B, Hers, His2A:CG33847, His3:CG33851, His3:CG33848, His4:CG33899, His4:CG31611, His1:CG33819, Frl, His1:CG33816, His3:CG33836, His3:CG33863, fry, His1:CG33846, His4:CG33887
cellular protein-containing complex assembly	34 of 146 genes, 23.3%	559 of 16085 genes, 3.5%	1.98e-16	0. 00%	0.00	His3:CG33815, chic, His1:CG33840, His4:CG33885, His3:CG33818, His3:CG33860, His1:CG33837, His3:CG31613, His3:CG33842, His4:CG33909, His1:CG33801, His3:CG33803, His4:CG33883, His4:CG33877, His4:CG33869, His1:CG33834, His1:CG33861, His1:CG33849, His1:CG33843, His1:CG31617, His4:CG33879, His3:CG33845, His3:CG33839, COX6B, His3:CG33848, His3:CG33851, His4:CG33899, His4:CG31611, His1:CG33819, His1:CG33816,

						His3:CG33836, His4:CG33887, His1:CG33846, His3:CG33863
protein-containing complex assembly	35 of 146 genes, 24.0%	602 of 16085 genes, 3.7%	2.30e-16	0.00%	0.00	His3:CG33815, chic, His1:CG33840, His4:CG33885, His3:CG33818, His3:CG33860, His1:CG33837, His3:CG31613, His3:CG33842, His4:CG33909, His1:CG33801, His3:CG33803, His4:CG33883, His4:CG33877, His4:CG33869, His1:CG33834, His1:CG33861, His1:CG33849, His1:CG33843, His1:CG31617, His4:CG33879, Shaw, His3:CG33845, His3:CG33839, COX6B, His3:CG33848, His3:CG33851, His4:CG33899, His4:CG31611, His1:CG33819, His1:CG33816, His3:CG33836, His4:CG33887, His1:CG33846, His3:CG33863
protein-containing complex subunit organization	37 of 146 genes, 25.3%	733 of 16085 genes, 4.6%	2.07e-15	0.00%	0.00	His3:CG33815, chic, His1:CG33840, His4:CG33885, His3:CG33818, His3:CG33860, His1:CG33837, His3:CG31613, His3:CG33842, Mical, His4:CG33909, His1:CG33801, His3:CG33803, His4:CG33883, His4:CG33877, His4:CG33869, His1:CG33834, His1:CG33861, His1:CG33849, His1:CG33843, His1:CG31617, His4:CG33879, Shaw, His3:CG33845, His3:CG33839, COX6B, Nipped-A, His3:CG33848, His3:CG33851, His4:CG33899, His4:CG31611, His1:CG33819, His1:CG33816, His3:CG33836, His1:CG33846, His4:CG33887, His3:CG33863
cellular component assembly	41 of 146 genes, 28.1%	1314 of 16085 genes, 8.2%	3.83e-10	0.00%	0.00	His3:CG33815, chic, His1:CG33840, His4:CG33885, His3:CG33818, His3:CG33860, His1:CG33837, cep290, His3:CG31613, His3:CG33842, Mical, His4:CG33909, parvin, His1:CG33801, His3:CG33803, His4:CG33883, His4:CG33877, His4:CG33869, His1:CG33834, sti, His1:CG33861, His1:CG33843, His1:CG33849, His1:CG31617, His4:CG33879, Shaw, cnc, His3:CG33845, His3:CG33839, COX6B, His3:CG33848, His3:CG33851, His4:CG33899, His4:CG31611, His1:CG33819, mesh, His1:CG33816, His3:CG33836, His4:CG33887, His1:CG33846, His3:CG33863
cellular component organization	65 of 146 genes, 44.5%	3146 of 16085 genes, 19.6%	2.04e-09	0.00%	0.00	His3:CG33815, chic, His2A:CG33850, SmydA-8, His1:CG33840, His4:CG33885, His2B:CG33884, His2A:CG33817, His2A:CG33841, His1:CG33837, cep290, His4:CG33909, His2A:CG33838, His1:CG33801, His4:CG33883, His2B:CG33886, His4:CG33877, His4:CG33869, His1:CG33843, His1:CG33849, His4:CG33879, Shaw, His2B:CG33888, His3:CG33839, Nipped-A, COX6B, Hers, His2A:CG33847, His3:CG33848, His4:CG33899, Frl, His1:CG33816, His3:CG33836, His3:CG33863, His1:CG33846, His4:CG33887, His2A:CG31618, His3:CG33818, His2B:CG33900, His3:CG33860, His3:CG31613, His3:CG33842, parvin, Mical, His3:CG33803, His2A:CG33862, JMJD5, His1:CG33834, His2B:CG17949, sti, His1:CG33861, His2B:CG33870, His1:CG31617, His2B:CG33878, His2A:CG33835, bchs, His2B:CG33880, His2B:CG33910, cnc, His3:CG33845, His3:CG33851, His4:CG31611, His1:CG33819, mesh, fry
cellular component biogenesis	42 of 146 genes, 28.8%	1477 of 16085 genes, 9.2%	3.93e-09	0.00%	0.00	His3:CG33815, chic, His1:CG33840, His4:CG33885, His3:CG33818, His3:CG33860, His1:CG33837, cep290, His3:CG31613, His3:CG33842, Mical, His4:CG33909, parvin, His1:CG33801, His3:CG33803, His4:CG33883, His4:CG33877, His4:CG33869, His1:CG33834, sti, His1:CG33861, His1:CG33843, His1:CG33849, His1:CG31617, His4:CG33879, Shaw, cnc, His3:CG33845, His3:CG33839, COX6B, Rpp20, His3:CG33848, His3:CG33851, His4:CG33899, His4:CG31611, His1:CG33819, mesh, His1:CG33816, His3:CG33836, His1:CG33846, His4:CG33887, His3:CG33863

cellular component organization or biogenesis	66 of 146 genes, 45.2%	3291 of 16085 genes, 20.5%	4.97e-09	0. 00%	0.00	His3:CG33815, chic, His2A:CG33850, SmydA-8, His1:CG33840, His4:CG33885, His2B:CG33884, His2A:CG33817, His2A:CG33841, His1:CG33837, cep290, His4:CG33909, His2A:CG33838, His1:CG33801, His4:CG33883, His2B:CG33886, His4:CG33877, His4:CG33869, His1:CG33843, His1:CG33849, His4:CG33879, Shaw, His2B:CG33888, His3:CG33839, Nipped-A, COX6B, Hers, His2A:CG33847, His3:CG33848, His4:CG33899, Frl, His1:CG33816, His3:CG33836, His3:CG33863, His1:CG33846, His4:CG33887, His2A:CG31618, His3:CG33818, His2B:CG33900, His3:CG33860, His3:CG31613, His3:CG33842, parvin, Mical, His3:CG33803, His2A:CG33862, JMJD5, His1:CG33834, His2B:CG17949, sti, His1:CG33861, His2B:CG33870, His1:CG31617, His2B:CG33878, His2A:CG33835, bchs, His2B:CG33910, His2B:CG33880, cnc, His3:CG33845, Rpp20, His3:CG33851, His4:CG31611, His1:CG33819, mesh, fry
DNA-templated transcription, initiation	9 of 146 genes, 6.2%	114 of 16085 genes, 0.7%	0.00037	0. 00%	0.00	His4:CG33909, His4:CG33879, His4:CG33885, His4:CG33883, His4:CG33899, His4:CG33877, His4:CG31611, His4:CG33869, His4:CG33887

Table S3. GO associations with Biological Process (GENERIC GENE ONTOLOGY (GO) TERM FINDER) of 588 rDNA-contacting genes shown in Venn Diagram in Figure 1,*C*.

Gene Ontology term	Cluster frequency	Genome frequency	Corrected P-value	FDR	False Positives	Genes annotated to the term
chromatin assembly or disassembly	47 of 578 genes, 8.1%	147 of 16085 genes, 0.9%	7.91e-29	0.00%	0.00	His3:CG33815, His1:CG33840, His4:CG33885, His3:CG33818, His2B:CG33900, His2B:CG33884, His3:CG33866, His3:CG33860, His1:CG33837, His3:CG31613, His3:CG33842, His4:CG33909, His1:CG33801, His3:CG33803, His4:CG33883, His2B:CG33886, His4:CG33877, His4:CG33869, His1:CG33834, His2B:CG17949, His2B:CG33902, His1:CG33861, His1:CG33843, His1:CG33849, His2B:CG33870, His4:CG33901, His4:CG33879, His1:CG31617, His1:CG33864, His2B:CG33878, His2B:CG33868, His2B:CG33888, His2B:CG33880, His2B:CG33910, His3:CG33845, His3:CG33839, His1:CG33813, His3:CG33848, His3:CG33851, His4:CG33899, His4:CG31611, His1:CG33819, His1:CG33816, His3:CG33836, His4:CG33887, His3:CG33863, His1:CG33846
nucleosome assembly	36 of 578 genes, 6.2%	99 of 16085 genes, 0.6%	7.84e-24	0.00%	0.00	His3:CG33815, His1:CG33840, His4:CG33885, His3:CG33818, His3:CG33866, His1:CG33837, His3:CG33860, His3:CG31613, His3:CG33842, His4:CG33909, His1:CG33801, His3:CG33803, His4:CG33883, His4:CG33877, His4:CG33869, His1:CG33834, His1:CG33861, His1:CG33843, His1:CG33849, His4:CG33901, His4:CG33879, His1:CG31617, His1:CG33864, His3:CG33845, His3:CG33839, His3:CG33848, His3:CG33851, His1:CG33813, His4:CG33899, His4:CG31611, His1:CG33819, His3:CG33836, His1:CG33816, His1:CG33846, His3:CG33863, His4:CG33887
chromatin assembly	36 of 578 genes, 6.2%	116 of 16085 genes, 0.7%	4.47e-21	0.00%	0.00	His3:CG33815, His1:CG33840, His4:CG33885, His3:CG33818, His3:CG33866, His1:CG33837, His3:CG33860, His3:CG31613, His3:CG33842, His4:CG33909, His1:CG33801, His3:CG33803, His4:CG33883, His4:CG33877, His4:CG33869, His1:CG33834, His1:CG33861, His1:CG33843, His1:CG33849, His4:CG33901, His4:CG33879, His1:CG31617, His1:CG33864, His3:CG33845, His3:CG33839, His3:CG33848, His3:CG33851,

						His1:CG33813, His4:CG33899, His4:CG31611, His1:CG33819, His3:CG33836, His1:CG33816, His1:CG33846, His3:CG33863, His4:CG33887
nucleosome organization	37 of 578 genes, 6.4%	143 of 16085 genes, 0.9%	1.21e-18	0.00%	0.00	His3:CG33815, His1:CG33840, His4:CG33885, His3:CG33818, His3:CG33866, His1:CG33837, His3:CG33860, His3:CG31613, His3:CG33842, His4:CG33909, His1:CG33801, His3:CG33803, His4:CG33883, His4:CG33877, His4:CG33869, His1:CG33834, His1:CG33861, His1:CG33843, His1:CG33849, His4:CG33901, His4:CG33879, His1:CG31617, His1:CG33864, His3:CG33845, His3:CG33839, Nipped-A, His3:CG33848, His3:CG33851, His1:CG33813, His4:CG33899, His4:CG31611, His1:CG33819, His3:CG33836, His1:CG33816, His1:CG33846, His3:CG33863, His4:CG33887
chromatin organization	69 of 578 genes, 11.9%	516 of 16085 genes, 3.2%	2.22e-18	0.00%	0.00	His3:CG33815, His2A:CG33850, His1:CG33840, SmydA-8, His4:CG33885, His2B:CG33884, His2A:CG33817, His1:CG33837, His2A:CG33841, His4:CG33909, His2A:CG33838, His2A:CG33865, His1:CG33801, His4:CG33883, His2B:CG33886, His4:CG33877, His4:CG33869, His2B:CG33902, chif, His1:CG33849, His1:CG33843, His4:CG33879, Usp7, His2B:CG33868, His2B:CG33888, His3:CG33839, Nipped-A, Hers, His3:CG33848, His2A:CG33847, His4:CG33899, His1:CG33816, His3:CG33836, His1:CG33846, His3:CG33863, His4:CG33887, His2A:CG31618, Sfmbt, His3:CG33818, CG6220, His2B:CG33900, bon, His3:CG33866, His3:CG33860, His3:CG31613, His3:CG33842, His3:CG33803, His2A:CG33862, JMJD5, His1:CG33834, His2B:CG17949, sti, His1:CG33861, His2B:CG33870, His2A:CG33814, His4:CG33901, His1:CG31617, His1:CG33864, His2B:CG33878, His2A:CG33835, His2B:CG33880, His2B:CG33910, His3:CG33845, His3:CG33851, His1:CG33813, His4:CG31611, Samuel, His1:CG33819, tou
protein-DNA complex assembly	36 of 578 genes, 6.2%	155 of 16085 genes, 1.0%	2.19e-16	0.00%	0.00	His3:CG33815, His1:CG33840, His4:CG33885, His3:CG33818, His3:CG33866, His1:CG33837, His3:CG33860, His3:CG31613, His3:CG33842, His4:CG33909, His1:CG33801, His3:CG33803, His4:CG33883, His4:CG33877, His4:CG33869, His1:CG33834, His1:CG33861, His1:CG33843, His1:CG33849, His4:CG33901, His4:CG33879, His1:CG31617, His1:CG33864, His3:CG33845, His3:CG33839, His3:CG33848, His3:CG33851, His1:CG33813, His4:CG33899, His4:CG31611, His1:CG33819, His3:CG33836, His1:CG33816, His1:CG33846, His3:CG33863, His4:CG33887
protein-DNA complex subunit organization	38 of 578 genes, 6.6%	205 of 16085 genes, 1.3%	7.53e-14	0.00%	0.00	His3:CG33815, hay, His1:CG33840, His4:CG33885, His3:CG33818, His3:CG33866, His1:CG33837, His3:CG33860, His3:CG31613, His3:CG33842, His4:CG33909, His1:CG33801, His3:CG33803, His4:CG33883, His4:CG33877, His4:CG33869, His1:CG33834, His1:CG33861, His1:CG33843, His1:CG33849, His4:CG33901, His4:CG33879, His1:CG31617, His1:CG33864, His3:CG33845, His3:CG33839, Nipped-A, His3:CG33848, His3:CG33851, His1:CG33813, His4:CG33899, His4:CG31611, His1:CG33819, His1:CG33816, His3:CG33836, His1:CG33846, His3:CG33863, His4:CG33887
DNA packaging	36 of 578 genes, 6.2%	184 of 16085 genes, 1.1%	8.40e-14	0.00%	0.00	His3:CG33815, His1:CG33840, His4:CG33885, His3:CG33818, His3:CG33866, His1:CG33837, His3:CG33860, His3:CG31613, His3:CG33842, His4:CG33909, His1:CG33801, His3:CG33803, His4:CG33883, His4:CG33877, His4:CG33869, His1:CG33834, His1:CG33861, His1:CG33843, His1:CG33849, His4:CG33901, His4:CG33879, His1:CG31617, His1:CG33864, His3:CG33845, His3:CG33839, His3:CG33848, His3:CG33851, His1:CG33813, His4:CG33899, His4:CG31611,

						His1:CG33819, His3:CG33836, His1:CG33816, His1:CG33846, His3:CG33863, His4:CG33887
chromosome organization	76 of 578 genes, 13.1%	762 of 16085 genes, 4.7%	5.65e-13	0.00%	0.00	His3:CG33815, His2A:CG33850, His1:CG33840, SmydA-8, chic, His4:CG33885, tea, His2B:CG33884, His2A:CG33817, His2A:CG33841, His1:CG33837, His4:CG33909, His2A:CG33838, His2A:CG33865, His1:CG33801, His4:CG33883, cuff, His2B:CG33886, His4:CG33877, His4:CG33869, His2B:CG33902, chif, His1:CG33849, His1:CG33843, His4:CG33879, Usp7, His2B:CG33868, His2B:CG33888, c(3)G, His3:CG33839, Nipped-A, Hers, His3:CG33848, His2A:CG33847, His4:CG33899, CG34001, His1:CG33816, His3:CG33836, His1:CG33846, His3:CG33863, His4:CG33887, His2A:CG31618, Sfmbt, CG15237, His3:CG33818, His2B:CG33900, CG6220, bon, His3:CG33866, His3:CG33860, His3:CG31613, His3:CG33842, His3:CG33803, His2A:CG33862, JMJD5, His1:CG33834, His2B:CG17949, vih, sti, His1:CG33861, His2A:CG33814, His2B:CG33870, His4:CG33901, His1:CG31617, His1:CG33864, His2B:CG33878, His2A:CG33835, His2B:CG33880, His2B:CG33910, His3:CG33845, His1:CG33813, His3:CG33851, His4:CG31611, His1:CG33819, Samuel, tou
DNA conformation change	36 of 578 genes, 6.2%	209 of 16085 genes, 1.3%	5.68e-12	0.00%	0.00	His3:CG33815, His1:CG33840, His4:CG33885, His3:CG33818, His3:CG33866, His1:CG33837, His3:CG33860, His3:CG31613, His3:CG33842, His4:CG33909, His1:CG33801, His3:CG33803, His4:CG33883, His4:CG33877, His4:CG33869, His1:CG33834, His1:CG33861, His1:CG33843, His1:CG33849, His4:CG33901, His4:CG33879, His1:CG31617, His1:CG33864, His3:CG33845, His3:CG33839, His3:CG33848, His3:CG33851, His1:CG33813, His4:CG33899, His4:CG31611, His1:CG33819, His3:CG33836, His1:CG33816, His1:CG33846, His3:CG33863, His4:CG33887
cellular component assembly	84 of 578 genes, 14.5%	1314 of 16085 genes, 8.2%	0.00019	0.00%	0.00	His3:CG33815, bnl, His1:CG33840, chic, His4:CG33885, His1:CG33837, Dg, scra, cep290, Tom20, His4:CG33909, His1:CG33801, wun, His4:CG33883, cuff, His4:CG33877, His4:CG33869, Octbeta2R, chif, Liprin-alpha, His1:CG33849, His1:CG33843, His4:CG33879, TTLL3B, svr, Shaw, atl, CG13185, c(3)G, His3:CG33839, Atg8a, COX6B, Prp8, caps, His3:CG33848, Sema1a, His4:CG33899, His1:CG33816, His3:CG33836, His1:CG33846, His3:CG33863, His4:CG33887, Nedd4, CG17768, Pxn, His3:CG33818, CG6220, His3:CG33866, His3:CG33860, ens, His3:CG31613, His3:CG33842, parvin, Galphai, Mical, His3:CG33803, aPKC, His1:CG33834, Sdc, sti, LanB1, shot, x16, His1:CG33861, Zasp52, Hrb27C, His4:CG33901, His1:CG31617, Skeletor, His1:CG33864, Nlg4, cnc, spin, His3:CG33845, cdc14, Atpalpha, His1:CG33813, Got2, His3:CG33851, His4:CG31611, BBS1, His1:CG33819, mesh, Shark
protein-containing complex subunit organization	55 of 578 genes, 9.5%	733 of 16085 genes, 4.6%	0.00027	0.00%	0.00	CG17768, His3:CG33815, hay, His1:CG33840, chic, His4:CG33885, Pxn, His3:CG33818, CG6220, His3:CG33866, His3:CG33860, His1:CG33837, ens, His3:CG31613, Tom20, His3:CG33842, His4:CG33909, Mical, His1:CG33801, His3:CG33803, His4:CG33883, cuff, His4:CG33877, His4:CG33869, His1:CG33834, x16, shot, His1:CG33861, His1:CG33843, His1:CG33849, His4:CG33901, His1:CG31617, His4:CG33879, His1:CG33864, svr, Shaw, atl, CG13185, His3:CG33845, Atg8a, His3:CG33839, Prp8, COX6B, Nipped-A, His1:CG33813, His3:CG33851, His3:CG33848, His4:CG33899, His4:CG31611, His1:CG33819, His1:CG33816, His3:CG33836, His4:CG33887, His3:CG33863, His1:CG33846
protein-containing	48 of 578 genes, 8.3%	602 of 16085 genes, 3.7%	0.00029	0.00%	0.00	CG17768, His3:CG33815, His1:CG33840, chic, His4:CG33885, His3:CG33818, CG6220,

complex assembly						His3:CG33866, His3:CG33860, His1:CG33837, ens, His3:CG31613, Tom20, His3:CG33842, His4:CG33909, His1:CG33801, His3:CG33803, His4:CG33883, His4:CG33877, His4:CG33869, His1:CG33834, x16, His1:CG33861, His1:CG33843, His1:CG33849, His4:CG33901, His1:CG31617, His4:CG33879, His1:CG33864, svr, Shaw, atl, CG13185, His3:CG33845, His3:CG33839, Prp8, COX6B, His1:CG33813, His3:CG33848, His3:CG33851, His4:CG33899, His4:CG31611, His1:CG33819, His1:CG33816, His3:CG33836, His4:CG33887, His3:CG33863, His1:CG33846
cellular component organization	163 of 578 genes, 28.2%	3146 of 16085 genes, 19.6%	0.00031	0.00%	0.00	cwo, bnl, chico, side, SmydA-8, tea, His2B:CG33884, His2A:CG33841, Dg, scra, CG18635, CG45105, wun, RunxA, cuff, His4:CG33877, His4:CG33869, Nak, ReepB, chif, Glt, Liprin-alpha, His1:CG33849, His1:CG33843, His4:CG33879, svr, atl, CG13185, His3:CG33839, Nipped-A, COX6B, caps, Sema1a, Traf4, Frl, His1:CG33816, His3:CG33836, His1:CG33846, His3:CG33863, His4:CG33887, Nedd4, Sfmbt, CG15237, Pxn, CG6220, pan, His3:CG31613, PlexA, Galphai, parvin, His3:CG33803, aPKC, His2A:CG33862, JMJD5, Tango4, sti, LanB1, His1:CG33861, x16, Zasp52, His2A:CG33814, His2B:CG33870, spen, His4:CG33901, His1:CG31617, His2A:CG33835, bchs, NiPp1, Tim17a2, cnc, spin, His3:CG33845, Pvf2, Atpalpha, BBS1, pico, His1:CG33819, fry, DNaseII, His3:CG33815, chic, His1:CG33840, His2A:CG33850, His4:CG33885, CG5984, His2A:CG33817, Prosbeta7, His1:CG33837, cep290, Tom20, His4:CG33909, His2A:CG33838, His2A:CG33865, His1:CG33801, His4:CG33883, His2B:CG33886, Octbeta2R, haf, His2B:CG33902, CG10413, Itp-r83A, tai, TTLL3B, Svil, Shaw, Usp7, His2B:CG33868, His2B:CG33888, c(3)G, Atg8a, Prp8, dpr17, Drep4, Hers, His2A:CG33847, His3:CG33848, His4:CG33899, CG34001, CG17768, hay, Frq1, His2A:CG31618, His3:CG33818, His2B:CG33900, bon, fru, Toll-7, His3:CG33866, His3:CG33860, ens, His3:CG33842, Sry-alpha, Mical, Eph, His1:CG33834, His2B:CG17949, Syx18, vih, Sdc, shot, mgl, Hrb27C, betaTub97EF, CadN, Skeletor, His1:CG33864, His2B:CG33878, Pkn, Nlg4, His2B:CG33880, His2B:CG33910, cdc14, His1:CG33813, Got2, Hph, His3:CG33851, His4:CG31611, Rab5, Samuel, mesh, Shark, tou, sle
cellular protein-containing complex assembly	45 of 578 genes, 7.8%	559 of 16085 genes, 3.5%	0.00055	0.00%	0.00	CG17768, His3:CG33815, His1:CG33840, chic, His4:CG33885, His3:CG33818, CG6220, His3:CG33866, His3:CG33860, His1:CG33837, ens, His3:CG31613, Tom20, His3:CG33842, His4:CG33909, His1:CG33801, His3:CG33803, His4:CG33883, His4:CG33877, His4:CG33869, His1:CG33834, His1:CG33861, His1:CG33843, His1:CG33849, His4:CG33901, His4:CG33879, His1:CG31617, His1:CG33864, svr, CG13185, His3:CG33845, His3:CG33839, Prp8, COX6B, His1:CG33813, His3:CG33848, His3:CG33851, His4:CG33899, His4:CG31611, His1:CG33819, His1:CG33816, His3:CG33836, His4:CG33887, His3:CG33863, His1:CG33846
organelle organization	117 of 578 genes, 20.2%	2093 of 16085 genes, 13.0%	0.00073	0.00%	0.00	His3:CG33815, His2A:CG33850, His1:CG33840, SmydA-8, chic, His4:CG33885, CG5984, tea, His2B:CG33884, His2A:CG33817, Prosbeta7, His2A:CG33841, His1:CG33837, Dg, scra, Tom20, cep290, His4:CG33909, His2A:CG33838, His2A:CG33865, CG45105, His1:CG33801, His4:CG33883, cuff, His2B:CG33886, His4:CG33877, His4:CG33869, ReepB, His2B:CG33902, chif, His1:CG33849, His1:CG33843, Itp-r83A, His4:CG33879, TTLL3B, Svil, svr, atl, Usp7, His2B:CG33868, His2B:CG33888, CG13185, c(3)G, His3:CG33839, Atg8a, COX6B, Nipped-A, Hers, His3:CG33848, His2A:CG33847, His4:CG33899,

						CG34001, Frl, His1:CG33816, His3:CG33836, His1:CG33846, His3:CG33863, His4:CG33887, Nedd4, CG17768, His2A:CG31618, Sfmbt, CG15237, His3:CG33818, His2B:CG33900, CG6220, bon, His3:CG33866, His3:CG33860, ens, His3:CG31613, His3:CG33842, Galphai, parvin, Sry-alpha, Mical, His3:CG33803, aPKC, His2A:CG33862, JMJD5, His1:CG33834, His2B:CG17949, vih, Tango4, sti, His1:CG33861, shot, Zasp52, His2A:CG33814, His2B:CG33870, His4:CG33901, His1:CG31617, betaTub97EF, His2B:CG33878, His1:CG33864, Skeletor, Pkn, His2A:CG33835, bchs, Tim17a2, His2B:CG33910, His2B:CG33880, cnc, spin, His3:CG33845, cdc14, His1:CG33813, His3:CG33851, His4:CG31611, pico, BBS1, His1:CG33819, Rab5, Samuel, tou, sle, fry
cellular component organization or biogenesis	166 of 578 genes, 28.7%	3291 of 16085 genes, 20.5%	0.00137	0.00%	0.00	cwo, bnl, chico, side, SmydA-8, tea, His2B:CG33884, His2A:CG33841, Dg, scra, CG18635, CG45105, wun, RunxA, cuff, His4:CG33877, His4:CG33869, Nak, ReepB, chif, Glt, Liprin-alpha, His1:CG33849, His1:CG33843, His4:CG33879, svr, atl, CG13185, His3:CG33839, Nipped-A, COX6B, caps, Sema1a, Traf4, Frl, His1:CG33816, His3:CG33836, His1:CG33846, His3:CG33863, His4:CG33887, Nedd4, nop5, Sfmbt, CG15237, Pxn, CG6220, pan, His3:CG31613, PlexA, Galphai, parvin, His3:CG33803, aPKC, His2A:CG33862, JMJD5, Tango4, sti, LanB1, His1:CG33861, x16, Zasp52, His2A:CG33814, His2B:CG33870, spen, His4:CG33901, His1:CG31617, His2A:CG33835, bchs, CG10286, NiPp1, Tim17a2, cnc, spin, His3:CG33845, Pvf2, Atpalpha, Rpp20, BBS1, pico, His1:CG33819, fry, DNaseII, His3:CG33815, chic, His1:CG33840, His2A:CG33850, His4:CG33885, CG5984, His2A:CG33817, Prosbeta7, His1:CG33837, cep290, Tom20, His4:CG33909, His2A:CG33838, His2A:CG33865, His1:CG33801, His4:CG33883, His2B:CG33886, Octbeta2R, haf, His2B:CG33902, CG10413, Itp-r83A, tai, TTLL3B, Svil, Shaw, Usp7, His2B:CG33868, His2B:CG33888, c(3)G, Atg8a, Prp8, dpr17, Drep4, Hers, His2A:CG33847, His3:CG33848, His4:CG33899, CG34001, CG17768, hay, Frq1, His2A:CG31618, His3:CG33818, His2B:CG33900, bon, fru, Toll-7, His3:CG33866, His3:CG33860, ens, His3:CG33842, Sry-alpha, Mical, Eph, His1:CG33834, His2B:CG17949, Syx18, vih, Sdc, shot, mgl, Hrb27C, betaTub97EF, CadN, Skeletor, His1:CG33864, His2B:CG33878, Pkn, Nlg4, His2B:CG33910, His2B:CG33880, cdc14, His1:CG33813, Got2, Hph, His3:CG33851, His4:CG31611, Rab5, Samuel, mesh, Shark, tou, sle
cellular component biogenesis	87 of 578 genes, 15.1%	1477 of 16085 genes, 9.2%	0.00372	0.00%	0.00	His3:CG33815, bnl, His1:CG33840, chic, His4:CG33885, His1:CG33837, Dg, scra, cep290, Tom20, His4:CG33909, His1:CG33801, wun, His4:CG33883, cuff, His4:CG33877, His4:CG33869, Octbeta2R, chif, Liprin-alpha, His1:CG33849, His1:CG33843, His4:CG33879, TTLL3B, svr, Shaw, atl, CG13185, c(3)G, His3:CG33839, Atg8a, Prp8, COX6B, caps, His3:CG33848, Sema1a, His4:CG33899, His1:CG33816, His3:CG33836, His1:CG33846, His3:CG33863, His4:CG33887, Nedd4, CG17768, nop5, Pxn, His3:CG33818, CG6220, His3:CG33866, His3:CG33860, ens, His3:CG31613, His3:CG33842, parvin, Galphai, Mical, His3:CG33803, aPKC, His1:CG33834, Sdc, sti, LanB1, shot, x16, His1:CG33861, Zasp52, Hrb27C, His4:CG33901, His1:CG31617, Skeletor, His1:CG33864, Nlg4, CG10286, cnc, spin, His3:CG33845, cdc14, Atpalpha, His1:CG33813, Got2, Rpp20, His3:CG33851, His4:CG31611, BBS1, His1:CG33819, mesh, Shark
unannotated	170 of 578 genes, 29.4%	3494 of 16085 genes, 21.7%	0.00945	0.00%	0.00	asRNA:CR44083, CG3764, lncRNA:CR32385, lncRNA:CR44730, red, CG18327, asRNA:CR44368, fipi, CG15211, asRNA:CR43465, CG1888, snmRNA:430c, CG8366, pre-rRNA:CR45845,

						CG15286, 2SrRNA:CR45840, CG5787, snmRNA:430s, 18SrRNA:CR45841, CG17716, CG42322, CR45495, mir-3643, CG15186, 18SrRNA:CR45838, mir-2500, CG15219, CG7943, lncRNA:CR44787, CG32369, CG16986, CG8628, CG3176, lncRNA:CR45039, asRNA:CR46256, lncRNA:CR44833, snmRNA:430k, mir-10404-1, CG3823, CG7755, Sws1, CG1161, CG2157, CG12592, lncRNA:CR45580, CG34166, CG30430, lncRNA:CR43334, lncRNA:CR44917, CG14005, lncRNA:CR45306, CR43383, CG10621, CR15821, 2SrRNA:CR45843, CG33932, CG12655, CG42340, lncRNA:CR44999, CG42231, CG32152, CG9016, CG13698, CG7289, CR18166, CG13203, lncRNA:CR44602, 2SrRNA-Psi:CR45850, CG13992, CG13954, CG15829, mir-10404-2, CG16732, CG32459, lncRNA:CR43423, snmRNA:430n, CG44085, 28SrRNA-Psi:CR45851, snmRNA:430j, 18SrRNA:CR41548, CG15894, 28SrRNA:CR45844, lncRNA:CR44874, CG17068, kek3, CG1513, lncRNA:CR45668, lncRNA:CR44741, CG15370, Shroom, snmRNA:430l, CG46310, CG7031, lncRNA:CR45038, His-Psi:CR33802, lncRNA:CR44938, CR45498, lncRNA:CR44811, snmRNA:430a, CR18275, asRNA:CR44416, asRNA:CR45501, snmRNA:430t, pre-rRNA:CR45847, CG9068, snmRNA:430d, CR45458, lncRNA:CR45037, CG2082, asRNA:CR43908, asRNA:CR45137, CG17121, CR45033, 28SrRNA:CR45837, snoRNA:nop5-x16-a, CG13983, lncRNA:CR45363, snRNA:U3, CG31636, CG12121, lncRNA:CR45460, 28SrRNA-Psi:CR45862, CG6051, CG31211, CG15258, Glut4EF, lncRNA:CR44544, CG13177, CG10170, snmRNA:430o, CG18273, CG3597, CG32436, CG10834, CG8323, lncRNA:CR45735, asRNA:CR44995, CR43382, pre-rRNA:CR45856, lncRNA:CR45459, lncRNA:CR44184, lncRNA:CR45222, 28SrRNA-Psi:CR45859, His-Psi:CR33867, CG6511, 2SrRNA:CR45836, CG18324, smal, asRNA:CR44047, 18SrRNA-Psi:CR41602, mir-2282, pre-rRNA:CR45846, CG2186, 2SrRNA-Psi:CR45858, 18SrRNA-Psi:CR45861, CG8629, CR43377, CG5151, CG16762, prim, lncRNA:CR45660, CG31751, CG12535, CG31703, Sry-beta, 28SrRNA-Psi:CR40596, CG10011, CG31223, CG10168, CG14370

Table S4. GO associations with Biological Process (GENERIC GENE ONTOLOGY (GO) TERM FINDER) of 871 rDNA-contacting genes shown in the Venn diagram in Fig. 1*C*.

Gene Ontology term	Cluster frequency	Genome frequency	Corrected P-value	FDR	False Positives	Genes annotated to the term
chromatin assembly or disassembly	39 of 862 genes, 4.5%	147 of 16085 genes, 0.9%	5.95e-14	0.00%	0.00	His2B:CG33874, Df31, His3:CG33821, mam, His4:CG33907, His1:CG33855, His2B:CG33894, His4:CG33871, His2B:CG33898, His1:CG33828, His3:CG33824, His4:CG33891, His3:CG33827, His2B:CG33896, His2B:CG33890, His2B:CG33872, His4:CG33893, His3:CG33833, His4:CG33875, His1:CG33822, His4:CG33897, His1:CG33858, His2B:CG33892, His2B:CG33876, His3:CG33857, His1:CG33831, His4:CG33889, His4:CG33881, His3:CG33806, mod(mdg4), His1:CG33852, His3:CG33854, His4:CG33895, His4:CG33873, His2B:CG33908, His1:CG33825, His2B:CG33882,

						His1:CG33804, His3:CG33830
regulation of protein serine/threonine phosphatase activity	12 of 862 genes, 1.4%	13 of 16085 genes, 0.1%	1.29e-11	0.00%	0.00	Ste:CG33243, Ste:CG33238, Ste:CG33242, Ste:CG33239, Ste:CG33241, Ste:CG33244, Ste:CG33246, Ste:CG33240, Ste:CG33236, Ste:CG33245, Ste:CG33247, Ste:CG33237
nucleosome assembly	28 of 862 genes, 3.2%	99 of 16085 genes, 0.6%	3.20e-10	0.00%	0.00	His4:CG33897, His1:CG33858, Df31, His1:CG33831, His3:CG33857, His4:CG33881, His4:CG33889, His3:CG33821, His3:CG33806, mam, His4:CG33907, His1:CG33855, His1:CG33852, His4:CG33871, His1:CG33828, His3:CG33824, His4:CG33891, His3:CG33827, His3:CG33854, His4:CG33895, His4:CG33873, His1:CG33825, His4:CG33893, His3:CG33833, His4:CG33875, His1:CG33822, His1:CG33804, His3:CG33830
chromatin assembly	28 of 862 genes, 3.2%	116 of 16085 genes, 0.7%	2.23e-08	0.00%	0.00	His4:CG33897, His1:CG33858, Df31, His1:CG33831, His3:CG33857, His4:CG33881, His4:CG33889, His3:CG33821, His3:CG33806, mam, His4:CG33907, His1:CG33855, His1:CG33852, His4:CG33871, His1:CG33828, His3:CG33824, His4:CG33891, His3:CG33827, His3:CG33854, His4:CG33895, His4:CG33873, His1:CG33825, His4:CG33893, His3:CG33833, His4:CG33875, His1:CG33822, His1:CG33804, His3:CG33830
regulation of phosphoprotein phosphatase activity	13 of 862 genes, 1.5%	24 of 16085 genes, 0.1%	7.97e-08	0.00%	0.00	Ste:CG33243, Ste:CG33238, Ste:CG33242, Ste:CG33239, Ste:CG33241, Ste:CG33244, I-2, Ste:CG33246, Ste:CG33240, Ste:CG33236, Ste:CG33245, Ste:CG33247, Ste:CG33237
protein-DNA complex assembly	31 of 862 genes, 3.6%	155 of 16085 genes, 1.0%	3.14e-07	0.00%	0.00	His4:CG33897, His1:CG33858, Df31, His1:CG33831, His3:CG33857, Spc105R, His4:CG33881, His4:CG33889, His3:CG33821, His3:CG33806, mam, His4:CG33907, His1:CG33855, His1:CG33852, His4:CG33871, Orc3, His1:CG33828, His3:CG33824, His4:CG33891, His3:CG33827, His4:CG33895, His3:CG33854, His4:CG33873, His1:CG33825, His4:CG33893, His3:CG33833, Taf11, His4:CG33875, His1:CG33822, His1:CG33804, His3:CG33830
regulation of phosphatase activity	13 of 862 genes, 1.5%	30 of 16085 genes, 0.2%	2.83e-06	0.00%	0.00	Ste:CG33243, Ste:CG33238, Ste:CG33242, Ste:CG33239, Ste:CG33241, Ste:CG33244, I-2, Ste:CG33246, Ste:CG33240, Ste:CG33236, Ste:CG33245, Ste:CG33247, Ste:CG33237
regulation of protein dephosphorylation	13 of 862 genes, 1.5%	30 of 16085 genes, 0.2%	2.83e-06	0.00%	0.00	Ste:CG33243, Ste:CG33238, Ste:CG33242, Ste:CG33239, Ste:CG33241, Ste:CG33244, I-2, Ste:CG33246, Ste:CG33240, Ste:CG33236, Ste:CG33245, Ste:CG33247, Ste:CG33237
nucleosome organization	28 of 862 genes, 3.2%	143 of 16085 genes, 0.9%	4.01e-06	0.00%	0.00	His4:CG33897, His1:CG33858, Df31, His1:CG33831, His3:CG33857, His4:CG33881, His4:CG33889, His3:CG33821, His3:CG33806, mam, His4:CG33907, His1:CG33855, His1:CG33852, His4:CG33871, His1:CG33828, His3:CG33824, His4:CG33891, His3:CG33827, His3:CG33854, His4:CG33895, His4:CG33873, His1:CG33825, His4:CG33893, His3:CG33833,

						His4:CG33875, His1:CG33822, His1:CG33804, His3:CG33830
regulation of dephosphorylation	13 of 862 genes, 1.5%	36 of 16085 genes, 0.2%	4.04e-05	0.00%	0.00	Ste:CG33243, Ste:CG33238, Ste:CG33242, Ste:CG33239, Ste:CG33241, Ste:CG33244, I-2, Ste:CG33246, Ste:CG33240, Ste:CG33236, Ste:CG33245, Ste:CG33247, Ste:CG33237
chromatin organization	58 of 862 genes, 6.7%	516 of 16085 genes, 3.2%	0.00013	0.00%	0.00	kis, His2B:CG33874, ash2, His2A:CG33859, Df31, His2A:CG33820, His3:CG33821, mam, His4:CG33907, His2A:CG33823, His1:CG33855, His2B:CG33894, His4:CG33871, Naa30A, His2B:CG33898, His1:CG33828, His3:CG33824, His4:CG33891, CG40228, His3:CG33827, His2B:CG33896, His2B:CG33890, His2B:CG33872, His4:CG33893, His3:CG33833, His1:CG33822, His4:CG33875, His2A:CG33844, His4:CG33897, His1:CG33858, His2B:CG33892, Nlp, His2B:CG33876, His3:CG33857, His1:CG33831, His4:CG33889, His4:CG33881, His3:CG33806, mod(mdg4), Nph, His1:CG33852, Hnf4, His4:CG33895, His3:CG33854, His2A:CG33829, His2A:CG33826, Sgf11, His4:CG33873, Nup153, His2A:CG33853, His2B:CG33908, tara, His2A:CG33856, His1:CG33825, His2A:CG33832, His2B:CG33882, His1:CG33804, His3:CG33830
protein-DNA complex subunit organization	31 of 862 genes, 3.6%	205 of 16085 genes, 1.3%	0.00032	0.00%	0.00	His4:CG33897, His1:CG33858, Df31, His1:CG33831, His3:CG33857, Spc105R, His4:CG33881, His4:CG33889, His3:CG33821, His3:CG33806, mam, His4:CG33907, His1:CG33855, His1:CG33852, His4:CG33871, Orc3, His1:CG33828, His3:CG33824, His4:CG33891, His3:CG33827, His4:CG33895, His3:CG33854, His4:CG33873, His1:CG33825, His4:CG33893, His3:CG33833, Taf11, His4:CG33875, His1:CG33822, His1:CG33804, His3:CG33830
DNA packaging	29 of 862 genes, 3.4%	184 of 16085 genes, 1.1%	0.00032	0.00%	0.00	His4:CG33897, His1:CG33858, Df31, His1:CG33831, His3:CG33857, His4:CG33881, His4:CG33889, His3:CG33821, His3:CG33806, mam, His4:CG33907, mod(mdg4), His1:CG33855, His1:CG33852, His4:CG33871, His1:CG33828, His3:CG33824, His4:CG33891, His3:CG33827, His3:CG33854, His4:CG33895, His4:CG33873, His1:CG33825, His4:CG33893, His3:CG33833, His4:CG33875, His1:CG33822, His1:CG33804, His3:CG33830
DNA conformation change	31 of 862 genes, 3.6%	209 of 16085 genes, 1.3%	0.00049	0.00%	0.00	His4:CG33897, His1:CG33858, Df31, His1:CG33831, His3:CG33857, His4:CG33881, His4:CG33889, His3:CG33821, His3:CG33806, mam, His4:CG33907, mod(mdg4), His1:CG33855, His1:CG33852, His4:CG33871, His1:CG33828, His3:CG33824, His4:CG33891, His3:CG33827, His4:CG33895, His3:CG33854, Top3alpha, His4:CG33873, rad50, His1:CG33825, His4:CG33893, His3:CG33833, His4:CG33875, His1:CG33822, His1:CG33804, His3:CG33830
unannotated	249 of 862 genes, 28.9%	3573 of 16085 genes, 22.2%	0.00304	0.00%	0.00	CG31663, lncRNA:CR44905, mir-967, CG5565, snmRNA:430h, lncRNA:CR45810, snmRNA:430e, Dlip1, Su(Ste):CR42428,

						lncRNA:CR44710, CG33223, lncRNA:CR45632, lncRNA:CR44143, lncRNA:CR44413, zf30C, lncRNA:CR44875, CG14044, CG1273, CG10182, CG17180, snoRNA:Psi18S-1347a, CG34228, 28SrRNA-Psi:CR41609, CG16723, lncRNA:CR45267, lncRNA:CR46212, CG34315, lncRNA:CR45971, CG46308, CG10803, lncRNA:CR43705, lncRNA:CR45256, lncRNA:CR44443, CG34351, CG13405, CG13280, lncRNA:CR44075, snmRNA:430m, CG13560, Kaz1-ORFA, lncRNA:CR44779, CG7058, asRNA:CR44592, lncRNA:CR45373, CG14339, 2SrRNA-Psi:CR40677, lectin-24A, lncRNA:CR44349, CG5561, Ste12DOR, snmRNA:430f, snmRNA:430q, lncRNA:CR43904, side-VIII, CG40470, CR41320, lncRNA:CR45755, CG2611, CR40450, CG30268, CG34278, lncRNA:CR45374, CG43200, snoRNA:Psi18S-1347b, lncRNA:CR44357, LRP1, CG17490, asRNA:CR46109, lncRNA:CR44528, lncRNA:CR43960, CG6621, lncRNA:CR45421, CG42733, CG17279, lncRNA:CR44997, lncRNA:CR45750, CG9034, asRNA:CR45396, CR32821, CG5664, lncRNA:CR43650, CG34254, lncRNA:CR46103, CG3402, CG13898, Hlc, 28SrRNA-Psi:CR45853, sisRNA:CR46360, lncRNA:CR45168, lncRNA:CR43838, CG12541, lncRNA:CR45262, lncRNA:CR45321, CG5050, CR41423, CG11210, lncRNA:CR44802, CG43201, lncRNA:CR44993, CG14427, lncRNA:CR45809, lncRNA:CR44931, lncRNA:CR44909, CG4467, snoRNA:Me28S-C2645b, CG33946, CG44838, mir-4953, CG3842, lncRNA:CR45740, lncRNA:CR46249, snoRNA:Me28S-C2645c, snoRNA:Or-CD12, CG1468, CG32695, lncRNA:CR44568, CG12898, CG42705, mir-4965, lncRNA:CR46268, CG3698, CG43171, 28SrRNA-Psi:CR40741, lncRNA:CR44860, snoRNA:Psi18S-1347c, CR45470, CG13135, asRNA:CR44992, 28SrRNA-Psi:CR45848, lncRNA:CR43498, CG13110, CG32428, CG8108, CG32202, lncRNA:CR44685, CG46307, CR43384, CG7548, Ady43A, lncRNA:CR43492, CR45496, firl, CG12902, lncRNA:CR44991, lncRNA:CR44530, 28SrRNA-Psi:CR45855, lncRNA:CR44865, CG7509, Uhg3, CG43350, CG6959, snoRNA:Me28S-C3351, lncRNA:CR44470, CR43378, lncRNA:CR46042, lncRNA:CR44594, Aef1, CR43080, asRNA:CR45161, CG14285, mir-4978, Ir48a, lncRNA:CR46106, Su(Ste):CR42427, CG43178, CG14830, asRNA:CR43470, lncRNA:CR45754, zormin, lncRNA:CR46215, CG46305, lncRNA:CR42735, CG43172, asRNA:CR45600, CG9173, lncRNA:CR46075, CG13397, lncRNA:CR44621, CG7295, asRNA:CR45397, lncRNA:CR46024, Paf-AHalpha, CG2608, CR40801, CG10178, CG34200, CG16786, CG34402, snmRNA:430p, His-Psi:CR33805, side-III, mir-2489, lncRNA:CR45878, CG13144, CG9582, lncRNA:CR43654, CG5367, lncRNA:CR44352, CG15147, lncRNA:CR43706, Su(Ste):CR42414,

						lncRNA:CR44844, CR41508, CG3984, lncRNA:CR43485, Kaz1-ORFB, CG4133, CG12516, CG7692, snmRNA:430r, CG7029, lncRNA:CR44241, lncRNA:CR45519, CG42524, lncRNA:CR32657, asRNA:CR44509, Gnpnat, lncRNA:CR45808, snoRNA:Me28S-C2645a, CG6912, CG13297, lncRNA:CR44593, CG13285, CG2233, lncRNA:CR45883, lncRNA:CR33938, lncRNA:CR46197, CG15570, snmRNA:430g, mir-9371, CG11300, lncRNA:CR46080, snmRNA:430i, lncRNA:CR44022, Cp38, Ptr, CG42704, CG31924, CG3909, lncRNA:CR44494, phr6-4, CG43759, lncRNA:CR42696, snoRNA:MeU6-A47, CG11123, CG5810, Leash, asRNA:CR45189, CG30460

Table S5. Changes in contact frequencies of DNA-contacting genes with rDNA clusters after heat shock treatment. Excel file attached separately.

Table S6. GO associations with Biological Process (GENERIC GENE ONTOLOGY (GO) TERM FINDER) of 418 rDNA-contacting genes shown in the Venn diagram in Fig. 5B and in the diagram in Fig. 5C.

Gene Ontology term	Cluster frequency	Genome frequency	Corrected P-value	FDR	False Positives	Genes annotated to the term
chromatin assembly or disassembly	47 of 412 genes, 11.4%	147 of 16085 genes, 0.9%	7.60e-36	0.00%	0.00	His3:CG33815, His1:CG33840, His4:CG33885, His3:CG33818, His2B:CG33884, His2B:CG33900, His3:CG33866, His3:CG33860, His1:CG33837, His3:CG31613, His3:CG33842, His4:CG33909, His1:CG33801, His3:CG33803, His4:CG33883, His2B:CG33886, His4:CG33877, His4:CG33869, His1:CG33834, His2B:CG17949, His2B:CG33902, His1:CG33861, His1:CG33843, His1:CG33849, His2B:CG33870, His1:CG31617, His4:CG33879, His4:CG33901, His2B:CG33878, His1:CG33864, His2B:CG33868, His2B:CG33910, His2B:CG33888, His2B:CG33880, His3:CG33845, His3:CG33839, His3:CG33851, His1:CG33813, His3:CG33848, His4:CG33899, His4:CG31611, His1:CG33819, His1:CG33816, His3:CG33836, His3:CG33863, His1:CG33846, His4:CG33887
nucleosome assembly	36 of 412 genes, 8.7%	99 of 16085 genes, 0.6%	2.97e-29	0.00%	0.00	His3:CG33815, His1:CG33840, His4:CG33885, His3:CG33818, His3:CG33866, His3:CG33860, His1:CG33837, His3:CG31613, His3:CG33842, His4:CG33909, His1:CG33801, His3:CG33803, His4:CG33883, His4:CG33877, His4:CG33869, His1:CG33834, His1:CG33861, His1:CG33849, His1:CG33843, His1:CG31617, His4:CG33901, His4:CG33879, His1:CG33864, His3:CG33845, His3:CG33839, His1:CG33813, His3:CG33848, His3:CG33851, His4:CG33899, His4:CG31611, His1:CG33819, His1:CG33816, His3:CG33836, His4:CG33887, His1:CG33846, His3:CG33863
chromatin assembly	36 of 412 genes, 8.7%	116 of 16085 genes, 0.7%	2.02e-26	0.00%	0.00	His3:CG33815, His1:CG33840, His4:CG33885, His3:CG33818, His3:CG33866, His3:CG33860, His1:CG33837, His3:CG31613, His3:CG33842, His4:CG33909, His1:CG33801, His3:CG33803, His4:CG33883, His4:CG33877, His4:CG33869, His1:CG33834, His1:CG33861, His1:CG33849, His1:CG33843, His1:CG31617, His4:CG33901, His4:CG33879, His1:CG33864, His3:CG33845, His3:CG33839, His1:CG33813, His3:CG33848,

						His3:CG33851, His4:CG33899, His4:CG31611, His1:CG33819, His1:CG33816, His3:CG33836, His4:CG33887, His1:CG33846, His3:CG33863
chromatin organization	64 of 412 genes, 15.5%	516 of 16085 genes, 3.2%	2.26e-23	0.00%	0.00	His3:CG33815, His2A:CG33850, SmydA-8, His1:CG33840, His4:CG33885, His2B:CG33884, His2A:CG33817, His2A:CG33841, His1:CG33837, His4:CG33909, His2A:CG33838, His2A:CG33865, His1:CG33801, His4:CG33883, His2B:CG33886, His4:CG33877, His4:CG33869, chif, His2B:CG33902, His1:CG33843, His1:CG33849, His4:CG33879, Usp7, His2B:CG33868, His2B:CG33888, His3:CG33839, His2A:CG33847, His3:CG33848, His4:CG33899, His1:CG33816, His3:CG33836, His3:CG33863, His1:CG33846, His4:CG33887, His2A:CG31618, Sfmbt, His3:CG33818, CG6220, His2B:CG33900, bon, His3:CG33866, His3:CG33860, His3:CG31613, His3:CG33842, His3:CG33803, His2A:CG33862, JMJD5, His1:CG33834, His2B:CG17949, His1:CG33861, His2B:CG33870, His2A:CG33814, His4:CG33901, His1:CG31617, His1:CG33864, His2B:CG33878, His2A:CG33835, His2B:CG33910, His2B:CG33880, His3:CG33845, His1:CG33813, His3:CG33851, His4:CG31611, His1:CG33819
nucleosome organization	36 of 412 genes, 8.7%	143 of 16085 genes, 0.9%	7.17e-23	0.00%	0.00	His3:CG33815, His1:CG33840, His4:CG33885, His3:CG33818, His3:CG33866, His3:CG33860, His1:CG33837, His3:CG31613, His3:CG33842, His4:CG33909, His1:CG33801, His3:CG33803, His4:CG33883, His4:CG33877, His4:CG33869, His1:CG33834, His1:CG33861, His1:CG33849, His1:CG33843, His1:CG31617, His4:CG33901, His4:CG33879, His1:CG33864, His3:CG33845, His3:CG33839, His1:CG33813, His3:CG33848, His3:CG33851, His4:CG33899, His4:CG31611, His1:CG33819, His1:CG33816, His3:CG33836, His4:CG33887, His1:CG33846, His3:CG33863
protein-DNA complex assembly	36 of 412 genes, 8.7%	155 of 16085 genes, 1.0%	1.48e-21	0.00%	0.00	His3:CG33815, His1:CG33840, His4:CG33885, His3:CG33818, His3:CG33866, His3:CG33860, His1:CG33837, His3:CG31613, His3:CG33842, His4:CG33909, His1:CG33801, His3:CG33803, His4:CG33883, His4:CG33877, His4:CG33869, His1:CG33834, His1:CG33861, His1:CG33849, His1:CG33843, His1:CG31617, His4:CG33901, His4:CG33879, His1:CG33864, His3:CG33845, His3:CG33839, His1:CG33813, His3:CG33848, His3:CG33851, His4:CG33899, His4:CG31611, His1:CG33819, His1:CG33816, His3:CG33836, His4:CG33887, His1:CG33846, His3:CG33863
DNA packaging	36 of 412 genes, 8.7%	184 of 16085 genes, 1.1%	7.69e-19	0.00%	0.00	His3:CG33815, His1:CG33840, His4:CG33885, His3:CG33818, His3:CG33866, His3:CG33860, His1:CG33837, His3:CG31613, His3:CG33842, His4:CG33909, His1:CG33801, His3:CG33803, His4:CG33883, His4:CG33877, His4:CG33869, His1:CG33834, His1:CG33861, His1:CG33849, His1:CG33843, His1:CG31617, His4:CG33901, His4:CG33879, His1:CG33864, His3:CG33845, His3:CG33839, His1:CG33813, His3:CG33848, His3:CG33851, His4:CG33899, His4:CG31611, His1:CG33819, His1:CG33816, His3:CG33836, His4:CG33887, His1:CG33846, His3:CG33863
protein-DNA complex subunit organization	37 of 412 genes, 9.0%	205 of 16085 genes, 1.3%	3.80e-18	0.00%	0.00	His3:CG33815, hay, His1:CG33840, His4:CG33885, His3:CG33818, His3:CG33866, His3:CG33860, His1:CG33837, His3:CG31613, His3:CG33842, His4:CG33909, His1:CG33801, His3:CG33803, His4:CG33883, His4:CG33877, His4:CG33869, His1:CG33834, His1:CG33861, His1:CG33849, His1:CG33843, His1:CG31617, His4:CG33879, His4:CG33901, His1:CG33864, His3:CG33845, His3:CG33839, His1:CG33813, His3:CG33848, His3:CG33851, His4:CG33899,

						His4:CG31611, His1:CG33819, His1:CG33816, His3:CG33836, His4:CG33887, His1:CG33846, His3:CG33863
chromosome organization	70 of 412 genes, 17.0%	762 of 16085 genes, 4.7%	4.31e-18	0.00%	0.00	His3:CG33815, His2A:CG33850, SmydA-8, His1:CG33840, His4:CG33885, tea, His2B:CG33884, His2A:CG33817, His2A:CG33841, His1:CG33837, His4:CG33909, His2A:CG33838, His2A:CG33865, His1:CG33801, His4:CG33883, His2B:CG33886, cuff, His4:CG33877, His4:CG33869, chif, His2B:CG33902, His1:CG33843, His1:CG33849, His4:CG33879, Usp7, His2B:CG33868, His2B:CG33888, c(3)G, His3:CG33839, His2A:CG33847, His3:CG33848, CG34001, His4:CG33899, His3:CG33836, His1:CG33816, His3:CG33863, His4:CG33887, His1:CG33846, His2A:CG31618, Sfmbt, CG15237, His3:CG33818, CG6220, His2B:CG33900, bon, His3:CG33866, His3:CG33860, His3:CG31613, His3:CG33842, His3:CG33803, His2A:CG33862, JMJD5, His1:CG33834, His2B:CG17949, vih, His1:CG33861, His2B:CG33870, His2A:CG33814, His1:CG31617, His4:CG33901, His1:CG33864, His2B:CG33878, His2A:CG33835, His2B:CG33910, His2B:CG33880, His3:CG33845, His1:CG33813, His3:CG33851, His4:CG31611, His1:CG33819
DNA conformation change	36 of 412 genes, 8.7%	209 of 16085 genes, 1.3%	6.74e-17	0.00%	0.00	His3:CG33815, His1:CG33840, His4:CG33885, His3:CG33818, His3:CG33866, His3:CG33860, His1:CG33837, His3:CG31613, His3:CG33842, His4:CG33909, His1:CG33801, His3:CG33803, His4:CG33883, His4:CG33877, His4:CG33869, His1:CG33834, His1:CG33861, His1:CG33849, His1:CG33843, His1:CG31617, His4:CG33901, His4:CG33879, His1:CG33864, His3:CG33845, His3:CG33839, His1:CG33813, His3:CG33848, His3:CG33851, His4:CG33899, His4:CG31611, His1:CG33819, His1:CG33816, His3:CG33836, His4:CG33887, His1:CG33846, His3:CG33863
protein-containing complex assembly	45 of 412 genes, 10.9%	602 of 16085 genes, 3.7%	1.02e-07	0.00%	0.00	CG17768, His3:CG33815, His1:CG33840, His4:CG33885, His3:CG33818, CG6220, His3:CG33866, His3:CG33860, His1:CG33837, His3:CG31613, Tom20, His3:CG33842, His4:CG33909, His1:CG33801, His3:CG33803, His4:CG33883, His4:CG33877, His4:CG33869, His1:CG33834, x16, His1:CG33861, His1:CG33843, His1:CG33849, His1:CG31617, His4:CG33879, His4:CG33901, His1:CG33864, atl, Shaw, CG13185, His3:CG33845, His3:CG33839, Prp8, COX6B, His1:CG33813, His3:CG33848, His3:CG33851, His4:CG33899, His4:CG31611, His1:CG33819, His1:CG33816, His3:CG33836, His3:CG33863, His1:CG33846, His4:CG33887
cellular protein-containing complex assembly	42 of 412 genes, 10.2%	559 of 16085 genes, 3.5%	3.84e-07	0.00%	0.00	CG17768, His3:CG33815, His1:CG33840, His4:CG33885, His3:CG33818, CG6220, His3:CG33866, His3:CG33860, His1:CG33837, His3:CG31613, Tom20, His3:CG33842, His4:CG33909, His1:CG33801, His3:CG33803, His4:CG33883, His4:CG33877, His4:CG33869, His1:CG33834, His1:CG33861, His1:CG33843, His1:CG33849, His1:CG31617, His4:CG33879, His4:CG33901, His1:CG33864, CG13185, His3:CG33845, His3:CG33839, Prp8, COX6B, His1:CG33813, His3:CG33848, His3:CG33851, His4:CG33899, His4:CG31611, His1:CG33819, His1:CG33816, His3:CG33836, His1:CG33846, His4:CG33887, His3:CG33863
protein-containing complex subunit	49 of 412 genes, 11.9%	733 of 16085 genes, 4.6%	6.82e-07	0.00%	0.00	CG17768, His3:CG33815, hay, His1:CG33840, His4:CG33885, Pxn, His3:CG33818, CG6220, His3:CG33866, His3:CG33860, His1:CG33837, His3:CG31613, Tom20, His3:CG33842,

organization						His4:CG33909, His1:CG33801, His3:CG33803, His4:CG33883, cuff, His4:CG33877, His4:CG33869, His1:CG33834, x16, His1:CG33861, His1:CG33843, His1:CG33849, His1:CG31617, His4:CG33879, His4:CG33901, His1:CG33864, atl, Shaw, CG13185, His3:CG33845, Atg8a, His3:CG33839, Prp8, COX6B, His3:CG33851, His1:CG33813, His3:CG33848, His4:CG33899, His4:CG31611, His1:CG33819, His1:CG33816, His3:CG33836, His3:CG33863, His1:CG33846, His4:CG33887
unannotated	135 of 412 genes, 32.8%	3459 of 16085 genes, 21.5%	4.95e-05	0.00%	0.00	asRNA:CR44083, lncRNA:CR32385, asRNA:CR44368, CG18327, CG15211, asRNA:CR43465, snmRNA:430c, CG8366, pre-rRNA:CR45845, CG15286, 2SrRNA:CR45840, CG5787, 18SrRNA:CR45841, snmRNA:430s, CG42322, CR45495, mir-3643, 18SrRNA:CR45838, mir-2500, CG15219, CG7943, lncRNA:CR44787, CG32369, CG8628, CG16986, CG3176, lncRNA:CR45039, asRNA:CR46256, snmRNA:430k, mir-10404-1, CG3823, CG7755, Sws1, CG1161, CG12592, CG2157, CG34166, CG30430, CG14005, lncRNA:CR45306, CR43383, CG10621, CR15821, 2SrRNA:CR45843, CG33932, CG12655, lncRNA:CR44999, CG32152, CG42231, CG9016, CR18166, CG7289, 2SrRNA-Psi:CR45850, CG13992, CG15829, mir-10404-2, CG16732, lncRNA:CR43423, CG44085, snmRNA:430n, snmRNA:430j, 18SrRNA:CR41548, CG15894, 28SrRNA:CR45844, lncRNA:CR44874, CG17068, CG1513, lncRNA:CR44741, CG15370, CG46310, snmRNA:430l, lncRNA:CR45038, His-Psi:CR33802, CR45498, lncRNA:CR44811, CR18275, snmRNA:430a, asRNA:CR44416, asRNA:CR45501, CG9068, snmRNA:430t, CR45458, snmRNA:430d, lncRNA:CR45037, asRNA:CR43908, asRNA:CR45137, CG17121, CR45033, 28SrRNA:CR45837, snoRNA:nop5-x16-a, lncRNA:CR45363, CG13983, CG31636, CG12121, lncRNA:CR45460, 28SrRNA-Psi:CR45862, CG31211, CG13177, CG10170, CG18273, CG3597, snmRNA:430o, CG32436, CG10834, CG8323, asRNA:CR44995, CR43382, lncRNA:CR45459, pre-rRNA:CR45856, lncRNA:CR45222, 28SrRNA-Psi:CR45859, His-Psi:CR33867, CG6511, 2SrRNA:CR45836, CG18324, asRNA:CR44047, 18SrRNA-Psi:CR41602, pre-rRNA:CR45846, mir-2282, 2SrRNA-Psi:CR45858, 18SrRNA-Psi:CR45861, CG8629, CG2186, CR43377, CG5151, prim, CG16762, lncRNA:CR45660, CG31751, CG12535, CG31703, Sry-beta, CG10011, CG31223, CG10168
organelle organization	92 of 412 genes, 22.3%	2093 of 16085 genes, 13.0%	8.56e-05	0.00%	0.00	His3:CG33815, His2A:CG33850, SmydA-8, His1:CG33840, His4:CG33885, tea, CG5984, His2B:CG33884, His2A:CG33817, Prosbeta7, His2A:CG33841, His1:CG33837, cep290, Tom20, scra, His4:CG33909, His2A:CG33838, CG45105, His2A:CG33865, His1:CG33801, His4:CG33883, His2B:CG33886, cuff, His4:CG33877, His4:CG33869, ReepB, chif, His2B:CG33902, His1:CG33843, His1:CG33849, His4:CG33879, TTLL3B, atl, Usp7, His2B:CG33868, His2B:CG33888, CG13185, c(3)G, Atg8a, His3:CG33839, COX6B, His2A:CG33847, His3:CG33848, CG34001, His4:CG33899, His3:CG33836, His1:CG33816, His3:CG33863, His4:CG33887, His1:CG33846, CG17768, His2A:CG31618, Sfmbt, CG15237, His3:CG33818, CG6220, His2B:CG33900, bon, His3:CG33866, His3:CG33860, His3:CG31613,

						His3:CG33842, Galphai, Sry-alpha, parvin, His3:CG33803, aPKC, His2A:CG33862, JMJD5, His1:CG33834, His2B:CG17949, vih, Tango4, His1:CG33861, His2B:CG33870, His2A:CG33814, His1:CG31617, His4:CG33901, His2B:CG33878, His1:CG33864, His2A:CG33835, Tim17a2, His2B:CG33910, His2B:CG33880, His3:CG33845, His1:CG33813, His3:CG33851, BBS1, His4:CG31611, Rab5, His1:CG33819, sle
DNA-templated transcription, initiation	13 of 412 genes, 3.2%	114 of 16085 genes, 0.7%	0.00648	0.12%	0.02	hay, His4:CG33909, x16, His4:CG33879, His4:CG33901, His4:CG33885, His4:CG33883, His4:CG31611, His4:CG33899, His4:CG33877, TfIIA-L, His4:CG33869, His4:CG33887
cellular component assembly	60 of 412 genes, 14.6%	1314 of 16085 genes, 8.2%	0.00714	0.12%	0.02	CG17768, His3:CG33815, His1:CG33840, His4:CG33885, Pxn, His3:CG33818, CG6220, His3:CG33866, His3:CG33860, His1:CG33837, cep290, His3:CG31613, Tom20, scra, His3:CG33842, Galphai, His4:CG33909, parvin, His1:CG33801, His3:CG33803, His4:CG33883, aPKC, cuff, His4:CG33877, His4:CG33869, His1:CG33834, chif, x16, His1:CG33861, His1:CG33843, His1:CG33849, Hrb27C, His1:CG31617, His4:CG33879, His4:CG33901, TTLL3B, His1:CG33864, atl, Shaw, CG13185, His3:CG33845, c(3)G, Atg8a, His3:CG33839, Prp8, COX6B, Got2, His3:CG33851, His3:CG33848, His1:CG33813, BBS1, His4:CG33899, His4:CG31611, His1:CG33819, Shark, His1:CG33816, His3:CG33836, His3:CG33863, His1:CG33846, His4:CG33887

Table S7. GO associations with Biological Process (GENERIC GENE ONTOLOGY (GO) TERM FINDER) of 135 rDNA-contacting genes shown in the Venn diagram in Fig. 5*B*. The genes decreased their number of contacts with rDNA.

Gene Ontology term	Cluster frequency	Genome frequency	Corrected P-value	FDR	False Positives	Genes annotated to the term
system development	48 of 132 genes, 36.4%	1999 of 16085 genes, 12.4%	1.07e-09	0.00%	0.00	cwo, Nedd4, bnl, apt, side, GEFmeso, pan, Nckx30C, fru, slou, Toll-7, Dg, PlexA, Mical, wech, Tom, wun, RunxA, CG14073, Eph, Octbeta2R, Dap160, Sdc, haf, LanB1, shot, Liprin-alpha, toy, Itp-r83A, Sobp, eg, tai, CadN, svr, Pkn, Nlg4, spin, hb, Nipped-A, IA-2, Pvf2, Atpalpha, caps, Sema1a, pico, Frl, tou, fry
plasma membrane bounded cell projection morphogenesis	21 of 132 genes, 15.9%	487 of 16085 genes, 3.0%	3.73e-07	0.00%	0.00	cwo, Nedd4, Sdc, haf, bnl, side, shot, Liprin-alpha, tai, CadN, fru, Toll-7, Dg, PlexA, Mical, RunxA, caps, Sema1a, Eph, Frl, fry
cell projection morphogenesis	21 of 132 genes, 15.9%	489 of 16085 genes, 3.0%	4.01e-07	0.00%	0.00	cwo, Nedd4, Sdc, haf, bnl, side, shot, Liprin-alpha, tai, CadN, fru, Toll-7, Dg, PlexA, Mical, RunxA, caps, Sema1a, Eph, Frl, fry
multicellular organism development	52 of 132 genes, 39.4%	2715 of 16085 genes, 16.9%	4.67e-07	0.00%	0.00	cwo, Nedd4, bnl, apt, side, sona, GEFmeso, pan, Nckx30C, fru, slou, Toll-7, Dg, PlexA, Mical, wech, Tom, Cpr50Ca, wun, RunxA, CG14073, Eph, Octbeta2R, Dap160, Sdc, haf, LanB1, shot, mgl, Liprin-alpha, toy, Itp-r83A, Sobp, eg, tai, CadN, svr, Pkn, Nlg4, spin, hb, Nipped-A, IA-2, Pvf2, Atpalpha, caps, Sema1a, pico, Frl, tou, Ac76E, fry

cell part morphogenesis	21 of 132 genes, 15.9%	497 of 16085 genes, 3.1%	5.39e-07	0.00%	0.00	cwo, Nedd4, Sdc, haf, bnl, side, shot, Liprin-alpha, tai, CadN, fru, Toll-7, Dg, PlexA, Mical, RunxA, caps, Sema1a, Eph, Frl, fry
cell morphogenesis	23 of 132 genes, 17.4%	608 of 16085 genes, 3.8%	6.51e-07	0.00%	0.00	cwo, Nedd4, Sdc, haf, bnl, LanB1, side, shot, Liprin-alpha, tai, CadN, pan, fru, Toll-7, Dg, PlexA, Mical, RunxA, caps, Sema1a, Eph, Frl, fry
nervous system development	32 of 132 genes, 24.2%	1205 of 16085 genes, 7.5%	1.59e-06	0.00%	0.00	cwo, Dap160, Nedd4, Sdc, haf, bnl, apt, side, shot, Liprin-alpha, toy, Itp-r83A, tai, eg, CadN, Nlg4, fru, Toll-7, Dg, PlexA, spin, Mical, hb, RunxA, Atpalpha, caps, Sema1a, Eph, Frl, tou, Octbeta2R, fry
cell morphogenesis involved in differentiation	21 of 132 genes, 15.9%	532 of 16085 genes, 3.3%	1.82e-06	0.00%	0.00	cwo, Nedd4, Sdc, haf, LanB1, side, shot, Liprin-alpha, tai, CadN, fru, Toll-7, Dg, PlexA, Mical, RunxA, caps, Sema1a, Eph, Frl, fry
cell morphogenesis involved in neuron differentiation	20 of 132 genes, 15.2%	485 of 16085 genes, 3.0%	2.18e-06	0.00%	0.00	cwo, Nedd4, Sdc, haf, side, shot, Liprin-alpha, tai, CadN, fru, Toll-7, Dg, PlexA, Mical, RunxA, caps, Sema1a, Eph, Frl, fry
neuron projection morphogenesis	20 of 132 genes, 15.2%	486 of 16085 genes, 3.0%	2.26e-06	0.00%	0.00	cwo, Nedd4, Sdc, haf, side, shot, Liprin-alpha, tai, CadN, fru, Toll-7, Dg, PlexA, Mical, RunxA, caps, Sema1a, Eph, Frl, fry
chemotaxis	16 of 132 genes, 12.1%	304 of 16085 genes, 1.9%	3.19e-06	0.00%	0.00	Nedd4, Sdc, haf, side, shot, Liprin-alpha, CadN, fru, Toll-7, Dg, PlexA, Mical, wun, caps, Sema1a, Eph
axonogenesis	17 of 132 genes, 12.9%	360 of 16085 genes, 2.2%	5.15e-06	0.00%	0.00	Nedd4, Sdc, haf, side, shot, Liprin-alpha, tai, CadN, fru, Toll-7, Dg, PlexA, Mical, caps, Sema1a, Eph, Frl
cellular component morphogenesis	24 of 132 genes, 18.2%	745 of 16085 genes, 4.6%	6.37e-06	0.00%	0.00	cwo, Nedd4, Sdc, haf, bnl, LanB1, side, shot, Zasp52, Liprin-alpha, tai, CadN, pan, fru, Toll-7, Dg, PlexA, Mical, RunxA, caps, Sema1a, Eph, Frl, fry
neuron projection development	20 of 132 genes, 15.2%	522 of 16085 genes, 3.2%	7.60e-06	0.00%	0.00	cwo, Nedd4, Sdc, haf, side, shot, Liprin-alpha, tai, CadN, fru, Toll-7, Dg, PlexA, Mical, RunxA, caps, Sema1a, Eph, Frl, fry
biological regulation	67 of 132 genes, 50.8%	4421 of 16085 genes, 27.5%	9.06e-06	0.00%	0.00	cwo, bnl, apt, sona, Ddr, Nckx30C, slou, Dg, wun, Calx, RunxA, FoxL1, Octbeta2R, Dap160, Liprin-alpha, Glut1, CG10413, Itp-r83A, tai, eg, svr, BtbVII, nAChRalpha7, Tg, Nipped-A, IA-2, Mid1, TpnC41C, Sema1a, Ac76E, RapGAP1, Nedd4, Strn-Mlck, Frq1, GEFmeso, corn, pan, fru, Toll-7, PlexA, Mical, wech, Tom, Eph, CG43366, Lkr, Sdc, LanB1, Myo81F, shot, mgl, Zasp52, toy, Aps, trv, CadN, Pkn, Nlg4, spin, hb, Pvf2, Atpalpha, pico, Samuel, tou, Tgi, fry
axon development	17 of 132 genes, 12.9%	375 of 16085 genes, 2.3%	9.44e-06	0.00%	0.00	Nedd4, Sdc, haf, side, shot, Liprin-alpha, tai, CadN, fru, Toll-7, Dg, PlexA, Mical, caps, Sema1a, Eph, Frl
axon guidance	15 of 132 genes, 11.4%	286 of 16085 genes, 1.8%	1.06e-05	0.00%	0.00	Toll-7, Dg, Nedd4, Sdc, PlexA, haf, Mical, side, shot, Liprin-alpha, caps, Sema1a, CadN, Eph, fru
anatomical structure development	54 of 132 genes, 40.9%	3173 of 16085 genes, 19.7%	1.40e-05	0.00%	0.00	cwo, Nedd4, bnl, apt, side, sona, GEFmeso, pan, Nckx30C, fru, slou, Toll-7, Dg, PlexA, Mical, wech, Tom, Cpr50Ca, wun, RunxA, FoxL1, CG14073, Eph, Octbeta2R, Dap160, Sdc, haf, LanB1, shot, mgl, Liprin-alpha, Zasp52, toy, Itp-r83A, Sobp, eg, tai, CadN, svr, Pkn, Nlg4, spin, hb, Nipped-A, IA-2, Pvf2, Atpalpha, caps, Sema1a, pico, Frl, tou, Ac76E, fry
neuron projection guidance	15 of 132 genes, 11.4%	295 of 16085 genes, 1.8%	1.60e-05	0.00%	0.00	Toll-7, Dg, Nedd4, Sdc, PlexA, haf, Mical, side, shot, Liprin-alpha, caps, Sema1a, CadN, Eph, fru
movement of cell or subcellular	23 of 132 genes, 17.4%	756 of 16085 genes, 4.7%	3.92e-05	0.00%	0.00	Nedd4, Sdc, haf, bnl, LanB1, apt, side, shot, Liprin-alpha, tai, CadN, Dnah3, fru, Toll-7, Dg,

component						PlexA, spin, Mical, wun, Pvf2, caps, Sema1a, Eph
developmental process	54 of 132 genes, 40.9%	3278 of 16085 genes, 20.4%	4.47e-05	0.00%	0.00	cwo, Nedd4, bnl, apt, side, sona, GEFmeso, pan, Nckx30C, fru, slou, Toll-7, Dg, PlexA, Mical, wech, Tom, Cpr50Ca, wun, RunxA, FoxL1, CG14073, Eph, Octbeta2R, Dap160, Sdc, haf, LanB1, shot, mgl, Liprin-alpha, Zasp52, toy, Itp-r83A, Sobp, eg, tai, CadN, svr, Pkn, Nlg4, spin, hb, Nipped-A, IA-2, Pvf2, Atpalpha, caps, Sema1a, pico, Frl, tou, Ac76E, fry
locomotion	22 of 132 genes, 16.7%	710 of 16085 genes, 4.4%	5.81e-05	0.00%	0.00	Nedd4, Sdc, haf, bnl, LanB1, apt, side, shot, Liprin-alpha, tai, CadN, fru, Toll-7, Dg, PlexA, spin, Mical, wun, Pvf2, caps, Sema1a, Eph
taxis	16 of 132 genes, 12.1%	383 of 16085 genes, 2.4%	8.13e-05	0.00%	0.00	Nedd4, Sdc, haf, side, shot, Liprin-alpha, CadN, fru, Toll-7, Dg, PlexA, Mical, wun, caps, Sema1a, Eph
regulation of biological process	60 of 132 genes, 45.5%	3934 of 16085 genes, 24.5%	8.63e-05	0.00%	0.00	cwo, Nedd4, bnl, apt, Frq1, sona, GEFmeso, Ddr, corn, pan, fru, slou, Toll-7, Dg, PlexA, Mical, wech, Tom, wun, Calx, RunxA, FoxL1, Eph, CG43366, Octbeta2R, Lkr, Dap160, Sdc, LanB1, shot, Myo81F, Zasp52, mgl, Liprin-alpha, toy, Glut1, Itp-r83A, eg, tai, CadN, trv, svr, Pkn, Nlg4, BtbVII, nAChRalpha7, spin, hb, Nipped-A, IA-2, Pvf2, TpnC41C, Sema1a, pico, Samuel, tou, Ac76E, RapGAP1, fry, Tgi
animal organ development	31 of 132 genes, 23.5%	1379 of 16085 genes, 8.6%	0.00014	0.00%	0.00	Nedd4, bnl, LanB1, apt, shot, Liprin-alpha, toy, Itp-r83A, GEFmeso, Sobp, CadN, svr, Pkn, pan, Nckx30C, fru, slou, Dg, wech, hb, Tom, Nipped-A, Pvf2, Atpalpha, caps, CG14073, Sema1a, Eph, pico, Frl, fry
anatomical structure morphogenesis	35 of 132 genes, 26.5%	1724 of 16085 genes, 10.7%	0.00022	0.00%	0.00	cwo, Nedd4, Sdc, haf, bnl, LanB1, side, shot, Zasp52, Liprin-alpha, toy, Itp-r83A, GEFmeso, tai, CadN, svr, Pkn, pan, fru, slou, Toll-7, Dg, PlexA, spin, Mical, Tom, RunxA, Pvf2, FoxL1, Atpalpha, caps, Sema1a, Eph, Frl, fry
plasma membrane bounded cell projection organization	21 of 132 genes, 15.9%	721 of 16085 genes, 4.5%	0.00033	0.00%	0.00	cwo, Nedd4, Sdc, haf, bnl, side, shot, Liprin-alpha, tai, CadN, fru, Toll-7, Dg, PlexA, Mical, RunxA, caps, Sema1a, Eph, Frl, fry
neuron development	20 of 132 genes, 15.2%	666 of 16085 genes, 4.1%	0.00040	0.00%	0.00	cwo, Nedd4, Sdc, haf, side, shot, Liprin-alpha, tai, CadN, fru, Toll-7, Dg, PlexA, Mical, RunxA, caps, Sema1a, Eph, Frl, fry
cell projection organization	21 of 132 genes, 15.9%	734 of 16085 genes, 4.6%	0.00044	0.00%	0.00	cwo, Nedd4, Sdc, haf, bnl, side, shot, Liprin-alpha, tai, CadN, fru, Toll-7, Dg, PlexA, Mical, RunxA, caps, Sema1a, Eph, Frl, fry
regulation of cellular process	55 of 132 genes, 41.7%	3609 of 16085 genes, 22.4%	0.00046	0.00%	0.00	cwo, Nedd4, bnl, apt, Frq1, sona, GEFmeso, Ddr, corn, pan, fru, slou, Toll-7, Dg, PlexA, Mical, Tom, Calx, RunxA, FoxL1, Eph, CG43366, Octbeta2R, Lkr, Dap160, Sdc, shot, Myo81F, mgl, Liprin-alpha, toy, Glut1, Itp-r83A, eg, tai, CadN, trv, svr, Pkn, Nlg4, BtbVII, nAChRalpha7, spin, hb, Nipped-A, Pvf2, TpnC41C, Sema1a, pico, Samuel, tou, Ac76E, RapGAP1, Tgi, fry
neurogenesis	24 of 132 genes, 18.2%	965 of 16085 genes, 6.0%	0.00078	0.00%	0.00	cwo, Dap160, Nedd4, Sdc, haf, bnl, side, shot, Liprin-alpha, tai, CadN, fru, Toll-7, Dg, PlexA, spin, Mical, hb, RunxA, caps, Sema1a, Eph, Frl, fry
cell communication	34 of 132 genes, 25.8%	1740 of 16085 genes, 10.8%	0.00084	0.00%	0.00	Nedd4, bnl, apt, Frq1, sona, GEFmeso, Ddr, pan, Toll-7, Dg, PlexA, Tom, Calx, Eph, Octbeta2R, Lkr, Dap160, Myo81F, shot, Liprin-alpha, Glut1, Itp-r83A, CadN, Pkn, Nlg4, nAChRalpha7, spin, Pvf2, Atpalpha, Sema1a, TpnC41C, pico, Ac76E, RapGAP1
synapse assembly	10 of 132	170 of 16085	0.00112	0.00%	0.00	Nedd4, Sdc, spin, Mical, Liprin-alpha, caps,

	genes, 7.6%	genes, 1.1%				Atpalpha, Sema1a, Nlg4, Octbeta2R
multicellular organismal process	60 of 132 genes, 45.5%	4222 of 16085 genes, 26.2%	0.00115	0.00%	0.00	cwo, Nedd4, bnl, apt, side, sona, GEFmeso, pan, Dnah3, Nckx30C, fru, slou, Toll-7, Dg, PlexA, Mical, wech, Tom, Cpr50Ca, wun, Calx, RunxA, CG14073, Eph, Or43a, BG642163, Octbeta2R, Dap160, Sdc, haf, LanB1, shot, mgl, Liprin-alpha, toy, Itp-r83A, Sobp, eg, tai, CadN, svr, Pkn, Nlg4, CG18135, nAChRalpha7, Tg, spin, hb, Nipped-A, IA-2, dpr17, Pvf2, Atpalpha, caps, Sema1a, pico, Frl, tou, Ac76E, fry
axon midline choice point recognition	5 of 132 genes, 3.8%	25 of 16085 genes, 0.2%	0.00131	0.00%	0.00	Nedd4, PlexA, Sema1a, shot, fru
axon choice point recognition	5 of 132 genes, 3.8%	27 of 16085 genes, 0.2%	0.00196	0.00%	0.00	Nedd4, PlexA, Sema1a, shot, fru
regulation of nervous system development	14 of 132 genes, 10.6%	374 of 16085 genes, 2.3%	0.00197	0.00%	0.00	cwo, Dap160, Nedd4, Sdc, PlexA, spin, Mical, shot, Liprin-alpha, hb, CadN, Sema1a, Octbeta2R, fry
response to external stimulus	24 of 132 genes, 18.2%	1019 of 16085 genes, 6.3%	0.00204	0.00%	0.00	Nedd4, Sdc, haf, LanB1, side, shot, Liprin-alpha, Itp-r83A, Aps, CadN, Dnah3, fru, Toll-7, Dg, PlexA, spin, Mical, Calx, wun, Atpalpha, caps, Sema1a, Eph, Ac76E
developmental growth	14 of 132 genes, 10.6%	379 of 16085 genes, 2.4%	0.00230	0.00%	0.00	Dg, Sdc, spin, shot, Liprin-alpha, Atpalpha, tai, CadN, Sema1a, pico, Frl, pan, Octbeta2R, Ac76E
regulation of localization	16 of 132 genes, 12.1%	494 of 16085 genes, 3.1%	0.00238	0.00%	0.00	Nedd4, bnl, Frq1, Liprin-alpha, mgl, Glut1, Itp-r83A, sona, tai, CadN, corn, Pkn, PlexA, Pvf2, IA-2, Sema1a
growth	15 of 132 genes, 11.4%	447 of 16085 genes, 2.8%	0.00324	0.00%	0.00	Dg, Sdc, spin, shot, Liprin-alpha, Atpalpha, tai, CadN, Sema1a, pico, Frl, Samuel, pan, Octbeta2R, Ac76E
signaling	32 of 132 genes, 24.2%	1694 of 16085 genes, 10.5%	0.00396	0.00%	0.00	Nedd4, bnl, apt, Frq1, sona, GEFmeso, Ddr, pan, Toll-7, Dg, PlexA, Tom, Calx, Eph, Octbeta2R, Lkr, Dap160, Myo81F, shot, Glut1, Itp-r83A, Pkn, Nlg4, nAChRalpha7, spin, Pvf2, Atpalpha, Sema1a, TpnC41C, pico, RapGAP1, Ac76E
axon extension	6 of 132 genes, 4.5%	55 of 16085 genes, 0.3%	0.00464	0.00%	0.00	tai, CadN, Sema1a, shot, Frl, Liprin-alpha
generation of neurons	22 of 132 genes, 16.7%	923 of 16085 genes, 5.7%	0.00472	0.00%	0.00	cwo, Dap160, Nedd4, Sdc, haf, side, shot, Liprin-alpha, tai, CadN, fru, Toll-7, Dg, PlexA, Mical, hb, RunxA, caps, Sema1a, Eph, Frl, fry
synapse organization	12 of 132 genes, 9.1%	299 of 16085 genes, 1.9%	0.00533	0.00%	0.00	Nedd4, Sdc, spin, Mical, Frq1, Liprin-alpha, dpr17, caps, Atpalpha, Sema1a, Nlg4, Octbeta2R
cell recognition	8 of 132 genes, 6.1%	122 of 16085 genes, 0.8%	0.00596	0.00%	0.00	Nedd4, PlexA, shot, igl, Sema1a, CadN, fru, fry
neuron projection extension	6 of 132 genes, 4.5%	58 of 16085 genes, 0.4%	0.00635	0.00%	0.00	tai, CadN, Sema1a, shot, Frl, Liprin-alpha
regulation of cell development	13 of 132 genes, 9.8%	364 of 16085 genes, 2.3%	0.00765	0.00%	0.00	cwo, Dap160, Nedd4, PlexA, spin, Mical, shot, Liprin-alpha, hb, tai, CadN, Sema1a, fry
regulation of multicellular organismal development	17 of 132 genes, 12.9%	617 of 16085 genes, 3.8%	0.00978	0.00%	0.00	cwo, Dap160, Nedd4, Sdc, bnl, shot, Liprin-alpha, mgl, tai, CadN, PlexA, spin, Mical, hb, Sema1a, Octbeta2R, fry

Table S8. GO associations with Biological Process (GENERIC GENE ONTOLOGY (GO) TERM FINDER) of 139 rDNA-contacting genes shown in the Venn diagram in Fig. 5*B*. The genes decreased their number of contacts with rDNA.

Gene Ontology term	Cluster frequency	Genome frequency	Corrected P-value	FDR	False Positives	Genes annotated to the term
animal organ morphogenesis	33 of 138 genes, 23.9%	849 of 16085 genes, 5.3%	1.37e-10	0.00%	0.00	NetA, Ire1, fz, gus, slow, psq, Egfr, dac, px, nmo, nrv2, Raf, pros, flw, CG34383, wg, ss, klar, Abd-B, chp, rdx, odd, Atg17, salr, CrebA, Pka-C1, scrib, MYPT-75D, RhoGAP100F, hdc, unc-5, otk2, ct
anatomical structure morphogenesis	45 of 138 genes, 32.6%	1724 of 16085 genes, 10.7%	2.90e-09	0.00%	0.00	oys, NetA, Ire1, fz, sdt, gus, slow, Myo10A, psq, Egfr, dac, px, nmo, nrv2, Raf, pros, zfh1, neo, flw, wg, CG34383, Tollo, ss, Abd-B, klar, chp, rdx, odd, CG32006, mmy, Atg17, salr, Appl, CrebA, Pka-C1, scrib, MYPT-75D, RhoGAP100F, hdc, unc-5, Acsl, otk2, bru1, Pax, ct
system development	48 of 138 genes, 34.8%	1999 of 16085 genes, 12.4%	8.44e-09	0.00%	0.00	NetA, Ire1, fz, sdt, gus, slow, psq, Egfr, dac, px, plum, Octbeta1R, nmo, nolo, nrv2, Raf, pros, zfh1, flw, wg, CG34383, Atf3, Tollo, ss, Abd-B, klar, chp, inaC, rdx, odd, Atg17, mmy, salr, Appl, CrebA, Pka-C1, scrib, MYPT-75D, RhoGAP100F, nab, serp, hdc, unc-5, Acsl, otk2, Con, Pax, ct
animal organ development	39 of 138 genes, 28.3%	1379 of 16085 genes, 8.6%	1.21e-08	0.00%	0.00	NetA, Ire1, fz, gus, slow, psq, Egfr, dac, px, nmo, nrv2, nolo, Raf, pros, zfh1, flw, wg, CG34383, ss, Abd-B, klar, chp, inaC, rdx, odd, Atg17, salr, Appl, CrebA, Pka-C1, scrib, MYPT-75D, RhoGAP100F, nab, hdc, unc-5, otk2, Pax, ct
morphogenesis of an epithelium	26 of 138 genes, 18.8%	672 of 16085 genes, 4.2%	9.00e-08	0.00%	0.00	odd, fz, rdx, mmy, salr, sdt, gus, slow, Pka-C1, Myo10A, scrib, psq, Egfr, MYPT-75D, dac, px, hdc, nmo, Raf, otk2, flw, CG34383, wg, Tollo, ss, ct
tissue morphogenesis	26 of 138 genes, 18.8%	691 of 16085 genes, 4.3%	1.65e-07	0.00%	0.00	odd, fz, rdx, mmy, salr, sdt, gus, slow, Pka-C1, Myo10A, scrib, psq, Egfr, MYPT-75D, dac, px, hdc, nmo, Raf, otk2, flw, CG34383, wg, Tollo, ss, ct
instar larval or pupal morphogenesis	21 of 138 genes, 15.2%	489 of 16085 genes, 3.0%	1.13e-06	0.00%	0.00	odd, fz, Atg17, salr, gus, slow, Pka-C1, scrib, psq, Egfr, MYPT-75D, dac, px, hdc, nmo, Raf, otk2, flw, wg, ss, ct
neuron differentiation	27 of 138 genes, 19.6%	823 of 16085 genes, 5.1%	1.44e-06	0.00%	0.00	NetA, Ire1, fz, mmy, Appl, sdt, slow, Pka-C1, scrib, Egfr, RhoGAP100F, nab, dac, plum, hdc, unc-5, Acsl, Raf, pros, zfh1, wg, Tollo, ss, Con, klar, chp, ct
instar larval or pupal development	23 of 138 genes, 16.7%	605 of 16085 genes, 3.8%	1.77e-06	0.00%	0.00	odd, fz, Atg17, salr, CrebA, gus, slow, Pka-C1, scrib, psq, Egfr, MYPT-75D, dac, px, serp, hdc, nmo, Raf, otk2, flw, wg, ss, ct
post-embryonic animal morphogenesis	21 of 138 genes, 15.2%	503 of 16085 genes, 3.1%	1.87e-06	0.00%	0.00	odd, fz, Atg17, salr, gus, slow, Pka-C1, scrib, psq, Egfr, MYPT-75D, dac, px, hdc, nmo, Raf, otk2, flw, wg, ss, ct
neuron development	24 of 138 genes, 17.4%	666 of 16085 genes, 4.1%	2.17e-06	0.00%	0.00	NetA, Ire1, fz, mmy, Appl, sdt, slow, Pka-C1, RhoGAP100F, nab, dac, plum, hdc, unc-5, Acsl, Raf, pros, zfh1, Tollo, ss, klar, Con, chp, ct
post-embryonic development	24 of 138 genes, 17.4%	676 of 16085 genes, 4.2%	2.91e-06	0.00%	0.00	odd, fz, Atg17, salr, CrebA, gus, slow, Pka-C1, scrib, psq, Egfr, MYPT-75D, dac, px, serp, hdc, nmo, Raf, otk2, flw, wg, ss, klar, ct
metamorphosis	21 of 138 genes, 15.2%	517 of 16085 genes, 3.2%	3.05e-06	0.00%	0.00	odd, fz, Atg17, salr, gus, slow, Pka-C1, scrib, psq, Egfr, MYPT-75D, dac, px, hdc, nmo, Raf, otk2, flw, wg, ss, ct
regionalization	21 of 138 genes, 15.2%	518 of 16085 genes, 3.2%	3.16e-06	0.00%	0.00	odd, fz, rdx, CrebA, gus, Pka-C1, scrib, psq, Egfr, nab, dac, nmo, unc-5, Acsl, Raf, flw, wg, klar, Abd-B, ss, ct
tube morphogenesis	22 of 138 genes, 15.9%	572 of 16085 genes, 3.6%	3.37e-06	0.00%	0.00	odd, fz, mmy, salr, gus, slow, Pka-C1, scrib, psq, Egfr, MYPT-75D, dac, px, hdc, nmo, Raf, otk2, flw, wg, klar, ss, ct

tissue development	32 of 138 genes, 23.2%	1186 of 16085 genes, 7.4%	4.26e-06	0.00%	0.00	NetA, fz, sdt, gus, slow, Myo10A, psq, Egfr, dac, px, nmo, Raf, zfh1, neo, flw, CG34383, wg, Tollo, ss, Abd-B, rdx, odd, mmy, salr, Pka-C1, scrib, MYPT-75D, nab, hdc, otk2, Pax, ct
epithelial tube morphogenesis	21 of 138 genes, 15.2%	527 of 16085 genes, 3.3%	4.29e-06	0.00%	0.00	odd, fz, mmy, salr, gus, slow, Pka-C1, scrib, psq, Egfr, MYPT-75D, dac, px, hdc, nmo, Raf, otk2, flw, wg, ss, ct
nervous system development	32 of 138 genes, 23.2%	1205 of 16085 genes, 7.5%	6.26e-06	0.00%	0.00	NetA, Ire1, fz, sdt, slow, Egfr, dac, plum, nmo, Octbeta1R, nrv2, nolo, Raf, pros, zfh1, wg, Atf3, Tollo, ss, klar, chp, mmy, Appl, Pka-C1, scrib, RhoGAP100F, nab, hdc, unc-5, Acsl, Con, ct
imaginal disc morphogenesis	19 of 138 genes, 13.8%	441 of 16085 genes, 2.7%	7.18e-06	0.00%	0.00	odd, fz, salr, gus, slow, Pka-C1, scrib, psq, Egfr, MYPT-75D, dac, px, nmo, Raf, otk2, flw, wg, ss, ct
post-embryonic animal organ morphogenesis	19 of 138 genes, 13.8%	441 of 16085 genes, 2.7%	7.18e-06	0.00%	0.00	odd, fz, salr, gus, slow, Pka-C1, scrib, psq, Egfr, MYPT-75D, dac, px, nmo, Raf, otk2, flw, wg, ss, ct
epithelium development	30 of 138 genes, 21.7%	1088 of 16085 genes, 6.8%	8.84e-06	0.00%	0.00	odd, fz, rdx, mmy, salr, sdt, gus, slow, Pka-C1, Myo10A, scrib, psq, Egfr, MYPT-75D, nab, dac, px, hdc, nmo, Raf, otk2, neo, flw, CG34383, wg, Tollo, ss, Abd-B, Pax, ct
neurogenesis	28 of 138 genes, 20.3%	965 of 16085 genes, 6.0%	1.02e-05	0.00%	0.00	NetA, Ire1, fz, mmy, Appl, sdt, slow, Pka-C1, scrib, Egfr, RhoGAP100F, nab, dac, plum, hdc, unc-5, Acsl, nrv2, Raf, pros, zfh1, wg, Tollo, ss, Con, klar, chp, ct
multicellular organism development	51 of 138 genes, 37.0%	2715 of 16085 genes, 16.9%	1.08e-05	0.00%	0.00	oys, NetA, Ire1, fz, sdt, gus, slow, Myo10A, psq, Egfr, dac, px, plum, Octbeta1R, nmo, nolo, nrv2, Raf, pros, zfh1, neo, flw, wg, CG34383, Atf3, Tollo, ss, Abd-B, klar, chp, inaC, rdx, odd, Atg17, mmy, salr, Appl, CrebA, Pka-C1, scrib, MYPT-75D, RhoGAP100F, nab, serp, hdc, unc-5, Acsl, otk2, Con, Pax, ct
pattern specification process	21 of 138 genes, 15.2%	559 of 16085 genes, 3.5%	1.20e-05	0.00%	0.00	odd, fz, rdx, CrebA, gus, Pka-C1, scrib, psq, Egfr, nab, dac, nmo, unc-5, Acsl, Raf, flw, wg, klar, Abd-B, ss, ct
post-embryonic animal organ development	20 of 138 genes, 14.5%	507 of 16085 genes, 3.2%	1.23e-05	0.00%	0.00	odd, fz, salr, gus, slow, Pka-C1, scrib, psq, Egfr, MYPT-75D, dac, px, nmo, Raf, otk2, flw, wg, klar, ss, ct
generation of neurons	27 of 138 genes, 19.6%	923 of 16085 genes, 5.7%	1.66e-05	0.00%	0.00	NetA, Ire1, fz, mmy, Appl, sdt, slow, Pka-C1, scrib, Egfr, RhoGAP100F, nab, dac, plum, hdc, unc-5, Acsl, Raf, pros, zfh1, wg, Tollo, ss, Con, klar, chp, ct
tube development	25 of 138 genes, 18.1%	801 of 16085 genes, 5.0%	1.73e-05	0.00%	0.00	odd, fz, mmy, salr, gus, slow, Pka-C1, scrib, psq, Egfr, MYPT-75D, nab, dac, px, hdc, nmo, Raf, otk2, flw, wg, ss, klar, Abd-B, Pax, ct
imaginal disc development	22 of 138 genes, 15.9%	631 of 16085 genes, 3.9%	2.00e-05	0.00%	0.00	odd, fz, salr, gus, slow, Pka-C1, scrib, psq, Egfr, MYPT-75D, nab, dac, px, nmo, Raf, otk2, flw, wg, Abd-B, ss, Pax, ct
imaginal disc-derived appendage morphogenesis	17 of 138 genes, 12.3%	375 of 16085 genes, 2.3%	2.25e-05	0.00%	0.00	odd, fz, salr, slow, Pka-C1, scrib, psq, Egfr, MYPT-75D, dac, px, nmo, Raf, wg, flw, ss, ct
appendage morphogenesis	17 of 138 genes, 12.3%	378 of 16085 genes, 2.4%	2.53e-05	0.00%	0.00	odd, fz, salr, slow, Pka-C1, scrib, psq, Egfr, MYPT-75D, dac, px, nmo, Raf, wg, flw, ss, ct
imaginal disc-derived appendage development	17 of 138 genes, 12.3%	382 of 16085 genes, 2.4%	2.96e-05	0.00%	0.00	odd, fz, salr, slow, Pka-C1, scrib, psq, Egfr, MYPT-75D, dac, px, nmo, Raf, wg, flw, ss, ct
appendage development	17 of 138 genes, 12.3%	386 of 16085 genes, 2.4%	3.44e-05	0.00%	0.00	odd, fz, salr, slow, Pka-C1, scrib, psq, Egfr, MYPT-75D, dac, px, nmo, Raf, wg, flw, ss, ct
cell development	36 of 138 genes, 26.1%	1606 of 16085 genes, 10.0%	4.80e-05	0.00%	0.00	oys, NetA, Ire1, fz, sdt, gus, slow, psq, Egfr, dac, plum, nrv2, Raf, pros, zfh1, neo, flw, wg, Tollo, ss, klar, chp, mmy, Atg17, Appl, Pka-C1, scrib, RhoGAP100F, nab, unc-5, hdc, Acsl, bru1, Con, Pax, ct

segmentation	14 of 138 genes, 10.1%	266 of 16085 genes, 1.7%	6.83e-05	0.00%	0.00	odd, fz, rdx, Acsl, Raf, wg, gus, ss, Pka-C1, klar, Abd-B, scrib, psq, Egfr
post-embryonic appendage morphogenesis	16 of 138 genes, 11.6%	364 of 16085 genes, 2.3%	9.21e-05	0.00%	0.00	odd, fz, salr, slow, Pka-C1, scrib, psq, Egfr, MYPT-75D, px, nmo, Raf, wg, flw, ss, ct
multicellular organismal process	65 of 138 genes, 47.1%	4222 of 16085 genes, 26.2%	0.00010	0.00%	0.00	oys, CG32395, NetA, Ire1, fz, shakB, Ktl, sdt, gus, slow, Myo10A, CG10000, psq, Egfr, dac, px, plum, Octbeta1R, nmo, 5-HT1A, nAChRalpha4, nolo, nrv2, Raf, pros, zfh1, neo, flw, wg, CG34383, Atf3, Tollo, ss, Abd-B, klar, pHCl-2, chp, pxb, inaC, odd, rdx, Atg17, mmy, salr, Appl, MsR2, lncRNA:CR44344, CrebA, Pka-C1, scrib, MYPT-75D, RhoGAP100F, nab, serp, CG9400, unc-5, hdc, Acsl, GluClalpha, otk2, CrzR, bru1, Con, Pax, ct
wing disc morphogenesis	15 of 138 genes, 10.9%	325 of 16085 genes, 2.0%	0.00012	0.00%	0.00	MYPT-75D, fz, px, nmo, salr, Raf, flw, wg, gus, slow, Pka-C1, scrib, ct, Egfr, psq
wing disc development	17 of 138 genes, 12.3%	427 of 16085 genes, 2.7%	0.00014	0.00%	0.00	fz, salr, gus, slow, Pka-C1, scrib, psq, Egfr, MYPT-75D, nab, px, nmo, Raf, wg, flw, Pax, ct
anatomical structure development	53 of 138 genes, 38.4%	3173 of 16085 genes, 19.7%	0.00027	0.00%	0.00	oys, NetA, Ire1, fz, sdt, gus, slow, Myo10A, psq, Egfr, dac, px, plum, Octbeta1R, nmo, nolo, nrv2, Raf, pros, zfh1, neo, flw, wg, CG34383, Atf3, Tollo, ss, Abd-B, klar, chp, inaC, rdx, odd, CG32006, Atg17, mmy, salr, Appl, CrebA, Pka-C1, scrib, MYPT-75D, RhoGAP100F, nab, serp, hdc, unc-5, Acsl, otk2, bru1, Con, Pax, ct
developmental process	54 of 138 genes, 39.1%	3278 of 16085 genes, 20.4%	0.00030	0.00%	0.00	oys, NetA, Ire1, fz, sdt, gus, slow, Myo10A, psq, Egfr, dac, px, plum, Octbeta1R, nmo, nolo, nrv2, Raf, pros, zfh1, neo, flw, wg, CG34383, Atf3, Tollo, ss, Abd-B, klar, chp, pxb, inaC, rdx, odd, CG32006, Atg17, mmy, salr, Appl, CrebA, Pka-C1, scrib, MYPT-75D, RhoGAP100F, nab, serp, hdc, unc-5, Acsl, otk2, bru1, Con, Pax, ct
cell differentiation	38 of 138 genes, 27.5%	1890 of 16085 genes, 11.8%	0.00031	0.00%	0.00	oys, NetA, Ire1, fz, sdt, gus, slow, psq, Egfr, dac, plum, nrv2, Raf, pros, zfh1, neo, flw, wg, Tollo, ss, klar, Abd-B, chp, CG32006, mmy, Atg17, Appl, Pka-C1, scrib, RhoGAP100F, nab, hdc, unc-5, Acsl, bru1, Con, Pax, ct
cellular component morphogenesis	22 of 138 genes, 15.9%	745 of 16085 genes, 4.6%	0.00036	0.00%	0.00	NetA, Ire1, fz, mmy, Appl, gus, scrib, Egfr, RhoGAP100F, dac, hdc, unc-5, Acsl, pros, Raf, zfh1, bru1, neo, wg, ss, Pax, ct
blastoderm segmentation	12 of 138 genes, 8.7%	219 of 16085 genes, 1.4%	0.00040	0.00%	0.00	odd, fz, rdx, Raf, wg, gus, Pka-C1, klar, Abd-B, scrib, psq, Egfr
sex differentiation	9 of 138 genes, 6.5%	110 of 16085 genes, 0.7%	0.00042	0.00%	0.00	inaC, otk2, zfh1, dac, wg, Abd-B, salr, Egfr, ct
imaginal disc-derived wing morphogenesis	14 of 138 genes, 10.1%	315 of 16085 genes, 2.0%	0.00053	0.00%	0.00	MYPT-75D, fz, px, nmo, salr, Raf, flw, wg, slow, Pka-C1, scrib, ct, Egfr, psq
photoreceptor cell differentiation	12 of 138 genes, 8.7%	226 of 16085 genes, 1.4%	0.00056	0.00%	0.00	RhoGAP100F, Ire1, fz, dac, pros, Raf, sdt, ss, klar, chp, scrib, Egfr
cellular developmental process	38 of 138 genes, 27.5%	1938 of 16085 genes, 12.0%	0.00059	0.00%	0.00	oys, NetA, Ire1, fz, sdt, gus, slow, psq, Egfr, dac, plum, nrv2, Raf, pros, zfh1, neo, flw, wg, Tollo, ss, klar, Abd-B, chp, CG32006, mmy, Atg17, Appl, Pka-C1, scrib, RhoGAP100F, nab, hdc, unc-5, Acsl, bru1, Con, Pax, ct
compound eye development	16 of 138 genes, 11.6%	421 of 16085 genes, 2.6%	0.00065	0.00%	0.00	NetA, Ire1, fz, rdx, Pka-C1, scrib, Egfr, RhoGAP100F, dac, nmo, pros, Raf, wg, klar, ss, chp
compound eye photoreceptor cell differentiation	11 of 138 genes, 8.0%	192 of 16085 genes, 1.2%	0.00080	0.00%	0.00	RhoGAP100F, Ire1, fz, dac, pros, Raf, klar, ss, chp, scrib, Egfr
embryonic pattern specification	12 of 138 genes, 8.7%	236 of 16085 genes, 1.5%	0.00089	0.00%	0.00	odd, fz, rdx, Raf, wg, gus, Pka-C1, klar, Abd-B, scrib, psq, Egfr

regulation of developmental process	22 of 138 genes, 15.9%	787 of 16085 genes, 4.9%	0.00091	0.00%	0.00	NetA, fz, mmy, Pka-C1, scrib, Egfr, plum, hdc, nmo, Octbeta1R, Raf, pros, zfh1, neo, bru1, CG34383, wg, ss, klar, Abd-B, Pax, pxb
regulation of cellular process	56 of 138 genes, 40.6%	3609 of 16085 genes, 22.4%	0.00123	0.00%	0.00	NetA, Ire1, fz, Crtc, shakB, SNF4Agamma, Pkcdelta, sdt, gus, CG30158, psq, Egfr, dac, plum, Octbeta1R, nmo, 5-HT1A, nAChRalpha4, Raf, pros, zfh1, neo, flw, wg, Atf3, Tollo, ss, Abd-B, klar, pHCl-2, pxb, inaC, odd, rdx, CG32006, Atg17, mmy, salr, Appl, MsR2, Gbp3, CrebA, Pka-C1, TyrRII, scrib, RhoGAP102A, RhoGAP100F, nab, unc-5, hdc, GluClalpha, CrzR, bru1, Pax, ct, GluRIIC
regulation of multicellular organismal process	22 of 138 genes, 15.9%	804 of 16085 genes, 5.0%	0.00130	0.00%	0.00	NetA, fz, mmy, Pka-C1, scrib, Egfr, plum, hdc, nmo, Octbeta1R, 5-HT1A, Raf, pros, neo, bru1, CG34383, wg, ss, pHCl-2, klar, Abd-B, pxb
eye photoreceptor cell differentiation	11 of 138 genes, 8.0%	203 of 16085 genes, 1.3%	0.00139	0.00%	0.00	RhoGAP100F, Ire1, fz, dac, pros, Raf, klar, ss, chp, scrib, Egfr
regulation of biological process	59 of 138 genes, 42.8%	3934 of 16085 genes, 24.5%	0.00167	0.00%	0.00	NetA, Ire1, fz, Crtc, shakB, Ktl, SNF4Agamma, Pkcdelta, sdt, gus, CG30158, psq, Egfr, dac, plum, Octbeta1R, nmo, 5-HT1A, nAChRalpha4, Raf, pros, zfh1, neo, flw, wg, CG34383, Atf3, Tollo, ss, Abd-B, klar, pHCl-2, pxb, inaC, odd, rdx, CG32006, Atg17, mmy, salr, Appl, MsR2, Gbp3, CrebA, Pka-C1, TyrRII, scrib, RhoGAP102A, RhoGAP100F, nab, unc-5, hdc, Acsl, GluClalpha, CrzR, bru1, Pax, ct, GluRIIC
compound eye morphogenesis	14 of 138 genes, 10.1%	347 of 16085 genes, 2.2%	0.00170	0.00%	0.00	RhoGAP100F, Ire1, fz, rdx, dac, nmo, Raf, pros, wg, ss, klar, chp, scrib, Egfr
eye development	16 of 138 genes, 11.6%	455 of 16085 genes, 2.8%	0.00181	0.00%	0.00	NetA, Ire1, fz, rdx, Pka-C1, scrib, Egfr, RhoGAP100F, dac, nmo, pros, Raf, wg, klar, ss, chp
sensory system development	16 of 138 genes, 11.6%	455 of 16085 genes, 2.8%	0.00181	0.00%	0.00	NetA, Ire1, fz, rdx, Pka-C1, scrib, Egfr, RhoGAP100F, dac, nmo, pros, Raf, wg, klar, ss, chp
visual system development	16 of 138 genes, 11.6%	455 of 16085 genes, 2.8%	0.00181	0.00%	0.00	NetA, Ire1, fz, rdx, Pka-C1, scrib, Egfr, RhoGAP100F, dac, nmo, pros, Raf, wg, klar, ss, chp
locomotion	20 of 138 genes, 14.5%	710 of 16085 genes, 4.4%	0.00269	0.00%	0.00	inaC, oys, NetA, fz, Ktl, mmy, gus, Egfr, RhoGAP100F, dac, nmo, unc-5, Acsl, pros, Raf, zfh1, klar, Abd-B, Con, ct
eye morphogenesis	14 of 138 genes, 10.1%	369 of 16085 genes, 2.3%	0.00348	0.00%	0.00	RhoGAP100F, Ire1, fz, rdx, dac, nmo, Raf, pros, wg, ss, klar, chp, scrib, Egfr
sensory organ morphogenesis	14 of 138 genes, 10.1%	369 of 16085 genes, 2.3%	0.00348	0.00%	0.00	RhoGAP100F, Ire1, fz, rdx, dac, nmo, Raf, pros, wg, ss, klar, chp, scrib, Egfr
biological regulation	63 of 138 genes, 45.7%	4421 of 16085 genes, 27.5%	0.00350	0.00%	0.00	NetA, Ire1, fz, Crtc, shakB, Ktl, SNF4Agamma, Pkcdelta, sdt, gus, slow, CG30158, psq, Egfr, dac, plum, Octbeta1R, nmo, 5-HT1A, nAChRalpha4, nrv2, Raf, pros, zfh1, neo, flw, wg, CG34383, Atf3, Tollo, ss, Abd-B, klar, pHCl-2, pxb, inaC, CG1695, odd, rdx, CG32006, Atg17, mmy, salr, Appl, MsR2, Gbp3, CrebA, Pka-C1, TyrRII, scrib, RhoGAP102A, RhoGAP100F, nab, serp, unc-5, hdc, Acsl, GluClalpha, CrzR, bru1, Pax, ct, GluRIIC
cell projection organization	20 of 138 genes, 14.5%	734 of 16085 genes, 4.6%	0.00444	0.00%	0.00	NetA, Ire1, fz, mmy, Appl, Pka-C1, Myo10A, scrib, Egfr, RhoGAP100F, dac, hdc, unc-5, Acsl, pros, zfh1, Con, ss, chp, ct
sensory organ development	17 of 138 genes, 12.3%	547 of 16085 genes, 3.4%	0.00447	0.00%	0.00	NetA, Ire1, fz, rdx, salr, Pka-C1, scrib, Egfr, RhoGAP100F, dac, nmo, pros, Raf, wg, klar, ss, chp
gland development	10 of 138 genes, 7.2%	189 of 16085 genes, 1.2%	0.00528	0.00%	0.00	fz, Atg17, unc-5, hdc, Raf, zfh1, CrebA, wg, Abd-B, Egfr
embryo	18 of 138	615 of 16085	0.00528	0.00%	0.00	odd, fz, rdx, mmy, sdt, gus, Pka-C1, Myo10A,

development	genes, 13.0%	genes, 3.8%				scrib, psq, Egfr, Raf, zfh1, neo, wg, klar, Abd-B, ct
regulation of multicellular organismal development	18 of 138 genes, 13.0%	617 of 16085 genes, 3.8%	0.00552	0.00%	0.00	NetA, fz, mmy, Pka-C1, scrib, Egfr, plum, hdc, nmo, Octbeta1R, pros, Raf, neo, CG34383, wg, klar, Abd-B, ss
genital disc sexually dimorphic development	3 of 138 genes, 2.2%	5 of 16085 genes, 0.0%	0.00605	0.03%	0.02	dac, wg, Abd-B
antennal joint development	3 of 138 genes, 2.2%	5 of 16085 genes, 0.0%	0.00605	0.03%	0.02	dac, salr, ct
system process	19 of 138 genes, 13.8%	685 of 16085 genes, 4.3%	0.00613	0.03%	0.02	inaC, CG32395, Atg17, salr, Appl, Pka-C1, scrib, Egfr, nAChRalpha4, 5-HT1A, GluClalpha, nrv2, pros, bru1, wg, pHCl-2, ss, pxb, ct
movement of cell or subcellular component	20 of 138 genes, 14.5%	756 of 16085 genes, 4.7%	0.00690	0.03%	0.02	inaC, oys, NetA, fz, mmy, gus, Egfr, RhoGAP100F, dac, Dhc98D, unc-5, Acsl, pros, Raf, zfh1, neo, Abd-B, klar, kl-5, ct
photoreceptor cell development	8 of 138 genes, 5.8%	116 of 16085 genes, 0.7%	0.00692	0.03%	0.02	Raf, sdt, RhoGAP100F, Ire1, dac, ss, klar, chp
regulation of cell differentiation	15 of 138 genes, 10.9%	452 of 16085 genes, 2.8%	0.00780	0.03%	0.02	NetA, fz, mmy, hdc, Raf, pros, zfh1, bru1, wg, ss, Abd-B, klar, Pka-C1, Pax, Egfr
cell communication	33 of 138 genes, 23.9%	1740 of 16085 genes, 10.8%	0.00836	0.03%	0.02	inaC, Ire1, fz, rdx, Atg17, shakB, SNF4Agamma, Pkcdelta, MsR2, Gbp3, gus, CG1909, CG30158, Pka-C1, TyrRII, scrib, Egfr, RhoGAP102A, RhoGAP100F, unc-5, nmo, Octbeta1R, 5-HT1A, Acsl, nAChRalpha4, GluClalpha, Raf, CrzR, flw, wg, Tollo, pHCl-2, GluRIIC

Table S9. GO associations with Biological Process (GENERIC GENE ONTOLOGY (GO) TERM FINDER) of 878 rDNA-contacting genes shown in the Venn diagram in Fig. 5*B* and in the diagram in Fig. 5*D*. The genes increased their number of contacts with rDNA.

Gene Ontology term	Cluster frequency	Genome frequency	Corrected P-value	FDR	False Positives	Genes annotated to the term
anatomical structure morphogenesis	223 of 856 genes, 26.1%	1724 of 16085 genes, 10.7%	1.69e-35	0.00%	0.00	fz2, sbb, Sh, Npc1b, dome, ec, kuz, jing, caup, Fs(2)Ket, lilli, CG42674, dpy, CG5921, CLIP-190, osa, alpha-Cat, Dys, Trim9, Gnf1, pwn, crb, robo3, pyr, sns, hid, if, Mef2, inv, Ten-a, cno, Ptp99A, lab, poe, ap, C3G, jvl, Gprk2, bdg, nw, sano, cv-c, Snoo, DIP-gamma, rut, Nrg, pum, Pka-C3, uif, Mmp2, amon, trol, alph, msi, CG30456, Src42A, beat-IIb, beat-Ib, CG13251, ds, dysc, hth, CG43658, ft, qua, Doa, sas, ara, Ser, FER, S, mam, Ptp61F, shn, foxo, DAAM, grn, Scgdelta, neur, mew, beat-Vc, Prosap, app, Pka-R2, bun, ed, CadN2, opa, kay, InR, pdm3, f, tinc, PDZ-GEF, l(3)psg2, nerfin-1, wnd, RhoBTB, Stat92E, Src64B, Btk29A, CG6701, dsx, Sema2a, Bsg, cdi, klu, loco, elB, fra, Lim1, Parp, lola, vkg, boss, TfAP-2, vvl, abd-A, nau, mbl, Duox, stl, bab2, CalpA, PyK, egh, pnt, melt, jbug, Hs6st, corto, ltl, smog, cv-2, T48, rost, CG41099, rhea, dia, lmd, ich, AdamTS-A, disco-r, Debcl, hh, drl, pot, Oaz, l(2)gl, Dr, Grip, Drl-2, csw, ex, ush, beat-Ic, Poxm, Mbs, RasGAP1, spz3, ko, beat-Va, CtBP, tyn, stan, ckn, cta, futsch, beat-VI, IP3K2, gukh, cora, spir, sff, zen, beat-IIa, BicD, Dhc64C, kibra, al, EcR,

						Rok, Ten-m, e(y)3, nej, chrb, ACC, Mmp1, ey, Pura, dlg1, step, par-1, spri, heph, siz, Hipk, Abl, chas, hbs, Lim3, Unc-115a, Dad, cic, lncRNA:acal, crol, sd, RhoGEF64C, RecQ4, en, ths, fs(1)h, sfl, beat-IIIb, Rbfox1, kirre, rl, vn, TwdlQ, cher, Fas3, pyd, unk
neurogenesis	157 of 856 genes, 18.3%	965 of 16085 genes, 6.0%	5.05e-35	0.00%	0.00	fz2, sbb, Sh, kuz, jing, lilli, CG5921, osa, Trim9, Gnf1, crb, robo3, pyr, hid, if, inv, Ten-a, cno, Ptp99A, poe, ap, Awh, bdg, cv-c, Snoo, rut, DIP-gamma, Nrg, pum, Mmp2, amon, trol, alph, msi, Src42A, beat-IIb, beat-Ib, stau, CG13251, ds, dysc, hth, ft, qua, Doa, sas, ara, Ser, FER, S, mam, Ptp61F, shep, shn, foxo, DAAM, grn, neur, mew, beat-Vc, Prosap, app, Pka-R2, bun, ed, CadN2, pdm3, InR, kay, f, tinc, PDZ-GEF, nerfin-1, wnd, Src64B, CG6701, dsx, Sema2a, chinmo, Bsg, cdi, klu, loco, elB, fra, Lim1, nkd, lola, boss, vvl, abd-A, dpr12, mbl, stl, stai, CalpA, egh, pnt, melt, jbug, esn, hh, drl, pot, l(2)gl, Dr, Fur1, csw, Drl-2, ex, beat-Ic, Poxm, Mbs, RasGAP1, Antp, ko, beat-Va, stan, ckn, futsch, ced-6, beat-VI, wake, gukh, cora, sff, beat-IIa, kibra, Dhc64C, EcR, Rok, Ten-m, nej, Mmp1, ey, Pura, par-1, spri, Abl, hbs, Lim3, Unc-115a, cac, Dad, Patronin, sd, RhoGEF64C, en, fs(1)h, ths, beat-IIIb, rl, cher, Tl, Fas3, CG5758, unk
generation of neurons	153 of 856 genes, 17.9%	923 of 16085 genes, 5.7%	5.33e-35	0.00%	0.00	fz2, sbb, Sh, kuz, jing, lilli, CG5921, osa, Trim9, Gnf1, crb, robo3, pyr, hid, if, inv, Ten-a, Ptp99A, ap, Awh, bdg, cv-c, Snoo, rut, DIP-gamma, Nrg, pum, Mmp2, amon, trol, alph, msi, Src42A, beat-IIb, beat-Ib, stau, CG13251, ds, dysc, hth, ft, qua, Doa, sas, ara, FER, S, mam, Ptp61F, shep, shn, foxo, DAAM, grn, neur, mew, beat-Vc, Prosap, app, Pka-R2, bun, ed, CadN2, pdm3, InR, kay, f, tinc, PDZ-GEF, nerfin-1, wnd, Src64B, CG6701, dsx, Sema2a, chinmo, Bsg, cdi, klu, loco, elB, fra, Lim1, nkd, lola, boss, vvl, abd-A, dpr12, mbl, stl, CalpA, stai, egh, pnt, melt, jbug, esn, hh, drl, pot, l(2)gl, Dr, Fur1, csw, Drl-2, ex, beat-Ic, Poxm, Mbs, RasGAP1, Antp, ko, beat-Va, stan, ckn, futsch, ced-6, beat-VI, wake, gukh, cora, sff, beat-IIa, kibra, Dhc64C, EcR, Rok, Ten-m, nej, Mmp1, ey, Pura, par-1, spri, Abl, hbs, Lim3, Unc-115a, cac, Dad, Patronin, sd, RhoGEF64C, en, fs(1)h, beat-IIIb, rl, cher, Tl, Fas3, CG5758, unk
system development	243 of 856 genes, 28.4%	1999 of 16085 genes, 12.4%	9.20e-35	0.00%	0.00	fz2, sbb, Sh, Npc1b, dome, ec, Nlg1, kuz, jing, caup, lilli, CG42674, dpy, CG5921, Sxl, osa, l(3)72Ab, Dys, Trim9, Gnf1, Nlg3, pwn, crb, robo3, pyr, sns, hid, if, Mef2, inv, Ten-a, cno, Ptp99A, lab, poe, ap, C3G, Gprk2, Awh, bdg, nw, sano, Sox21a, cv-c, Snoo, DIP-gamma, rut, Nrg, crp, pum, Pka-C3, uif, Mmp2, amon, Blimp-1, trol, alph, msi, CG30456, Src42A, beat-IIb, beat-Ib, stau, CG13251, ds, dysc, hth, CG43658, mtg, ft, qua, Doa, sas, ara, Ser, FER, S, mam, Ptp61F, shep, PsGEF, shn, foxo, DAAM, grn, Scgdelta, neur, mew, beat-Vc, Sox100B, Prosap, app, Pka-R2, bun, Tao, ed, CadN2, loh, opa, kay, InR, pdm3, sima, f, tinc, Tsp, PDZ-GEF, l(3)psg2, nerfin-1, wnd, Stat92E, Src64B, Btk29A, CG6701, dsx, Sema2a, chinmo, Bsg, cdi, klu, loco, elB, fra, Lim1, nkd, lola, Smr, vkg, boss, Sema5c, TfAP-2, vvl, Fife, abd-A, dpr12,

						nau, mbl, Duox, stl, bab2, stai, CalpA, CG5890, egh, Gie, pnt, melt, jbug, Hs6st, corto, ltl, cv-2, Msp300, Sb, rhea, dia, lmd, ich, esn, AdamTS-A, disco-r, hh, drl, pot, Oaz, l(2)gl, Fur1, Dr, Grip, Drl-2, csw, ex, ush, beat-Ic, Poxm, Mbs, RasGAP1, spz3, Antp, ko, beat-Va, CtBP, tara, stan, ckn, futsch, beat-VI, ced-6, IP3K2, wake, gukh, cora, sff, ken, zen, beat-IIa, Dhc64C, kibra, al, EcR, Rok, Ten-m, e(y)3, Nrx-1, nej, Mmp1, ey, Pura, step, dlg1, par-1, spri, heph, siz, Hipk, slo, Abl, CG8405, hbs, Lim3, Unc-115a, cac, Dad, cic, Patronin, crol, sd, RhoGEF64C, en, ths, fs(1)h, sfl, beat-IIIb, Rbfox1, rl, kirre, vn, cher, Tl, Fas3, CG5758, pyd, unk
nervous system development	178 of 856 genes, 20.8%	1205 of 16085 genes, 7.5%	1.35e-34	0.00%	0.00	fz2, sbb, Sh, Npc1b, Nlg1, kuz, jing, lilli, CG5921, osa, l(3)72Ab, Trim9, Gnf1, Nlg3, crb, robo3, pyr, hid, if, inv, Ten-a, cno, Ptp99A, lab, poe, ap, Awh, bdg, cv-c, Snoo, DIP-gamma, rut, Nrg, pum, Mmp2, amon, trol, alph, msi, Src42A, beat-IIb, beat-Ib, stau, CG13251, ds, dysc, hth, mtg, ft, qua, Doa, sas, ara, Ser, FER, S, mam, Ptp61F, shep, PsGEF, shn, foxo, DAAM, grn, neur, mew, beat-Vc, Prosap, app, Pka-R2, bun, Tao, ed, CadN2, opa, kay, InR, pdm3, f, tinc, PDZ-GEF, nerfin-1, wnd, Stat92E, Src64B, CG6701, dsx, Sema2a, chinmo, Bsg, cdi, klu, loco, elB, fra, Lim1, nkd, lola, boss, Sema5c, vvl, Fife, abd-A, dpr12, mbl, stl, stai, CalpA, egh, pnt, Gie, melt, jbug, lmd, esn, hh, drl, pot, l(2)gl, Fur1, Dr, Drl-2, csw, ex, beat-Ic, Poxm, Mbs, RasGAP1, spz3, Antp, ko, beat-Va, stan, ckn, futsch, beat-VI, ced-6, wake, gukh, cora, sff, beat-IIa, Dhc64C, kibra, EcR, Rok, Ten-m, Nrx-1, nej, Mmp1, ey, Pura, dlg1, par-1, spri, siz, slo, Abl, hbs, Lim3, Unc-115a, cac, Dad, Patronin, sd, RhoGEF64C, en, fs(1)h, ths, beat-IIIb, Rbfox1, rl, vn, cher, Tl, Fas3, CG5758, unk
signaling	217 of 856 genes, 25.4%	1694 of 16085 genes, 10.5%	1.13e-33	0.00%	0.00	fz2, Sh, DCX-EMAP, dome, CG31183, Sesn, CG34393, Nlg1, kuz, Camta, CG42674, nAChRalpha2, rdgA, Sxl, osa, Dys, Cnx99A, Trim9, TyrR, Nlg3, Ggamma30A, crb, pyr, hid, Ten-a, Sytbeta, cno, GluRIB, Usp10, dtr, C3G, Oct-TyrR, Tlk, Gprk2, sra, CG7094, Eip75B, cv-c, Snoo, rut, CG42684, pum, Pka-C3, unc-13-4A, uif, CG34384, Dh31-R, Mmp2, trol, ETHR, CG32447, alph, Ac78C, CG30456, Src42A, S6KL, Snap25, ds, dysc, kek6, CG43658, ft, GluRIA, nAChRalpha6, Doa, pHCl-1, Ser, FER, S, mam, Ptp61F, nemy, CG33639, PsGEF, CCHa1-R, shn, foxo, Mctp, neur, Prosap, Pka-R2, Tao, ed, wry, kay, InR, nAChRbeta2, hppy, sima, mAChR-B, Cdep, Syt7, CG12344, PDZ-GEF, CG31760, wnd, RhoBTB, Stat92E, 5-HT7, Src64B, Btk29A, Sema2a, cdi, SCAP, loco, pip, Cngl, fra, nkd, Parp, lola, Smr, boss, Gyc88E, Fs, Fife, PVRAP, nau, Duox, Dgk, Grd, Gie, pnt, melt, LRR, tefu, rdgC, hang, Pde6, ltl, smog, cv-2, Sap47, CG32758, Debcl, hh, drl, Pde1c, l(2)gl, wdb, Grip, Drl-2, csw, ex, Rph, RasGAP1, X11Lbeta, spz3, CG15611, sNPF-R, CtBP, axo, RhoGAP18B, stan, ckn, cta, trp, Cbp53E, wake, sff, ken, kek5, Hr4, CG14669, BicD, kibra, EcR, Tie, Rok, CG34357, Ten-m, Nrx-1, nej, ACXC, chrb, Ca-alpha1T, Shab, ey, Pura, CG4629, dlg1, Oamb, step, par-1, spri, siz, TrissinR,

						Proc-R, Hipk, slo, KaiR1D, Abl, hbs, Rh7, cac, Dad, cic, CCKLR-17D1, Pde8, lncRNA:acal, crol, sd, RhoGEF64C, Ac3, Gr28b, ths, sfl, Dop1R2, Syt1, rl, vn, Pde11, CG32683, Tl, ogre, pyd, Gprk1
neuron differentiation	141 of 856 genes, 16.5%	823 of 16085 genes, 5.1%	1.61e-33	0.00%	0.00	fz2, sbb, Sh, kuz, jing, lilli, CG5921, osa, Trim9, Gnf1, crb, robo3, hid, if, inv, Ten-a, Ptp99A, ap, Awh, bdg, cv-c, Snoo, rut, DIP-gamma, Nrg, pum, Mmp2, amon, trol, alph, msi, Src42A, beat-IIb, beat-Ib, CG13251, ds, dysc, hth, ft, qua, Doa, sas, ara, FER, S, mam, Ptp61F, shep, shn, foxo, DAAM, grn, neur, mew, beat-Vc, Prosap, app, Pka-R2, bun, ed, CadN2, pdm3, InR, kay, f, tinc, PDZ-GEF, nerfin-1, wnd, Src64B, CG6701, dsx, Sema2a, chinmo, Bsg, cdi, klu, elB, fra, Lim1, lola, boss, vvl, dpr12, mbl, CalpA, stai, egh, pnt, melt, jbug, esn, hh, drl, pot, Fur1, csw, Drl-2, ex, beat-Ic, Poxm, Mbs, RasGAP1, ko, beat-Va, stan, ckn, futsch, ced-6, beat-VI, gukh, sff, cora, beat-IIa, Dhc64C, EcR, Rok, Ten-m, nej, Mmp1, ey, Pura, par-1, spri, Abl, hbs, Unc-115a, Lim3, Dad, Patronin, cac, RhoGEF64C, fs(1)h, en, beat-IIIb, rl, cher, Tl, Fas3, CG5758, unk
cell communication	220 of 856 genes, 25.7%	1740 of 16085 genes, 10.8%	2.38e-33	0.00%	0.00	fz2, sbb, Sh, DCX-EMAP, dome, CG31183, Sesn, CG34393, Nlg1, kuz, Camta, CG42674, nAChRalpha2, rdgA, Sxl, osa, Dys, Cnx99A, Trim9, TyrR, Nlg3, Ggamma30A, crb, pyr, hid, Ten-a, Sytbeta, cno, GluRIB, Usp10, dtr, C3G, Oct-TyrR, lt, Tlk, Gprk2, sra, CG7094, Eip75B, cv-c, Snoo, rut, CG42684, pum, Pka-C3, unc-13-4A, uif, CG34384, Dh31-R, Mmp2, trol, ETHR, CG32447, alph, Ac78C, CG30456, Src42A, S6KL, Snap25, ds, dysc, kek6, CG43658, ft, GluRIA, nAChRalpha6, Doa, pHCl-1, Ser, FER, S, mam, Ptp61F, nemy, CG33639, PsGEF, CCHa1-R, shn, foxo, Mctp, neur, Prosap, Pka-R2, Tao, ed, wry, kay, InR, pdm3, nAChRbeta2, hppy, sima, mAChR-B, Cdep, Syt7, CG12344, PDZ-GEF, CG31760, wnd, RhoBTB, Stat92E, 5-HT7, Src64B, Btk29A, Sema2a, cdi, SCAP, loco, pip, Cngl, fra, nkd, Parp, lola, Smr, boss, Gyc88E, Fs, Fife, PVRAP, nau, Duox, Dgk, Grd, Gie, pnt, melt, tefu, LRR, rdgC, hang, Pde6, Itl, smog, cv-2, Sap47, CG32758, Debcl, hh, drl, Pde1c, l(2)gl, wdb, Grip, Drl-2, csw, ex, Rph, RasGAP1, X11Lbeta, spz3, CG15611, sNPF-R, CtBP, axo, RhoGAP18B, stan, ckn, cta, trp, Cbp53E, wake, sff, ken, kek5, Hr4, CG14669, BicD, kibra, EcR, Tie, Rok, CG34357, Ten-m, Nrx-1, nej, ACXC, chrb, Ca-alpha1T, Shab, ey, Pura, CG4629, dlg1, Oamb, step, par-1, spri, siz, TrissinR, Proc-R, Hipk, slo, KaiR1D, Abl, hbs, Rh7, cac, Dad, cic, CCKLR-17D1, Pde8, lncRNA:acal, crol, sd, RhoGEF64C, Ac3, Gr28b, ths, sfl, Dop1R2, Syt1, rl, vn, Pde11, CG32683, Tl, ogre, pyd, Gprk1
regulation of cellular process	351 of 856 genes, 41.0%	3609 of 16085 genes, 22.4%	1.18e-32	0.00%	0.00	fz2, Sh, CG10185, Nlg1, lilli, nAChRalpha2, rdgA, tna, Sxl, osa, l(3)72Ab, Atf6, Ggamma30A, CG12054, hid, if, Ten-a, Sytbeta, GluRIB, cno, lab, Hcf, dsf, scrt, C3G, Oct-TyrR, lt, sra, CG7094, Eip75B, cv-c, Snoo, hng3, DIP-gamma, rut, CG8312, pum, Pka-C3, uif, mamo, Dh31-R, Mmp2, Blimp-1, trol, ETHR, stv, alph, Ac78C, msi, Src42A, Eip78C, Snap25, ds, dysc, hth, kek6,

						GluRIA, Doa, sas, FER, S, Ptp61F, nemy, PsGEF, CCHa1-R, Mctp, CG5694, Hk, DAAM, neur, per, CG3726, Prosap, Tao, ed, opa, InR, nAChRbeta2, sima, Rx, CG12769, RyR, wnd, bin3, RhoBTB, Stat92E, Src64B, Btk29A, cdi, loco, fra, tral, MBD-R2, Smr, Gyc88E, Fs, Fife, mbl, Duox, stl, MED14, bab2, Grd, melt, rdgC, hang, Pde6, smog, cv-2, Sap47, CG32758, lmd, ich, disco-r, Pde1c, Oaz, l(2)gl, bol, Dr, ex, Poxm, Mbs, Antp, CG15611, Xrp1, htt, Men, bru3, CG1815, trp, futsch, Cbp53E, CG11486, wake, Tet, RunxB, Jupiter, spir, sff, CG14669, Wdr62, BicD, EcR, Ten-m, e(y)3, Nrx-1, upSET, Shab, ey, Pura, step, dlg1, Oamb, par-1, spri, heph, TrissinR, Proc-R, Hipk, slo, Abl, ps, Lim3, gpp, cac, Pde8, CCKLR-17D1, Dad, cic, lncRNA:acal, crol, sd, RhoGEF64C, RecQ4, ths, Gr28b, Gyf, fs(1)h, Ac3, Rbfox1, Syt1, rl, kirre, CG32683, cher, Tl, Gbs-70E, unk, sbb, DCX-EMAP, dome, CG31183, ec, CG34393, Sesn, jing, kuz, caup, CG42674, Camta, dpy, eIF4EHP, luna, CG33144, Dys, Trim9, Cnx99A, CG12605, TyrR, Nlg3, crb, AGO3, pyr, inv, Mef2, CG10947, Usp10, ap, Gprk2, Tlk, Awh, Spt3, Sox21a, Kank, Fancm, crp, CG42684, CG34384, CG32447, CG30456, stau, S6KL, timeout, Tis11, CG43658, CG40178, ft, nAChRalpha6, pHCl-1, ara, Ser, mam, CG33639, foxo, shn, grn, CG17514, Pka-R2, bun, Spt20, loh, pdm3, kay, wry, hppy, tinc, mAChR-B, Cdep, CG11247, Syt7, CG12344, PDZ-GEF, nerfin-1, l(3)psg2, CG31760, 5-HT7, dsx, Sema2a, chinmo, klu, SCAP, pip, Cngl, Lim1, nkd, Parp, lola, boss, TfAP-2, vvl, abd-A, PVRAP, nau, Dgk, Eip93F, stai, Rev1, pnt, Ccn, LRR, tefu, corto, ltl, rhea, dia, Debcl, hh, drl, wdb, Drl-2, csw, ush, RasGAP1, Rme-8, spz3, ko, sNPF-R, CtBP, tyn, tara, stan, RhoGAP18B, ckn, cta, CG4238, ken, zen, e, kek5, Hr4, Dhc64C, kibra, al, Tie, Rok, CG34357, nej, ACXC, chrb, Maf1, CG31612, CG4629, smg, siz, KaiR1D, CG8405, hbs, Rh7, Trxr-2, Patronin, bru2, en, sfl, Dop1R2, CG9932, vn, Pde11, tut, ogre, pyd, Gprk1
biological regulation	402 of 856 genes, 47.0%	4421 of 16085 genes, 27.5%	1.63e-32	0.00%	0.00	fz2, Sh, CG10185, Nlg1, Fs(2)Ket, lilli, nAChRalpha2, rdgA, tna, Sxl, osa, l(3)72Ab, Atf6, Ggamma30A, CG12054, hid, if, Ten-a, Sytbeta, GluRIB, cno, lab, Hcf, dsf, scrt, C3G, Oct-TyrR, lt, sra, nw, CG7094, Eip75B, cv-c, nvd, Snoo, hng3, DIP-gamma, rut, CG8312, pum, Pka-C3, uif, mamo, Dh31-R, amon, Mmp2, Blimp-1, trol, ETHR, stv, alph, Ac78C, msi, Src42A, Eip78C, Snap25, CG1090, ds, dysc, hth, kek6, GluRIA, Doa, CPT2, sas, FER, S, Ptp61F, nemy, PsGEF, CCHa1-R, Mctp, CG5694, Hk, DAAM, Scgdelta, neur, per, CG3726, Prosap, Tao, ed, opa, InR, nAChRbeta2, Mvl, sima, Rx, CG12769, RyR, wnd, bin3, RhoBTB, Stat92E, Src64B, Btk29A, CG6701, cdi, loco, fra, tral, MBD-R2, Smr, Gyc88E, NKCC, Fs, Fife, mbl, Duox, stl, MED14, bab2, Grd, egh, Gie, melt, Elk, rdgC, hang, Pde6, smog, cv-2, Msp300, Sap47, CG32758, lmd, ich, disco-r, Pde1c, Oaz, l(2)gl, bol, Dr, ex, Poxm, Rph, Mbs, Antp, Xrp1, CG15611, htt, Men, bru3, CG1815, trp, futsch, Cbp53E, CG11486, Tet, wake,

						RunxB, Jupiter, cora, spir, sff, CG14669, Wdr62, BicD, EcR, Ten-m, e(y)3, Nrx-1, CG42795, upSET, Ca-alpha1T, Mmp1, Shab, ey, Pura, step, dlg1, Oamb, SK, Drip, par-1, spri, heph, TrissinR, Proc-R, Hipk, slo, Abl, ps, Lim3, gpp, cac, Pde8, CCKLR-17D1, Dad, cic, lncRNA:acal, crol, sd, RhoGEF64C, RecQ4, ths, Gr28b, Gyf, fs(1)h, Ac3, Rbfox1, Syt1, rl, kirre, CG32683, Orco, olf413, cher, Tl, Gbs-70E, unk, sbb, DCX-EMAP, dome, CG31183, ec, CG34393, Sesn, jing, kuz, caup, CG8177, CG42674, Vps13D, Camta, dpy, eIF4EHP, luna, CG33144, Dys, alpha-Cat, Cnx99A, Trim9, CG12605, TyrR, Nlg3, crb, AGO3, pyr, Mef2, inv, CG10947, Usp10, ap, Tlk, Gprk2, Awh, Spt3, sano, Sox21a, Kank, Fancm, CG33298, Nrg, crp, CG42684, unc-13-4A, CG34384, CG32447, CG30456, stau, S6KL, timeout, Tis11, Ncc69, CG43658, CG40178, ft, nAChRalpha6, nrv1, pHCl-1, ara, Ser, mam, CG33639, foxo, shn, CG2121, Tob, grn, mew, bgm, CG17514, Pka-R2, bun, Spt20, loh, pdm3, kay, wry, hppy, Apoltp, tinc, mAChR-B, Cdep, CG11247, Syt7, Trpm, CG12344, PDZ-GEF, Achl, nerfin-1, l(3)psg2, CG31760, CG30377, 5-HT7, dsx, Sema2a, chinmo, klu, SCAP, elB, pip, Cngl, Lim1, nkd, Parp, lola, Sara, boss, TfAP-2, vvl, abd-A, PVRAP, nau, Dgk, Eip93F, stai, PyK, Rev1, pnt, Ccn, LRR, tefu, Hs6st, corto, ltl, rhea, dia, Debcl, hh, drl, wdb, Fur1, KCNQ, Drl-2, csw, ush, RasGAP1, Rme-8, spz3, ko, sNPF-R, CtBP, tyn, tara, RhoGAP18B, stan, ckn, cta, CG4238, ken, zen, e, kek5, Hr4, Dhc64C, kibra, al, Tie, Rok, CG34357, nej, ACXC, ATP8B, chrb, Maf1, CG31612, CG4629, smg, siz, KaiR1D, CG8405, hbs, Rh7, Trxr-2, Patronin, CG4744, bru2, en, sfl, Dop1R2, CG9932, Ttd14, vn, tut, Pde11, SerT, unc80, ogre, pyd, Gprk1
regulation of biological process	368 of 856 genes, 43.0%	3934 of 16085 genes, 24.5%	4.21e-31	0.00%	0.00	fz2, Sh, CG10185, Nlg1, Fs(2)Ket, lilli, nAChRalpha2, rdgA, tna, Sxl, osa, l(3)72Ab, Atf6, Ggamma30A, CG12054, hid, if, Ten-a, Sytbeta, GluRIB, cno, lab, Hcf, dsf, scrt, C3G, Oct-TyrR, lt, sra, CG7094, Eip75B, cv-c, Snoo, hng3, DIP-gamma, rut, CG8312, pum, Pka-C3, uif, mamo, Dh31-R, Mmp2, Blimp-1, trol, ETHR, stv, alph, Ac78C, msi, Src42A, Eip78C, Snap25, ds, dysc, hth, kek6, GluRIA, Doa, sas, FER, S, Ptp61F, nemy, PsGEF, CCHa1-R, Mctp, CG5694, Hk, DAAM, neur, per, CG3726, Prosap, Tao, ed, opa, InR, nAChRbeta2, Mvl, sima, Rx, CG12769, RyR, wnd, bin3, RhoBTB, Stat92E, Src64B, Btk29A, CG6701, cdi, loco, fra, tral, MBD-R2, Smr, Gyc88E, Fs, Fife, mbl, Duox, stl, MED14, bab2, Grd, egh, melt, rdgC, hang, Pde6, smog, cv-2, Sap47, CG32758, lmd, ich, disco-r, Pde1c, Oaz, l(2)gl, bol, Dr, ex, Poxm, Mbs, Antp, Xrp1, CG15611, htt, Men, bru3, CG1815, trp, futsch, Cbp53E, CG11486, Tet, wake, RunxB, Jupiter, spir, sff, CG14669, Wdr62, BicD, EcR, Ten-m, e(y)3, Nrx-1, upSET, Shab, ey, Pura, step, dlg1, Oamb, Drip, par-1, spri, heph, TrissinR, Proc-R, Hipk, slo, Abl, ps, Lim3, gpp, cac, Pde8, CCKLR-17D1, Dad, cic, lncRNA:acal, crol, sd, RhoGEF64C, RecQ4, ths, Gr28b, Gyf, fs(1)h, Ac3, Rbfox1, Syt1, rl, kirre,

						CG32683, Orco, cher, Tl, Gbs-70E, unk, sbb, DCX-EMAP, dome, CG31183, ec, CG34393, Sesn, jing, kuz, caup, CG42674, Camta, dpy, eIF4EHP, luna, CG33144, Dys, Trim9, Cnx99A, CG12605, TyrR, Nlg3, crb, AGO3, pyr, inv, Mef2, CG10947, Usp10, ap, Gprk2, Tlk, Awh, Spt3, Sox21a, Kank, Fancm, crp, CG42684, CG34384, CG32447, CG30456, stau, S6KL, timeout, Tis11, CG43658, CG40178, ft, nAChRalpha6, pHCl-1, ara, Ser, mam, CG33639, foxo, shn, CG2121, Tob, grn, bgm, CG17514, Pka-R2, bun, Spt20, loh, pdm3, kay, wry, hppy, Apoltp, tinc, mAChR-B, Cdep, CG11247, Syt7, CG12344, PDZ-GEF, Achl, nerfin-1, l(3)psg2, CG31760, 5-HT7, dsx, Sema2a, chinmo, klu, SCAP, elB, pip, Cngl, Lim1, nkd, Parp, lola, boss, TfAP-2, vvl, abd-A, PVRAP, nau, Dgk, Eip93F, stai, Rev1, pnt, Ccn, LRR, tefu, Hs6st, corto, Itl, rhea, dia, Debcl, hh, drl, wdb, KCNQ, Drl-2, csw, ush, RasGAP1, Rme-8, spz3, ko, sNPF-R, CtBP, tyn, tara, stan, RhoGAP18B, ckn, cta, CG4238, ken, zen, e, kek5, Hr4, Dhc64C, kibra, al, Tie, Rok, CG34357, nej, ACXC, ATP8B, chrb, Maf1, CG31612, CG4629, smg, siz, KaiR1D, CG8405, hbs, Rh7, Trxr-2, Patronin, CG4744, bru2, en, sfl, Dop1R2, CG9932, Ttd14, vn, tut, Pde11, ogre, pyd, Gprk1
cell differentiation	226 of 856 genes, 26.4%	1890 of 16085 genes, 11.8%	1.24e-30	0.00%	0.00	fz2, sbb, Sh, dome, ec, kuz, jing, caup, Fs(2)Ket, lilli, CG5921, Sxl, osa, alpha-Cat, Trim9, Gnf1, crb, robo3, pyr, sns, hid, if, Mef2, inv, Ten-a, cno, Ptp99A, lab, poe, dsf, ap, C3G, jvl, Gprk2, Awh, bdg, Sox21a, Eip75B, cv-c, Snoo, DIP-gamma, rut, Nrg, pum, uif, Mmp2, amon, trol, Blimp-1, alph, msi, Src42A, beat-IIb, beat-Ib, stau, CG13251, bbg, ds, dysc, hth, ft, qua, Doa, sas, ara, Ser, FER, S, mam, Ptp61F, shep, shn, foxo, DAAM, grn, Scgdelta, neur, toc, mew, beat-Vc, Prosap, app, Pka-R2, bun, Tao, ed, CadN2, kay, InR, pdm3, sima, f, tinc, PDZ-GEF, l(3)psg2, nerfin-1, wnd, Stat92E, Src64B, Btk29A, CG6701, dsx, Sema2a, chinmo, Bsg, cdi, klu, loco, elB, fra, Lim1, nkd, Parp, lola, Sara, Smr, boss, vvl, Fs, abd-A, dpr12, nau, mbl, stl, bab2, milt, stai, CalpA, PyK, egh, pnt, melt, jbug, tefu, smog, lncRNA:flam, Msp300, rost, CG41099, Rbp6, rhea, mei-P26, dia, lmd, esn, disco-r, Debcl, Rbp9, hh, drl, pot, l(2)gl, bol, Fur1, Dr, Drl-2, csw, ex, ush, beat-Ic, Poxm, Mbs, RasGAP1, Rme-8, Antp, ko, beat-Va, CtBP, tyn, stan, ckn, cta, futsch, beat-VI, ced-6, IP3K2, wake, Tet, gukh, cora, spir, sff, beat-IIa, BicD, Dhc64C, kibra, EcR, Tie, Rok, Ten-m, nej, Mmp1, ey, Pura, dlg1, par-1, spri, heph, siz, Abl, hbs, Lim3, Unc-115a, cac, cic, Dad, Patronin, sd, RhoGEF64C, RecQ4, en, ths, fs(1)h, beat-IIIb, Rbfox1, Syt1, kirre, rl, vn, tut, cher, Tl, Fas3, CG5758, pyd, unk
multicellular organism development	286 of 856 genes, 33.4%	2715 of 16085 genes, 16.9%	1.25e-30	0.00%	0.00	fz2, Sh, Nlg1, Cpr50Cb, lilli, CG5921, Sxl, CLIP-190, osa, l(3)72Ab, sns, hid, Ccp84Ac, if, Ten-a, cno, Ptp99A, lab, poe, dsf, C3G, lt, nw, Eip75B, cv-c, nvd, Snoo, DIP-gamma, rut, pum, Pka-C3, uif, Mmp2, amon, Blimp-1, trol, alph, msi, Src42A, ds, dysc, hth, Doa, sas, FER, S, Ptp61F, PsGEF, DAAM, Scgdelta, neur, beat-Vc, per, Sox100B, Prosap, Tao, ed, opa, InR,

						sima, f, Tsp, wnd, Cht7, Stat92E, Src64B, Btk29A, CG6701, cdi, frm, loco, fra, tral, Smr, Sema5c, Fs, Fife, dpr12, mbl, Duox, stl, bab2, egh, Gie, melt, jbug, smog, cv-2, Msp300, T48, CG41099, lmd, ich, esn, disco-r, pot, Oaz, l(2)gl, Dr, ex, beat-Ic, Poxm, Mbs, Antp, Men, futsch, beat-VI, wake, gukh, cora, spir, sff, beat-IIa, BicD, EcR, Ten-m, e(y)3, Nrx-1, Mmp1, ey, Pura, step, dlg1, par-1, spri, heph, Hipk, slo, Abl, Lim3, Unc-115a, cac, Dad, cic, lncRNA:acal, crol, sd, RhoGEF64C, RecQ4, ths, fs(1)h, Rbfox1, Syt1, rl, kirre, cher, Tl, unk, sbb, Npc1b, dome, ec, Sesn, jing, kuz, caup, CG42674, dpy, eIF4EHP, luna, Dys, alpha-Cat, Trim9, Gnf1, Nlg3, crb, pwn, pyr, robo3, inv, Mef2, ap, jvl, Gprk2, Tlk, Awh, bdg, sano, alpha-Man-Ia, Sox21a, Nrg, crp, CG30456, CG13251, stau, beat-Ib, beat-IIb, CG43658, ft, mtg, qua, ara, Ser, mam, shep, foxo, shn, grn, mew, toc, Pka-R2, app, bun, CadN2, loh, pdm3, kay, tinc, PDZ-GEF, nerfin-1, l(3)psg2, dsx, chinmo, Sema2a, Bsg, klu, elB, pip, Lim1, nkd, Parp, lola, boss, vkg, TfAP-2, vvl, abd-A, nau, milt, CalpA, stai, CG5890, pnt, Hs6st, corto, ltl, Sb, rhea, dia, AdamTS-A, hh, drl, wdb, KCNQ, Grip, Fur1, csw, Drl-2, ush, RasGAP1, Rme-8, spz3, ko, CtBP, beat-Va, tara, tyn, stan, cta, ckn, ced-6, IP3K2, zen, e, ken, Hr4, kibra, Dhc64C, Tie, al, Rok, nej, chrb, siz, CG42663, CG8405, hbs, Patronin, en, beat-IIIb, sfl, vn, TwdlQ, Fas3, CG5758, pyd
cellular developmental process	228 of 856 genes, 26.6%	1938 of 16085 genes, 12.0%	7.22e-30	0.00%	0.00	fz2, sbb, Sh, dome, ec, kuz, jing, caup, Fs(2)Ket, lilli, CG5921, Sxl, osa, alpha-Cat, Trim9, Gnf1, crb, robo3, pyr, sns, hid, if, Mef2, inv, Ten-a, cno, Ptp99A, lab, poe, dsf, ap, C3G, jvl, Gprk2, Awh, bdg, Sox21a, Eip75B, cv-c, Snoo, DIP-gamma, rut, Nrg, pum, uif, Mmp2, amon, Blimp-1, trol, alph, msi, Src42A, beat-IIb, beat-Ib, stau, CG13251, bbg, ds, dysc, hth, ft, qua, Doa, sas, ara, Ser, FER, S, mam, Ptp61F, shep, shn, foxo, DAAM, grn, Scgdelta, neur, toc, mew, beat-Vc, Prosap, app, Pka-R2, bun, Tao, ed, CadN2, kay, InR, pdm3, sima, f, tinc, PDZ-GEF, l(3)psg2, nerfin-1, wnd, RhoBTB, Stat92E, Src64B, Btk29A, CG6701, dsx, Sema2a, chinmo, Bsg, cdi, klu, loco, elB, fra, Lim1, nkd, Parp, lola, Sara, Smr, boss, vvl, Fs, abd-A, dpr12, nau, mbl, stl, bab2, milt, stai, CalpA, PyK, egh, pnt, melt, jbug, tefu, smog, lncRNA:flam, Msp300, rost, CG41099, Rbp6, rhea, mei-P26, dia, lmd, esn, disco-r, Debcl, Rbp9, hh, drl, pot, l(2)gl, bol, Fur1, Dr, Drl-2, csw, ex, ush, beat-Ic, Poxm, Mbs, RasGAP1, Rme-8, Antp, ko, beat-Va, CtBP, tyn, stan, ckn, cta, futsch, beat-VI, ced-6, IP3K2, wake, Tet, gukh, cora, spir, sff, beat-IIa, BicD, Dhc64C, kibra, EcR, Tie, Rok, Ten-m, nej, ACC, Mmp1, ey, Pura, dlg1, par-1, spri, heph, siz, Abl, hbs, Lim3, Unc-115a, cac, cic, Dad, Patronin, sd, RhoGEF64C, RecQ4, en, ths, fs(1)h, beat-IIIb, Rbfox1, Syt1, kirre, rl, vn, tut, cher, Tl, Fas3, CG5758, pyd, unk
animal organ development	183 of 856 genes, 21.4%	1379 of 16085 genes, 8.6%	2.21e-29	0.00%	0.00	fz2, sbb, dome, ec, kuz, jing, caup, lilli, CG42674, dpy, CG5921, Sxl, osa, l(3)72Ab, Dys, Trim9, pwn, crb, robo3, pyr, sns, hid, if, Mef2, inv, Ten-a, cno, lab, ap, C3G, Gprk2, Awh, bdg, nw, sano, cv-c,

						Nrg, crp, Pka-C3, uif, Mmp2, amon, trol, alph, msi, CG30456, Src42A, CG13251, ds, dysc, hth, CG43658, ft, qua, Doa, ara, Ser, S, mam, PsGEF, shn, foxo, DAAM, grn, Scgdelta, neur, mew, Sox100B, Prosap, app, bun, Tao, ed, CadN2, loh, opa, kay, InR, sima, f, tinc, Tsp, PDZ-GEF, l(3)psg2, Stat92E, Src64B, Btk29A, dsx, Sema2a, chinmo, cdi, klu, loco, elB, fra, Lim1, lola, Smr, vkg, boss, Sema5c, TfAP-2, vvl, abd-A, mbl, nau, Duox, stl, bab2, CG5890, pnt, melt, Hs6st, corto, ltl, cv-2, Msp300, Sb, rhea, dia, lmd, ich, AdamTS-A, disco-r, hh, drl, pot, l(2)gl, Grip, Dr, Drl-2, csw, ex, ush, Poxm, Mbs, RasGAP1, Antp, CtBP, tara, stan, futsch, ced-6, IP3K2, cora, ken, zen, Dhc64C, kibra, al, EcR, Rok, Ten-m, e(y)3, nej, Mmp1, ey, Pura, dlg1, step, par-1, heph, siz, Hipk, CG8405, Abl, hbs, cic, Dad, crol, sd, RhoGEF64C, en, ths, sfl, Rbfox1, kirre, rl, vn, cher, Tl, pyd, unk
tissue development	164 of 856 genes, 19.2%	1186 of 16085 genes, 7.4%	6.62e-28	0.00%	0.00	sbb, Npc1b, dome, ec, kuz, jing, caup, Fs(2)Ket, lilli, CG42674, dpy, Sxl, osa, alpha-Cat, Dys, crb, pyr, hid, if, Mef2, inv, cno, ap, jvl, Gprk2, Awh, bdg, nw, sano, Sox21a, cv-c, Nrg, Pka-C3, rgn, Mmp2, trol, alph, CG30456, Src42A, bbg, ds, hth, CG43658, ft, qua, ara, Ser, FER, S, mam, shn, grn, Scgdelta, neur, toc, mew, app, bun, Tao, ed, CadN2, opa, InR, kay, f, PDZ-GEF, l(3)psg2, Stat92E, Src64B, Btk29A, dsx, Sema2a, chinmo, loco, elB, Lim1, Parp, lola, vkg, Smr, TfAP-2, abd-A, nau, Duox, stl, bab2, stai, CG5890, egh, pnt, jbug, Hs6st, corto, smog, ltl, cv-2, lncRNA:flam, Msp300, Sb, CG41099, rhea, dia, lmd, disco-r, hh, drl, pot, l(2)gl, Dr, csw, ex, ush, Mbs, RasGAP1, Rme-8, CtBP, tyn, tara, stan, cta, cora, spir, ken, kibra, Dhc64C, al, Tie, EcR, Rok, e(y)3, nej, Mmp1, ey, Pura, dlg1, step, par-1, CG43897, spri, heph, Hipk, CG8405, Abl, hbs, chas, cic, Dad, Pde8, lncRNA:acal, crol, sd, RhoGEF64C, RecQ4, en, ths, sfl, Rbfox1, kirre, rl, vn, cher, Fas3, pyd, unk
animal organ morphogenesis	134 of 856 genes, 15.7%	849 of 16085 genes, 5.3%	8.22e-28	0.00%	0.00	fz2, sbb, dome, ec, kuz, jing, caup, lilli, CG42674, dpy, CG5921, osa, Dys, pwn, crb, sns, hid, if, inv, cno, ap, Gprk2, bdg, nw, sano, cv-c, Nrg, Pka-C3, uif, Mmp2, amon, trol, alph, msi, CG30456, Src42A, CG13251, ds, dysc, hth, CG43658, ft, qua, Doa, ara, Ser, S, mam, shn, foxo, grn, neur, mew, Prosap, app, bun, ed, CadN2, opa, kay, f, tinc, PDZ-GEF, l(3)psg2, Stat92E, Src64B, Btk29A, cdi, klu, elB, fra, Lim1, lola, boss, TfAP-2, mbl, Duox, bab2, pnt, melt, Hs6st, corto, ltl, cv-2, rhea, dia, AdamTS-A, ich, disco-r, hh, pot, drl, l(2)gl, Grip, Dr, csw, Drl-2, ex, Mbs, RasGAP1, CtBP, stan, IP3K2, cora, kibra, EcR, al, Rok, Ten-m, e(y)3, nej, Mmp1, ey, Pura, dlg1, step, par-1, heph, siz, Hipk, hbs, cic, Dad, sd, crol, RhoGEF64C, en, sfl, kirre, rl, Rbfox1, vn, pyd, unk
signal transduction	183 of 856 genes, 21.4%	1445 of 16085 genes, 9.0%	9.75e-27	0.00%	0.00	fz2, DCX-EMAP, dome, CG31183, Sesn, CG34393, kuz, Camta, CG42674, nAChRalpha2, rdgA, Sxl, osa, Cnx99A, Trim9, TyrR, Ggamma30A, crb, pyr, hid, cno, Usp10, C3G, Oct-TyrR, Tlk, Gprk2, sra, CG7094, Eip75B, cv-c, Snoo, rut, CG42684, pum, Pka-C3, uif, CG34384, Dh31-R, Mmp2, trol, ETHR, CG32447,

						alph, Ac78C, CG30456, Src42A, S6KL, ds, CG43658, ft, nAChRalpha6, Doa, pHCl-1, Ser, FER, S, mam, Ptp61F, CG33639, PsGEF, CCHa1-R, shn, foxo, neur, Prosap, Pka-R2, Tao, ed, wry, InR, kay, nAChRbeta2, hppy, sima, mAChR-B, Cdep, CG12344, PDZ-GEF, CG31760, wnd, RhoBTB, Stat92E, 5-HT7, Src64B, Btk29A, Sema2a, cdi, SCAP, loco, pip, Cngl, fra, nkd, Parp, lola, Smr, boss, Gyc88E, Fs, PVRAP, Duox, Dgk, Grd, pnt, melt, LRR, tefu, rdgC, hang, Pde6, ltl, smog, cv-2, CG32758, Debcl, hh, drl, Pde1c, l(2)gl, wdb, Drl-2, csw, ex, RasGAP1, spz3, CG15611, sNPF-R, CtBP, stan, RhoGAP18B, ckn, cta, trp, wake, sff, ken, kek5, Hr4, CG14669, kibra, Tie, EcR, Rok, CG34357, nej, ACXC, chrb, ey, Pura, CG4629, dlg1, Oamb, step, par-1, spri, siz, TrissinR, Proc-R, Hipk, hbs, Rh7, cac, cic, Dad, CCKLR-17D1, Pde8, lncRNA:acal, crol, sd, RhoGEF64C, Ac3, Gr28b, ths, sfl, Dop1R2, rl, vn, Pde11, CG32683, Tl, ogre, pyd, Gprk1
developmental process	313 of 856 genes, 36.6%	3278 of 16085 genes, 20.4%	3.32e-26	0.00%	0.00	fz2, Sh, Nlg1, Cpr50Cb, Fs(2)Ket, lilli, CG5921, Sxl, CLIP-190, osa, l(3)72Ab, sns, hid, Ccp84Ac, if, Ten-a, cno, Ptp99A, lab, poe, dsf, C3G, lt, nw, Eip75B, cv-c, nvd, Snoo, DIP-gamma, rut, pum, Pka-C3, uif, rgn, amon, Mmp2, Blimp-1, trol, alph, msi, Src42A, bbg, ds, dysc, hth, Doa, sas, FER, S, Ptp61F, PsGEF, DAAM, Scgdelta, neur, beat-Vc, per, Sox100B, Prosap, Tao, DNApol-epsilon255, ed, opa, InR, sima, f, Tif-IA, Tsp, RyR, wnd, bin3, RhoBTB, Cht7, Stat92E, Src64B, Btk29A, CG6701, cdi, frm, loco, fra, tral, Smr, Sema5c, Fs, Fife, dpr12, mbl, Duox, stl, bab2, egh, Gie, melt, jbug, smog, cv-2, lncRNA:flam, Msp300, T48, CG41099, Imd, ich, esn, disco-r, pot, Oaz, l(2)gl, bol, Dr, ex, beat-Ic, Poxm, Mbs, Antp, Men, futsch, beat-VI, wake, Tet, gukh, cora, spir, sff, beat-IIa, BicD, EcR, Ten-m, e(y)3, Nrx-1, ACC, Mmp1, ey, Pura, step, dlg1, par-1, spri, CG43897, heph, Hipk, slo, Abl, chas, Lim3, Unc-115a, cac, Pde8, Dad, cic, lncRNA:acal, crol, sd, RhoGEF64C, RecQ4, ths, fs(1)h, Rbfox1, Syt1, rl, kirre, cher, Tl, unk, sbb, Npc1b, dome, ec, Sesn, jing, kuz, caup, CG42674, dpy, eIF4EHP, luna, Dys, alpha-Cat, Trim9, Gnf1, Nlg3, crb, pwn, pyr, robo3, inv, Mef2, ap, jvl, Gprk2, Tlk, Awh, bdg, alpha-Man-Ia, sano, Sox21a, Nrg, crp, CG30456, CG13251, stau, beat-Ib, beat-IIb, CG43658, mtg, ft, qua, ara, Ser, mam, shep, foxo, shn, grn, mew, toc, Pka-R2, app, bun, CadN2, loh, pdm3, kay, tinc, PDZ-GEF, nerfin-1, l(3)psg2, dsx, chinmo, Sema2a, Bsg, klu, elB, pip, Lim1, nkd, Parp, lola, boss, vkg, Sara, TfAP-2, vvl, abd-A, nau, milt, PyK, CalpA, stai, CG5890, pnt, tefu, Hs6st, corto, ltl, Sb, Rbp6, rost, rhea, mei-P26, dia, AdamTS-A, Debcl, Rbp9, hh, drl, wdb, KCNQ, Grip, Fur1, csw, Drl-2, ush, RasGAP1, Rme-8, spz3, ko, beat-Va, CtBP, sNPF-R, tara, tyn, stan, cta, ckn, ced-6, IP3K2, zen, e, ken, Hr4, kibra, Dhc64C, Tie, al, Rok, nej, chrb, siz, CG42319, CG42663, CG8405, hbs, Patronin, en, sfl, beat-IIIb, Dop1R2, vn, TwdlQ, tut, Fas3, CG5758, pyd
anatomical structure development	306 of 856 genes, 35.7%	3173 of 16085 genes, 19.7%	3.57e-26	0.00%	0.00	fz2, Sh, Nlg1, Cpr50Cb, Fs(2)Ket, lilli, CG5921, Sxl, CLIP-190, osa, l(3)72Ab,

						sns, hid, Ccp84Ac, if, Ten-a, cno, Ptp99A, lab, poe, dsf, C3G, lt, nw, Eip75B, cv-c, nvd, Snoo, DIP-gamma, rut, pum, Pka-C3, uif, rgn, amon, Mmp2, Blimp-1, trol, alph, msi, Src42A, bbg, ds, dysc, hth, Doa, sas, FER, S, Ptp61F, PsGEF, DAAM, Scgdelta, neur, beat-Vc, per, Sox100B, Prosap, Tao, ed, opa, InR, sima, f, Tsp, wnd, RhoBTB, Cht7, Stat92E, Src64B, Btk29A, CG6701, cdi, frm, loco, fra, tral, Smr, Sema5c, Fs, Fife, dpr12, mbl, Duox, stl, bab2, egh, Gie, melt, jbug, smog, cv-2, lncRNA:flam, Msp300, T48, CG41099, lmd, ich, esn, disco-r, pot, Oaz, l(2)gl, bol, Dr, ex, beat-Ic, Poxm, Mbs, Antp, Men, futsch, beat-VI, wake, Tet, gukh, cora, spir, sff, beat-IIa, BicD, EcR, Ten-m, e(y)3, Nrx-1, ACC, Mmp1, ey, Pura, step, dlg1, par-1, spri, CG43897, heph, Hipk, slo, Abl, chas, Lim3, Unc-115a, cac, Pde8, Dad, cic, lncRNA:acal, crol, sd, RhoGEF64C, RecQ4, ths, fs(1)h, Rbfox1, Syt1, rl, kirre, cher, Tl, unk, sbb, Npc1b, dome, ec, Sesn, jing, kuz, caup, CG42674, dpy, eIF4EHP, luna, Dys, alpha-Cat, Trim9, Gnf1, Nlg3, crb, pwn, pyr, robo3, inv, Mef2, ap, jvl, Gprk2, Tlk, Awh, bdg, sano, alpha-Man-Ia, Sox21a, Nrg, crp, CG30456, CG13251, stau, beat-Ib, beat-IIb, CG43658, mtg, ft, qua, ara, Ser, mam, shep, foxo, shn, grn, mew, toc, Pka-R2, app, bun, CadN2, loh, pdm3, kay, tinc, PDZ-GEF, nerfin-1, l(3)psg2, dsx, chinmo, Sema2a, Bsg, klu, elB, pip, Lim1, nkd, Parp, lola, boss, vkg, TfAP-2, vvl, abd-A, nau, milt, PyK, CalpA, stai, CG5890, pnt, tefu, Hs6st, corto, ltl, Sb, Rbp6, rost, rhea, mei-P26, dia, AdamTS-A, Debcl, hh, Rbp9, drl, wdb, KCNQ, Grip, Fur1, csw, Drl-2, ush, RasGAP1, Rme-8, spz3, ko, CtBP, beat-Va, tara, tyn, stan, cta, ckn, ced-6, IP3K2, zen, e, ken, Hr4, kibra, Dhc64C, Tie, al, Rok, nej, chrb, siz, CG42319, CG42663, CG8405, hbs, Patronin, en, sfl, beat-IIIb, vn, TwdlQ, tut, Fas3, CG5758, pyd
epithelium development	151 of 856 genes, 17.6%	1088 of 16085 genes, 6.8%	1.71e-25	0.00%	0.00	sbb, Npc1b, dome, ec, kuz, jing, caup, Fs(2)Ket, lilli, CG42674, dpy, Sxl, osa, alpha-Cat, Dys, crb, hid, if, Mef2, inv, cno, ap, jvl, Gprk2, Awh, bdg, nw, sano, Sox21a, cv-c, Nrg, Pka-C3, Mmp2, alph, CG30456, Src42A, bbg, ds, hth, CG43658, ft, qua, ara, Ser, FER, S, mam, shn, neur, toc, mew, app, bun, Tao, ed, CadN2, opa, InR, kay, f, PDZ-GEF, l(3)psg2, Stat92E, Src64B, Btk29A, dsx, chinmo, loco, elB, Lim1, Parp, lola, vkg, Smr, TfAP-2, abd-A, Duox, stl, bab2, stai, egh, CG5890, pnt, jbug, Hs6st, corto, smog, ltl, cv-2, lncRNA:flam, Sb, CG41099, rhea, dia, disco-r, hh, drl, pot, l(2)gl, Dr, csw, ex, ush, Mbs, RasGAP1, Rme-8, CtBP, tara, tyn, stan, cta, cora, spir, ken, kibra, Dhc64C, Tie, EcR, al, Rok, e(y)3, nej, Mmp1, ey, Pura, dlg1, step, par-1, spri, heph, Hipk, CG8405, Abl, hbs, chas, cic, Dad, lncRNA:acal, crol, sd, RhoGEF64C, RecQ4, en, sfl, Rbfox1, rl, vn, cher, Fas3, pyd, unk
tissue morphogenesis	115 of 856 genes, 13.4%	691 of 16085 genes, 4.3%	2.01e-25	0.00%	0.00	Parp, sbb, lola, vkg, Npc1b, dome, ec, TfAP-2, jing, abd-A, caup, Duox, CG42674, dpy, bab2, egh, osa, pnt, alpha-Cat, Dys, jbug, Hs6st, crb, pyr, hid, corto, ltl, smog, cv-2, if, inv, cno, rhea, ap, Gprk2, disco-r, hh, pot, bdg, l(2)gl, nw, sano, cv-c, Dr, csw,

						ush, Nrg, Pka-C3, Mbs, RasGAP1, Mmp2, alph, CG30456, Src42A, stan, ds, cta, hth, CG43658, ft, qua, cora, ara, Ser, FER, S, mam, Dhc64C, shn, al, EcR, Rok, neur, mew, e(y)3, nej, app, Mmp1, ey, ed, Pura, CadN2, step, dlg1, opa, kay, par-1, heph, f, Hipk, Abl, chas, hbs, Dad, cic, lncRNA:acal, PDZ-GEF, crol, sd, l(3)psg2, Stat92E, RhoGEF64C, Src64B, en, ths, Btk29A, sfl, Rbfox1, rl, vn, cher, loco, pyd, Lim1, unk
post-embryonic animal organ development	96 of 856 genes, 11.2%	507 of 16085 genes, 3.2%	4.14e-25	0.00%	0.00	sbb, lola, dome, TfAP-2, jing, nau, caup, Duox, CG42674, dpy, bab2, osa, pnt, Dys, Hs6st, pyr, sns, hid, corto, ltl, cv-2, if, inv, Msp300, rhea, ap, Gprk2, disco-r, hh, pot, l(2)gl, nw, cv-c, Dr, ush, Nrg, crp, Poxm, Pka-C3, Mbs, RasGAP1, Mmp2, alph, CG30456, Src42A, ds, hth, CG43658, ft, qua, cora, ara, Ser, mam, S, shn, al, EcR, Rok, neur, mew, e(y)3, app, Mmp1, ed, ey, Pura, loh, dlg1, opa, step, kay, par-1, heph, f, Hipk, Dad, cic, PDZ-GEF, crol, sd, l(3)psg2, Stat92E, RhoGEF64C, en, Btk29A, ths, sfl, Rbfox1, kirre, rl, vn, cher, Tl, Lim1, unk
cell development	192 of 856 genes, 22.4%	1606 of 16085 genes, 10.0%	5.05e-25	0.00%	0.00	fz2, sbb, Sh, dome, ec, kuz, jing, Fs(2)Ket, lilli, CG5921, Sxl, osa, alpha-Cat, Trim9, Gnf1, crb, robo3, pyr, sns, hid, if, Mef2, Ten-a, Ptp99A, poe, ap, C3G, jvl, Gprk2, Eip75B, cv-c, Snoo, rut, DIP-gamma, Nrg, pum, Mmp2, trol, alph, msi, Src42A, beat-IIb, beat-Ib, stau, CG13251, bbg, ds, dysc, hth, ft, qua, Doa, sas, ara, FER, S, mam, Ptp61F, shep, shn, foxo, DAAM, grn, Scgdelta, neur, toc, mew, beat-Vc, Prosap, Pka-R2, bun, Tao, ed, CadN2, kay, InR, pdm3, sima, tinc, PDZ-GEF, l(3)psg2, nerfin-1, wnd, Stat92E, Src64B, Btk29A, CG6701, dsx, Sema2a, chinmo, Bsg, cdi, klu, loco, fra, Parp, lola, Smr, boss, vvl, abd-A, dpr12, mbl, stl, milt, stai, CalpA, egh, pnt, jbug, tefu, smog, lncRNA:flam, Msp300, CG41099, Rbp6, rhea, mei-P26, dia, esn, Debcl, Rbp9, hh, drl, pot, l(2)gl, bol, Fur1, Dr, Drl-2, csw, ex, beat-Ic, Poxm, Mbs, RasGAP1, Rme-8, Antp, ko, beat-Va, tyn, stan, ckn, cta, futsch, beat-VI, ced-6, IP3K2, Tet, gukh, cora, sff, spir, beat-IIa, BicD, Dhc64C, kibra, Tie, EcR, Rok, Ten-m, nej, Mmp1, Pura, dlg1, par-1, spri, heph, Abl, Lim3, Unc-115a, cac, cic, Dad, Patronin, sd, RhoGEF64C, RecQ4, en, fs(1)h, ths, beat-IIIb, Rbfox1, Syt1, rl, vn, tut, cher, Tl, Fas3, CG5758, unk
multicellular organismal process	369 of 856 genes, 43.1%	4222 of 16085 genes, 26.2%	8.91e-25	0.00%	0.00	fz2, Sh, Nlg1, Cpr50Cb, CG9492, Fs(2)Ket, lilli, nAChRalpha2, rdgA, CG5921, Sxl, CLIP-190, osa, l(3)72Ab, Ggamma30A, sns, hid, Ccp84Ac, if, Ten-a, cno, Ptp99A, lab, Gr23a, poe, dsf, C3G, Oct-TyrR, lt, sra, snky, dpr6, nw, Eip75B, cv-c, nvd, Snoo, DIP-gamma, rut, pum, Pka-C3, uif, mamo, lncRNA:CR43848, amon, Mmp2, Blimp-1, trol, alph, Ac78C, msi, Src42A, bbg, ds, dysc, lobo, hth, Doa, sas, FER, S, Ptp61F, MRP, nemy, polyph, PsGEF, Hk, DAAM, Scgdelta, neur, beat-Vc, per, Ir40a, Sox100B, Prosap, Tao, DNApol-epsilon255, ed, opa, InR, nAChRbeta2, Mvl, sima, f, Tif-IA, Dhc36C, Tsp, RyR, wnd, Cht7, Stat92E, Src64B, Btk29A, CG6701, cdi, frm, loco, fra, tral, Smr, Sema5c, Neurochondrin, Fs, Fife, dpr12, mbl, dpr8, Duox, stl, dpr9, bab2, Grd, egh,

						Gie, melt, jbug, rdgC, hang, smog, cv-2, lncRNA:flam, Msp300, Sap47, T48, CG41099, Imd, ich, esn, disco-r, Pde1c, pot, Oaz, l(2)gl, bol, Dr, ex, beat-Ic, Poxm, Mbs, Antp, axo, Men, trp, Or67d, futsch, beat-VI, Tet, wake, gukh, cora, spir, sff, beat-IIa, BicD, EcR, Ten-m, e(y)3, Nrx-1, Ca-alpha1T, Mmp1, Shab, ey, Pura, step, dlg1, Oamb, Drip, par-1, spri, heph, Hipk, slo, Abl, Lim3, Unc-115a, cac, CCKLR-17D1, Dad, cic, lncRNA:acal, crol, sd, CG8086, RhoGEF64C, RecQ4, ths, Gr28b, fs(1)h, Rbfox1, Syt1, rl, kirre, Orco, cher, CG30463, Tl, Gbs-70E, unk, sbb, DCX-EMAP, Npc1b, dome, ec, Sesn, jing, kuz, caup, CG42674, dpy, eIF4EHP, Obp56d, luna, CG42326, Dys, alpha-Cat, Trim9, Cnx99A, Gnf1, Nlg3, crb, pwn, pyr, robo3, inv, Mef2, ap, Nha2, jvl, Gprk2, Tlk, Awh, bdg, alpha-Man-Ia, sano, Sox21a, Nrg, crp, CG30456, CG13251, stau, beat-Ib, beat-IIb, dpr13, CG43658, mtg, ft, qua, nAChRalpha6, pHCl-1, ara, Ser, mam, shep, Gem3, foxo, shn, CG2121, Tob, grn, mew, toc, bgm, Pka-R2, app, bun, CadN2, loh, pdm3, kay, wry, hppy, tinc, CG12344, PDZ-GEF, dpr2, nerfin-1, l(3)psg2, 5-HT7, dsx, Sema2a, chinmo, Bsg, klu, elB, pip, SKIP, Lim1, nkd, Parp, lola, Gr22e, vkg, boss, TfAP-2, vvl, abd-A, nau, dpr1, milt, stai, CalpA, CG5890, pnt, tefu, Hs6st, corto, ltl, Sb, rhea, mei-P26, dia, AdamTS-A, Rbp9, hh, drl, wdb, Fur1, KCNQ, Grip, Drl-2, csw, ush, RasGAP1, Rme-8, spz3, ko, beat-Va, sNPF-R, CtBP, tyn, tara, stan, ckn, cta, ced-6, IP3K2, ken, zen, e, Hr4, nompC, Dhc64C, kibra, al, Tie, Rok, nej, ACXC, ATP8B, chrb, siz, CG42663, CG8405, hbs, Mdr50, Rh7, Patronin, en, sfl, beat-IIIb, Dop1R2, Ttd14, vn, TwdlQ, tut, Fas3, ogre, CG5758, pyd
morphogenesis of an epithelium	112 of 856 genes, 13.1%	672 of 16085 genes, 4.2%	1.00e-24	0.00%	0.00	Parp, sbb, lola, vkg, Npc1b, dome, ec, TfAP-2, jing, caup, Duox, CG42674, dpy, bab2, egh, osa, pnt, alpha-Cat, Dys, jbug, Hs6st, crb, hid, corto, ltl, smog, cv-2, if, inv, cno, rhea, ap, Gprk2, disco-r, hh, pot, bdg, l(2)gl, nw, sano, cv-c, Dr, csw, ush, Nrg, Pka-C3, Mbs, RasGAP1, Mmp2, alph, CG30456, Src42A, stan, ds, cta, hth, CG43658, ft, qua, cora, ara, Ser, FER, S, mam, Dhc64C, shn, al, EcR, Rok, neur, mew, e(y)3, nej, app, Mmp1, ey, ed, Pura, CadN2, step, dlg1, opa, kay, par-1, heph, f, Hipk, Abl, chas, hbs, Dad, cic, lncRNA:acal, PDZ-GEF, crol, sd, l(3)psg2, Stat92E, RhoGEF64C, Src64B, en, Btk29A, sfl, Rbfox1, rl, vn, cher, loco, pyd, Lim1, unk
neuron development	110 of 856 genes, 12.9%	666 of 16085 genes, 4.1%	7.05e-24	0.00%	0.00	fz2, sbb, Sh, lola, boss, vvl, kuz, jing, dpr12, lilli, mbl, CG5921, stai, CalpA, egh, pnt, Trim9, jbug, Gnf1, crb, robo3, hid, if, Ten-a, Ptp99A, ap, esn, hh, drl, pot, cv-c, Fur1, Snoo, Drl-2, csw, rut, DIP-gamma, Nrg, beat-Ic, Poxm, pum, Mbs, Mmp2, trol, ko, beat-Va, Src42A, beat-IIb, beat-Ib, CG13251, stan, dysc, ckn, futsch, beat-VI, ced-6, Doa, gukh, sas, sff, ara, FER, Ptp61F, shep, beat-IIa, Dhc64C, shn, foxo, EcR, Rok, grn, DAAM, neur, mew, beat-Vc, Ten-m, Prosap, Pka-R2, Mmp1, Pura, CadN2, pdm3, InR, kay, spri, tinc, Abl, Lim3, Unc-115a, cac, Dad, Patronin, PDZ-GEF, nerfin-1, wnd, RhoGEF64C, Src64B, en, fs(1)h, CG6701, dsx, beat-IIIb,

						Sema2a, chinmo, Bsg, cher, Tl, Fas3, CG5758, fra
cell morphogenesis	104 of 856 genes, 12.1%	608 of 16085 genes, 3.8%	1.15e-23	0.00%	0.00	fz2, sbb, Sh, lola, vvl, kuz, jing, stl, egh, alpha-Cat, Trim9, jbug, Gnf1, crb, robo3, hid, smog, if, Ten-a, cno, Ptp99A, CG41099, dia, ap, Gprk2, hh, drl, l(2)gl, cv-c, Snoo, Drl-2, rut, DIP-gamma, Nrg, beat-Ic, Poxm, pum, Mbs, Mmp2, trol, ko, beat-Va, tyn, Src42A, beat-IIb, beat-Ib, stan, ds, dysc, ckn, cta, futsch, beat-VI, ft, gukh, sas, sff, ara, FER, Ptp61F, beat-IIa, Dhc64C, shn, foxo, EcR, Rok, grn, DAAM, mew, beat-Vc, Ten-m, Prosap, Pka-R2, ACC, ed, Pura, CadN2, pdm3, InR, kay, spri, Abl, Lim3, Unc-115a, Dad, PDZ-GEF, nerfin-1, RhoBTB, wnd, Stat92E, RhoGEF64C, Src64B, en, ths, fs(1)h, CG6701, dsx, beat-IIIb, Sema2a, Bsg, cher, Fas3, fra, pyd
cell morphogenesis involved in differentiation	95 of 856 genes, 11.1%	532 of 16085 genes, 3.3%	8.52e-23	0.00%	0.00	fz2, sbb, Sh, lola, vvl, kuz, jing, stl, egh, alpha-Cat, Trim9, jbug, Gnf1, crb, robo3, hid, smog, if, Ten-a, Ptp99A, CG41099, dia, ap, Gprk2, hh, drl, cv-c, Snoo, Drl-2, rut, DIP-gamma, Nrg, beat-Ic, Poxm, pum, Mbs, Mmp2, trol, ko, beat-Va, tyn, Src42A, beat-IIb, beat-Ib, stan, ds, dysc, ckn, cta, futsch, beat-VI, ft, gukh, sas, sff, ara, FER, Ptp61F, beat-IIa, Dhc64C, foxo, shn, EcR, Rok, grn, DAAM, mew, beat-Vc, Ten-m, Prosap, Pka-R2, CadN2, pdm3, InR, kay, spri, Abl, Lim3, Unc-115a, Dad, nerfin-1, wnd, Stat92E, RhoGEF64C, Src64B, en, fs(1)h, CG6701, beat-IIIb, dsx, Sema2a, Bsg, cher, Fas3, fra
response to stimulus	288 of 856 genes, 33.6%	3053 of 16085 genes, 19.0%	1.98e-22	0.00%	0.00	fz2, Sh, nAChRalpha2, rdgA, Sxl, osa, Ggamma30A, hid, if, Ten-a, Sytbeta, cno, Ptp99A, C3G, Oct-TyrR, lt, sra, CG7094, Eip75B, cv-c, Snoo, DIP-gamma, rut, pum, Pka-C3, uif, Dh31-R, rgn, Mmp2, Blimp-1, trol, ETHR, alph, Ac78C, msi, Src42A, bbg, ds, dysc, Doa, CPT2, sas, FER, S, Ptp61F, MRP, polyph, PsGEF, CCHa1-R, DAAM, neur, beat-Vc, Ir40a, per, CG42339, Prosap, Tao, DNApol-epsilon255, ed, InR, nAChRbeta2, Mvl, sima, RyR, wnd, RhoBTB, Cht7, Stat92E, Src64B, Btk29A, cdi, loco, fra, Smr, Sema5c, Gyc88E, Fs, Duox, dpr9, Grd, egh, Gie, melt, jbug, rdgC, hang, Pde6, smog, cv-2, CG32758, Pde1c, l(2)gl, ex, beat-Ic, Lmpt, CG15611, Xrp1, trp, Or67d, futsch, beat-VI, Cbp53E, wake, gukh, sff, beat-IIa, CG14669, EcR, Ten-m, Nrx-1, ACC, Mmp1, ey, Pura, step, dlg1, Oamb, par-1, spri, TrissinR, Proc-R, Hipk, slo, Abl, Lim3, gpp, Unc-115a, cac, Pde8, Dad, cic, CCKLR-17D1, lncRNA:acal, crol, sd, Ir41a, RhoGEF64C, RecQ4, ths, Gr28b, Ac3, Syt1, rl, CG32683, Orco, cher, Tl, Gbs-70E, sbb, DCX-EMAP, CG31183, dome, CG34393, Sesn, jing, kuz, CG42674, Camta, Obp56d, Trim9, Cnx99A, TyrR, Gnf1, crb, pyr, robo3, inv, Mef2, Usp10, ap, Gprk2, Tlk, alpha-Man-Ia, Fancm, CG33298, Nrg, CG42684, CG34384, CG32447, CG30456, beat-Ib, S6KL, beat-IIb, timeout, CG43658, ft, nAChRalpha6, slgA, pHCl-1, Ser, mam, CG33639, shep, foxo, shn, grn, mew, bgm, Pka-R2, bun, CadN2, pdm3, kay, wry, CG34353, hppy, mAChR-B, Cdep, Syt7, PDZ-GEF, CG12344, nerfin-1, l(3)psg2, CG31760, 5-HT7, dsx, Sema2a, SCAP, pip, Cngl, nkd, Parp, lola, Pino, boss, vvl,

						PVRAP, Dgk, Eip93F, dpr1, PyK, CalpA, Rev1, pnt, LRR, tefu, CG9003, ltl, dia, Debcl, hh, drl, wdb, csw, Drl-2, ush, RasGAP1, spz3, ko, CtBP, sNPF-R, beat-Va, stan, RhoGAP18B, cta, ckn, kek5, ken, Hr4, nompC, kibra, Dhc64C, Tie, Rok, CG34357, nej, ACXC, ATP8B, chrb, CG4629, Keap1, siz, CG8405, hbs, Mdr50, Rh7, Tom40, en, beat-IIIb, Dop1R2, sfl, Ttd14, vn, Pde11, Fas3, ogre, pyd, Gprk1
imaginal disc development	104 of 856 genes, 12.1%	631 of 16085 genes, 3.9%	2.53e-22	0.00%	0.00	sbb, lola, Smr, dome, TfAP-2, jing, abd-A, caup, lilli, Duox, CG42674, dpy, Sxl, bab2, CG5890, osa, pnt, Dys, Hs6st, crb, hid, corto, ltl, cv-2, if, inv, Sb, rhea, ap, Gprk2, disco-r, hh, Awh, drl, pot, l(2)gl, nw, cv-c, Dr, csw, ex, Nrg, Pka-C3, Mbs, RasGAP1, Mmp2, alph, CtBP, tara, CG30456, Src42A, ds, hth, CG43658, ft, qua, cora, ara, Ser, ken, mam, S, shn, al, EcR, Rok, neur, mew, e(y)3, app, Mmp1, ed, ey, Pura, dlg1, opa, step, InR, kay, par-1, heph, f, Hipk, CG8405, Dad, cic, PDZ-GEF, crol, sd, l(3)psg2, Stat92E, RhoGEF64C, en, Btk29A, sfl, dsx, Rbfox1, chinmo, rl, vn, elB, pyd, Lim1, unk
cellular response to stimulus	209 of 856 genes, 24.4%	1918 of 16085 genes, 11.9%	3.01e-22	0.00%	0.00	fz2, Sh, DCX-EMAP, dome, CG31183, Sesn, CG34393, kuz, Camta, CG42674, nAChRalpha2, rdgA, Sxl, osa, Cnx99A, Trim9, TyrR, Gnf1, Ggamma30A, crb, pyr, hid, Sytbeta, cno, Usp10, C3G, Oct-TyrR, lt, Tlk, Gprk2, sra, CG7094, Eip75B, cv-c, Snoo, Fancm, CG33298, rut, CG42684, pum, Pka-C3, uif, CG34384, Dh31-R, rgn, Mmp2, trol, Blimp-1, ETHR, CG32447, alph, Ac78C, msi, CG30456, Src42A, S6KL, timeout, ds, CG43658, ft, nAChRalpha6, Doa, pHCl-1, Ser, FER, S, mam, Ptp61F, shep, CG33639, PsGEF, CCHa1-R, shn, foxo, neur, bgm, Prosap, Pka-R2, Tao, DNApol-epsilon255, ed, wry, kay, InR, nAChRbeta2, hppy, sima, mAChR-B, Cdep, Syt7, CG12344, PDZ-GEF, CG31760, wnd, RhoBTB, Stat92E, 5-HT7, Src64B, Btk29A, Sema2a, cdi, SCAP, loco, pip, Cngl, fra, nkd, Parp, lola, Smr, boss, Gyc88E, Fs, PVRAP, Duox, Dgk, Eip93F, Rev1, Grd, pnt, melt, tefu, LRR, rdgC, CG9003, hang, Pde6, ltl, smog, cv-2, CG32758, Debcl, hh, drl, Pde1c, l(2)gl, wdb, Drl-2, csw, ex, RasGAP1, spz3, CG15611, Xrp1, sNPF-R, CtBP, RhoGAP18B, stan, ckn, cta, trp, wake, sff, ken, kek5, Hr4, CG14669, nompC, kibra, Tie, EcR, Rok, CG34357, nej, ACXC, chrb, ACC, ey, Pura, CG4629, dlg1, Oamb, step, par-1, spri, siz, TrissinR, Proc-R, Hipk, CG8405, hbs, Rh7, gpp, cac, cic, Dad, CCKLR-17D1, Pde8, lncRNA:acal, crol, sd, RhoGEF64C, Tom40, RecQ4, Ac3, Gr28b, ths, sfl, Dop1R2, Syt1, Ttd14, rl, vn, Pde11, CG32683, Tl, ogre, pyd, Gprk1
post-embryonic development	108 of 856 genes, 12.6%	676 of 16085 genes, 4.2%	3.73e-22	0.00%	0.00	fz2, sbb, lola, dome, TfAP-2, jing, nau, caup, Duox, CG42674, dpy, eIF4EHP, bab2, osa, pnt, Dys, Hs6st, pyr, sns, hid, corto, ltl, cv-2, if, inv, Msp300, rhea, ap, Gprk2, AdamTS-A, disco-r, hh, pot, l(2)gl, nw, cv-c, Eip75B, nvd, Dr, ush, Nrg, crp, Poxm, Pka-C3, Mbs, RasGAP1, amon, Mmp2, Blimp-1, alph, CG30456, Src42A, ds, hth, CG43658, IP3K2, ft, qua, cora, sas, ara, Ser, mam, S, Hr4, foxo, shn, al, EcR, Rok, neur, mew, e(y)3, app, Mmp1, ed, ey, Pura, loh, dlg1, opa, step, kay, par-

						1, heph, f, Hipk, Dad, cic, PDZ-GEF, crol, sd, l(3)psg2, Stat92E, RhoGEF64C, RecQ4, en, Btk29A, ths, sfl, Rbfox1, kirre, rl, vn, cher, Tl, Lim1, unk
imaginal disc morphogenesis	84 of 856 genes, 9.8%	441 of 16085 genes, 2.7%	8.66e-22	0.00%	0.00	sbb, lola, dome, TfAP-2, jing, caup, Duox, CG42674, dpy, bab2, osa, pnt, Dys, Hs6st, hid, corto, ltl, cv-2, if, inv, rhea, ap, Gprk2, disco-r, hh, pot, l(2)gl, nw, cv-c, Dr, Nrg, Pka-C3, Mbs, RasGAP1, Mmp2, alph, CG30456, Src42A, ds, hth, CG43658, ft, qua, cora, ara, Ser, mam, S, shn, EcR, al, Rok, mew, neur, e(y)3, app, Mmp1, ed, ey, Pura, dlg1, opa, step, kay, par-1, heph, f, Hipk, Dad, cic, PDZ-GEF, crol, sd, l(3)psg2, Stat92E, RhoGEF64C, en, Btk29A, sfl, Rbfox1, rl, vn, Lim1, unk
post-embryonic animal organ morphogenesis	84 of 856 genes, 9.8%	441 of 16085 genes, 2.7%	8.66e-22	0.00%	0.00	sbb, lola, dome, TfAP-2, jing, caup, Duox, CG42674, dpy, bab2, osa, pnt, Dys, Hs6st, hid, corto, ltl, cv-2, if, inv, rhea, ap, Gprk2, disco-r, hh, pot, l(2)gl, nw, cv-c, Dr, Nrg, Pka-C3, Mbs, RasGAP1, Mmp2, alph, CG30456, Src42A, ds, hth, CG43658, ft, qua, cora, ara, Ser, mam, S, shn, EcR, al, Rok, mew, neur, e(y)3, app, Mmp1, ed, ey, Pura, dlg1, opa, step, kay, par-1, heph, f, Hipk, Dad, cic, PDZ-GEF, crol, sd, l(3)psg2, Stat92E, RhoGEF64C, en, Btk29A, sfl, Rbfox1, rl, vn, Lim1, unk
cellular component morphogenesis	113 of 856 genes, 13.2%	745 of 16085 genes, 4.6%	2.28e-21	0.00%	0.00	fz2, sbb, Sh, lola, vvl, kuz, jing, stl, egh, alpha-Cat, Trim9, jbug, Gnf1, crb, robo3, hid, smog, if, Ten-a, cno, Ptp99A, CG41099, rhea, dia, C3G, ap, jvl, Gprk2, hh, drl, l(2)gl, cv-c, Snoo, Drl-2, rut, DIP-gamma, Nrg, beat-Ic, Poxm, pum, Mbs, Mmp2, trol, ko, beat-Va, tyn, Src42A, beat-IIb, beat-Ib, stan, ds, dysc, ckn, cta, futsch, beat-VI, ft, gukh, sas, sff, ara, FER, S, Ptp61F, beat-IIa, Dhc64C, shn, foxo, EcR, Rok, Scgdelta, grn, DAAM, mew, beat-Vc, Ten-m, Prosap, Pka-R2, bun, ACC, ed, Pura, CadN2, pdm3, InR, kay, spri, Abl, Lim3, Unc-115a, Dad, cic, PDZ-GEF, nerfin-1, RhoBTB, wnd, Stat92E, RhoGEF64C, RecQ4, Src64B, en, ths, fs(1)h, CG6701, dsx, beat-IIIb, Sema2a, rl, Bsg, cher, Fas3, fra, pyd
imaginal disc-derived appendage development	75 of 856 genes, 8.8%	382 of 16085 genes, 2.4%	5.22e-20	0.00%	0.00	sbb, lola, TfAP-2, jing, caup, Duox, CG42674, dpy, osa, pnt, Dys, Hs6st, hid, corto, ltl, cv-2, if, inv, rhea, ap, Gprk2, disco-r, hh, drl, pot, nw, cv-c, Dr, Pka-C3, Mbs, RasGAP1, CG30456, Src42A, ds, hth, CG43658, ft, qua, cora, ara, Ser, mam, S, shn, EcR, al, Rok, mew, neur, e(y)3, app, ed, Pura, step, par-1, heph, f, Hipk, Dad, cic, PDZ-GEF, sd, l(3)psg2, crol, Stat92E, RhoGEF64C, Btk29A, en, sfl, rl, Rbfox1, vn, elB, Lim1, unk
movement of cell or subcellular component	111 of 856 genes, 13.0%	756 of 16085 genes, 4.7%	9.47e-20	0.00%	0.00	fz2, sbb, lola, dome, vvl, kuz, jing, abd-A, CG9492, stl, milt, stai, CG5142, egh, Gie, pnt, alpha-Cat, Trim9, jbug, crb, robo3, pyr, smog, if, Ten-a, Msp300, Ptp99A, rhea, dia, ap, jvl, Gprk2, AdamTS-A, hh, CG3339, drl, Drl-2, csw, ex, DIP-gamma, Nrg, beat-Ic, pum, Rme-8, Mmp2, trol, ko, beat-Va, tyn, htt, Src42A, beat-IIb, kl-2, beat-Ib, bbg, stan, dysc, ckn, cta, lobo, futsch, beat-VI, gukh, sas, FER, Ptp61F, beat-IIa, Dhc64C, kibra, EcR, Tie, Rok, grn, DAAM, mew, beat-Vc, Ten-m, nej, Pka-R2, bun, ey, CadN2, opa, pdm3, InR, kay, par-1, spri, sima, Abl, Dhc36C, Lim3, Unc-115a, Dad, PDZ-GEF, nerfin-1,

						Dhc62B, RhoBTB, Stat92E, RhoGEF64C, Src64B, en, ths, sfl, dsx, beat-IIIb, Sema2a, vn, cher, Fas3, fra
appendage development	75 of 856 genes, 8.8%	386 of 16085 genes, 2.4%	1.01e-19	0.00%	0.00	sbb, lola, TfAP-2, jing, caup, Duox, CG42674, dpy, osa, pnt, Dys, Hs6st, hid, corto, ltl, cv-2, if, inv, rhea, ap, Gprk2, disco-r, hh, drl, pot, nw, cv-c, Dr, Pka-C3, Mbs, RasGAP1, CG30456, Src42A, ds, hth, CG43658, ft, qua, cora, ara, Ser, mam, S, shn, EcR, al, Rok, mew, neur, e(y)3, app, ed, Pura, step, par-1, heph, f, Hipk, Dad, cic, PDZ-GEF, sd, l(3)psg2, crol, Stat92E, RhoGEF64C, Btk29A, en, sfl, rl, Rbfox1, vn, elB, Lim1, unk
metamorphosis	88 of 856 genes, 10.3%	517 of 16085 genes, 3.2%	2.05e-19	0.00%	0.00	Parp, sbb, lola, dome, TfAP-2, jing, caup, Duox, CG42674, dpy, bab2, osa, pnt, Dys, Hs6st, hid, corto, ltl, cv-2, if, inv, rhea, ap, Gprk2, disco-r, hh, pot, l(2)gl, nw, cv-c, nvd, Dr, Nrg, Pka-C3, Mbs, RasGAP1, Mmp2, alph, CG30456, Src42A, ds, hth, CG43658, IP3K2, ft, qua, cora, ara, Ser, mam, S, shep, shn, al, EcR, Rok, mew, neur, e(y)3, app, Mmp1, ed, ey, Pura, dlg1, opa, step, kay, par-1, heph, f, Hipk, Dad, cic, PDZ-GEF, crol, sd, l(3)psg2, Stat92E, RhoGEF64C, en, Btk29A, sfl, Rbfox1, rl, vn, Lim1, unk
post-embryonic appendage morphogenesis	72 of 856 genes, 8.4%	364 of 16085 genes, 2.3%	2.63e-19	0.00%	0.00	sbb, lola, TfAP-2, jing, caup, Duox, CG42674, dpy, osa, pnt, Dys, Hs6st, corto, ltl, cv-2, if, inv, rhea, ap, Gprk2, disco-r, hh, pot, nw, cv-c, Dr, Pka-C3, Mbs, RasGAP1, CG30456, Src42A, ds, hth, CG43658, ft, qua, cora, ara, Ser, mam, S, shn, EcR, al, Rok, mew, neur, e(y)3, app, ed, Pura, step, par-1, heph, f, Hipk, Dad, cic, PDZ-GEF, sd, l(3)psg2, crol, Stat92E, RhoGEF64C, Btk29A, en, sfl, rl, Rbfox1, vn, unk, Lim1
instar larval or pupal morphogenesis	85 of 856 genes, 9.9%	489 of 16085 genes, 3.0%	2.77e-19	0.00%	0.00	sbb, lola, dome, TfAP-2, jing, caup, Duox, CG42674, dpy, bab2, osa, pnt, Dys, Hs6st, hid, corto, ltl, cv-2, if, inv, rhea, ap, Gprk2, disco-r, hh, pot, l(2)gl, nw, cv-c, Dr, Nrg, Pka-C3, Mbs, RasGAP1, Mmp2, alph, CG30456, Src42A, ds, hth, CG43658, IP3K2, ft, qua, cora, ara, Ser, mam, S, shn, EcR, al, Rok, mew, neur, e(y)3, app, Mmp1, ed, ey, Pura, dlg1, opa, step, kay, par-1, heph, f, Hipk, Dad, cic, PDZ-GEF, crol, sd, l(3)psg2, Stat92E, RhoGEF64C, en, Btk29A, sfl, Rbfox1, rl, vn, Lim1, unk
cell projection morphogenesis	85 of 856 genes, 9.9%	489 of 16085 genes, 3.0%	2.77e-19	0.00%	0.00	fz2, sbb, Sh, lola, vvl, kuz, jing, egh, Trim9, jbug, Gnf1, crb, robo3, hid, if, Ten-a, Ptp99A, ap, hh, drl, cv-c, Snoo, Drl-2, rut, DIP-gamma, Nrg, beat-Ic, Poxm, pum, Mbs, Mmp2, trol, ko, beat-Va, Src42A, beat-IIb, beat-Ib, stan, dysc, ckn, futsch, beat-VI, gukh, sff, sas, ara, FER, Ptp61F, beat-IIa, Dhc64C, foxo, shn, EcR, Rok, grn, DAAM, mew, beat-Vc, Ten-m, Prosap, Pka-R2, Pura, CadN2, pdm3, InR, kay, spri, Abl, Lim3, Unc-115a, Dad, nerfin-1, wnd, RhoGEF64C, Src64B, en, CG6701, fs(1)h, ths, beat-IIIb, dsx, Sema2a, cher, Fas3, fra
imaginal disc-derived appendage morphogenesis	73 of 856 genes, 8.5%	375 of 16085 genes, 2.3%	3.55e-19	0.00%	0.00	sbb, lola, TfAP-2, jing, caup, Duox, CG42674, dpy, osa, pnt, Dys, Hs6st, hid, corto, ltl, cv-2, if, inv, rhea, ap, Gprk2, disco-r, hh, pot, nw, cv-c, Dr, Pka-C3, Mbs, RasGAP1, CG30456, Src42A, ds, hth, CG43658, ft, qua, cora, ara, Ser, mam, S, shn, EcR, al, Rok, mew, neur, e(y)3, app, ed, Pura, step, par-1, heph, f, Hipk, Dad, cic, PDZ-GEF, sd, l(3)psg2, crol, Stat92E,

						RhoGEF64C, Btk29A, en, sfl, rl, Rbfox1, vn, unk, Lim1
regulation of cell communication	120 of 856 genes, 14.0%	872 of 16085 genes, 5.4%	3.81e-19	0.00%	0.00	Parp, Sh, lola, Smr, Sesn, CG34393, Nlg1, kuz, Fs, Fife, PVRAP, Camta, CG42674, rdgA, Sxl, Grd, pnt, Dys, melt, TyrR, tefu, LRR, Nlg3, rdgC, crb, ltl, cv-2, Sap47, cno, GluRIB, Usp10, Oct-TyrR, Gprk2, sra, l(2)gl, CG7094, wdb, cv-c, Snoo, ex, rut, CG42684, pum, uif, RasGAP1, spz3, Mmp2, trol, CG15611, CG32447, alph, sNPF-R, CtBP, CG30456, Src42A, Snap25, ds, dysc, kek6, CG43658, Cbp53E, ft, GluRIA, Doa, ken, S, kek5, Ptp61F, nemy, Hr4, PsGEF, BicD, Mctp, kibra, foxo, Rok, neur, Nrx-1, nej, chrb, Prosap, Tao, Shab, ey, ed, Pura, step, dlg1, InR, kay, par-1, spri, hppy, siz, Hipk, slo, KaiR1D, hbs, Cdep, cac, Dad, Pde8, CG12344, lncRNA:acal, crol, wnd, RhoGEF64C, Src64B, Syt1, rl, vn, cdi, CG32683, Tl, loco, pip, fra, pyd, Gprk1, nkd
regulation of multicellular organismal process	114 of 856 genes, 13.3%	804 of 16085 genes, 5.0%	4.27e-19	0.00%	0.00	fz2, sbb, Sh, lola, ec, TfAP-2, vvl, kuz, abd-A, nau, stl, eIF4EHP, Grd, egh, osa, pnt, Trim9, Hs6st, crb, pyr, hid, smog, Mef2, cno, dia, dsf, hh, l(2)gl, cv-c, Eip75B, KCNQ, csw, ex, ush, rut, pum, Mbs, RasGAP1, Rme-8, Antp, alph, sNPF-R, tyn, Src42A, stau, stan, ds, dysc, cta, hth, futsch, ft, wake, Doa, Ser, FER, e, Hr4, Dhc64C, kibra, foxo, shn, Hk, EcR, Tie, CG2121, Rok, DAAM, neur, per, bgm, Prosap, bun, Tao, Shab, ey, ed, Oamb, dlg1, step, InR, kay, Drip, par-1, spri, Hipk, tinc, slo, Abl, hbs, cac, Dad, CG12344, PDZ-GEF, RyR, sd, nerfin-1, Src64B, sfl, Dop1R2, Sema2a, Syt1, kirre, rl, vn, cdi, klu, cher, Tl, pip, fra, pyd, unk, tral
post-embryonic animal morphogenesis	86 of 856 genes, 10.0%	503 of 16085 genes, 3.1%	4.81e-19	0.00%	0.00	sbb, lola, dome, TfAP-2, jing, caup, Duox, CG42674, dpy, bab2, osa, pnt, Dys, Hs6st, hid, corto, ltl, cv-2, if, inv, rhea, ap, Gprk2, AdamTS-A, disco-r, hh, pot, l(2)gl, nw, cv-c, Dr, Nrg, Pka-C3, Mbs, RasGAP1, Mmp2, alph, CG30456, Src42A, ds, hth, CG43658, IP3K2, ft, qua, cora, ara, Ser, mam, S, shn, al, EcR, Rok, mew, neur, e(y)3, app, Mmp1, ed, ey, Pura, dlg1, opa, step, kay, par-1, heph, f, Hipk, Dad, cic, PDZ-GEF, crol, sd, l(3)psg2, Stat92E, RhoGEF64C, en, Btk29A, sfl, Rbfox1, rl, vn, Lim1, unk
regulation of signaling	120 of 856 genes, 14.0%	875 of 16085 genes, 5.4%	5.13e-19	0.00%	0.00	Parp, Sh, lola, Smr, Sesn, CG34393, Nlg1, kuz, Fs, Fife, PVRAP, Camta, CG42674, rdgA, Sxl, Grd, pnt, Dys, melt, TyrR, tefu, LRR, Nlg3, rdgC, crb, ltl, cv-2, Sap47, cno, GluRIB, Usp10, Oct-TyrR, Gprk2, sra, l(2)gl, CG7094, wdb, cv-c, Snoo, ex, rut, CG42684, pum, uif, RasGAP1, spz3, Mmp2, trol, CG15611, CG32447, alph, sNPF-R, CtBP, CG30456, Src42A, Snap25, ds, dysc, kek6, CG43658, Cbp53E, ft, GluRIA, Doa, ken, S, kek5, Ptp61F, nemy, Hr4, PsGEF, BicD, Mctp, kibra, foxo, Rok, neur, Nrx-1, nej, chrb, Prosap, Tao, Shab, ey, ed, Pura, step, dlg1, InR, kay, par-1, spri, hppy, siz, Hipk, slo, KaiR1D, hbs, Cdep, cac, Dad, Pde8, CG12344, lncRNA:acal, crol, wnd, RhoGEF64C, Src64B, Syt1, rl, vn, cdi, CG32683, Tl, loco, pip, fra, pyd, Gprk1, nkd
appendage morphogenesis	73 of 856 genes, 8.5%	378 of 16085 genes, 2.4%	5.83e-19	0.00%	0.00	sbb, lola, TfAP-2, jing, caup, Duox, CG42674, dpy, osa, pnt, Dys, Hs6st, hid,

						corto, ltl, cv-2, if, inv, rhea, ap, Gprk2, disco-r, hh, pot, nw, cv-c, Dr, Pka-C3, Mbs, RasGAP1, CG30456, Src42A, ds, hth, CG43658, ft, qua, cora, ara, Ser, mam, S, shn, EcR, al, Rok, mew, neur, e(y)3, app, ed, Pura, step, par-1, heph, f, Hipk, Dad, cic, PDZ-GEF, sd, l(3)psg2, crol, Stat92E, RhoGEF64C, Btk29A, en, sfl, rl, Rbfox1, vn, unk, Lim1
cell morphogenesis involved in neuron differentiation	84 of 856 genes, 9.8%	485 of 16085 genes, 3.0%	6.44e-19	0.00%	0.00	fz2, sbb, Sh, lola, vvl, kuz, jing, egh, Trim9, jbug, Gnf1, crb, robo3, hid, if, Ten-a, Ptp99A, ap, hh, drl, cv-c, Snoo, Drl-2, rut, DIP-gamma, Nrg, beat-Ic, Poxm, pum, Mbs, Mmp2, trol, ko, beat-Va, Src42A, beat-IIb, beat-Ib, stan, dysc, ckn, futsch, beat-VI, gukh, sff, sas, ara, FER, Ptp61F, beat-IIa, Dhc64C, foxo, shn, EcR, Rok, grn, DAAM, mew, beat-Vc, Ten-m, Prosap, Pka-R2, CadN2, pdm3, InR, kay, spri, Abl, Lim3, Unc-115a, Dad, nerfin-1, wnd, RhoGEF64C, Src64B, en, CG6701, fs(1)h, beat-IIIb, dsx, Sema2a, Bsg, cher, Fas3, fra
neuron projection morphogenesis	84 of 856 genes, 9.8%	486 of 16085 genes, 3.0%	7.41e-19	0.00%	0.00	fz2, sbb, Sh, lola, vvl, kuz, jing, egh, Trim9, jbug, Gnf1, crb, robo3, hid, if, Ten-a, Ptp99A, ap, hh, drl, cv-c, Snoo, Drl-2, rut, DIP-gamma, Nrg, beat-Ic, Poxm, pum, Mbs, Mmp2, trol, ko, beat-Va, Src42A, beat-IIb, beat-Ib, stan, dysc, ckn, futsch, beat-VI, gukh, sff, sas, ara, FER, Ptp61F, beat-IIa, Dhc64C, foxo, shn, EcR, Rok, grn, DAAM, mew, beat-Vc, Ten-m, Prosap, Pka-R2, Pura, CadN2, pdm3, InR, kay, spri, Abl, Lim3, Unc-115a, Dad, nerfin-1, wnd, RhoGEF64C, Src64B, en, CG6701, fs(1)h, beat-IIIb, dsx, Sema2a, cher, Fas3, fra
cell part morphogenesis	85 of 856 genes, 9.9%	497 of 16085 genes, 3.1%	8.45e-19	0.00%	0.00	fz2, sbb, Sh, lola, vvl, kuz, jing, egh, Trim9, jbug, Gnf1, crb, robo3, hid, if, Ten-a, Ptp99A, ap, hh, drl, cv-c, Snoo, Drl-2, rut, DIP-gamma, Nrg, beat-Ic, Poxm, pum, Mbs, Mmp2, trol, ko, beat-Va, Src42A, beat-IIb, beat-Ib, stan, dysc, ckn, futsch, beat-VI, gukh, sff, sas, ara, FER, Ptp61F, beat-IIa, Dhc64C, foxo, shn, EcR, Rok, grn, DAAM, mew, beat-Vc, Ten-m, Prosap, Pka-R2, Pura, CadN2, pdm3, InR, kay, spri, Abl, Lim3, Unc-115a, Dad, nerfin-1, wnd, RhoGEF64C, Src64B, en, CG6701, fs(1)h, ths, beat-IIIb, dsx, Sema2a, cher, Fas3, fra
plasma membrane bounded cell projection morphogenesis	84 of 856 genes, 9.8%	487 of 16085 genes, 3.0%	8.52e-19	0.00%	0.00	fz2, sbb, Sh, lola, vvl, kuz, jing, egh, Trim9, jbug, Gnf1, crb, robo3, hid, if, Ten-a, Ptp99A, ap, hh, drl, cv-c, Snoo, Drl-2, rut, DIP-gamma, Nrg, beat-Ic, Poxm, pum, Mbs, Mmp2, trol, ko, beat-Va, Src42A, beat-IIb, beat-Ib, stan, dysc, ckn, futsch, beat-VI, gukh, sff, sas, ara, FER, Ptp61F, beat-IIa, Dhc64C, foxo, shn, EcR, Rok, grn, DAAM, mew, beat-Vc, Ten-m, Prosap, Pka-R2, Pura, CadN2, pdm3, InR, kay, spri, Abl, Lim3, Unc-115a, Dad, nerfin-1, wnd, RhoGEF64C, Src64B, en, CG6701, fs(1)h, beat-IIIb, dsx, Sema2a, cher, Fas3, fra
locomotion	105 of 856 genes, 12.3%	710 of 16085 genes, 4.4%	9.30e-19	0.00%	0.00	fz2, sbb, lola, dome, vvl, kuz, jing, abd-A, rdgA, stl, stai, egh, pnt, Trim9, jbug, rdgC, robo3, pyr, smog, if, Ten-a, Msp300, Ptp99A, rhea, ap, AdamTS-A, hh, drl, Drl-2, csw, ex, DIP-gamma, Nrg, beat-Ic, pum, Rme-8, Mmp2, trol, ko, beat-Va, Src42A, beat-IIb, beat-Ib, bbg, stan, dysc, ckn, cta,

						lobo, beat-VI, gukh, sas, slgA, FER, Ptp61F, shep, beat-IIa, nompC, Dhc64C, kibra, EcR, Tie, grn, DAAM, mew, beat-Vc, Ten-m, Nrx-1, nej, Pka-R2, bun, ey, CadN2, dlg1, opa, pdm3, InR, kay, par-1, CG34353, spri, sima, Hipk, Abl, Lim3, Unc-115a, Dad, PDZ-GEF, nerfin-1, RhoBTB, Stat92E, RhoGEF64C, Src64B, en, Gr28b, ths, sfl, beat-IIIb, dsx, Sema2a, vn, cher, Tl, Fas3, fra
instar larval or pupal development	95 of 856 genes, 11.1%	605 of 16085 genes, 3.8%	1.44e-18	0.00%	0.00	fz2, sbb, lola, dome, TfAP-2, jing, caup, Duox, CG42674, dpy, eIF4EHP, bab2, osa, pnt, Dys, Hs6st, hid, corto, ltl, cv-2, if, inv, rhea, ap, Gprk2, AdamTS-A, disco-r, hh, pot, l(2)gl, nw, cv-c, Eip75B, nvd, Dr, Nrg, Pka-C3, Mbs, RasGAP1, amon, Mmp2, Blimp-1, alph, CG30456, Src42A, ds, hth, CG43658, IP3K2, ft, qua, cora, sas, ara, Ser, mam, S, Hr4, foxo, shn, al, EcR, Rok, neur, mew, e(y)3, app, Mmp1, ed, ey, Pura, dlg1, opa, step, kay, par-1, heph, f, Hipk, Dad, cic, PDZ-GEF, crol, sd, l(3)psg2, Stat92E, RhoGEF64C, en, Btk29A, sfl, Rbfox1, rl, vn, Lim1, unk
neuron projection development	87 of 856 genes, 10.2%	522 of 16085 genes, 3.2%	1.58e-18	0.00%	0.00	fz2, sbb, Sh, lola, vvl, kuz, jing, stai, egh, Trim9, jbug, Gnf1, crb, robo3, hid, if, Ten-a, Ptp99A, ap, hh, drl, cv-c, Snoo, Drl-2, rut, DIP-gamma, Nrg, beat-Ic, Poxm, pum, Mbs, Mmp2, trol, ko, beat-Va, Src42A, beat-IIb, beat-Ib, stan, dysc, ckn, futsch, beat-VI, gukh, sff, sas, ara, FER, Ptp61F, beat-IIa, Dhc64C, foxo, shn, EcR, Rok, grn, DAAM, mew, beat-Vc, Ten-m, Prosap, Pka-R2, Mmp1, Pura, CadN2, pdm3, InR, kay, spri, Abl, Lim3, Unc-115a, Dad, Patronin, nerfin-1, wnd, RhoGEF64C, Src64B, en, CG6701, fs(1)h, beat-IIIb, dsx, Sema2a, cher, Fas3, fra
positive regulation of biological process	179 of 856 genes, 20.9%	1639 of 16085 genes, 10.2%	2.07e-18	0.00%	0.00	Sh, dome, ec, Sesn, CG34393, kuz, jing, caup, lilli, Camta, CG42674, tna, eIF4EHP, Sxl, osa, CG33144, TyrR, CG12054, crb, AGO3, pyr, hid, Mef2, Ten-a, lab, Hcf, Usp10, dsf, ap, Oct-TyrR, Gprk2, Awh, Eip75B, cv-c, Snoo, CG8312, pum, Mmp2, trol, stv, CG30456, Src42A, stau, Tis11, ds, dysc, hth, CG43658, ft, Doa, ara, S, mam, PsGEF, shn, foxo, Hk, DAAM, neur, bgm, CG17514, Prosap, bun, Tao, ed, loh, opa, InR, kay, Mvl, hppy, sima, Apoltp, CG12769, CG11247, l(3)psg2, nerfin-1, wnd, Stat92E, Src64B, dsx, Sema2a, chinmo, klu, SCAP, pip, fra, Lim1, tral, Parp, MBD-R2, lola, TfAP-2, Fs, vvl, Fife, abd-A, PVRAP, nau, Eip93F, pnt, melt, smog, cv-2, dia, Imd, ich, Debcl, hh, Oaz, l(2)gl, wdb, bol, ex, ush, RasGAP1, Rme-8, Antp, CG15611, sNPF-R, CtBP, htt, tara, stan, Cbp53E, CG11486, wake, Tet, Jupiter, CG4238, spir, zen, Hr4, BicD, Dhc64C, kibra, Tie, EcR, Rok, Ten-m, e(y)3, Nrx-1, nej, Shab, ey, Pura, CG31612, dlg1, Oamb, step, smg, spri, siz, Hipk, KaiR1D, CG8405, Abl, hbs, ps, Lim3, gpp, cac, Pde8, crol, sd, RhoGEF64C, RecQ4, en, fs(1)h, Dop1R2, Rbfox1, rl, Ttd14, Syt1, vn, cher, Tl, pyd, Gprk1
epithelial tube morphogenesis	87 of 856 genes, 10.2%	527 of 16085 genes, 3.3%	3.05e-18	0.00%	0.00	sbb, lola, vkg, dome, TfAP-2, jing, caup, Duox, CG42674, dpy, bab2, osa, pnt, Dys, Hs6st, crb, hid, corto, ltl, cv-2, if, inv, rhea, ap, Gprk2, disco-r, hh, pot, l(2)gl, nw, cv-c, Dr, Nrg, Pka-C3, Mbs, RasGAP1, Mmp2, alph, CG30456, Src42A, ds, hth, CG43658, ft, qua, cora, ara, Ser, mam, S, shn, al,

						EcR, Rok, mew, neur, e(y)3, app, Mmp1, ed, ey, Pura, dlg1, opa, step, kay, par-1, heph, f, Hipk, Dad, cic, PDZ-GEF, crol, sd, l(3)psg2, Stat92E, RhoGEF64C, en, Btk29A, sfl, Rbfox1, rl, vn, pyd, Lim1, unk
tube development	112 of 856 genes, 13.1%	801 of 16085 genes, 5.0%	3.28e-18	0.00%	0.00	sbb, lola, Smr, vkg, dome, TfAP-2, jing, abd-A, caup, lilli, Duox, CG42674, dpy, Sxl, bab2, CG5890, osa, pnt, Dys, Hs6st, crb, hid, corto, ltl, cv-2, if, Mef2, inv, Sb, lab, rhea, ap, ich, Gprk2, disco-r, hh, Awh, drl, pot, l(2)gl, nw, Sox21a, cv-c, Dr, csw, ex, Nrg, Pka-C3, Mbs, RasGAP1, Mmp2, Antp, alph, CtBP, tara, CG30456, Src42A, ds, hth, CG43658, ft, qua, cora, ara, Ser, ken, mam, S, Dhc64C, shn, al, EcR, Rok, neur, mew, e(y)3, app, Mmp1, ed, ey, Pura, dlg1, opa, step, InR, kay, par-1, heph, f, Hipk, CG8405, hbs, Dad, cic, PDZ-GEF, crol, sd, l(3)psg2, Stat92E, RhoGEF64C, en, Btk29A, sfl, dsx, Rbfox1, chinmo, rl, vn, elB, pyd, Lim1, unk
response to chemical	147 of 856 genes, 17.2%	1250 of 16085 genes, 7.8%	2.23e-17	0.00%	0.00	fz2, sbb, Sh, dome, Sesn, kuz, jing, Obp56d, Cnx99A, Trim9, TyrR, Ggamma30A, robo3, pyr, hid, if, Ten-a, Sytbeta, Ptp99A, ap, sra, alpha-Man-Ia, Eip75B, Snoo, rut, DIP-gamma, Nrg, pum, Mmp2, trol, Blimp-1, ETHR, alph, Ac78C, msi, Src42A, beat-IIb, beat-Ib, dysc, nAChRalpha6, sas, FER, S, Ptp61F, MRP, PsGEF, shn, foxo, DAAM, grn, mew, beat-Vc, Ir40a, per, Pka-R2, Tao, CadN2, pdm3, InR, Mvl, nAChRbeta2, hppy, sima, mAChR-B, Syt7, RyR, l(3)psg2, nerfin-1, Stat92E, Src64B, dsx, Sema2a, fra, lola, Pino, boss, Sema5c, Gyc88E, Fs, vvl, Duox, Eip93F, dpr1, dpr9, PyK, egh, pnt, melt, jbug, LRR, tefu, hang, ltl, cv-2, hh, drl, l(2)gl, wdb, csw, Drl-2, beat-Ic, RasGAP1, ko, beat-Va, stan, ckn, Or67d, trp, futsch, beat-VI, gukh, kek5, beat-IIa, Hr4, EcR, Ten-m, Nrx-1, ATP8B, ACC, Oamb, dlg1, step, TrissinR, slo, CG8405, Abl, Mdr50, Lim3, Unc-115a, Dad, Pde8, Ir41a, RhoGEF64C, Tom40, en, ths, beat-IIIb, Dop1R2, sfl, rl, Syt1, vn, Orco, cher, Gbs-70E, Tl, Fas3
positive regulation of cellular process	163 of 856 genes, 19.0%	1464 of 16085 genes, 9.1%	3.17e-17	0.00%	0.00	dome, ec, Sesn, CG34393, kuz, jing, caup, lilli, Camta, CG42674, tna, eIF4EHP, Sxl, osa, CG33144, TyrR, CG12054, crb, AGO3, pyr, hid, Mef2, lab, Hcf, Usp10, dsf, ap, Oct-TyrR, Gprk2, Awh, Eip75B, cv-c, Snoo, CG8312, pum, trol, stv, CG30456, Src42A, stau, Tis11, ds, hth, CG43658, ft, Doa, ara, S, mam, PsGEF, shn, foxo, Hk, DAAM, neur, CG17514, Prosap, bun, Tao, ed, loh, opa, InR, kay, hppy, sima, CG12769, CG11247, l(3)psg2, nerfin-1, wnd, Stat92E, Src64B, dsx, Sema2a, chinmo, klu, SCAP, pip, fra, Lim1, Parp, MBD-R2, lola, TfAP-2, Fs, vvl, Fife, abd-A, PVRAP, nau, Eip93F, pnt, melt, smog, cv-2, dia, Imd, ich, Debcl, Oaz, l(2)gl, wdb, bol, ex, ush, RasGAP1, Rme-8, Antp, CG15611, sNPF-R, CtBP, htt, tara, stan, CG11486, Cbp53E, Tet, Jupiter, spir, zen, BicD, kibra, Dhc64C, Tie, EcR, Rok, e(y)3, Nrx-1, nej, ey, Pura, CG31612, Oamb, dlg1, step, smg, spri, siz, Hipk, KaiR1D, CG8405, Abl, hbs, ps, gpp, cac, Pde8, crol, sd, RhoGEF64C, RecQ4, en, fs(1)h, Dop1R2, Rbfox1, rl, Syt1, vn, cher, Tl, pyd, Gprk1
tube morphogenesis	89 of 856	572 of 16085	6.05e-17	0.00%	0.00	sbb, lola, vkg, dome, TfAP-2, jing, caup,

	genes, 10.4%	genes, 3.6%				Duox, CG42674, dpy, bab2, osa, pnt, Dys, Hs6st, crb, hid, corto, ltl, cv-2, if, inv, rhea, ap, ich, Gprk2, disco-r, hh, pot, l(2)gl, nw, cv-c, Dr, Nrg, Pka-C3, Mbs, RasGAP1, Mmp2, alph, CG30456, Src42A, ds, hth, CG43658, ft, qua, cora, ara, Ser, mam, S, Dhc64C, shn, al, EcR, Rok, mew, neur, e(y)3, app, Mmp1, ed, ey, Pura, dlg1, opa, step, kay, par-1, heph, f, Hipk, Dad, cic, PDZ-GEF, crol, sd, l(3)psg2, Stat92E, RhoGEF64C, en, Btk29A, sfl, Rbfox1, rl, vn, pyd, Lim1, unk
axonogenesis	68 of 856 genes, 7.9%	360 of 16085 genes, 2.2%	6.12e-17	0.00%	0.00	fz2, sbb, Sh, lola, vvl, kuz, jing, egh, Trim9, jbug, robo3, if, Ten-a, Ptp99A, ap, hh, drl, Drl-2, DIP-gamma, rut, Nrg, beat-Ic, Mbs, Mmp2, trol, ko, beat-Va, Src42A, beat-Ib, beat-IIb, stan, dysc, ckn, futsch, beat-VI, gukh, sff, sas, FER, Ptp61F, beat-IIa, grn, DAAM, Rok, mew, beat-Vc, Ten-m, Pka-R2, CadN2, pdm3, InR, kay, spri, Abl, Unc-115a, Lim3, Dad, nerfin-1, wnd, RhoGEF64C, Src64B, en, beat-IIIb, dsx, Sema2a, cher, Fas3, fra
axon development	69 of 856 genes, 8.1%	375 of 16085 genes, 2.3%	1.43e-16	0.00%	0.00	fz2, sbb, Sh, lola, vvl, kuz, jing, egh, Trim9, jbug, robo3, if, Ten-a, Ptp99A, ap, hh, drl, Drl-2, DIP-gamma, rut, Nrg, beat-Ic, Mbs, Mmp2, trol, ko, beat-Va, Src42A, beat-Ib, beat-IIb, stan, dysc, ckn, futsch, beat-VI, gukh, sff, sas, FER, Ptp61F, beat-IIa, Rok, grn, DAAM, mew, beat-Vc, Ten-m, Pka-R2, Mmp1, CadN2, pdm3, InR, kay, spri, Abl, Unc-115a, Lim3, Dad, nerfin-1, wnd, RhoGEF64C, Src64B, en, beat-IIIb, dsx, Sema2a, cher, Fas3, fra
wing disc development	74 of 856 genes, 8.6%	427 of 16085 genes, 2.7%	2.25e-16	0.00%	0.00	sbb, lola, Smr, dome, jing, caup, lilli, Duox, dpy, CG5890, osa, pnt, Dys, Hs6st, crb, corto, ltl, cv-2, if, inv, Sb, rhea, ap, Gprk2, disco-r, hh, pot, nw, cv-c, Dr, Pka-C3, Mbs, RasGAP1, CtBP, tara, Src42A, ds, hth, ft, qua, cora, ara, Ser, mam, S, shn, EcR, Rok, mew, neur, app, ed, step, kay, par-1, heph, f, Hipk, CG8405, cic, Dad, PDZ-GEF, sd, crol, Stat92E, Btk29A, en, sfl, rl, Rbfox1, vn, elB, pyd, unk
imaginal disc-derived wing morphogenesis	62 of 856 genes, 7.2%	315 of 16085 genes, 2.0%	3.56e-16	0.00%	0.00	sbb, hth, lola, ft, qua, jing, cora, caup, ara, Ser, S, mam, Duox, dpy, shn, EcR, Rok, neur, mew, osa, pnt, Dys, app, Hs6st, corto, ltl, ed, cv-2, if, inv, step, par-1, heph, f, rhea, Hipk, ap, Gprk2, disco-r, hh, pot, Dad, cic, nw, PDZ-GEF, crol, sd, cv-c, Dr, Stat92E, en, Btk29A, sfl, Rbfox1, Pka-C3, rl, Mbs, RasGAP1, vn, Src42A, ds, unk
regulation of biological quality	153 of 856 genes, 17.9%	1366 of 16085 genes, 8.5%	3.59e-16	0.00%	0.00	Sh, kuz, lilli, CG8177, Vps13D, nAChRalpha2, rdgA, dpy, eIF4EHP, Sxl, alpha-Cat, Dys, Cnx99A, crb, if, Mef2, Ten-a, Sytbeta, cno, dsf, sra, nw, sano, Eip75B, cv-c, nvd, Kank, CG33298, DIP-gamma, Nrg, pum, unc-13-4A, uif, amon, Blimp-1, Src42A, stau, Snap25, CG1090, Tis11, ds, dysc, Ncc69, kek6, CG40178, ft, nrv1, nAChRalpha6, CPT2, pHCl-1, FER, Ptp61F, nemy, PsGEF, foxo, Mctp, DAAM, Scgdelta, mew, per, bgm, Prosap, bun, Tao, ed, InR, Mvl, nAChRbeta2, hppy, Syt7, Trpm, CG12344, PDZ-GEF, RyR, CG30377, wnd, RhoBTB, Src64B, Btk29A, Sema2a, chinmo, fra, Parp, lola, boss, Sara, NKCC, Fife, mbl, PyK, egh, Grd, Gie, Elk, tefu, Msp300, Sap47, rhea, dia, Imd, ich, Debcl, l(2)gl, wdb, bol, KCNQ, Fur1, ush, Rph, RasGAP1, tyn, stan, cta, trp, futsch, Cbp53E, cora, spir, e, BicD,

						Dhc64C, EcR, Rok, Ten-m, Nrx-1, nej, ATP8B, Ca-alpha1T, Shab, Mmp1, ey, Oamb, dlg1, Drip, SK, smg, Hipk, slo, KaiR1D, Abl, Trxr-2, cac, CCKLR-17D1, Dad, Gyf, Dop1R2, rl, Ttd14, Syt1, olf413, SerT, unc80, pyd
wing disc morphogenesis	63 of 856 genes, 7.4%	325 of 16085 genes, 2.0%	4.06e-16	0.00%	0.00	sbb, hth, lola, dome, ft, qua, jing, cora, caup, ara, Ser, S, mam, Duox, dpy, shn, EcR, Rok, neur, mew, osa, pnt, Dys, app, Hs6st, corto, Itl, ed, cv-2, if, inv, step, par-1, heph, f, rhea, Hipk, ap, Gprk2, disco-r, hh, pot, Dad, cic, nw, PDZ-GEF, crol, sd, cv-c, Dr, Stat92E, en, Btk29A, sfl, Rbfox1, Pka-C3, rl, Mbs, RasGAP1, vn, Src42A, ds, unk
cell projection organization	102 of 856 genes, 11.9%	734 of 16085 genes, 4.6%	4.08e-16	0.00%	0.00	fz2, sbb, Sh, lola, vvl, kuz, jing, stai, CG5142, egh, Trim9, jbug, LRR, Gnf1, crb, robo3, hid, if, Ten-a, Ptp99A, dia, dtr, ap, hh, drl, cv-c, Snoo, Drl-2, rut, DIP-gamma, Nrg, beat-Ic, Poxm, pum, Mbs, Mmp2, trol, ko, beat-Va, Src42A, beat-IIb, beat-Ib, stan, ds, dysc, ckn, futsch, beat-VI, ft, qua, gukh, cora, sas, sff, ara, FER, Ptp61F, beat-IIa, PsGEF, Dhc64C, shn, foxo, EcR, Rok, grn, DAAM, mew, beat-Vc, Ten-m, Prosap, Pka-R2, app, Mmp1, Pura, CadN2, pdm3, InR, kay, par-1, spri, f, Abl, Lim3, Unc-115a, Dad, Patronin, PDZ-GEF, nerfin-1, wnd, RhoGEF64C, Src64B, en, ths, fs(1)h, CG6701, beat-IIIb, dsx, Sema2a, vn, cher, Fas3, fra
plasma membrane bounded cell projection organization	100 of 856 genes, 11.7%	721 of 16085 genes, 4.5%	1.10e-15	0.00%	0.00	fz2, sbb, Sh, lola, vvl, kuz, jing, stai, CG5142, egh, Trim9, jbug, LRR, Gnf1, crb, robo3, hid, if, Ten-a, Ptp99A, dia, dtr, ap, hh, drl, cv-c, Snoo, Drl-2, rut, DIP-gamma, Nrg, beat-Ic, Poxm, pum, Mbs, Mmp2, trol, ko, beat-Va, Src42A, beat-IIb, beat-Ib, stan, ds, dysc, ckn, futsch, beat-VI, ft, qua, gukh, cora, sas, sff, ara, FER, Ptp61F, beat-IIa, PsGEF, Dhc64C, shn, foxo, EcR, Rok, grn, DAAM, mew, beat-Vc, Ten-m, Prosap, Pka-R2, app, Mmp1, Pura, CadN2, pdm3, InR, kay, par-1, spri, f, Abl, Lim3, Unc-115a, Dad, Patronin, PDZ-GEF, nerfin-1, wnd, RhoGEF64C, Src64B, en, fs(1)h, CG6701, beat-IIIb, dsx, Sema2a, cher, Fas3, fra
chemotaxis	60 of 856 genes, 7.0%	304 of 16085 genes, 1.9%	1.22e-15	0.00%	0.00	fz2, sbb, lola, beat-VI, vvl, kuz, jing, gukh, sas, FER, Ptp61F, beat-IIa, DAAM, grn, beat-Vc, mew, egh, Ten-m, Trim9, jbug, Pka-R2, robo3, if, Ten-a, CadN2, InR, pdm3, Ptp99A, ap, Abl, hh, drl, Lim3, Unc-115a, Dad, nerfin-1, Drl-2, RhoGEF64C, Src64B, en, DIP-gamma, Nrg, beat-Ic, dsx, beat-IIIb, Sema2a, Mmp2, trol, ko, beat-Va, cher, Tl, Src42A, beat-Ib, Fas3, beat-IIb, fra, stan, dysc, ckn
axon guidance	58 of 856 genes, 6.8%	286 of 16085 genes, 1.8%	1.25e-15	0.00%	0.00	fz2, sbb, lola, beat-VI, vvl, kuz, jing, gukh, sas, FER, Ptp61F, beat-IIa, DAAM, grn, beat-Vc, mew, egh, Ten-m, Trim9, jbug, Pka-R2, robo3, if, Ten-a, CadN2, InR, pdm3, Ptp99A, ap, Abl, drl, Lim3, Unc-115a, Dad, nerfin-1, Drl-2, RhoGEF64C, Src64B, en, DIP-gamma, Nrg, beat-Ic, dsx, beat-IIIb, Sema2a, Mmp2, trol, ko, beat-Va, cher, Src42A, beat-Ib, Fas3, beat-IIb, fra, stan, dysc, ckn
taxis	68 of 856 genes, 7.9%	383 of 16085 genes, 2.4%	1.99e-15	0.00%	0.00	fz2, sbb, lola, vvl, kuz, jing, rdgA, egh, Trim9, jbug, rdgC, robo3, if, Ten-a, Ptp99A, ap, hh, drl, Drl-2, DIP-gamma, Nrg, beat-Ic, Mmp2, trol, ko, beat-Va, Src42A, beat-Ib, beat-IIb, stan, dysc, ckn, beat-VI, gukh, slgA, sas, FER, Ptp61F, shep, beat-IIa,

						grn, DAAM, mew, beat-Vc, Ten-m, Pka-R2, CadN2, dlg1, pdm3, InR, CG34353, Hipk, Abl, Unc-115a, Lim3, Dad, nerfin-1, RhoGEF64C, Src64B, Gr28b, en, beat-IIIb, dsx, Sema2a, cher, Tl, Fas3, fra
neuron projection guidance	58 of 856 genes, 6.8%	295 of 16085 genes, 1.8%	5.85e-15	0.00%	0.00	fz2, sbb, lola, beat-VI, vvl, kuz, jing, gukh, sas, FER, Ptp61F, beat-IIa, DAAM, grn, beat-Vc, mew, egh, Ten-m, Trim9, jbug, Pka-R2, robo3, if, Ten-a, CadN2, InR, pdm3, Ptp99A, ap, Abl, drl, Lim3, Unc-115a, Dad, nerfin-1, Drl-2, RhoGEF64C, Src64B, en, DIP-gamma, Nrg, beat-Ic, dsx, beat-IIIb, Sema2a, Mmp2, trol, ko, beat-Va, cher, Src42A, beat-Ib, Fas3, beat-IIb, fra, stan, dysc, ckn
cell fate commitment	61 of 856 genes, 7.1%	326 of 16085 genes, 2.0%	9.60e-15	0.00%	0.00	hth, lola, Sara, boss, wake, kuz, abd-A, caup, ara, Ser, S, mam, BicD, Dhc64C, EcR, neur, egh, osa, pnt, melt, nej, bun, pyr, ey, ed, inv, dlg1, kay, par-1, lab, ap, hh, bdg, l(2)gl, Patronin, Dr, csw, Src64B, ush, en, ths, rl, uif, RasGAP1, vn, amon, Mmp2, cdi, Blimp-1, Antp, klu, alph, CtBP, msi, loco, Src42A, elB, stau, stan, pyd, nkd
growth	72 of 856 genes, 8.4%	447 of 16085 genes, 2.8%	4.71e-14	0.00%	0.00	fz2, sbb, Sh, Sesn, Nlg1, kuz, jing, caup, Sxl, stai, Hs6st, crb, hid, Ten-a, cno, Hcf, poe, disco-r, hh, l(2)gl, nvd, ex, rut, Nrg, pum, Mbs, rgn, sNPF-R, Src42A, stau, stan, dysc, futsch, ft, ara, FER, Hr4, kibra, foxo, al, Rok, DAAM, Ten-m, Nrx-1, Prosap, chrb, Tao, bun, Mmp1, DNApol-epsilon255, ey, CadN2, dlg1, step, InR, kay, par-1, spri, sima, hppy, Hipk, slo, Abl, cac, Dad, cic, wnd, Src64B, sfl, Sema2a, Tl, pip
regulation of developmental process	102 of 856 genes, 11.9%	787 of 16085 genes, 4.9%	6.05e-14	0.00%	0.00	fz2, sbb, lola, ec, vvl, kuz, abd-A, nau, stl, eIF4EHP, bab2, osa, pnt, Trim9, Hs6st, crb, pyr, hid, smog, Mef2, cno, dia, dsf, hh, l(2)gl, Sox21a, cv-c, Eip75B, Dr, csw, ex, ush, pum, Mbs, RasGAP1, Rme-8, Antp, alph, sNPF-R, tyn, Src42A, stau, stan, ds, dysc, cta, hth, futsch, ft, Ser, FER, e, Hr4, Dhc64C, kibra, foxo, shn, EcR, Tie, Rok, DAAM, neur, Prosap, bun, Tao, ed, ey, dlg1, step, InR, kay, par-1, spri, Hipk, tinc, slo, Abl, hbs, cac, Dad, PDZ-GEF, RyR, sd, nerfin-1, RhoBTB, Stat92E, Src64B, sfl, Sema2a, Syt1, kirre, rl, vn, cdi, klu, cher, Tl, pip, fra, pyd, unk, tral
pattern specification process	82 of 856 genes, 9.6%	559 of 16085 genes, 3.5%	7.92e-14	0.00%	0.00	fz2, Parp, sbb, boss, dome, ec, TfAP-2, vvl, jing, abd-A, caup, lilli, milt, CalpA, CG5890, osa, pnt, Dys, crb, corto, ltl, cv-2, inv, cno, lab, ap, jvl, hh, l(2)gl, Dr, csw, pum, uif, RasGAP1, Antp, CtBP, tara, stau, stan, ds, hth, ft, Doa, cora, spir, ara, Ser, S, BicD, shn, Dhc64C, EcR, Rok, mew, neur, app, ed, CadN2, dlg1, step, par-1, heph, f, CG8405, Dad, cic, Stat92E, en, fs(1)h, sfl, Rbfox1, rl, Syt1, vn, klu, loco, Tl, pip, fra, Lim1, tral, nkd
developmental growth	64 of 856 genes, 7.5%	379 of 16085 genes, 2.4%	2.92e-13	0.00%	0.00	fz2, sbb, Sh, futsch, ft, Nlg1, kuz, jing, FER, Hr4, Sxl, foxo, kibra, al, Rok, stai, DAAM, Ten-m, Nrx-1, Prosap, Hs6st, crb, bun, Tao, DNApol-epsilon255, hid, Mmp1, ey, Ten-a, CadN2, step, dlg1, kay, InR, cno, par-1, spri, poe, Hipk, slo, Abl, disco-r, hh, cac, l(2)gl, Dad, nvd, wnd, ex, Src64B, rut, Nrg, sfl, pum, Sema2a, Mbs, rgn, sNPF-R, Tl, Src42A, stau, pip, stan, dysc
regulation of multicellular organismal	86 of 856 genes, 10.0%	617 of 16085 genes, 3.8%	3.07e-13	0.00%	0.00	sbb, lola, ec, vvl, kuz, abd-A, nau, stl, eIF4EHP, osa, pnt, Trim9, Hs6st, crb, pyr, hid, smog, Mef2, cno, dia, hh, l(2)gl, cv-c,

development						Eip75B, csw, ex, pum, Mbs, RasGAP1, Rme-8, Antp, alph, tyn, Src42A, stau, stan, ds, dysc, cta, hth, futsch, ft, Ser, e, FER, Hr4, kibra, shn, Dhc64C, EcR, Tie, Rok, DAAM, neur, Prosap, bun, ed, dlg1, InR, kay, par-1, spri, tinc, slo, Abl, hbs, cac, Dad, PDZ-GEF, nerfin-1, sd, Src64B, sfl, Sema2a, kirre, rl, Syt1, vn, cdi, klu, cher, Tl, pyd, fra, unk, tral
regulation of response to stimulus	114 of 856 genes, 13.3%	956 of 16085 genes, 5.9%	3.58e-13	0.00%	0.00	Parp, lola, Smr, Sesn, CG34393, vvl, kuz, Fs, PVRAP, Camta, CG42674, rdgA, Sxl, Rev1, pnt, melt, TyrR, tefu, LRR, rdgC, crb, hid, ltl, smog, cv-2, Sytbeta, cno, Usp10, dia, Oct-TyrR, Gprk2, sra, l(2)gl, CG7094, wdb, cv-c, Snoo, ex, ush, rut, CG42684, pum, uif, RasGAP1, spz3, Mmp2, trol, CG15611, alph, sNPF-R, CtBP, CG30456, Src42A, stan, ds, CG43658, Cbp53E, ft, Doa, ken, S, kek5, Ptp61F, Hr4, PsGEF, kibra, foxo, Tie, Rok, neur, nej, chrb, Prosap, Tao, ey, ed, Pura, step, dlg1, Oamb, InR, kay, par-1, spri, hppy, sima, siz, Hipk, CG8405, hbs, Cdep, Syt7, Dad, Pde8, lncRNA:acal, crol, wnd, Stat92E, RhoGEF64C, Src64B, Dop1R2, Sema2a, Syt1, rl, vn, cdi, CG32683, Tl, loco, pip, fra, pyd, Gprk1, nkd
eye development	71 of 856 genes, 8.3%	455 of 16085 genes, 2.8%	4.51e-13	0.00%	0.00	lola, boss, dome, ec, caup, lilli, mbl, CG5921, pnt, melt, Trim9, crb, sns, hid, cno, hh, Awh, bdg, sano, csw, ex, ush, Mbs, RasGAP1, amon, alph, msi, Src42A, CG13251, stan, ds, dysc, hth, futsch, ft, Doa, ara, Ser, zen, S, kibra, foxo, shn, Rok, neur, Ten-m, nej, bun, ed, ey, CadN2, kay, siz, tinc, Hipk, Abl, hbs, cic, PDZ-GEF, sd, Stat92E, Src64B, kirre, rl, cdi, klu, elB, pyd, fra, Lim1, unk
sensory system development	71 of 856 genes, 8.3%	455 of 16085 genes, 2.8%	4.51e-13	0.00%	0.00	lola, boss, dome, ec, caup, lilli, mbl, CG5921, pnt, melt, Trim9, crb, sns, hid, cno, hh, Awh, bdg, sano, csw, ex, ush, Mbs, RasGAP1, amon, alph, msi, Src42A, CG13251, stan, ds, dysc, hth, futsch, ft, Doa, ara, Ser, zen, S, kibra, foxo, shn, Rok, neur, Ten-m, nej, bun, ed, ey, CadN2, kay, siz, tinc, Hipk, Abl, hbs, cic, PDZ-GEF, sd, Stat92E, Src64B, kirre, rl, cdi, klu, elB, pyd, fra, Lim1, unk
visual system development	71 of 856 genes, 8.3%	455 of 16085 genes, 2.8%	4.51e-13	0.00%	0.00	lola, boss, dome, ec, caup, lilli, mbl, CG5921, pnt, melt, Trim9, crb, sns, hid, cno, hh, Awh, bdg, sano, csw, ex, ush, Mbs, RasGAP1, amon, alph, msi, Src42A, CG13251, stan, ds, dysc, hth, futsch, ft, Doa, ara, Ser, zen, S, kibra, foxo, shn, Rok, neur, Ten-m, nej, bun, ed, ey, CadN2, kay, siz, tinc, Hipk, Abl, hbs, cic, PDZ-GEF, sd, Stat92E, Src64B, kirre, rl, cdi, klu, elB, pyd, fra, Lim1, unk
sensory organ development	79 of 856 genes, 9.2%	547 of 16085 genes, 3.4%	7.74e-13	0.00%	0.00	lola, boss, dome, ec, caup, lilli, mbl, CG5921, pnt, melt, Trim9, Hs6st, pwn, crb, sns, hid, cno, hh, Awh, bdg, l(2)gl, sano, csw, ex, ush, Mbs, uif, RasGAP1, amon, alph, CtBP, msi, Src42A, CG13251, stan, ds, dysc, hth, futsch, ft, Doa, ara, Ser, zen, S, kibra, foxo, shn, al, Rok, neur, Ten-m, nej, bun, ed, ey, CadN2, kay, siz, f, tinc, Hipk, Abl, hbs, cic, PDZ-GEF, sd, Stat92E, Src64B, dsx, kirre, rl, cdi, klu, elB, pyd, fra, Lim1, unk
compound eye development	67 of 856 genes, 7.8%	421 of 16085 genes, 2.6%	1.21e-12	0.00%	0.00	lola, boss, dome, ec, caup, lilli, mbl, pnt, melt, Trim9, crb, sns, hid, cno, hh, Awh, bdg, sano, csw, ex, ush, Mbs, RasGAP1, amon, alph, msi, Src42A, ds, stan, dysc,

						hth, futsch, ft, Doa, ara, zen, S, Ser, kibra, foxo, shn, Rok, neur, Ten-m, nej, bun, ed, ey, CadN2, kay, siz, Hipk, Abl, hbs, PDZ-GEF, sd, Stat92E, Src64B, kirre, rl, cdi, klu, elB, pyd, fra, unk, Lim1
muscle structure development	50 of 856 genes, 5.8%	255 of 16085 genes, 1.6%	1.53e-12	0.00%	0.00	hth, lola, vkg, kuz, abd-A, cora, nau, caup, ara, mbl, foxo, Rok, CalpA, PyK, DAAM, Scgdelta, mew, pnt, Dys, pyr, sns, ed, if, Mef2, Msp300, InR, CG43897, rost, siz, rhea, C3G, ap, lmd, CG42319, hbs, Tsp, drl, Dad, sd, Dr, Grip, Stat92E, ths, crp, Poxm, kirre, Bsg, Antp, cher, Tl
cell-cell adhesion	35 of 856 genes, 4.1%	130 of 16085 genes, 0.8%	1.55e-12	0.00%	0.00	beat-VI, ft, Nlg1, beat-IIa, mew, beat-Vc, egh, Ten-m, Nlg3, sns, ed, if, CadN2, Ten-a, dlg1, par-1, rhea, disco-r, hbs, Grip, Src64B, beat-IIIb, beat-Ic, Nrg, kirre, Bsg, beat-Va, Fas3, beat-Ib, beat-IIb, Src42A, ds, stan, pyd, beat-VII
regulation of signal transduction	95 of 856 genes, 11.1%	742 of 16085 genes, 4.6%	1.86e-12	0.00%	0.00	Parp, lola, Smr, Sesn, CG34393, kuz, Fs, PVRAP, Camta, CG42674, rdgA, Sxl, pnt, melt, TyrR, tefu, LRR, rdgC, crb, ltl, cv-2, cno, Usp10, Oct-TyrR, Gprk2, sra, l(2)gl, CG7094, wdb, cv-c, Snoo, ex, rut, CG42684, pum, uif, RasGAP1, spz3, Mmp2, trol, CG15611, alph, sNPF-R, CtBP, CG30456, Src42A, ds, CG43658, ft, Doa, ken, kek5, S, Ptp61F, Hr4, PsGEF, kibra, foxo, Rok, neur, nej, Prosap, chrb, Tao, ed, ey, Pura, dlg1, step, InR, kay, par-1, spri, hppy, siz, Hipk, hbs, Cdep, Dad, Pde8, lncRNA:acal, crol, wnd, RhoGEF64C, Src64B, vn, cdi, CG32683, Tl, loco, pip, fra, pyd, Gprk1, nkd
cell adhesion	45 of 856 genes, 5.3%	213 of 16085 genes, 1.3%	2.47e-12	0.00%	0.00	beat-VI, ft, Nlg1, cora, sas, beat-IIa, EcR, mew, beat-Vc, egh, Ten-m, alpha-Cat, Ccn, Nlg3, sns, Mmp1, ed, if, CadN2, Ten-a, dlg1, par-1, rhea, disco-r, hbs, Tsp, crol, Grip, Src64B, Nrg, beat-IIIb, beat-Ic, kirre, Bsg, beat-Va, tyn, Src42A, beat-Ib, Fas3, beat-IIb, CG5758, ds, stan, pyd, beat-VII
negative regulation of cellular process	139 of 856 genes, 16.2%	1314 of 16085 genes, 8.2%	2.94e-12	0.00%	0.00	sbb, Sesn, jing, eIF4EHP, Sxl, osa, CG12605, crb, AGO3, Usp10, scrt, Tlk, Gprk2, CG7094, Sox21a, cv-c, Eip75B, Snoo, Kank, Fancm, CG8312, CG42684, pum, uif, Mmp2, trol, Blimp-1, alph, msi, Src42A, Eip78C, timeout, Tis11, ds, dysc, ft, Doa, sas, Ser, Ptp61F, shn, foxo, neur, per, bun, ed, InR, hppy, sima, CG11247, CG12344, RyR, nerfin-1, bin3, Stat92E, Src64B, dsx, Sema2a, chinmo, cdi, klu, Lim1, tral, nkd, Smr, TfAP-2, Fs, abd-A, Duox, stl, Grd, pnt, Ccn, LRR, tefu, corto, ltl, rhea, lmd, ich, disco-r, Debcl, hh, l(2)gl, Oaz, wdb, bol, csw, ex, ush, RasGAP1, Antp, Xrp1, CtBP, bru3, trp, futsch, CG4238, e, kek5, ken, Hr4, kibra, Tie, EcR, al, e(y)3, nej, upSET, chrb, Maf1, Oamb, dlg1, smg, par-1, heph, Hipk, slo, Abl, gpp, Patronin, cic, Dad, Pde8, lncRNA:acal, sd, crol, bru2, fs(1)h, en, rl, CG9932, Rbfox1, tut, CG32683, cher, Tl, pyd, Gprk1
regionalization	75 of 856 genes, 8.8%	518 of 16085 genes, 3.2%	4.01e-12	0.00%	0.00	fz2, Parp, sbb, boss, dome, TfAP-2, vvl, jing, abd-A, caup, lilli, milt, CalpA, CG5890, osa, pnt, Dys, crb, corto, ltl, cv-2, inv, lab, ap, jvl, hh, l(2)gl, Dr, csw, pum, uif, RasGAP1, Antp, CtBP, tara, stau, ds, stan, hth, ft, Doa, cora, spir, ara, Ser, S, BicD, shn, Dhc64C, EcR, mew, neur, dlg1, step, par-1, f, CG8405, cic, Dad, Stat92E, en, fs(1)h, sfl, Rbfox1, rl, Syt1, vn, klu, loco, Tl, pip, fra, Lim1, tral, nkd

behavior	88 of 856 genes, 10.3%	673 of 16085 genes, 4.2%	6.80e-12	0.00%	0.00	sbb, Sh, Pino, DCX-EMAP, Sema5c, dome, TfAP-2, lilli, mbl, Obp56d, dpr9, dpr1, CalpA, egh, Trim9, hang, Mef2, lncRNA:flam, Sap47, dsf, sra, drl, Pde1c, bol, rut, Nrg, pum, Mbs, amon, sNPF-R, tara, stau, dysc, Or67d, trp, futsch, wake, slgA, e, S, nemy, shep, Gem3, nompC, foxo, Hk, EcR, Tob, neur, per, bgm, Nrx-1, nej, Pka-R2, Shab, ey, Pura, Oamb, dlg1, InR, kay, Drip, hppy, slo, Abl, Rh7, cac, CCKLR-17D1, wnd, Stat92E, 5-HT7, Src64B, Ac3, Btk29A, Gr28b, dsx, Dop1R2, Rbfox1, Sema2a, Syt1, rl, vn, klu, Orco, cher, unc80, Gbs-70E, ogre
cell surface receptor signaling pathway	86 of 856 genes, 10.0%	656 of 16085 genes, 4.1%	1.24e-11	0.00%	0.00	fz2, Parp, lola, Smr, boss, dome, CG31183, kuz, Fs, PVRAP, Duox, Sxl, osa, pnt, melt, Trim9, crb, pyr, ltl, cv-2, Usp10, Gprk2, Debcl, hh, drl, l(2)gl, CG7094, wdb, Snoo, Drl-2, csw, pum, uif, RasGAP1, spz3, Dh31-R, Mmp2, trol, CG32447, alph, CtBP, Src42A, stan, ckn, Doa, Ser, kek5, mam, S, FER, Ptp61F, foxo, shn, Tie, Rok, CG34357, neur, nej, Prosap, ed, ey, dlg1, step, InR, kay, wry, hppy, sima, Hipk, hbs, Dad, cic, crol, Src64B, ths, sfl, Sema2a, rl, vn, cdi, Tl, pip, pyd, fra, Gprk1, nkd
biological adhesion	45 of 856 genes, 5.3%	222 of 16085 genes, 1.4%	1.25e-11	0.00%	0.00	beat-VI, ft, Nlg1, cora, sas, beat-IIa, EcR, mew, beat-Vc, egh, Ten-m, alpha-Cat, Ccn, Nlg3, sns, Mmp1, ed, if, CadN2, Ten-a, dlg1, par-1, rhea, disco-r, hbs, Tsp, crol, Grip, Src64B, Nrg, beat-IIIb, beat-Ic, kirre, Bsg, beat-Va, tyn, Src42A, beat-Ib, Fas3, beat-IIb, CG5758, ds, stan, pyd, beat-VII
eye morphogenesis	60 of 856 genes, 7.0%	369 of 16085 genes, 2.3%	1.62e-11	0.00%	0.00	hth, lola, boss, dome, ft, ec, Doa, caup, ara, lilli, mbl, Ser, S, CG5921, shn, foxo, kibra, Rok, neur, Ten-m, pnt, melt, nej, crb, bun, sns, hid, ey, ed, CadN2, kay, cno, siz, tinc, hbs, hh, bdg, cic, sano, PDZ-GEF, sd, csw, ex, Src64B, rl, Mbs, kirre, RasGAP1, amon, cdi, klu, alph, msi, Src42A, elB, CG13251, pyd, ds, stan, dysc
sensory organ morphogenesis	60 of 856 genes, 7.0%	369 of 16085 genes, 2.3%	1.62e-11	0.00%	0.00	hth, lola, boss, dome, ft, ec, Doa, caup, ara, lilli, mbl, Ser, S, CG5921, shn, foxo, kibra, Rok, neur, Ten-m, pnt, melt, nej, crb, bun, sns, hid, ey, ed, CadN2, kay, cno, siz, tinc, hbs, hh, bdg, cic, sano, PDZ-GEF, sd, csw, ex, Src64B, rl, Mbs, kirre, RasGAP1, amon, cdi, klu, alph, msi, Src42A, elB, CG13251, pyd, ds, stan, dysc
system process	88 of 856 genes, 10.3%	685 of 16085 genes, 4.3%	1.96e-11	0.00%	0.00	Sh, Gr22e, DCX-EMAP, boss, Npc1b, dome, Neurochondrin, CG9492, dpr12, lilli, dpr8, nAChRalpha2, rdgA, CG5921, Obp56d, dpr9, dpr1, Grd, Ggamma30A, rdgC, sns, if, Sap47, Gr23a, dia, Nha2, Oct-TyrR, sra, drl, dpr6, KCNQ, rut, pum, Ac78C, axo, stau, dpr13, dysc, Or67d, trp, futsch, nAChRalpha6, cora, pHCl-1, MRP, nemy, nompC, EcR, CG2121, Scgdelta, Tob, mew, neur, Ir40a, per, Nrx-1, ATP8B, Prosap, Ca-alpha1T, Shab, Oamb, pdm3, wry, Drip, nAChRbeta2, Mvl, f, Abl, Mdr50, Dhc36C, Rh7, cac, CG12344, RyR, dpr2, CG8086, wnd, Stat92E, Src64B, Btk29A, Gr28b, Rbfox1, kirre, vn, Orco, cher, loco, SKIP
embryo development	82 of 856 genes, 9.6%	615 of 16085 genes, 3.8%	2.16e-11	0.00%	0.00	fz2, vkg, Npc1b, Sema5c, dome, jing, abd-A, lilli, luna, pnt, alpha-Cat, crb, pyr, sns, hid, smog, if, cno, T48, CG41099, rhea, dia, Tlk, Gprk2, hh, l(2)gl, cv-c, Dr, KCNQ, csw, ush, pum, Mbs, RasGAP1, Mmp2, Antp, alph, tyn, Src42A, stau, stan, cta, hth,

						Doa, cora, spir, zen, mam, S, FER, Ptp61F, shn, EcR, Rok, toc, chrb, Mmp1, ed, dlg1, step, InR, kay, par-1, Abl, Dad, cic, lncRNA:acal, PDZ-GEF, Stat92E, en, fs(1)h, Btk29A, ths, sfl, kirre, rl, Syt1, loco, Tl, pip, pyd, nkd
negative regulation of biological process	152 of 856 genes, 17.8%	1536 of 16085 genes, 9.5%	3.47e-11	0.00%	0.00	sbb, Sesn, jing, caup, eIF4EHP, Sxl, osa, CG12605, crb, AGO3, Usp10, scrt, Tlk, Gprk2, CG7094, Sox21a, cv-c, Eip75B, Snoo, Kank, Fancm, CG8312, CG42684, pum, uif, Mmp2, trol, Blimp-1, alph, msi, Src42A, Eip78C, timeout, Tis11, ds, dysc, ft, Doa, sas, ara, Ser, Ptp61F, shn, foxo, neur, per, bun, Tao, ed, InR, hppy, sima, CG11247, CG12344, RyR, Achl, nerfin-1, bin3, Stat92E, Src64B, CG6701, dsx, Sema2a, chinmo, cdi, klu, elB, Lim1, tral, nkd, Smr, TfAP-2, Fs, abd-A, Duox, stl, Grd, pnt, melt, Ccn, LRR, tefu, corto, Pde6, ltl, rhea, lmd, ich, disco-r, Debcl, hh, l(2)gl, Oaz, wdb, bol, Dr, csw, ex, ush, RasGAP1, Antp, Xrp1, CtBP, bru3, trp, futsch, CG11486, Cbp53E, CG4238, e, kek5, ken, Hr4, kibra, Tie, EcR, al, Rok, e(y)3, nej, upSET, chrb, Maf1, Oamb, dlg1, par-1, smg, heph, Hipk, slo, Abl, gpp, Patronin, cic, Dad, Pde8, lncRNA:acal, sd, crol, bru2, fs(1)h, en, Dop1R2, rl, CG9932, Rbfox1, tut, CG32683, cher, Tl, pyd, Gprk1
epithelial cell differentiation	66 of 856 genes, 7.7%	440 of 16085 genes, 2.7%	3.91e-11	0.00%	0.00	Parp, Smr, dome, kuz, jing, Fs(2)Ket, stl, stai, egh, alpha-Cat, crb, smog, if, Mef2, lncRNA:flam, CG41099, rhea, dia, jvl, Gprk2, hh, Sox21a, csw, ex, ush, Mbs, Rme-8, tyn, bbg, ds, cta, ft, qua, spir, cora, mam, S, kibra, Dhc64C, EcR, Tie, Rok, toc, neur, nej, app, Tao, bun, ed, dlg1, InR, kay, spri, par-1, f, Abl, hbs, cic, Stat92E, Src64B, RecQ4, rl, vn, cher, loco, Fas3
regulation of cellular macromolecule biosynthetic process	136 of 856 genes, 15.9%	1317 of 16085 genes, 8.2%	4.33e-11	0.00%	0.00	sbb, CG10185, jing, caup, lilli, Camta, dpy, tna, eIF4EHP, Sxl, luna, osa, Atf6, CG12605, CG12054, Mef2, inv, lab, CG10947, Hcf, Usp10, dsf, scrt, ap, Tlk, Awh, Spt3, Sox21a, Eip75B, Snoo, hng3, crp, CG8312, pum, mamo, Blimp-1, stv, msi, Eip78C, stau, timeout, Tis11, hth, ara, mam, shn, foxo, CG5694, grn, per, CG3726, CG17514, bun, Spt20, opa, pdm3, kay, sima, Rx, CG12769, CG11247, nerfin-1, bin3, Stat92E, dsx, chinmo, klu, SCAP, fra, Lim1, Parp, MBD-R2, lola, Smr, TfAP-2, Fs, vvl, abd-A, nau, MED14, bab2, Eip93F, pnt, melt, tefu, corto, ltl, cv-2, rhea, lmd, ich, Oaz, bol, Dr, ush, Poxm, Antp, ko, CtBP, tara, bru3, CG1815, Tet, RunxB, zen, kek5, ken, Hr4, kibra, EcR, al, e(y)3, nej, upSET, Maf1, ey, CG31612, Oamb, dlg1, smg, heph, gpp, Lim3, cic, Dad, sd, crol, bru2, RecQ4, fs(1)h, en, rl, CG9932, Rbfox1, tut, Tl
regulation of macromolecule biosynthetic process	136 of 856 genes, 15.9%	1323 of 16085 genes, 8.2%	6.18e-11	0.00%	0.00	sbb, CG10185, jing, caup, lilli, Camta, dpy, tna, eIF4EHP, Sxl, luna, osa, Atf6, CG12605, CG12054, Mef2, inv, lab, CG10947, Hcf, Usp10, dsf, scrt, ap, Tlk, Awh, Spt3, Sox21a, Eip75B, Snoo, hng3, crp, CG8312, pum, mamo, Blimp-1, stv, msi, Eip78C, stau, timeout, Tis11, hth, ara, mam, shn, foxo, CG5694, grn, per, CG3726, CG17514, bun, Spt20, opa, pdm3, kay, sima, Rx, CG12769, CG11247, nerfin-1, bin3, Stat92E, dsx, chinmo, klu, SCAP, fra, Lim1, Parp, MBD-R2, lola, Smr, TfAP-2, Fs, vvl, abd-A, nau, MED14, bab2, Eip93F, pnt, melt, tefu, corto, ltl, cv-2, rhea,

						lmd, ich, Oaz, bol, Dr, ush, Poxm, Antp, ko, CtBP, tara, bru3, CG1815, Tet, RunxB, zen, kek5, ken, Hr4, kibra, EcR, al, e(y)3, nej, upSET, Maf1, ey, CG31612, Oamb, dlg1, smg, heph, gpp, Lim3, cic, Dad, sd, crol, bru2, RecQ4, fs(1)h, en, rl, CG9932, Rbfox1, tut, Tl
heterophilic cell-cell adhesion via plasma membrane cell adhesion molecules	16 of 856 genes, 1.9%	27 of 16085 genes, 0.2%	6.71e-11	0.00%	0.00	beat-VI, ft, hbs, beat-IIa, mew, beat-Vc, beat-IIIb, beat-Ic, kirre, beat-Va, beat-Ib, sns, beat-IIb, ds, if, beat-VII
embryonic morphogenesis	48 of 856 genes, 5.6%	262 of 16085 genes, 1.6%	9.11e-11	0.00%	0.00	vkg, Npc1b, abd-A, cora, FER, S, mam, zen, Ptp61F, shn, EcR, Rok, pnt, alpha-Cat, chrb, crb, pyr, hid, Mmp1, smog, ed, step, dlg1, cno, InR, kay, T48, CG41099, rhea, Gprk2, Abl, l(2)gl, Dad, lncRNA:acal, PDZ-GEF, cv-c, Dr, ush, Btk29A, ths, sfl, pum, Mbs, Mmp2, alph, Src42A, pyd, cta
regulation of cellular biosynthetic process	139 of 856 genes, 16.2%	1375 of 16085 genes, 8.5%	1.14e-10	0.00%	0.00	sbb, CG10185, jing, caup, lilli, Camta, dpy, tna, eIF4EHP, Sxl, luna, osa, Atf6, CG12605, CG12054, Mef2, inv, lab, CG10947, Hcf, Usp10, dsf, scrt, ap, Tlk, Gprk2, Awh, Spt3, Sox21a, Eip75B, Snoo, hng3, crp, CG8312, pum, mamo, Blimp-1, stv, msi, Eip78C, stau, timeout, Tis11, hth, ara, mam, shn, foxo, CG5694, grn, per, CG3726, CG17514, bun, Spt20, opa, pdm3, kay, sima, Rx, CG12769, CG11247, nerfin-1, bin3, Stat92E, dsx, chinmo, klu, SCAP, fra, Lim1, Parp, MBD-R2, lola, Smr, TfAP-2, Fs, vvl, abd-A, nau, MED14, Eip93F, bab2, pnt, melt, tefu, corto, smog, ltl, cv-2, rhea, lmd, ich, Oaz, bol, Dr, ush, Poxm, Antp, ko, CtBP, tara, bru3, CG1815, Tet, RunxB, zen, e, kek5, ken, Hr4, kibra, EcR, al, e(y)3, nej, upSET, Maf1, ey, CG31612, Oamb, dlg1, smg, heph, gpp, Lim3, cic, Dad, sd, crol, bru2, RecQ4, fs(1)h, en, rl, CG9932, Rbfox1, tut, Tl
regulation of biosynthetic process	139 of 856 genes, 16.2%	1379 of 16085 genes, 8.6%	1.43e-10	0.00%	0.00	sbb, CG10185, jing, caup, lilli, Camta, dpy, tna, eIF4EHP, Sxl, luna, osa, Atf6, CG12605, CG12054, Mef2, inv, lab, CG10947, Hcf, Usp10, dsf, scrt, ap, Tlk, Gprk2, Awh, Spt3, Sox21a, Eip75B, Snoo, hng3, crp, CG8312, pum, mamo, Blimp-1, stv, msi, Eip78C, stau, timeout, Tis11, hth, ara, mam, shn, foxo, CG5694, grn, per, CG3726, CG17514, bun, Spt20, opa, pdm3, kay, sima, Rx, CG12769, CG11247, nerfin-1, bin3, Stat92E, dsx, chinmo, klu, SCAP, fra, Lim1, Parp, MBD-R2, lola, Smr, TfAP-2, Fs, vvl, abd-A, nau, MED14, Eip93F, bab2, pnt, melt, tefu, corto, smog, ltl, cv-2, rhea, lmd, ich, Oaz, bol, Dr, ush, Poxm, Antp, ko, CtBP, tara, bru3, CG1815, Tet, RunxB, zen, e, kek5, ken, Hr4, kibra, EcR, al, e(y)3, nej, upSET, Maf1, ey, CG31612, Oamb, dlg1, smg, heph, gpp, Lim3, cic, Dad, sd, crol, bru2, RecQ4, fs(1)h, en, rl, CG9932, Rbfox1, tut, Tl
compound eye morphogenesis	56 of 856 genes, 6.5%	347 of 16085 genes, 2.2%	1.85e-10	0.00%	0.00	hth, lola, boss, dome, ft, ec, Doa, caup, ara, lilli, mbl, Ser, S, shn, foxo, kibra, Rok, neur, Ten-m, pnt, melt, nej, crb, bun, sns, hid, ey, ed, CadN2, kay, cno, siz, hbs, hh, bdg, sano, PDZ-GEF, sd, csw, ex, Src64B, rl, kirre, Mbs, RasGAP1, amon, cdi, klu, alph, msi, Src42A, elB, ds, stan, pyd, dysc
regulation of nucleobase-containing compound metabolic process	135 of 856 genes, 15.8%	1336 of 16085 genes, 8.3%	2.94e-10	0.00%	0.00	sbb, CG10185, jing, caup, lilli, Camta, dpy, tna, Sxl, luna, osa, l(3)72Ab, Atf6, CG12605, CG12054, AGO3, Mef2, inv, lab, Hcf, Usp10, dsf, scrt, ap, Tlk, Awh, Spt3, Sox21a, Eip75B, Snoo, Fancm, hng3, crp,

						CG8312, pum, mamo, Blimp-1, Eip78C, timeout, Tis11, hth, Doa, ara, mam, shn, foxo, CG5694, grn, per, CG3726, bun, Spt20, opa, pdm3, kay, sima, Rx, CG12769, CG11247, nerfin-1, bin3, Stat92E, dsx, chinmo, klu, SCAP, fra, Lim1, Parp, MBD-R2, lola, Smr, TfAP-2, Fs, vvl, abd-A, mbl, nau, MED14, bab2, Eip93F, Rev1, pnt, melt, tefu, corto, ltl, cv-2, rhea, lmd, ich, Oaz, bol, Dr, ush, Poxm, Antp, ko, CtBP, tara, bru3, CG1815, Tet, RunxB, zen, kek5, ken, Hr4, kibra, EcR, al, e(y)3, nej, upSET, Maf1, ey, CG31612, Oamb, dlg1, smg, ps, gpp, Lim3, cic, Dad, sd, crol, bru2, RecQ4, fs(1)h, en, rl, CG9932, Rbfox1, Tl
regulation of nitrogen compound metabolic process	172 of 856 genes, 20.1%	1870 of 16085 genes, 11.6%	3.54e-10	0.00%	0.00	sbb, CG10185, jing, caup, lilli, Camta, dpy, tna, eIF4EHP, Sxl, luna, osa, l(3)72Ab, CG33144, Atf6, CG12605, CG12054, crb, AGO3, hid, Mef2, inv, cno, lab, CG10947, Hcf, Usp10, dsf, scrt, ap, Tlk, Gprk2, Awh, Spt3, Sox21a, Eip75B, Snoo, Fancm, hng3, crp, CG8312, pum, mamo, Blimp-1, stv, alph, msi, Src42A, Eip78C, stau, timeout, Tis11, hth, Doa, ara, mam, Ptp61F, shn, foxo, CG5694, grn, per, CG3726, CG17514, Pka-R2, bun, Tao, Spt20, opa, pdm3, InR, kay, hppy, sima, Rx, CG12769, CG11247, nerfin-1, wnd, bin3, Stat92E, Src64B, dsx, chinmo, klu, SCAP, pip, fra, Lim1, Parp, MBD-R2, lola, Smr, TfAP-2, Fs, vvl, abd-A, mbl, nau, MED14, Eip93F, bab2, Rev1, pnt, melt, tefu, corto, smog, ltl, cv-2, rhea, lmd, ich, hh, Oaz, l(2)gl, wdb, bol, Dr, ush, Poxm, Antp, ko, sNPF-R, CtBP, tara, bru3, CG1815, Tet, RunxB, CG4238, ken, zen, kek5, Hr4, kibra, al, EcR, Rok, e(y)3, nej, upSET, Maf1, ey, CG31612, Oamb, dlg1, step, par-1, smg, heph, CG8405, hbs, ps, Lim3, gpp, cic, Dad, Pde8, lncRNA:acal, crol, sd, bru2, RecQ4, en, fs(1)h, Rbfox1, CG9932, rl, vn, tut, Tl
intracellular signal transduction	83 of 856 genes, 9.7%	661 of 16085 genes, 4.1%	4.35e-10	0.00%	0.00	DCX-EMAP, Gyc88E, dome, CG31183, Sesn, CG34393, PVRAP, Dgk, CG42674, rdgA, melt, TyrR, tefu, LRR, rdgC, crb, hid, hang, cno, C3G, Oct-TyrR, Tlk, Gprk2, sra, Debcl, wdb, cv-c, ex, rut, CG42684, RasGAP1, CG34384, CG15611, alph, sNPF-R, Ac78C, CG30456, Src42A, S6KL, ds, cta, CG43658, ft, sff, ken, Ptp61F, PsGEF, kibra, Rok, CG34357, ACXC, chrb, Pka-R2, Tao, ed, Pura, dlg1, step, CG4629, InR, par-1, spri, hppy, siz, Hipk, Cdep, cac, Pde8, lncRNA:acal, PDZ-GEF, sd, RhoBTB, wnd, Stat92E, RhoGEF64C, Src64B, 5-HT7, Ac3, Btk29A, Dop1R2, rl, vn, Cngl
photoreceptor cell differentiation	43 of 856 genes, 5.0%	226 of 16085 genes, 1.4%	5.18e-10	0.00%	0.00	hth, lola, boss, Doa, lilli, mbl, S, CG5921, EcR, neur, Ten-m, osa, pnt, melt, nej, crb, bun, ey, ed, CadN2, kay, tinc, hbs, hh, bdg, PDZ-GEF, csw, Src64B, ex, Mbs, rl, RasGAP1, Bsg, amon, cdi, klu, alph, msi, Src42A, CG13251, elB, stan, dysc
establishment or maintenance of cell polarity	42 of 856 genes, 4.9%	219 of 16085 genes, 1.4%	7.48e-10	0.00%	0.00	Parp, lola, ft, ec, wake, cora, sff, caup, ara, S, BicD, Dhc64C, Rok, egh, pnt, alpha-Cat, Dys, nej, crb, smog, ed, CadN2, dlg1, kay, cno, par-1, heph, jvl, hbs, bdg, l(2)gl, sano, cv-c, RhoBTB, Grip, csw, trol, loco, htt, ds, stan, cta
regulation of RNA metabolic process	129 of 856 genes, 15.1%	1272 of 16085 genes, 7.9%	8.80e-10	0.00%	0.00	sbb, jing, caup, lilli, Camta, dpy, tna, Sxl, luna, osa, l(3)72Ab, Atf6, CG12605,

						CG12054, AGO3, Mef2, inv, lab, Hcf, Usp10, dsf, scrt, ap, Tlk, Awh, Spt3, Sox21a, Eip75B, Snoo, hng3, crp, CG8312, pum, mamo, Blimp-1, Eip78C, Tis11, hth, Doa, ara, mam, shn, foxo, CG5694, grn, per, CG3726, bun, Spt20, opa, pdm3, kay, sima, Rx, CG12769, CG11247, nerfin-1, bin3, Stat92E, dsx, chinmo, klu, SCAP, fra, Lim1, Parp, MBD-R2, lola, Smr, TfAP-2, Fs, vvl, abd-A, mbl, nau, MED14, bab2, Eip93F, pnt, melt, tefu, corto, ltl, cv-2, rhea, lmd, ich, Oaz, bol, Dr, ush, Poxm, ko, Antp, CtBP, tara, bru3, CG1815, Tet, RunxB, zen, kek5, ken, Hr4, kibra, EcR, al, e(y)3, nej, upSET, Maf1, ey, Oamb, dlg1, smg, ps, gpp, Lim3, cic, Dad, sd, crol, bru2, fs(1)h, en, rl, CG9932, Rbfox1, Tl
cell-cell signaling	69 of 856 genes, 8.1%	505 of 16085 genes, 3.1%	1.01e-09	0.00%	0.00	fz2, Sh, Nlg1, Fife, nau, nAChRalpha2, Grd, osa, Gie, Dys, Nlg3, Ten-a, Sytbeta, GluRIB, Sap47, dtr, hh, drl, l(2)gl, CG7094, cv-c, Grip, Drl-2, rut, Rph, pum, unc-13-4A, X11Lbeta, Mmp2, CG32447, CtBP, Src42A, Snap25, dysc, kek6, Cbp53E, nAChRalpha6, GluRIA, pHCl-1, Ser, mam, nemy, BicD, Mctp, Rok, Ten-m, Nrx-1, nej, Prosap, Shab, dlg1, Oamb, InR, nAChRbeta2, Hipk, KaiR1D, slo, mAChR-B, Abl, Syt7, cac, CG12344, crol, Src64B, 5-HT7, sfl, rl, Syt1, nkd
chemical synaptic transmission	52 of 856 genes, 6.1%	321 of 16085 genes, 2.0%	1.27e-09	0.00%	0.00	kek6, Sh, Cbp53E, GluRIA, nAChRalpha6, Nlg1, Fife, pHCl-1, nAChRalpha2, nemy, BicD, Mctp, Grd, Ten-m, Gie, Dys, nej, Nrx-1, Prosap, Nlg3, Shab, Ten-a, dlg1, Oamb, Sytbeta, GluRIB, Sap47, nAChRbeta2, dtr, mAChR-B, slo, KaiR1D, Abl, Syt7, cac, l(2)gl, CG12344, cv-c, Grip, 5-HT7, Src64B, rut, Rph, pum, Syt1, rl, unc-13-4A, X11Lbeta, CG32447, Src42A, Snap25, dysc
anterograde trans-synaptic signaling	52 of 856 genes, 6.1%	321 of 16085 genes, 2.0%	1.27e-09	0.00%	0.00	kek6, Sh, Cbp53E, GluRIA, nAChRalpha6, Nlg1, Fife, pHCl-1, nAChRalpha2, nemy, BicD, Mctp, Grd, Ten-m, Gie, Dys, nej, Nrx-1, Prosap, Nlg3, Shab, Ten-a, dlg1, Oamb, Sytbeta, GluRIB, Sap47, nAChRbeta2, dtr, mAChR-B, slo, KaiR1D, Abl, Syt7, cac, l(2)gl, CG12344, cv-c, Grip, 5-HT7, Src64B, rut, Rph, pum, Syt1, rl, unc-13-4A, X11Lbeta, CG32447, Src42A, Snap25, dysc
regulation of transcription, DNA-templated	119 of 856 genes, 13.9%	1143 of 16085 genes, 7.1%	1.42e-09	0.00%	0.00	MBD-R2, Parp, sbb, lola, Smr, TfAP-2, vvl, jing, Fs, abd-A, nau, caup, lilli, Camta, dpy, MED14, tna, Eip93F, bab2, luna, osa, pnt, Atf6, melt, CG12605, tefu, CG12054, corto, ltl, cv-2, Mef2, inv, lab, Hcf, Usp10, rhea, dsf, scrt, ap, lmd, ich, Tlk, Awh, Spt3, Oaz, Sox21a, Eip75B, Dr, Snoo, ush, hng3, crp, CG8312, Poxm, pum, mamo, Blimp-1, Antp, ko, CtBP, tara, Eip78C, hth, CG1815, Tet, RunxB, ara, ken, kek5, zen, mam, Hr4, shn, kibra, foxo, al, CG5694, EcR, grn, per, e(y)3, CG3726, nej, upSET, Maf1, bun, ey, Spt20, Oamb, dlg1, opa, pdm3, kay, smg, sima, Rx, CG12769, Lim3, gpp, CG11247, Dad, cic, crol, sd, nerfin-1, bin3, Stat92E, en, fs(1)h, dsx, Rbfox1, CG9932, chinmo, rl, klu, SCAP, Tl, fra, Lim1
regulation of nucleic acid-templated transcription	119 of 856 genes, 13.9%	1143 of 16085 genes, 7.1%	1.42e-09	0.00%	0.00	MBD-R2, Parp, sbb, lola, Smr, TfAP-2, vvl, jing, Fs, abd-A, nau, caup, lilli, Camta, dpy, MED14, tna, Eip93F, bab2, luna, osa, pnt, Atf6, melt, CG12605, tefu, CG12054, corto, ltl, cv-2, Mef2, inv, lab, Hcf, Usp10, rhea,

						dsf, scrt, ap, lmd, ich, Tlk, Awh, Spt3, Oaz, Sox21a, Eip75B, Dr, Snoo, ush, hng3, crp, CG8312, Poxm, pum, mamo, Blimp-1, Antp, ko, CtBP, tara, Eip78C, hth, CG1815, Tet, RunxB, ara, ken, kek5, zen, mam, Hr4, shn, kibra, foxo, al, CG5694, EcR, grn, per, e(y)3, CG3726, nej, upSET, Maf1, bun, ey, Spt20, Oamb, dlg1, opa, pdm3, kay, smg, sima, Rx, CG12769, Lim3, gpp, CG11247, Dad, cic, crol, sd, nerfin-1, bin3, Stat92E, en, fs(1)h, dsx, Rbfox1, CG9932, chinmo, rl, klu, SCAP, Tl, fra, Lim1
regulation of RNA biosynthetic process	119 of 856 genes, 13.9%	1143 of 16085 genes, 7.1%	1.42e-09	0.00%	0.00	MBD-R2, Parp, sbb, lola, Smr, TfAP-2, vvl, jing, Fs, abd-A, nau, caup, lilli, Camta, dpy, MED14, tna, Eip93F, bab2, luna, osa, pnt, Atf6, melt, CG12605, tefu, CG12054, corto, ltl, cv-2, Mef2, inv, lab, Hcf, Usp10, rhea, dsf, scrt, ap, lmd, ich, Tlk, Awh, Spt3, Oaz, Sox21a, Eip75B, Dr, Snoo, ush, hng3, crp, CG8312, Poxm, pum, mamo, Blimp-1, Antp, ko, CtBP, tara, Eip78C, hth, CG1815, Tet, RunxB, ara, ken, kek5, zen, mam, Hr4, shn, kibra, foxo, al, CG5694, EcR, grn, per, e(y)3, CG3726, nej, upSET, Maf1, bun, ey, Spt20, Oamb, dlg1, opa, pdm3, kay, smg, sima, Rx, CG12769, Lim3, gpp, CG11247, Dad, cic, crol, sd, nerfin-1, bin3, Stat92E, en, fs(1)h, dsx, Rbfox1, CG9932, chinmo, rl, klu, SCAP, Tl, fra, Lim1
trans-synaptic signaling	52 of 856 genes, 6.1%	322 of 16085 genes, 2.0%	1.44e-09	0.00%	0.00	kek6, Sh, Cbp53E, GluRIA, nAChRalpha6, Nlg1, Fife, pHCl-1, nAChRalpha2, nemy, BicD, Mctp, Grd, Ten-m, Gie, Dys, nej, Nrx-1, Prosap, Nlg3, Shab, Ten-a, dlg1, Oamb, Sytbeta, GluRIB, Sap47, nAChRbeta2, dtr, mAChR-B, slo, KaiR1D, Abl, Syt7, cac, l(2)gl, CG12344, cv-c, Grip, 5-HT7, Src64B, rut, Rph, pum, Syt1, rl, unc-13-4A, X11Lbeta, CG32447, Src42A, Snap25, dysc
regulation of cellular metabolic process	176 of 856 genes, 20.6%	1962 of 16085 genes, 12.2%	1.61e-09	0.00%	0.00	sbb, CG10185, Sesn, jing, caup, lilli, Camta, dpy, tna, eIF4EHP, Sxl, luna, osa, l(3)72Ab, CG33144, Atf6, CG12605, CG12054, crb, AGO3, hid, Mef2, inv, cno, lab, CG10947, Hcf, Usp10, dsf, scrt, ap, Tlk, Gprk2, Awh, Spt3, Sox21a, Eip75B, Snoo, Fancm, hng3, crp, CG8312, pum, mamo, Blimp-1, stv, alph, msi, Src42A, Eip78C, stau, timeout, Tis11, hth, Doa, ara, mam, Ptp61F, shn, foxo, CG5694, grn, per, CG3726, CG17514, Pka-R2, bun, Tao, Spt20, opa, pdm3, InR, kay, hppy, sima, Rx, CG12769, CG11247, nerfin-1, wnd, bin3, Stat92E, Src64B, dsx, chinmo, klu, SCAP, pip, fra, Lim1, Parp, MBD-R2, lola, Smr, TfAP-2, Fs, vvl, abd-A, mbl, nau, MED14, Eip93F, bab2, Rev1, pnt, melt, tefu, corto, smog, ltl, cv-2, rhea, lmd, ich, Debcl, hh, Oaz, l(2)gl, wdb, bol, Dr, ush, Poxm, Antp, ko, sNPF-R, CtBP, tara, bru3, CG1815, Tet, RunxB, ken, zen, e, kek5, Hr4, kibra, al, EcR, Rok, e(y)3, nej, upSET, Maf1, ey, CG31612, Oamb, dlg1, step, par-1, smg, heph, CG8405, hbs, ps, Lim3, gpp, cic, Dad, Pde8, lncRNA:acal, crol, sd, bru2, RecQ4, en, fs(1)h, Gyf, Rbfox1, CG9932, rl, vn, tut, Tl, Gbs-70E
synaptic signaling	52 of 856 genes, 6.1%	323 of 16085 genes, 2.0%	1.63e-09	0.00%	0.00	kek6, Sh, Cbp53E, GluRIA, nAChRalpha6, Nlg1, Fife, pHCl-1, nAChRalpha2, nemy, BicD, Mctp, Grd, Ten-m, Gie, Dys, nej, Nrx-1, Prosap, Nlg3, Shab, Ten-a, dlg1, Oamb, Sytbeta, GluRIB, Sap47, nAChRbeta2, dtr, mAChR-B, slo, KaiR1D, Abl, Syt7, cac, l(2)gl, CG12344, cv-c, Grip, 5-HT7,

						Src64B, rut, Rph, pum, Syt1, rl, unc-13-4A, X11Lbeta, CG32447, Src42A, Snap25, dysc
regulation of primary metabolic process	171 of 856 genes, 20.0%	1889 of 16085 genes, 11.7%	1.73e-09	0.00%	0.00	sbb, CG10185, jing, caup, lilli, Camta, dpy, tna, eIF4EHP, Sxl, luna, osa, l(3)72Ab, CG33144, Atf6, CG12605, CG12054, crb, AGO3, hid, Mef2, inv, cno, lab, CG10947, Hcf, Usp10, dsf, scrt, ap, Tlk, Awh, Spt3, Sox21a, Eip75B, Snoo, Fancm, hng3, crp, CG8312, pum, mamo, Blimp-1, stv, alph, msi, Src42A, Eip78C, stau, timeout, Tis11, hth, Doa, ara, mam, Ptp61F, shn, foxo, CG5694, grn, per, CG3726, CG17514, Pka-R2, bun, Tao, Spt20, opa, pdm3, InR, kay, hppy, sima, Rx, CG12769, CG11247, nerfin-1, wnd, bin3, Stat92E, Src64B, dsx, chinmo, klu, SCAP, pip, fra, Lim1, Parp, MBD-R2, lola, Smr, TfAP-2, Fs, vvl, abd-A, mbl, nau, MED14, Eip93F, bab2, Rev1, pnt, melt, tefu, corto, ltl, cv-2, rhea, lmd, ich, hh, Oaz, l(2)gl, wdb, bol, Dr, ush, Poxm, Antp, ko, sNPF-R, CtBP, tara, bru3, CG1815, Tet, RunxB, CG4238, ken, zen, kek5, Hr4, kibra, al, EcR, Rok, e(y)3, nej, upSET, Maf1, ey, CG31612, Oamb, dlg1, step, par-1, smg, heph, CG8405, hbs, ps, Lim3, gpp, cic, Dad, Pde8, lncRNA:acal, crol, sd, bru2, RecQ4, en, fs(1)h, Rbfox1, CG9932, rl, vn, tut, Gbs-70E, Tl
regulation of macromolecule metabolic process	179 of 856 genes, 20.9%	2022 of 16085 genes, 12.6%	3.14e-09	0.00%	0.00	sbb, CG10185, jing, caup, lilli, Camta, dpy, tna, eIF4EHP, Sxl, luna, osa, l(3)72Ab, CG33144, Atf6, CG12605, CG12054, crb, AGO3, hid, Mef2, inv, cno, lab, CG10947, Hcf, Usp10, dsf, scrt, ap, Tlk, Awh, Spt3, Sox21a, Eip75B, Snoo, Fancm, hng3, crp, CG8312, pum, mamo, Blimp-1, stv, alph, msi, Src42A, Eip78C, stau, timeout, Tis11, hth, ft, Doa, ara, mam, Ptp61F, shn, foxo, CG5694, grn, Tob, per, CG3726, CG17514, Pka-R2, bun, Tao, Spt20, opa, pdm3, InR, kay, hppy, sima, Rx, CG12769, CG11247, Achl, nerfin-1, wnd, bin3, Stat92E, Src64B, CG6701, dsx, chinmo, klu, SCAP, elB, pip, fra, Lim1, Parp, MBD-R2, lola, Smr, TfAP-2, Fs, vvl, abd-A, mbl, nau, MED14, Eip93F, bab2, Rev1, pnt, melt, tefu, corto, ltl, cv-2, rhea, lmd, ich, hh, Oaz, l(2)gl, wdb, bol, Dr, ush, Poxm, Antp, ko, sNPF-R, CtBP, tara, bru3, CG1815, CG11486, Tet, RunxB, CG4238, ken, zen, kek5, Hr4, kibra, al, EcR, Rok, e(y)3, nej, upSET, Maf1, ey, CG31612, Oamb, dlg1, step, par-1, smg, heph, Hipk, CG8405, hbs, ps, Lim3, gpp, cic, Dad, Pde8, lncRNA:acal, crol, sd, CG4744, bru2, RecQ4, en, fs(1)h, Rbfox1, CG9932, rl, vn, tut, Tl, Gbs-70E
eye photoreceptor cell differentiation	39 of 856 genes, 4.6%	203 of 16085 genes, 1.3%	5.02e-09	0.00%	0.00	hth, lola, boss, Doa, lilli, mbl, S, CG5921, neur, Ten-m, pnt, melt, nej, crb, bun, ed, CadN2, kay, tinc, hbs, hh, bdg, PDZ-GEF, csw, Src64B, ex, Mbs, rl, RasGAP1, amon, cdi, klu, alph, msi, Src42A, CG13251, elB, stan, dysc
epithelial cell development	56 of 856 genes, 6.5%	381 of 16085 genes, 2.4%	9.61e-09	0.00%	0.00	Parp, Smr, dome, kuz, jing, spir, Fs(2)Ket, S, mam, stl, Dhc64C, kibra, Tie, EcR, Rok, stai, neur, toc, egh, alpha-Cat, nej, crb, bun, Tao, smog, ed, if, Mef2, lncRNA:flam, dlg1, kay, InR, par-1, spri, CG41099, rhea, dia, jvl, Abl, Gprk2, hh, cic, csw, Stat92E, RecQ4, Src64B, ex, rl, vn, Rme-8, cher, tyn, loco, Fas3, bbg, cta
enzyme linked	46 of 856	278 of 16085	1.35e-08	0.00%	0.00	boss, CG31183, Fs, PVRAP, FER, S,

receptor protein signaling pathway	genes, 5.4%	genes, 1.7%				kek5, Ptp61F, shn, foxo, Tie, CG34357, pnt, melt, pyr, ltl, ey, ed, cv-2, step, dlg1, InR, hppy, sima, drl, Dad, cic, wdb, Snoo, Drl-2, csw, Src64B, ths, sfl, pum, rl, vn, RasGAP1, Mmp2, trol, cdi, alph, Tl, Src42A, fra, ckn
circadian rhythm	33 of 856 genes, 3.9%	156 of 16085 genes, 1.0%	1.65e-08	0.00%	0.00	Sh, dome, TfAP-2, wake, e, foxo, Hk, EcR, Grd, per, nej, bgm, Pka-R2, hid, Shab, Pura, Mef2, dlg1, Oamb, InR, kay, slo, Rh7, CG12344, Ac3, rut, Dop1R2, Mbs, Ac78C, unc80, tara, timeout, dysc
rhythmic process	33 of 856 genes, 3.9%	158 of 16085 genes, 1.0%	2.39e-08	0.00%	0.00	Sh, dome, TfAP-2, wake, e, foxo, Hk, EcR, Grd, per, nej, bgm, Pka-R2, hid, Shab, Pura, Mef2, dlg1, Oamb, InR, kay, slo, Rh7, CG12344, Ac3, rut, Dop1R2, Mbs, Ac78C, unc80, tara, timeout, dysc
motor neuron axon guidance	23 of 856 genes, 2.7%	79 of 16085 genes, 0.5%	2.62e-08	0.00%	0.00	fz2, Ptp99A, beat-VI, vvl, Abl, Lim3, beat-IIa, Drl-2, grn, beat-Vc, Nrg, beat-Ic, Ten-m, beat-IIIb, Mmp2, trol, ko, beat-Va, cher, beat-Ib, beat-IIb, fra, Ten-a
positive regulation of cell communication	58 of 856 genes, 6.8%	414 of 16085 genes, 2.6%	2.92e-08	0.00%	0.00	Parp, lola, CG43658, Cbp53E, ft, CG34393, Doa, Fs, Fife, PVRAP, S, CG42674, PsGEF, BicD, Sxl, foxo, kibra, neur, melt, nej, TyrR, Prosap, crb, Tao, ed, cv-2, Pura, step, kay, InR, hppy, Usp10, siz, Hipk, KaiR1D, Oct-TyrR, Gprk2, hbs, Pde8, wdb, cv-c, wnd, Snoo, RhoGEF64C, ex, Src64B, vn, trol, CG15611, sNPF-R, CtBP, CG30456, Src42A, pip, fra, pyd, ds, Gprk1
positive regulation of signaling	58 of 856 genes, 6.8%	414 of 16085 genes, 2.6%	2.92e-08	0.00%	0.00	Parp, lola, CG43658, Cbp53E, ft, CG34393, Doa, Fs, Fife, PVRAP, S, CG42674, PsGEF, BicD, Sxl, foxo, kibra, neur, melt, nej, TyrR, Prosap, crb, Tao, ed, cv-2, Pura, step, kay, InR, hppy, Usp10, siz, Hipk, KaiR1D, Oct-TyrR, Gprk2, hbs, Pde8, wdb, cv-c, wnd, Snoo, RhoGEF64C, ex, Src64B, vn, trol, CG15611, sNPF-R, CtBP, CG30456, Src42A, pip, fra, pyd, ds, Gprk1
nervous system process	72 of 856 genes, 8.4%	582 of 16085 genes, 3.6%	3.95e-08	0.00%	0.00	Sh, Gr22e, DCX-EMAP, boss, dome, CG9492, dpr12, lilli, dpr8, nAChRalpha2, rdgA, CG5921, Obp56d, dpr9, dpr1, Grd, Ggamma30A, rdgC, if, Sap47, Gr23a, dia, Oct-TyrR, sra, drl, dpr6, rut, pum, Ac78C, axo, stau, dpr13, dysc, Or67d, trp, futsch, nAChRalpha6, pHCl-1, nemy, nompC, EcR, Tob, mew, neur, Ir40a, per, Nrx-1, ATP8B, Prosap, Ca-alpha1T, Oamb, pdm3, nAChRbeta2, Mvl, f, Abl, Dhc36C, Rh7, cac, CG12344, dpr2, CG8086, wnd, Stat92E, Src64B, Btk29A, Gr28b, Rbfox1, vn, Orco, cher, SKIP
regulation of cell differentiation	61 of 856 genes, 7.1%	452 of 16085 genes, 2.8%	4.19e-08	0.00%	0.00	hth, lola, ec, vvl, kuz, nau, Ser, stl, Dhc64C, kibra, Tie, EcR, bab2, Rok, DAAM, neur, osa, pnt, Trim9, Prosap, bun, pyr, hid, ed, dlg1, kay, InR, par-1, spri, tinc, Abl, l(2)gl, PDZ-GEF, sd, nerfin-1, Sox21a, cv-c, Dr, csw, Stat92E, Src64B, ex, ush, Sema2a, Syt1, kirre, Mbs, rl, RasGAP1, vn, Rme-8, cdi, Antp, klu, alph, cher, Src42A, stau, fra, stan, unk
positive regulation of response to stimulus	65 of 856 genes, 7.6%	499 of 16085 genes, 3.1%	4.30e-08	0.00%	0.00	Parp, lola, CG34393, vvl, Fs, PVRAP, CG42674, Sxl, melt, TyrR, crb, hid, smog, cv-2, Usp10, dia, Oct-TyrR, Gprk2, wdb, Snoo, ex, ush, Mmp2, trol, CG15611, sNPF-R, CtBP, CG30456, Src42A, ds, stan, CG43658, ft, Doa, S, PsGEF, kibra, foxo, Rok, neur, nej, Prosap, Tao, ed, Pura, step, InR, kay, hppy, siz, Hipk, CG8405, hbs, Pde8, wnd, RhoGEF64C,

						Src64B, Dop1R2, rl, vn, Tl, pip, pyd, fra, Gprk1
regulation of metabolic process	185 of 856 genes, 21.6%	2178 of 16085 genes, 13.5%	4.72e-08	0.00%	0.00	sbb, CG10185, Sesn, jing, caup, lilli, Camta, dpy, tna, eIF4EHP, Sxl, luna, osa, l(3)72Ab, CG33144, Atf6, CG12605, CG12054, crb, AGO3, hid, Mef2, inv, cno, lab, CG10947, Hcf, Usp10, dsf, scrt, ap, Tlk, Gprk2, Awh, Spt3, Sox21a, Eip75B, Snoo, Fancm, hng3, crp, CG8312, pum, mamo, Blimp-1, stv, alph, msi, Src42A, Eip78C, stau, timeout, Tis11, hth, ft, Doa, ara, mam, Ptp61F, shn, foxo, CG5694, grn, Tob, per, CG3726, CG17514, Pka-R2, bun, Tao, Spt20, opa, kay, pdm3, InR, hppy, sima, Rx, CG12769, CG11247, Achl, nerfin-1, wnd, bin3, Stat92E, Src64B, CG6701, dsx, chinmo, klu, SCAP, elB, pip, fra, Lim1, Parp, MBD-R2, lola, Smr, TfAP-2, Fs, vvl, abd-A, mbl, nau, MED14, Eip93F, bab2, Rev1, pnt, melt, tefu, corto, ltl, smog, cv-2, rhea, lmd, ich, Debcl, hh, Oaz, l(2)gl, wdb, bol, Dr, ush, Poxm, Antp, ko, sNPF-R, CtBP, tara, bru3, CG1815, CG11486, Tet, RunxB, CG4238, ken, zen, e, kek5, Hr4, kibra, al, EcR, Rok, e(y)3, nej, upSET, Maf1, ey, CG31612, Oamb, dlg1, step, par-1, smg, heph, Hipk, CG8405, hbs, ps, Lim3, gpp, cic, Dad, Pde8, lncRNA:acal, crol, sd, CG4744, bru2, RecQ4, en, fs(1)h, Gyf, Rbfox1, CG9932, rl, vn, tut, Tl, Gbs-70E
cellular component organization	246 of 856 genes, 28.7%	3146 of 16085 genes, 19.6%	4.85e-08	0.00%	0.00	fz2, sbb, Sh, DCX-EMAP, CG1724, Sesn, Nlg1, kuz, jing, Slc25A46, lilli, Vps13D, tna, CG5921, Sxl, CG5142, luna, osa, l(3)72Ab, alpha-Cat, Trim9, Gnf1, Nlg3, crb, robo3, sns, hid, if, Ten-a, cno, Ptp99A, Hcf, ap, dtr, C3G, jvl, lt, Tlk, Gprk2, sra, snky, dpr6, Spt3, CG42748, cv-c, Snoo, Kank, Fancm, CG33298, DIP-gamma, rut, Nrg, pum, unc-13-4A, mamo, CG34384, Mmp2, trol, Src42A, beat-IIb, beat-Ib, stau, dpr13, Snap25, ds, dysc, Ncc69, CG43658, mtg, ft, qua, sas, ara, FER, S, mam, Ptp61F, PsGEF, Gem3, shn, foxo, DAAM, grn, Tob, Scgdelta, neur, beat-Vc, mew, HP1Lcsd, Prosap, app, Pka-R2, bun, Tao, ed, Spt20, CadN2, loh, kay, InR, pdm3, hppy, sima, f, DIP-theta, Trpm, PDZ-GEF, nerfin-1, dpr2, wnd, bin3, RhoBTB, Stat92E, Src64B, Btk29A, CG6701, dsx, Sema2a, chinmo, Bsg, cdi, loco, fra, tral, Parp, lola, vkg, NKCC, vvl, Fife, dpr12, nau, mbl, dpr8, DIP-zeta, stl, dpr9, dpr1, milt, stai, egh, Gie, jbug, tefu, LRR, corto, smog, Msp300, Sb, DIP-epsilon, CG42342, CG41099, rhea, dia, ich, AdamTS-A, Debcl, hh, drl, l(2)gl, wdb, Fur1, Grip, Drl-2, csw, ex, beat-Ic, Poxm, Mbs, RasGAP1, Xrp1, ko, beat-Va, CtBP, tyn, tara, htt, bru3, RhoGAP18B, stan, ckn, cta, trp, futsch, beat-VI, CG11486, wake, gukh, Jupiter, cora, spir, sff, beat-IIa, Wdr62, BicD, Dhc64C, EcR, Rok, Ten-m, e(y)3, Nrx-1, nej, upSET, ATP8B, ACC, Mmp1, Shab, ey, Pura, step, dlg1, par-1, spri, CG43897, heph, eRF3, siz, slo, CG42319, Abl, Lim3, gpp, Unc-115a, cac, Dad, cic, CCKLR-17D1, Patronin, crol, RhoGEF64C, RecQ4, bru2, Tom40, en, ths, fs(1)h, beat-IIIb, Syt1, rl, kirre, vn, cher, Tl, Fas3, CG5758, pyd
synapse organization	47 of 856 genes, 5.5%	299 of 16085 genes, 1.9%	5.09e-08	0.00%	0.00	fz2, futsch, mtg, Nlg1, Fife, sff, dpr12, dpr8, DIP-zeta, Gem3, dpr9, dpr1, stai, Ten-m, Gie, nej, Nrx-1, Prosap, Nlg3, Ten-a, dlg1,

						kay, par-1, DIP-epsilon, DIP-theta, slo, Abl, drl, dpr6, cac, Dad, CCKLR-17D1, dpr2, wnd, Grip, Fur1, Src64B, DIP-gamma, rut, Nrg, pum, Tl, Src42A, stau, dpr13, stan, dysc
regulation of transcription by RNA polymerase II	83 of 856 genes, 9.7%	723 of 16085 genes, 4.5%	5.24e-08	0.00%	0.00	MBD-R2, sbb, lola, Smr, TfAP-2, vvl, jing, Fs, abd-A, nau, caup, lilli, Camta, MED14, tna, bab2, luna, osa, pnt, CG12605, CG12054, ltl, cv-2, Mef2, inv, lab, Usp10, dsf, scrt, ap, lmd, ich, Awh, Spt3, Eip75B, Snoo, ush, crp, CG8312, mamo, Blimp-1, Antp, ko, CtBP, hth, Tet, ara, kek5, zen, mam, foxo, shn, EcR, CG5694, per, e(y)3, CG3726, nej, ey, Spt20, Oamb, dlg1, opa, kay, sima, CG12769, CG11247, Dad, cic, crol, sd, nerfin-1, Stat92E, en, fs(1)h, dsx, CG9932, chinmo, klu, SCAP, Tl, fra, Lim1
circadian behavior	27 of 856 genes, 3.2%	112 of 16085 genes, 0.7%	5.64e-08	0.00%	0.00	Sh, dome, TfAP-2, wake, slo, Rh7, e, foxo, Hk, EcR, Ac3, Dop1R2, per, Mbs, bgm, nej, unc80, Pka-R2, tara, Shab, Pura, Mef2, dysc, dlg1, Oamb, InR, kay
compound eye photoreceptor cell differentiation	36 of 856 genes, 4.2%	192 of 16085 genes, 1.2%	7.44e-08	0.00%	0.00	hth, lola, boss, Doa, lilli, mbl, S, neur, Ten-m, pnt, melt, nej, crb, bun, ed, CadN2, kay, hbs, hh, bdg, PDZ-GEF, csw, Src64B, ex, rl, Mbs, RasGAP1, amon, cdi, klu, alph, msi, elB, Src42A, stan, dysc
transcription, DNA-templated	121 of 856 genes, 14.1%	1242 of 16085 genes, 7.7%	7.85e-08	0.00%	0.00	MBD-R2, Parp, sbb, lola, Smr, TfAP-2, vvl, jing, Fs, abd-A, nau, caup, lilli, Camta, dpy, MED14, tna, Eip93F, bab2, luna, osa, pnt, Atf6, melt, CG12605, tefu, CG12054, corto, ltl, cv-2, Mef2, inv, lab, Hcf, Usp10, rhea, dsf, scrt, ap, lmd, ich, Tlk, Awh, Spt3, Oaz, Sox21a, Eip75B, Dr, Snoo, ush, hng3, crp, CG8312, Poxm, pum, mamo, Blimp-1, Antp, ko, CtBP, tara, Eip78C, hth, CG1815, Tet, RunxB, ara, ken, kek5, zen, mam, Hr4, shn, kibra, foxo, al, CG5694, EcR, grn, per, e(y)3, CG3726, nej, upSET, Maf1, bun, Rpl135, ey, Spt20, dlg1, Oamb, opa, pdm3, kay, smg, sima, Rx, CG12769, Tif-IA, Lim3, gpp, CG11247, Dad, cic, crol, sd, nerfin-1, bin3, Stat92E, en, fs(1)h, dsx, Rbfox1, CG9932, rl, chinmo, klu, SCAP, Tl, fra, Lim1
nucleic acid-templated transcription	121 of 856 genes, 14.1%	1242 of 16085 genes, 7.7%	7.85e-08	0.00%	0.00	MBD-R2, Parp, sbb, lola, Smr, TfAP-2, vvl, jing, Fs, abd-A, nau, caup, lilli, Camta, dpy, MED14, tna, Eip93F, bab2, luna, osa, pnt, Atf6, melt, CG12605, tefu, CG12054, corto, ltl, cv-2, Mef2, inv, lab, Hcf, Usp10, rhea, dsf, scrt, ap, lmd, ich, Tlk, Awh, Spt3, Oaz, Sox21a, Eip75B, Dr, Snoo, ush, hng3, crp, CG8312, Poxm, pum, mamo, Blimp-1, Antp, ko, CtBP, tara, Eip78C, hth, CG1815, Tet, RunxB, ara, ken, kek5, zen, mam, Hr4, shn, kibra, foxo, al, CG5694, EcR, grn, per, e(y)3, CG3726, nej, upSET, Maf1, bun, Rpl135, ey, Spt20, dlg1, Oamb, opa, pdm3, kay, smg, sima, Rx, CG12769, Tif-IA, Lim3, gpp, CG11247, Dad, cic, crol, sd, nerfin-1, bin3, Stat92E, en, fs(1)h, dsx, Rbfox1, CG9932, rl, chinmo, klu, SCAP, Tl, fra, Lim1
rhythmic behavior	27 of 856 genes, 3.2%	114 of 16085 genes, 0.7%	8.77e-08	0.00%	0.00	Sh, dome, TfAP-2, wake, slo, Rh7, e, foxo, Hk, EcR, Ac3, Dop1R2, per, Mbs, bgm, nej, unc80, Pka-R2, tara, Shab, Pura, Mef2, dysc, dlg1, Oamb, InR, kay
RNA biosynthetic process	121 of 856 genes, 14.1%	1245 of 16085 genes, 7.7%	9.18e-08	0.00%	0.00	MBD-R2, Parp, sbb, lola, Smr, TfAP-2, vvl, jing, Fs, abd-A, nau, caup, lilli, Camta, dpy, MED14, tna, Eip93F, bab2, luna, osa, pnt, Atf6, melt, CG12605, tefu, CG12054, corto, ltl, cv-2, Mef2, inv, lab, Hcf, Usp10, rhea,

						dsf, scrt, ap, Imd, ich, Tlk, Awh, Spt3, Oaz, Sox21a, Eip75B, Dr, Snoo, ush, hng3, crp, CG8312, Poxm, pum, mamo, Blimp-1, Antp, ko, CtBP, tara, Eip78C, hth, CG1815, Tet, RunxB, ara, ken, kek5, zen, mam, Hr4, shn, kibra, foxo, al, CG5694, EcR, grn, per, e(y)3, CG3726, nej, upSET, Maf1, bun, Rpl135, ey, Spt20, dlg1, Oamb, opa, pdm3, kay, smg, sima, Rx, CG12769, Tif-IA, Lim3, gpp, CG11247, Dad, cic, crol, sd, nerfin-1, bin3, Stat92E, en, fs(1)h, dsx, Rbfox1, CG9932, rl, chinmo, klu, SCAP, Tl, fra, Lim1
regulation of gene expression	145 of 856 genes, 16.9%	1592 of 16085 genes, 9.9%	9.58e-08	0.00%	0.00	sbb, jing, caup, lilli, Camta, dpy, tna, eIF4EHP, Sxl, luna, osa, l(3)72Ab, Atf6, CG12605, CG12054, AGO3, Mef2, inv, lab, CG10947, Hcf, Usp10, dsf, scrt, ap, Tlk, Awh, Spt3, Sox21a, Eip75B, Snoo, hng3, crp, CG8312, pum, mamo, Blimp-1, msi, Eip78C, stau, Tis11, hth, ft, Doa, ara, mam, shn, foxo, CG5694, grn, Tob, per, CG3726, CG17514, bun, Spt20, opa, pdm3, kay, sima, Rx, CG12769, CG11247, Achl, nerfin-1, bin3, Stat92E, CG6701, dsx, chinmo, klu, SCAP, elB, fra, Lim1, Parp, MBD-R2, lola, Smr, TfAP-2, Fs, vvl, abd-A, mbl, nau, MED14, Eip93F, bab2, pnt, melt, tefu, corto, ltl, cv-2, rhea, Imd, ich, hh, Oaz, bol, Dr, ush, Poxm, Antp, ko, CtBP, tara, bru3, CG1815, CG11486, Tet, RunxB, zen, kek5, ken, Hr4, kibra, EcR, al, e(y)3, nej, upSET, Maf1, ey, Oamb, dlg1, smg, heph, Hipk, ps, gpp, Lim3, cic, Dad, sd, crol, CG4744, bru2, fs(1)h, en, rl, CG9932, Rbfox1, tut, Tl
positive regulation of cellular metabolic process	91 of 856 genes, 10.6%	842 of 16085 genes, 5.2%	1.44e-07	0.00%	0.00	MBD-R2, Parp, lola, TfAP-2, Sesn, vvl, jing, abd-A, nau, caup, lilli, Camta, tna, eIF4EHP, Sxl, Eip93F, osa, pnt, CG33144, CG12054, AGO3, hid, smog, Mef2, lab, Hcf, dsf, ap, Imd, ich, Gprk2, Debcl, Awh, Oaz, bol, Eip75B, ush, CG8312, pum, Antp, stv, sNPF-R, CtBP, tara, Src42A, stau, Tis11, hth, Tet, Doa, ara, zen, mam, foxo, shn, EcR, e(y)3, nej, CG17514, Tao, ey, CG31612, Oamb, opa, step, InR, kay, smg, hppy, sima, CG8405, CG12769, hbs, ps, gpp, CG11247, Pde8, sd, wnd, Stat92E, Src64B, RecQ4, en, fs(1)h, dsx, Rbfox1, vn, SCAP, Tl, pip, Lim1
larval development	33 of 856 genes, 3.9%	169 of 16085 genes, 1.1%	1.61e-07	0.00%	0.00	fz2, lola, ft, sas, nau, Ser, EcR, neur, pnt, pyr, sns, Mmp1, dlg1, loh, Msp300, rhea, ap, AdamTS-A, hh, Dad, l(2)gl, nvd, Stat92E, RecQ4, ths, ush, crp, Poxm, rl, kirre, cher, Tl, unk
response to external stimulus	104 of 856 genes, 12.1%	1019 of 16085 genes, 6.3%	1.63e-07	0.00%	0.00	fz2, Parp, sbb, Sh, lola, dome, Sesn, vvl, kuz, jing, Camta, Duox, rdgA, CalpA, egh, melt, Trim9, jbug, Ggamma30A, rdgC, robo3, hid, smog, if, Mef2, inv, Ten-a, Ptp99A, ap, lt, Gprk2, Debcl, hh, drl, Drl-2, ush, DIP-gamma, Nrg, beat-Ic, Lmpt, Mmp2, trol, ko, beat-Va, Src42A, beat-IIb, beat-Ib, timeout, stan, dysc, ckn, trp, beat-VI, Doa, gukh, CPT2, sas, slgA, FER, Ptp61F, shep, beat-IIa, polyph, nompC, foxo, EcR, grn, DAAM, mew, beat-Vc, Ten-m, per, Pka-R2, CadN2, dlg1, Oamb, pdm3, InR, kay, CG34353, Hipk, Abl, Rh7, Lim3, Unc-115a, cac, Dad, nerfin-1, Stat92E, RhoGEF64C, Src64B, en, Gr28b, beat-IIIb, dsx, Sema2a, rl, cher, Tl, loco, Fas3, ogre, fra, Gprk1
neuron recognition	27 of 856	117 of 16085	1.66e-07	0.00%	0.00	fz2, Ptp99A, lola, esn, Abl, dpr12, drl, pot,

	genes, 3.2%	genes, 0.7%				Fur1, Drl-2, mew, beat-Ic, Ten-m, dsx, Sema2a, Trim9, Mmp2, trol, Tl, robo3, Fas3, Mmp1, CG5758, if, fra, Ten-a, stan
cell migration	48 of 856 genes, 5.6%	320 of 16085 genes, 2.0%	1.67e-07	0.00%	0.00	fz2, dome, kuz, jing, abd-A, FER, stl, Dhc64C, kibra, Tie, EcR, stai, mew, egh, pnt, nej, bun, pyr, ey, smog, if, opa, Msp300, kay, par-1, spri, sima, rhea, AdamTS-A, Abl, hh, Dad, PDZ-GEF, RhoBTB, csw, Stat92E, ex, ths, sfl, pum, Sema2a, vn, Mmp2, Rme-8, Src42A, bbg, fra, cta
positive regulation of signal transduction	53 of 856 genes, 6.2%	381 of 16085 genes, 2.4%	3.02e-07	0.00%	0.00	Parp, lola, CG43658, ft, CG34393, Doa, Fs, PVRAP, S, CG42674, PsGEF, Sxl, foxo, kibra, neur, melt, nej, TyrR, Prosap, crb, Tao, ed, cv-2, Pura, step, kay, InR, hppy, Usp10, siz, Hipk, Oct-TyrR, Gprk2, hbs, Pde8, wdb, Snoo, wnd, RhoGEF64C, ex, Src64B, vn, trol, CG15611, sNPF-R, CtBP, CG30456, Src42A, pip, fra, pyd, ds, Gprk1
positive regulation of nitrogen compound metabolic process	87 of 856 genes, 10.2%	802 of 16085 genes, 5.0%	3.28e-07	0.00%	0.00	MBD-R2, Parp, lola, TfAP-2, vvl, jing, abd-A, nau, caup, lilli, Camta, tna, Sxl, Eip93F, osa, pnt, CG33144, CG12054, AGO3, hid, smog, Mef2, lab, Hcf, dsf, ap, lmd, ich, Gprk2, Awh, Oaz, bol, Eip75B, ush, CG8312, pum, Antp, stv, sNPF-R, CtBP, tara, Src42A, stau, Tis11, hth, Tet, CG4238, ara, zen, mam, foxo, shn, EcR, e(y)3, nej, Tao, ey, CG31612, Oamb, opa, step, InR, kay, smg, hppy, sima, CG8405, hbs, CG12769, ps, gpp, CG11247, Pde8, sd, wnd, Stat92E, Src64B, RecQ4, en, fs(1)h, dsx, Rbfox1, vn, SCAP, Tl, pip, Lim1
anatomical structure formation involved in morphogenesis	69 of 856 genes, 8.1%	574 of 16085 genes, 3.6%	3.87e-07	0.00%	0.00	abd-A, nau, caup, Fs(2)Ket, CalpA, PyK, CLIP-190, egh, pyr, sns, if, Mef2, T48, rost, rhea, poe, dia, C3G, jvl, lmd, ich, hh, l(2)gl, cv-c, uif, spz3, Mmp2, CtBP, ds, hth, ft, spir, ara, zen, mam, S, BicD, foxo, shn, Dhc64C, EcR, Rok, Scgdelta, DAAM, mew, neur, bun, Mmp1, ed, InR, par-1, heph, siz, Abl, hbs, cic, Stat92E, Src64B, RecQ4, Btk29A, ths, sfl, kirre, rl, Rbfox1, Bsg, klu, cher, fra
transcription by RNA polymerase II	84 of 856 genes, 9.8%	767 of 16085 genes, 4.8%	4.36e-07	0.00%	0.00	MBD-R2, sbb, lola, Smr, TfAP-2, vvl, jing, Fs, abd-A, nau, caup, lilli, Camta, MED14, tna, bab2, luna, osa, pnt, CG12605, CG12054, ltl, cv-2, Mef2, inv, lab, Usp10, dsf, scrt, ap, lmd, ich, Awh, Spt3, Eip75B, Snoo, ush, crp, CG8312, mamo, Blimp-1, Antp, ko, CtBP, hth, Tet, ara, kek5, zen, mam, foxo, shn, EcR, CG5694, per, e(y)3, CG3726, nej, ey, Spt20, Oamb, dlg1, opa, kay, sima, Rx, CG12769, CG11247, Dad, cic, crol, sd, nerfin-1, Stat92E, en, fs(1)h, dsx, CG9932, chinmo, klu, SCAP, Tl, fra, Lim1
cell recognition	27 of 856 genes, 3.2%	122 of 16085 genes, 0.8%	4.62e-07	0.00%	0.00	fz2, Ptp99A, lola, esn, Abl, dpr12, drl, pot, Fur1, Drl-2, mew, beat-Ic, Ten-m, dsx, Sema2a, Trim9, Mmp2, trol, Tl, robo3, Fas3, Mmp1, CG5758, if, fra, Ten-a, stan
regulation of anatomical structure morphogenesis	42 of 856 genes, 4.9%	265 of 16085 genes, 1.6%	4.64e-07	0.00%	0.00	hth, lola, ec, vvl, kuz, nau, FER, shn, Rok, DAAM, neur, Trim9, hid, smog, ed, cno, par-1, dia, tinc, Abl, l(2)gl, PDZ-GEF, sd, cv-c, RhoBTB, csw, Src64B, Sema2a, Mbs, kirre, rl, RasGAP1, cdi, klu, alph, tyn, Src42A, stan, ds, pyd, fra, cta
regulation of cell development	51 of 856 genes, 6.0%	364 of 16085 genes, 2.3%	5.26e-07	0.00%	0.00	hth, lola, ec, vvl, kuz, stl, Dhc64C, kibra, Tie, EcR, Rok, DAAM, neur, osa, pnt, Trim9, Prosap, bun, pyr, hid, ed, dlg1, InR, kay, par-1, spri, tinc, Abl, l(2)gl, PDZ-GEF,

						sd, nerfin-1, cv-c, csw, Src64B, Sema2a, Syt1, Mbs, rl, RasGAP1, vn, Rme-8, cdi, Antp, klu, alph, Src42A, stau, fra, stan, unk
positive regulation of metabolic process	94 of 856 genes, 11.0%	906 of 16085 genes, 5.6%	6.25e-07	0.00%	0.00	MBD-R2, Parp, lola, TfAP-2, Sesn, vvl, jing, abd-A, nau, caup, lilli, Camta, tna, eIF4EHP, Sxl, Eip93F, osa, pnt, CG33144, CG12054, AGO3, hid, smog, Mef2, lab, Hcf, dsf, ap, Imd, ich, Gprk2, Debcl, Awh, Oaz, bol, Eip75B, ush, CG8312, pum, Antp, stv, sNPF-R, CtBP, tara, Src42A, stau, Tis11, hth, Tet, Doa, CG4238, ara, zen, mam, foxo, shn, EcR, e(y)3, nej, CG17514, Tao, ey, CG31612, Oamb, opa, step, InR, kay, smg, hppy, sima, Hipk, CG8405, CG12769, hbs, ps, Lim3, gpp, CG11247, Pde8, sd, wnd, Stat92E, Src64B, RecQ4, en, fs(1)h, dsx, Rbfox1, vn, SCAP, Tl, pip, Lim1
cellular component organization or biogenesis	250 of 856 genes, 29.2%	3291 of 16085 genes, 20.5%	6.35e-07	0.00%	0.00	fz2, sbb, Sh, DCX-EMAP, CG1724, CG10185, Sesn, Nlg1, kuz, jing, Slc25A46, lilli, Vps13D, tna, CG5921, Sxl, CG5142, luna, osa, l(3)72Ab, alpha-Cat, Trim9, Gnf1, Nlg3, CG7544, crb, robo3, sns, hid, if, Ten-a, cno, Ptp99A, CG11030, Hcf, ap, dtr, C3G, jvl, lt, Tlk, Gprk2, sra, snky, dpr6, Spt3, CG42748, cv-c, Snoo, Kank, Fancm, CG33298, DIP-gamma, rut, Nrg, pum, unc-13-4A, mamo, CG34384, Mmp2, trol, Src42A, beat-IIb, beat-Ib, stau, dpr13, Snap25, ds, dysc, Ncc69, CG43658, mtg, ft, qua, sas, ara, FER, S, mam, Ptp61F, PsGEF, Gem3, shn, foxo, DAAM, grn, Tob, Scgdelta, neur, beat-Vc, mew, HP1Lcsd, Prosap, app, Pka-R2, bun, Tao, ed, Spt20, CadN2, loh, kay, InR, pdm3, hppy, sima, f, DIP-theta, Trpm, PDZ-GEF, nerfin-1, dpr2, wnd, bin3, RhoBTB, Stat92E, Src64B, Btk29A, CG6701, dsx, Sema2a, chinmo, Bsg, cdi, loco, fra, tral, Parp, lola, vkg, NKCC, vvl, Fife, dpr12, nau, mbl, dpr8, DIP-zeta, stl, dpr9, dpr1, milt, stai, egh, Gie, jbug, tefu, LRR, corto, smog, Msp300, Sb, DIP-epsilon, CG42342, CG41099, rhea, dia, ich, AdamTS-A, Debcl, hh, drl, l(2)gl, wdb, Fur1, Grip, Drl-2, csw, ex, beat-Ic, Poxm, Mbs, RasGAP1, Xrp1, ko, beat-Va, CtBP, tyn, tara, htt, bru3, RhoGAP18B, stan, ckn, cta, trp, futsch, beat-VI, CG11486, wake, gukh, Jupiter, cora, spir, sff, beat-IIa, Wdr62, BicD, Dhc64C, EcR, Rok, Ten-m, e(y)3, RluA-1, Nrx-1, nej, upSET, ATP8B, ACC, Mmp1, Shab, ey, Pura, step, dlg1, par-1, spri, CG43897, heph, eRF3, siz, slo, CG42319, Abl, Lim3, gpp, Unc-115a, cac, Dad, cic, CCKLR-17D1, Patronin, crol, RhoGEF64C, RecQ4, bru2, Tom40, en, ths, fs(1)h, beat-IIIb, Syt1, rl, kirre, vn, cher, Tl, Fas3, CG5758, pyd
muscle organ development	25 of 856 genes, 2.9%	107 of 16085 genes, 0.7%	6.38e-07	0.00%	0.00	vkg, rhea, ap, C3G, Imd, kuz, hbs, abd-A, Tsp, drl, nau, mbl, Dad, Dr, Grip, mew, ths, Dys, kirre, pyr, sns, ed, if, Mef2, Msp300
actin filament-based process	46 of 856 genes, 5.4%	312 of 16085 genes, 1.9%	7.56e-07	0.00%	0.00	CG43658, qua, gukh, cora, spir, FER, PsGEF, Rok, DAAM, Scgdelta, mew, alpha-Cat, crb, sns, smog, ed, if, step, Msp300, Sb, CG43897, siz, f, rhea, dia, C3G, jvl, CG42319, Gprk2, Abl, l(2)gl, cv-c, RhoBTB, Kank, Src64B, Btk29A, chinmo, Bsg, cdi, cher, tyn, loco, Src42A, RhoGAP18B, cta, tral
response to organic substance	67 of 856 genes, 7.8%	559 of 16085 genes, 3.5%	8.22e-07	0.00%	0.00	Sh, boss, dome, Sesn, Fs, Duox, Obp56d, dpr9, PyK, pnt, melt, Cnx99A, TyrR, tefu,

						Ggamma30A, pyr, hid, hang, ltl, cv-2, l(2)gl, wdb, Eip75B, Snoo, csw, rut, pum, RasGAP1, Mmp2, Blimp-1, trol, ETHR, alph, Ac78C, Or67d, kek5, S, Ptp61F, Hr4, foxo, shn, EcR, per, Pka-R2, Tao, ACC, dlg1, step, InR, sima, hppy, TrissinR, mAChR-B, CG8405, Dad, l(3)psg2, Stat92E, Src64B, ths, Dop1R2, sfl, rl, Orco, cher, Gbs-70E, Tl, fra
positive regulation of RNA metabolic process	61 of 856 genes, 7.1%	487 of 16085 genes, 3.0%	9.19e-07	0.00%	0.00	MBD-R2, hth, lola, TfAP-2, Tet, vvl, jing, abd-A, nau, caup, ara, lilli, Camta, mam, zen, tna, Sxl, shn, foxo, Eip93F, EcR, osa, pnt, e(y)3, nej, CG12054, AGO3, ey, Mef2, opa, kay, Hcf, smg, lab, sima, dsf, ap, lmd, ich, CG12769, ps, Awh, gpp, CG11247, Oaz, sd, Eip75B, Stat92E, en, fs(1)h, CG8312, dsx, Rbfox1, pum, Antp, CtBP, SCAP, Tl, tara, Tis11, Lim1
respiratory system development	42 of 856 genes, 4.9%	271 of 16085 genes, 1.7%	9.49e-07	0.00%	0.00	dome, ft, jing, cora, dpy, Dhc64C, DAAM, mew, pnt, Hs6st, crb, hid, Mmp1, ed, if, InR, sima, rhea, dia, ich, AdamTS-A, hh, Oaz, sano, cv-c, RhoGEF64C, Stat92E, csw, Src64B, en, Btk29A, Nrg, sfl, uif, rl, Mmp2, Blimp-1, Src42A, elB, ds, stan, pyd
open tracheal system development	41 of 856 genes, 4.8%	262 of 16085 genes, 1.6%	1.14e-06	0.00%	0.00	dome, ft, jing, cora, dpy, Dhc64C, DAAM, mew, pnt, Hs6st, crb, hid, Mmp1, ed, if, InR, rhea, dia, AdamTS-A, ich, hh, Oaz, sano, cv-c, RhoGEF64C, Stat92E, csw, Src64B, en, Btk29A, Nrg, sfl, uif, rl, Mmp2, Blimp-1, Src42A, elB, ds, stan, pyd
nucleobase-containing compound biosynthetic process	132 of 856 genes, 15.4%	1456 of 16085 genes, 9.1%	1.17e-06	0.00%	0.00	sbb, CG31183, jing, caup, lilli, Camta, dpy, tna, luna, osa, Atf6, CG12605, CG12054, Mef2, inv, lab, Hcf, Usp10, dsf, scrt, ap, Tlk, Awh, Spt3, Sox21a, Eip75B, Snoo, rut, hng3, crp, CG8312, pum, mamo, Blimp-1, Ac78C, Eip78C, hth, ara, mam, shn, foxo, CG5694, grn, per, CG3726, bun, Spt20, opa, pdm3, kay, sima, Rx, CG12769, Tif-IA, CG11247, nerfin-1, bin3, Stat92E, dsx, chinmo, klu, SCAP, fra, Lim1, Parp, MBD-R2, lola, Smr, Gyc88E, TfAP-2, Fs, vvl, abd-A, nau, MED14, bab2, Eip93F, PyK, Rev1, pnt, melt, tefu, corto, ltl, cv-2, rhea, lmd, ich, Oaz, Dr, ush, Poxm, Antp, ko, CtBP, tara, CG1815, Tet, RunxB, zen, kek5, ken, Hr4, kibra, EcR, al, CG34357, e(y)3, nej, upSET, ACXC, Maf1, Rpl135, ey, Oamb, dlg1, smg, CG11883, gpp, Lim3, cic, Dad, sd, crol, RecQ4, Ac3, fs(1)h, en, rl, CG9932, Rbfox1, Tl
positive regulation of nucleobase-containing compound metabolic process	63 of 856 genes, 7.4%	515 of 16085 genes, 3.2%	1.24e-06	0.00%	0.00	MBD-R2, hth, lola, TfAP-2, Tet, vvl, jing, abd-A, nau, caup, ara, lilli, Camta, mam, zen, tna, Sxl, shn, foxo, Eip93F, EcR, osa, pnt, e(y)3, nej, CG12054, AGO3, ey, CG31612, Mef2, opa, kay, Hcf, smg, lab, sima, dsf, ap, lmd, ich, CG12769, ps, Awh, gpp, CG11247, Oaz, sd, Eip75B, Stat92E, RecQ4, en, fs(1)h, CG8312, dsx, Rbfox1, pum, Antp, CtBP, SCAP, Tl, tara, Tis11, Lim1
actin cytoskeleton organization	44 of 856 genes, 5.1%	295 of 16085 genes, 1.8%	1.27e-06	0.00%	0.00	CG43658, qua, gukh, cora, spir, FER, PsGEF, Rok, DAAM, Scgdelta, mew, crb, sns, smog, ed, if, step, Msp300, Sb, CG43897, siz, f, rhea, dia, C3G, jvl, CG42319, Abl, l(2)gl, cv-c, RhoBTB, Kank, Src64B, Btk29A, chinmo, Bsg, cdi, cher, tyn, loco, Src42A, RhoGAP18B, cta, tral
negative regulation of cell communication	49 of 856 genes, 5.7%	352 of 16085 genes, 2.2%	1.50e-06	0.00%	0.00	Smr, Sesn, Fs, ken, kek5, Ptp61F, Hr4, foxo, Grd, pnt, nej, tefu, LRR, chrb, crb, ltl, ed, InR, par-1, hppy, Hipk, slo, Gprk2, l(2)gl, Dad, CG7094, CG12344,

						lncRNA:acal, crol, wdb, cv-c, Snoo, CG42684, pum, uif, RasGAP1, Mmp2, cdi, CG32683, trol, alph, CtBP, Tl, Src42A, ds, pyd, dysc, Gprk1, nkd
regulation of localization	61 of 856 genes, 7.1%	494 of 16085 genes, 3.1%	1.62e-06	0.00%	0.00	Parp, trp, lola, ft, kuz, Fife, Fs(2)Ket, lilli, S, Ptp61F, nemy, stl, PsGEF, BicD, foxo, Mctp, CG2121, Tie, Hk, Rok, neur, Dys, Cnx99A, Nrx-1, ATP8B, Prosap, crb, Tao, Pde6, step, Sytbeta, kay, InR, cno, par-1, spri, Mvl, sima, Apoltp, lt, Abl, Gprk2, hh, Syt7, cac, l(2)gl, RyR, wdb, RhoBTB, Sema2a, Syt1, Ttd14, vn, Rme-8, Orco, stau, fra, stan, pyd, dysc, tral
negative regulation of signaling	49 of 856 genes, 5.7%	353 of 16085 genes, 2.2%	1.65e-06	0.00%	0.00	Smr, Sesn, Fs, ken, kek5, Ptp61F, Hr4, foxo, Grd, pnt, nej, tefu, LRR, chrb, crb, Itl, ed, InR, par-1, hppy, Hipk, slo, Gprk2, l(2)gl, Dad, CG7094, CG12344, lncRNA:acal, crol, wdb, cv-c, Snoo, CG42684, pum, uif, RasGAP1, Mmp2, cdi, CG32683, trol, alph, CtBP, Tl, Src42A, ds, pyd, dysc, Gprk1, nkd
cell-cell adhesion via plasma-membrane adhesion molecules	20 of 856 genes, 2.3%	73 of 16085 genes, 0.5%	1.94e-06	0.00%	0.00	beat-VI, ft, hbs, beat-IIa, mew, beat-Vc, beat-Ic, beat-IIIb, kirre, beat-Va, sns, beat-Ib, Fas3, beat-IIb, ed, if, ds, CadN2, stan, beat-VII
photoreceptor cell fate commitment	21 of 856 genes, 2.5%	81 of 16085 genes, 0.5%	2.24e-06	0.00%	0.00	hth, lola, boss, bdg, S, csw, Src64B, rl, pnt, melt, nej, RasGAP1, amon, cdi, alph, msi, Src42A, elB, ey, stan, kay
localization	193 of 856 genes, 22.5%	2407 of 16085 genes, 15.0%	2.25e-06	0.00%	0.00	fz2, Sh, CG1724, Npc1b, dome, Nlg1, kuz, jing, Fs(2)Ket, lilli, CG8177, Vps13D, nAChRalpha2, CG5142, CG31693, CG17646, alpha-Cat, Dys, Cnx99A, Nlg3, crb, pyr, if, Mef2, Sytbeta, GluRIB, cno, Nha2, jvl, lt, Gprk2, mnd, bdg, cv-c, CG33298, pum, unc-13-4A, uif, Mmp2, Src42A, stau, tweek, Snap25, CG1090, bbg, Vha44, ds, dysc, lobo, Ncc69, hth, ft, qua, GluRIA, nrv1, nAChRalpha6, Doa, pHCl-1, FER, S, Ptp61F, MRP, nemy, polyph, PsGEF, ppk15, foxo, Mctp, CG2121, Hk, Scgdelta, neur, mew, tty, Prosap, app, bun, Tao, opa, kay, InR, Mvl, nAChRbeta2, sima, Apoltp, Syt7, Trpm, CG12344, PDZ-GEF, RyR, RhoBTB, Stat92E, Src64B, Btk29A, Sema2a, chinmo, Bsg, CG4662, loco, KrT95D, Cngl, fra, Dbp80, tral, Parp, lola, Sara, NKCC, Fife, abd-A, stl, milt, stai, egh, Grd, Gie, pnt, Elk, Pde6, smog, Msp300, CG41099, rhea, dia, CG32758, AdamTS-A, Debcl, hh, l(2)gl, wdb, Fur1, KCNQ, Grip, CG31121, csw, ex, Rph, X11Lbeta, Rme-8, htt, stan, cta, CG6231, trp, futsch, ced-6, wake, gukh, cora, spir, BicD, nompC, Dhc64C, kibra, rdog, Tie, EcR, Rok, Nrx-1, nej, ATP8B, Ca-alpha1T, Shab, ey, dlg1, step, Drip, SK, par-1, smg, spri, CG7720, slo, KaiR1D, Abl, Mdr50, cac, Dad, Patronin, Dhc62B, Tom40, ths, sfl, Syt1, Ttd14, vn, CG32683, Orco, SerT, cher, ogre, CG42269, pyd
cellular response to chemical stimulus	56 of 856 genes, 6.5%	440 of 16085 genes, 2.7%	2.73e-06	0.00%	0.00	trp, Sh, dome, Sesn, Fs, Duox, kek5, Ptp61F, Hr4, PsGEF, shn, foxo, Eip93F, EcR, pnt, melt, Cnx99A, TyrR, Ggamma30A, Pka-R2, pyr, ACC, Itl, cv-2, step, Sytbeta, InR, sima, TrissinR, mAChR-B, hh, drl, Syt7, Dad, Pde8, wdb, Eip75B, Snoo, Drl-2, csw, Stat92E, Src64B, Tom40, ths, sfl, Dop1R2, Syt1, rl, RasGAP1, Mmp2, Blimp-1, trol, ETHR, msi, Tl, fra
aromatic compound	135 of 856	1520 of 16085	2.79e-06	0.00%	0.00	sbb, CG31183, jing, caup, lilli, Camta, dpy,

biosynthetic process	genes, 15.8%	genes, 9.4%				tna, luna, osa, Atf6, CG12605, CG12054, Mef2, inv, lab, Hcf, Usp10, dsf, scrt, ap, lt, Tlk, Awh, Spt3, Sox21a, Eip75B, Snoo, rut, hng3, crp, CG8312, pum, mamo, Blimp-1, Ac78C, Eip78C, hth, ara, mam, shn, foxo, CG5694, grn, per, CG3726, bun, Spt20, opa, pdm3, kay, sima, Rx, CG12769, Tif-IA, CG11247, nerfin-1, bin3, Stat92E, dsx, chinmo, klu, SCAP, fra, Lim1, Parp, MBD-R2, lola, Smr, Gyc88E, TfAP-2, Fs, vvl, abd-A, nau, MED14, bab2, Eip93F, PyK, Rev1, pnt, melt, tefu, corto, ltl, cv-2, rhea, lmd, ich, Oaz, Dr, ush, Poxm, Antp, ko, CtBP, tara, CG1815, Tet, RunxB, zen, e, kek5, ken, Hr4, kibra, EcR, al, CG34357, e(y)3, nej, upSET, ACXC, Maf1, Rpl135, ey, Oamb, dlg1, smg, CG11883, gpp, Lim3, cic, Dad, sd, crol, RecQ4, Ac3, fs(1)h, en, rl, CG9932, Rbfox1, olf413, Tl
organic cyclic compound biosynthetic process	138 of 856 genes, 16.1%	1565 of 16085 genes, 9.7%	2.83e-06	0.00%	0.00	sbb, CG31183, jing, caup, lilli, Camta, dpy, tna, eIF4EHP, luna, osa, Atf6, CG12605, CG12054, Mef2, inv, lab, Hcf, Usp10, dsf, scrt, ap, lt, Tlk, Awh, Spt3, Sox21a, Eip75B, nvd, Snoo, rut, hng3, crp, CG8312, pum, mamo, Blimp-1, Ac78C, Eip78C, hth, ara, mam, shn, foxo, CG5694, grn, per, CG3726, bun, Spt20, opa, pdm3, kay, sima, Rx, CG12769, Tif-IA, CG11247, nerfin-1, bin3, Stat92E, dsx, chinmo, klu, SCAP, fra, Lim1, Parp, MBD-R2, lola, Smr, Gyc88E, TfAP-2, Fs, vvl, abd-A, nau, MED14, bab2, Eip93F, PyK, Rev1, pnt, melt, tefu, corto, ltl, cv-2, rhea, lmd, ich, Oaz, Dr, ush, Poxm, Antp, ko, CtBP, tara, CG1815, Tet, RunxB, zen, e, kek5, ken, Hr4, kibra, EcR, al, CG34357, e(y)3, nej, ACXC, upSET, Maf1, Rpl135, ey, Oamb, dlg1, smg, CG11883, gpp, Lim3, cic, Dad, sd, crol, CG7470, RecQ4, Ac3, fs(1)h, en, rl, CG9932, Rbfox1, olf413, Tl
modulation of chemical synaptic transmission	28 of 856 genes, 3.3%	141 of 16085 genes, 0.9%	3.02e-06	0.00%	0.00	kek6, Sh, Cbp53E, GluRIA, KaiR1D, slo, Nlg1, Fife, cac, nemy, BicD, cv-c, Mctp, Src64B, pum, Syt1, Dys, rl, Nrx-1, CG32447, Prosap, Nlg3, Src42A, Shab, Snap25, dysc, GluRIB, Sap47
regulation of trans-synaptic signaling	28 of 856 genes, 3.3%	141 of 16085 genes, 0.9%	3.02e-06	0.00%	0.00	kek6, Sh, Cbp53E, GluRIA, KaiR1D, slo, Nlg1, Fife, cac, nemy, BicD, cv-c, Mctp, Src64B, pum, Syt1, Dys, rl, Nrx-1, CG32447, Prosap, Nlg3, Src42A, Shab, Snap25, dysc, GluRIB, Sap47
positive regulation of biosynthetic process	66 of 856 genes, 7.7%	565 of 16085 genes, 3.5%	3.28e-06	0.00%	0.00	MBD-R2, Parp, lola, TfAP-2, vvl, jing, abd-A, nau, caup, lilli, Camta, tna, eIF4EHP, Eip93F, osa, pnt, CG12054, smog, Mef2, lab, Hcf, dsf, ap, lmd, ich, Gprk2, Awh, Oaz, bol, Eip75B, ush, CG8312, pum, Antp, stv, CtBP, tara, stau, hth, Tet, ara, zen, mam, foxo, shn, EcR, e(y)3, nej, ey, CG31612, opa, kay, sima, CG12769, CG11247, gpp, sd, Stat92E, RecQ4, fs(1)h, en, dsx, Rbfox1, Tl, SCAP, Lim1
positive regulation of cellular biosynthetic process	66 of 856 genes, 7.7%	565 of 16085 genes, 3.5%	3.28e-06	0.00%	0.00	MBD-R2, Parp, lola, TfAP-2, vvl, jing, abd-A, nau, caup, lilli, Camta, tna, eIF4EHP, Eip93F, osa, pnt, CG12054, smog, Mef2, lab, Hcf, dsf, ap, lmd, ich, Gprk2, Awh, Oaz, bol, Eip75B, ush, CG8312, pum, Antp, stv, CtBP, tara, stau, hth, Tet, ara, zen, mam, foxo, shn, EcR, e(y)3, nej, ey, CG31612, opa, kay, sima, CG12769, CG11247, gpp, sd, Stat92E, RecQ4, fs(1)h, en, dsx, Rbfox1, Tl, SCAP, Lim1
transmembrane receptor protein	35 of 856 genes, 4.1%	209 of 16085 genes, 1.3%	3.44e-06	0.00%	0.00	boss, PVRAP, FER, S, Ptp61F, foxo, Tie, pnt, melt, pyr, ey, ed, dlg1, step, InR, sima,

tyrosine kinase signaling pathway						hppy, drl, cic, wdb, csw, Drl-2, Src64B, ths, sfl, rl, pum, vn, RasGAP1, Mmp2, trol, cdi, alph, Tl, Src42A
imaginal disc pattern formation	25 of 856 genes, 2.9%	116 of 16085 genes, 0.7%	3.92e-06	0.00%	0.00	sbb, hth, ap, ft, CG8405, jing, abd-A, hh, lilli, Ser, cic, Dad, Dr, shn, en, CG5890, osa, rl, pnt, vn, tara, crb, ds, inv, Lim1
positive regulation of macromolecule metabolic process	85 of 856 genes, 9.9%	821 of 16085 genes, 5.1%	5.41e-06	0.00%	0.00	MBD-R2, lola, TfAP-2, vvl, jing, abd-A, nau, caup, lilli, Camta, tna, Sxl, Eip93F, osa, pnt, CG33144, CG12054, AGO3, hid, Mef2, lab, Hcf, dsf, ap, Imd, ich, Awh, Oaz, bol, Eip75B, CG8312, pum, Antp, stv, sNPF-R, CtBP, tara, Src42A, stau, Tis11, hth, Tet, CG4238, ara, zen, mam, foxo, shn, EcR, e(y)3, nej, Tao, ey, CG31612, Oamb, opa, step, InR, kay, smg, hppy, sima, Hipk, CG8405, hbs, CG12769, ps, Lim3, gpp, CG11247, Pde8, sd, wnd, Stat92E, Src64B, RecQ4, en, fs(1)h, dsx, Rbfox1, vn, SCAP, Tl, pip, Lim1
neuron fate commitment	22 of 856 genes, 2.6%	93 of 16085 genes, 0.6%	5.88e-06	0.00%	0.00	hth, lola, boss, bdg, S, mam, csw, Src64B, rl, pnt, melt, nej, RasGAP1, amon, cdi, alph, msi, Src42A, elB, ey, stan, kay
localization of cell	50 of 856 genes, 5.8%	378 of 16085 genes, 2.4%	5.92e-06	0.00%	0.00	fz2, dome, kuz, jing, abd-A, FER, stl, Dhc64C, kibra, Tie, EcR, stai, mew, egh, pnt, nej, bun, pyr, ey, smog, if, opa, Msp300, kay, par-1, spri, sima, rhea, jvl, AdamTS-A, Abl, hh, Dad, PDZ-GEF, RhoBTB, csw, Stat92E, ex, ths, sfl, pum, Sema2a, vn, Mmp2, Rme-8, Src42A, bbg, fra, cta, lobo
establishment of ommatidial planar polarity	18 of 856 genes, 2.1%	63 of 16085 genes, 0.4%	6.51e-06	0.00%	0.00	Rok, lola, pnt, nej, ft, ec, hbs, caup, ara, bdg, S, sano, ed, stan, CadN2, ds, cno, kay
heterocycle biosynthetic process	134 of 856 genes, 15.7%	1527 of 16085 genes, 9.5%	7.07e-06	0.00%	0.00	sbb, CG31183, jing, caup, lilli, Camta, dpy, tna, luna, osa, Atf6, CG12605, CG12054, Mef2, inv, lab, Hcf, Usp10, dsf, scrt, ap, lt, Tlk, Awh, Spt3, Sox21a, Eip75B, Snoo, rut, hng3, crp, CG8312, pum, mamo, Blimp-1, Ac78C, Eip78C, hth, ara, mam, shn, foxo, CG5694, grn, per, CG3726, bun, Spt20, opa, pdm3, kay, sima, Rx, CG12769, Tif-IA, CG11247, nerfin-1, bin3, Stat92E, dsx, chinmo, klu, SCAP, fra, Lim1, Parp, MBD-R2, lola, Smr, Gyc88E, TfAP-2, Fs, vvl, abd-A, nau, MED14, bab2, Eip93F, PyK, Rev1, pnt, melt, tefu, corto, Itl, cv-2, rhea, Imd, ich, Oaz, Dr, ush, Poxm, Antp, ko, CtBP, tara, CG1815, Tet, RunxB, zen, kek5, ken, Hr4, kibra, EcR, al, CG34357, e(y)3, nej, upSET, ACXC, Maf1, Rpl135, ey, Oamb, dlg1, smg, CG11883, gpp, Lim3, cic, Dad, sd, crol, CG7470, RecQ4, Ac3, fs(1)h, en, rl, CG9932, Rbfox1, Tl
muscle cell differentiation	26 of 856 genes, 3.0%	128 of 16085 genes, 0.8%	7.11e-06	0.00%	0.00	rost, siz, rhea, C3G, Imd, hbs, abd-A, nau, caup, ara, sd, foxo, Rok, CalpA, PyK, Scgdelta, DAAM, kirre, pnt, Bsg, Antp, cher, sns, if, Mef2, InR
locomotory behavior	34 of 856 genes, 4.0%	205 of 16085 genes, 1.3%	7.65e-06	0.00%	0.00	sbb, Sh, DCX-EMAP, dome, TfAP-2, slgA, e, shep, nemy, Gem3, foxo, EcR, CalpA, per, Trim9, nej, Pka-R2, Shab, ey, Pura, Mef2, dlg1, InR, kay, cac, CCKLR-17D1, Ac3, Mbs, Syt1, Sema2a, amon, unc80, tara, dysc
embryonic development via the syncytial blastoderm	34 of 856 genes, 4.0%	206 of 16085 genes, 1.3%	8.71e-06	0.00%	0.00	Npc1b, Sema5c, cora, FER, shn, EcR, Rok, alpha-Cat, chrb, crb, sns, hid, ed, step, dlg1, cno, InR, kay, rhea, dia, Abl, l(2)gl, lncRNA:acal, PDZ-GEF, cv-c, KCNQ, ush, Btk29A, kirre, Mbs, pum, alph, Src42A, pyd
cell motility	49 of 856	372 of 16085	9.88e-06	0.00%	0.00	fz2, dome, kuz, jing, abd-A, FER, stl,

	genes, 5.7%	genes, 2.3%				Dhc64C, kibra, Tie, EcR, stai, mew, egh, pnt, nej, bun, pyr, ey, smog, if, opa, Msp300, kay, par-1, spri, sima, rhea, AdamTS-A, Abl, hh, Dad, PDZ-GEF, RhoBTB, csw, Stat92E, ex, ths, sfl, pum, Sema2a, vn, Mmp2, Rme-8, Src42A, bbg, fra, cta, lobo
compound eye photoreceptor fate commitment	19 of 856 genes, 2.2%	72 of 16085 genes, 0.4%	1.02e-05	0.00%	0.00	hth, lola, boss, bdg, csw, Src64B, rl, pnt, melt, nej, RasGAP1, amon, cdi, alph, msi, Src42A, elB, stan, kay
regulation of growth	43 of 856 genes, 5.0%	304 of 16085 genes, 1.9%	1.05e-05	0.00%	0.00	sbb, futsch, ft, Sesn, kuz, caup, ara, Hr4, foxo, kibra, Rok, chrb, Hs6st, crb, bun, Tao, hid, ey, step, dlg1, InR, Hcf, hppy, sima, Hipk, slo, Abl, cac, l(2)gl, cic, Dad, Src64B, ex, sfl, pum, Sema2a, sNPF-R, Tl, Src42A, stau, pip, stan, dysc
regulation of animal organ morphogenesis	22 of 856 genes, 2.6%	96 of 16085 genes, 0.6%	1.11e-05	0.00%	0.00	par-1, hth, tinc, ec, PDZ-GEF, sd, shn, csw, Rok, Src64B, neur, Mbs, rl, RasGAP1, cdi, klu, alph, Src42A, hid, ed, ds, stan
morphogenesis of a polarized epithelium	25 of 856 genes, 2.9%	122 of 16085 genes, 0.8%	1.18e-05	0.00%	0.00	par-1, lola, ft, ec, hbs, chas, caup, ara, bdg, l(2)gl, S, sano, Dhc64C, Rok, pnt, nej, jbug, app, ed, ds, CadN2, stan, dlg1, cno, kay
positive regulation of macromolecule biosynthetic process	61 of 856 genes, 7.1%	520 of 16085 genes, 3.2%	1.20e-05	0.00%	0.00	MBD-R2, hth, lola, TfAP-2, Tet, vvl, jing, abd-A, nau, caup, ara, lilli, Camta, mam, zen, tna, shn, foxo, Eip93F, EcR, osa, pnt, e(y)3, nej, CG12054, ey, CG31612, Mef2, opa, kay, Hcf, lab, sima, dsf, ap, lmd, ich, CG12769, Awh, gpp, CG11247, Oaz, sd, bol, Eip75B, Stat92E, RecQ4, en, fs(1)h, CG8312, dsx, Rbfox1, pum, Antp, stv, CtBP, SCAP, Tl, tara, stau, Lim1
central nervous system development	42 of 856 genes, 4.9%	295 of 16085 genes, 1.8%	1.31e-05	0.00%	0.00	hth, ced-6, Npc1b, Sema5c, vvl, kuz, jing, mam, PsGEF, Dhc64C, EcR, DAAM, neur, l(3)72Ab, Prosap, bun, robo3, Tao, Mmp1, ey, if, Ten-a, opa, lab, siz, ap, lmd, Abl, hh, drl, Dr, csw, Src64B, en, Nrg, chinmo, vn, Mmp2, spz3, Antp, loco, stan
eye photoreceptor cell fate commitment	19 of 856 genes, 2.2%	73 of 16085 genes, 0.5%	1.31e-05	0.00%	0.00	hth, lola, boss, bdg, csw, Src64B, rl, pnt, melt, nej, RasGAP1, amon, cdi, alph, msi, Src42A, elB, stan, kay
regulation of intracellular signal transduction	47 of 856 genes, 5.5%	354 of 16085 genes, 2.2%	1.59e-05	0.00%	0.00	CG43658, ft, Sesn, CG34393, PVRAP, ken, CG42674, Ptp61F, PsGEF, kibra, melt, tefu, LRR, chrb, crb, Tao, ed, Pura, step, dlg1, InR, cno, par-1, spri, hppy, siz, Hipk, sra, Gprk2, Cdep, Pde8, lncRNA:acal, wdb, cv-c, wnd, RhoGEF64C, ex, Src64B, rut, CG42684, vn, CG15611, alph, sNPF-R, CG30456, Src42A, ds
G protein-coupled receptor signaling pathway	36 of 856 genes, 4.2%	232 of 16085 genes, 1.4%	1.66e-05	0.00%	0.00	trp, boss, Dgk, Camta, CG33639, rdgA, CCHa1-R, foxo, ACXC, TyrR, Ggamma30A, rdgC, smog, Oamb, TrissinR, Proc-R, Oct-TyrR, mAChR-B, Rh7, CCKLR-17D1, CG31760, 5-HT7, Ac3, rut, Dop1R2, CG34384, Dh31-R, CG32683, CG32447, ETHR, Ac78C, sNPF-R, loco, stan, cta, Gprk1
cell proliferation	47 of 856 genes, 5.5%	357 of 16085 genes, 2.2%	2.09e-05	0.00%	0.00	fz2, hth, dome, eIF4B, ft, wake, Doa, Ser, FER, S, Ptp61F, Dhc64C, shn, foxo, kibra, EcR, osa, pnt, crb, bun, pyr, ey, dlg1, InR, cno, dsf, Rbp9, hh, l(2)gl, RyR, sd, Sox21a, Stat92E, Src64B, ex, RecQ4, rl, vn, Mmp2, Xrp1, klu, Tl, loco, Src42A, stau, elB, ds
ovarian follicle cell development	46 of 856 genes, 5.4%	346 of 16085 genes, 2.2%	2.22e-05	0.00%	0.00	Parp, Smr, dome, kuz, jing, spir, Fs(2)Ket, S, mam, stl, Dhc64C, kibra, Tie, EcR, stai, neur, toc, egh, nej, bun, Tao, ed, if, Mef2, lncRNA:flam, dlg1, kay, InR, par-1, spri, rhea, jvl, hh, cic, csw, Stat92E, Src64B, ex, RecQ4, rl, vn, Rme-8, cher, loco, Fas3,

						bbg
columnar/cuboidal epithelial cell development	46 of 856 genes, 5.4%	347 of 16085 genes, 2.2%	2.43e-05	0.00%	0.00	Parp, Smr, dome, kuz, jing, spir, Fs(2)Ket, S, mam, stl, Dhc64C, kibra, Tie, EcR, stai, neur, toc, egh, nej, bun, Tao, ed, if, Mef2, lncRNA:flam, dlg1, kay, InR, par-1, spri, rhea, jvl, hh, cic, csw, Stat92E, Src64B, ex, RecQ4, rl, vn, Rme-8, cher, loco, Fas3, bbg
positive regulation of transcription by RNA polymerase II	46 of 856 genes, 5.4%	347 of 16085 genes, 2.2%	2.43e-05	0.00%	0.00	MBD-R2, hth, TfAP-2, Tet, vvl, jing, abd-A, nau, caup, ara, lilli, Camta, mam, zen, shn, foxo, EcR, pnt, e(y)3, nej, CG12054, ey, Mef2, opa, kay, lab, sima, dsf, ap, lmd, ich, CG12769, Awh, CG11247, sd, Eip75B, Stat92E, en, fs(1)h, CG8312, dsx, Antp, CtBP, SCAP, Tl, Lim1
negative regulation of signal transduction	44 of 856 genes, 5.1%	324 of 16085 genes, 2.0%	2.45e-05	0.00%	0.00	Smr, Sesn, Fs, ken, kek5, Ptp61F, Hr4, foxo, pnt, nej, tefu, LRR, chrb, crb, ltl, ed, par-1, hppy, Hipk, Gprk2, l(2)gl, Dad, CG7094, lncRNA:acal, crol, wdb, cv-c, Snoo, CG42684, pum, uif, RasGAP1, Mmp2, trol, CG32683, cdi, alph, CtBP, Tl, Src42A, ds, pyd, Gprk1, nkd
regulation of neurogenesis	40 of 856 genes, 4.7%	281 of 16085 genes, 1.7%	3.05e-05	0.00%	0.00	hth, lola, vvl, kuz, stl, Dhc64C, kibra, EcR, Rok, DAAM, neur, osa, pnt, Trim9, Prosap, bun, pyr, ed, InR, tinc, Abl, l(2)gl, PDZ-GEF, sd, nerfin-1, cv-c, csw, Src64B, Mbs, rl, Sema2a, RasGAP1, cdi, Antp, klu, alph, Src42A, stan, fra, unk
negative regulation of response to stimulus	49 of 856 genes, 5.7%	385 of 16085 genes, 2.4%	3.07e-05	0.00%	0.00	Smr, Cbp53E, Sesn, Fs, ken, kek5, Ptp61F, Hr4, foxo, Rok, pnt, nej, tefu, LRR, chrb, crb, ltl, ed, par-1, hppy, Hipk, Gprk2, l(2)gl, Dad, CG7094, Pde8, lncRNA:acal, crol, wdb, cv-c, Snoo, Stat92E, CG42684, pum, Sema2a, uif, RasGAP1, Mmp2, cdi, trol, CG32683, alph, CtBP, Tl, Src42A, ds, pyd, Gprk1, nkd
regulation of nervous system development	48 of 856 genes, 5.6%	374 of 16085 genes, 2.3%	3.32e-05	0.00%	0.00	hth, lola, futsch, vvl, kuz, stl, Dhc64C, kibra, EcR, Rok, DAAM, neur, osa, pnt, Trim9, Prosap, bun, pyr, ed, dlg1, InR, tinc, slo, Abl, cac, l(2)gl, Dad, PDZ-GEF, sd, nerfin-1, cv-c, csw, Src64B, pum, Sema2a, Mbs, rl, RasGAP1, cdi, Antp, klu, alph, Src42A, stau, stan, fra, dysc, unk
columnar/cuboidal epithelial cell differentiation	47 of 856 genes, 5.5%	363 of 16085 genes, 2.3%	3.56e-05	0.00%	0.00	Parp, Smr, dome, kuz, jing, spir, Fs(2)Ket, S, mam, stl, Dhc64C, kibra, Tie, EcR, stai, neur, toc, egh, nej, bun, Tao, ed, if, Mef2, lncRNA:flam, dlg1, kay, InR, par-1, spri, rhea, jvl, hh, cic, Sox21a, csw, Stat92E, Src64B, ex, RecQ4, rl, vn, Rme-8, cher, loco, Fas3, bbg
regulation of cellular component organization	83 of 856 genes, 9.7%	827 of 16085 genes, 5.1%	3.56e-05	0.00%	0.00	lola, Sesn, vvl, kuz, nau, lilli, stai, Trim9, smog, if, Ten-a, cno, dia, lt, Tlk, Gprk2, l(2)gl, cv-c, Snoo, Kank, ex, DIP-gamma, pum, mamo, Mmp2, tyn, htt, Src42A, stau, stan, dysc, cta, futsch, CG43658, CG11486, Jupiter, spir, FER, BicD, PsGEF, foxo, Dhc64C, Rok, DAAM, neur, Ten-m, e(y)3, Nrx-1, nej, upSET, Prosap, bun, Tao, ed, ey, dlg1, loh, step, InR, par-1, spri, sima, slo, Abl, cac, Dad, cic, Patronin, PDZ-GEF, crol, RhoBTB, wnd, Stat92E, Src64B, Btk29A, Sema2a, chinmo, kirre, Syt1, cher, Tl, pyd, fra
positive regulation of transcription, DNA-templated	55 of 856 genes, 6.4%	462 of 16085 genes, 2.9%	4.23e-05	0.00%	0.00	MBD-R2, hth, lola, TfAP-2, Tet, vvl, jing, abd-A, nau, caup, ara, lilli, Camta, mam, zen, tna, shn, foxo, Eip93F, EcR, osa, pnt, e(y)3, nej, CG12054, ey, Mef2, opa, kay, Hcf, lab, sima, dsf, ap, lmd, ich, CG12769, Awh, gpp, CG11247, Oaz, sd, Eip75B, Stat92E, en, fs(1)h, CG8312, dsx, Rbfox1,

						Antp, CtBP, SCAP, Tl, tara, Lim1
positive regulation of RNA biosynthetic process	55 of 856 genes, 6.4%	462 of 16085 genes, 2.9%	4.23e-05	0.00%	0.00	MBD-R2, hth, lola, TfAP-2, Tet, vvl, jing, abd-A, nau, caup, ara, lilli, Camta, mam, zen, tna, shn, foxo, Eip93F, EcR, osa, pnt, e(y)3, nej, CG12054, ey, Mef2, opa, kay, Hcf, lab, sima, dsf, ap, lmd, ich, CG12769, Awh, gpp, CG11247, Oaz, sd, Eip75B, Stat92E, en, fs(1)h, CG8312, dsx, Rbfox1, Antp, CtBP, SCAP, Tl, tara, Lim1
positive regulation of nucleic acid-templated transcription	55 of 856 genes, 6.4%	462 of 16085 genes, 2.9%	4.23e-05	0.00%	0.00	MBD-R2, hth, lola, TfAP-2, Tet, vvl, jing, abd-A, nau, caup, ara, lilli, Camta, mam, zen, tna, shn, foxo, Eip93F, EcR, osa, pnt, e(y)3, nej, CG12054, ey, Mef2, opa, kay, Hcf, lab, sima, dsf, ap, lmd, ich, CG12769, Awh, gpp, CG11247, Oaz, sd, Eip75B, Stat92E, en, fs(1)h, CG8312, dsx, Rbfox1, Antp, CtBP, SCAP, Tl, tara, Lim1
morphogenesis of embryonic epithelium	25 of 856 genes, 2.9%	130 of 16085 genes, 0.8%	4.59e-05	0.00%	0.00	Npc1b, Abl, cora, l(2)gl, FER, PDZ-GEF, lncRNA:acal, cv-c, shn, Rok, ush, Btk29A, alpha-Cat, Mbs, Mmp2, alph, crb, Src42A, Mmp1, ed, pyd, dlg1, step, kay, cno
gland development	31 of 856 genes, 3.6%	189 of 16085 genes, 1.2%	4.96e-05	0.00%	0.00	fz2, hth, lola, IP3K2, dia, kuz, AdamTS-A, drl, Ser, l(2)gl, l(3)psg2, shn, Drl-2, Stat92E, EcR, Src64B, ush, neur, mew, Btk29A, ths, rl, pnt, Antp, cher, crb, Src42A, pyr, hid, if, fra
negative regulation of macromolecule biosynthetic process	57 of 856 genes, 6.7%	490 of 16085 genes, 3.0%	5.29e-05	0.00%	0.00	sbb, Smr, TfAP-2, jing, abd-A, Hr4, eIF4EHP, Sxl, shn, foxo, al, EcR, per, e(y)3, upSET, CG12605, tefu, Maf1, corto, dlg1, Oamb, smg, heph, Usp10, rhea, scrt, lmd, ich, Tlk, gpp, CG11247, Oaz, cic, crol, sd, nerfin-1, Eip75B, bin3, bru2, ush, en, fs(1)h, dsx, Rbfox1, CG9932, pum, tut, Blimp-1, Antp, klu, CtBP, msi, bru3, Eip78C, timeout, Tis11, Lim1
negative regulation of cellular macromolecule biosynthetic process	57 of 856 genes, 6.7%	490 of 16085 genes, 3.0%	5.29e-05	0.00%	0.00	sbb, Smr, TfAP-2, jing, abd-A, Hr4, eIF4EHP, Sxl, shn, foxo, al, EcR, per, e(y)3, upSET, CG12605, tefu, Maf1, corto, dlg1, Oamb, smg, heph, Usp10, rhea, scrt, lmd, ich, Tlk, gpp, CG11247, Oaz, cic, crol, sd, nerfin-1, Eip75B, bin3, bru2, ush, en, fs(1)h, dsx, Rbfox1, CG9932, pum, tut, Blimp-1, Antp, klu, CtBP, msi, bru3, Eip78C, timeout, Tis11, Lim1
leg disc development	22 of 856 genes, 2.6%	104 of 16085 genes, 0.6%	5.37e-05	0.00%	0.00	hth, CG43658, ap, TfAP-2, jing, hh, Ser, CG42674, l(3)psg2, sd, al, RhoGEF64C, bab2, osa, e(y)3, rl, pnt, vn, CG30456, ds, Pura, Lim1
dorsal closure	23 of 856 genes, 2.7%	113 of 16085 genes, 0.7%	5.57e-05	0.00%	0.00	Npc1b, Abl, cora, l(2)gl, FER, lncRNA:acal, PDZ-GEF, cv-c, shn, Rok, ush, Btk29A, alpha-Cat, Mbs, alph, crb, Src42A, ed, pyd, dlg1, step, kay, cno
response to endogenous stimulus	39 of 856 genes, 4.6%	276 of 16085 genes, 1.7%	5.70e-05	0.00%	0.00	Sh, Sesn, Fs, kek5, Ptp61F, Hr4, shn, foxo, EcR, pnt, melt, TyrR, pyr, hid, ltl, cv-2, step, InR, sima, TrissinR, mAChR-B, Dad, l(2)gl, wdb, l(3)psg2, Eip75B, Snoo, csw, Src64B, ths, sfl, Dop1R2, rl, Mmp2, Blimp-1, trol, ETHR, Tl, fra
cellular response to endogenous stimulus	36 of 856 genes, 4.2%	243 of 16085 genes, 1.5%	5.75e-05	0.00%	0.00	Sh, Sesn, Fs, kek5, Ptp61F, Hr4, shn, foxo, EcR, pnt, melt, TyrR, pyr, ltl, cv-2, step, InR, sima, TrissinR, mAChR-B, Dad, wdb, Eip75B, Snoo, csw, Src64B, ths, Dop1R2, sfl, rl, Mmp2, Blimp-1, trol, ETHR, Tl, fra
embryo development ending in birth or egg hatching	34 of 856 genes, 4.0%	222 of 16085 genes, 1.4%	6.10e-05	0.00%	0.00	Npc1b, Sema5c, cora, FER, shn, EcR, Rok, alpha-Cat, chrb, crb, sns, hid, ed, step, dlg1, cno, InR, kay, rhea, dia, Abl, l(2)gl, lncRNA:acal, PDZ-GEF, cv-c, KCNQ, ush, Btk29A, kirre, Mbs, pum, alph, Src42A, pyd

negative regulation of biosynthetic process	58 of 856 genes, 6.8%	505 of 16085 genes, 3.1%	6.27e-05	0.00%	0.00	sbb, Smr, TfAP-2, jing, abd-A, e, Hr4, eIF4EHP, Sxl, shn, foxo, al, EcR, per, e(y)3, upSET, CG12605, tefu, Maf1, corto, dlg1, Oamb, smg, heph, Usp10, rhea, scrt, lmd, ich, Tlk, gpp, CG11247, Oaz, cic, crol, sd, nerfin-1, Eip75B, bin3, bru2, ush, en, fs(1)h, dsx, Rbfox1, CG9932, pum, tut, Blimp-1, Antp, klu, CtBP, msi, bru3, Eip78C, timeout, Tis11, Lim1
negative regulation of cellular biosynthetic process	58 of 856 genes, 6.8%	505 of 16085 genes, 3.1%	6.27e-05	0.00%	0.00	sbb, Smr, TfAP-2, jing, abd-A, e, Hr4, eIF4EHP, Sxl, shn, foxo, al, EcR, per, e(y)3, upSET, CG12605, tefu, Maf1, corto, dlg1, Oamb, smg, heph, Usp10, rhea, scrt, lmd, ich, Tlk, gpp, CG11247, Oaz, cic, crol, sd, nerfin-1, Eip75B, bin3, bru2, ush, en, fs(1)h, dsx, Rbfox1, CG9932, pum, tut, Blimp-1, Antp, klu, CtBP, msi, bru3, Eip78C, timeout, Tis11, Lim1
striated muscle tissue development	12 of 856 genes, 1.4%	31 of 16085 genes, 0.2%	6.53e-05	0.00%	0.00	Scgdelta, vkg, kirre, lmd, kuz, hbs, abd-A, nau, sd, Mef2, Msp300, EcR
positive regulation of gene expression	62 of 856 genes, 7.2%	565 of 16085 genes, 3.5%	0.00011	0.00%	0.00	MBD-R2, hth, lola, TfAP-2, Tet, vvl, jing, abd-A, nau, caup, ara, lilli, Camta, mam, zen, tna, Sxl, shn, foxo, Eip93F, EcR, osa, pnt, e(y)3, nej, CG12054, ey, Mef2, opa, kay, Hcf, lab, sima, dsf, Hipk, ap, lmd, ich, CG12769, ps, Awh, Lim3, gpp, CG11247, Oaz, sd, bol, Eip75B, Stat92E, en, fs(1)h, CG8312, dsx, Rbfox1, pum, Antp, CtBP, SCAP, Tl, tara, stau, Lim1
imaginal disc-derived wing vein specification	16 of 856 genes, 1.9%	59 of 16085 genes, 0.4%	0.00011	0.00%	0.00	jing, hh, caup, ara, S, cic, en, sfl, Dys, rl, Rbfox1, corto, ltl, inv, cv-2, step
negative regulation of nitrogen compound metabolic process	69 of 856 genes, 8.1%	658 of 16085 genes, 4.1%	0.00011	0.00%	0.00	sbb, Smr, TfAP-2, jing, abd-A, eIF4EHP, Sxl, CG12605, tefu, crb, corto, Usp10, rhea, scrt, lmd, ich, Tlk, hh, Oaz, l(2)gl, wdb, bol, Eip75B, Fancm, ush, CG8312, pum, Blimp-1, Antp, alph, CtBP, msi, Eip78C, bru3, timeout, Tis11, Ptp61F, Hr4, foxo, shn, EcR, al, per, e(y)3, upSET, Maf1, Oamb, dlg1, smg, heph, CG11247, gpp, cic, Dad, lncRNA:acal, nerfin-1, sd, crol, bin3, bru2, fs(1)h, en, dsx, chinmo, CG9932, Rbfox1, tut, klu, Lim1
regulation of cell proliferation	34 of 856 genes, 4.0%	229 of 16085 genes, 1.4%	0.00013	0.00%	0.00	fz2, hth, dome, ft, Doa, FER, Ptp61F, foxo, kibra, EcR, osa, pnt, crb, bun, pyr, ey, dlg1, InR, dsf, hh, l(2)gl, sd, RyR, Stat92E, Src64B, ex, RecQ4, rl, vn, Mmp2, Xrp1, klu, Tl, Src42A
cellular response to organic substance	43 of 856 genes, 5.0%	331 of 16085 genes, 2.1%	0.00013	0.00%	0.00	Sh, dome, Sesn, Fs, Duox, kek5, Ptp61F, Hr4, shn, foxo, EcR, pnt, melt, Cnx99A, TyrR, Pka-R2, pyr, ACC, ltl, cv-2, step, InR, sima, TrissinR, mAChR-B, Dad, wdb, Eip75B, Snoo, csw, Stat92E, Src64B, ths, sfl, Dop1R2, rl, RasGAP1, Mmp2, Blimp-1, trol, ETHR, Tl, fra
negative regulation of cellular metabolic process	72 of 856 genes, 8.4%	702 of 16085 genes, 4.4%	0.00014	0.00%	0.00	sbb, Smr, TfAP-2, jing, abd-A, eIF4EHP, Sxl, CG12605, tefu, crb, corto, Usp10, rhea, scrt, lmd, ich, Tlk, hh, Oaz, l(2)gl, wdb, bol, Eip75B, Fancm, ush, CG8312, pum, Blimp-1, Antp, alph, CtBP, msi, Eip78C, bru3, timeout, Tis11, e, Ptp61F, Hr4, foxo, shn, EcR, al, per, e(y)3, upSET, Maf1, Oamb, dlg1, InR, smg, heph, CG11247, gpp, cic, Dad, lncRNA:acal, nerfin-1, sd, crol, bin3, bru2, fs(1)h, en, dsx, chinmo, rl, CG9932, Rbfox1, tut, klu, Lim1
head development	27 of 856 genes, 3.2%	157 of 16085 genes, 1.0%	0.00014	0.00%	0.00	lab, hth, ced-6, Sema5c, vvl, jing, hh, drl, PsGEF, Dhc64C, Dr, EcR, Src64B, DAAM, en, Nrg, chinmo, alpha-Cat, vn, Antp,

						Prosap, bun, robo3, Tao, ey, stan, Ten-a
regulation of developmental growth	34 of 856 genes, 4.0%	231 of 16085 genes, 1.4%	0.00016	0.00%	0.00	sbb, futsch, ft, kuz, Hr4, foxo, kibra, Rok, Hs6st, crb, Tao, hid, ey, step, dlg1, InR, Hipk, slo, Abl, Dad, l(2)gl, cac, Src64B, ex, sfl, Sema2a, pum, sNPF-R, Tl, stau, Src42A, pip, stan, dysc
positive regulation of multicellular organismal process	39 of 856 genes, 4.6%	287 of 16085 genes, 1.8%	0.00016	0.00%	0.00	hth, Sh, ec, wake, kuz, abd-A, Hr4, eIF4EHP, Dhc64C, Tie, EcR, Hk, pnt, bgm, Prosap, bun, pyr, Shab, ey, step, dlg1, Oamb, kay, InR, spri, Hipk, cac, nerfin-1, Src64B, rl, vn, RasGAP1, Rme-8, klu, sNPF-R, Src42A, stau, stan, tral
supramolecular fiber organization	35 of 856 genes, 4.1%	243 of 16085 genes, 1.5%	0.00018	0.00%	0.00	CG43658, futsch, qua, Jupiter, spir, FER, PsGEF, Dhc64C, Rok, stai, Scgdelta, DAAM, mew, Ten-m, ed, if, Msp300, Sb, f, rhea, C3G, dia, jvl, Abl, Patronin, RhoBTB, Kank, Src64B, Btk29A, chinmo, Bsg, cdi, cher, tyn, Src42A
establishment of planar polarity	22 of 856 genes, 2.6%	111 of 16085 genes, 0.7%	0.00018	0.00%	0.00	par-1, lola, ft, ec, hbs, chas, caup, ara, bdg, S, sano, Rok, pnt, nej, jbug, app, ed, ds, CadN2, stan, cno, kay
establishment of tissue polarity	22 of 856 genes, 2.6%	111 of 16085 genes, 0.7%	0.00018	0.00%	0.00	par-1, lola, ft, ec, hbs, chas, caup, ara, bdg, S, sano, Rok, pnt, nej, jbug, app, ed, ds, CadN2, stan, cno, kay
cytoskeleton organization	67 of 856 genes, 7.8%	639 of 16085 genes, 4.0%	0.00018	0.00%	0.00	DCX-EMAP, lilli, stai, egh, alpha-Cat, crb, sns, smog, if, Msp300, cno, Sb, rhea, dia, C3G, jvl, l(2)gl, wdb, cv-c, Kank, tyn, htt, Src42A, RhoGAP18B, ds, cta, futsch, CG43658, ft, wake, qua, gukh, Jupiter, spir, cora, FER, BicD, PsGEF, Wdr62, foxo, Dhc64C, Scgdelta, DAAM, Rok, mew, Ten-m, ed, dlg1, step, CG43897, par-1, heph, siz, f, CG42319, Abl, Unc-115a, Patronin, RhoBTB, Src64B, Btk29A, chinmo, Bsg, cdi, cher, loco, tral
cellular process	521 of 856 genes, 60.9%	8391 of 16085 genes, 52.2%	0.00021	0.00%	0.00	Sh, CG10185, CG9492, lilli, rdgA, tna, CG17646, l(3)72Ab, Atf6, Ggamma30A, CG12054, hid, if, GluRIB, lab, Hcf, CG12009, dsf, scrt, C3G, lt, dpr6, CG7094, CG7378, Eip75B, nvd, CG8312, pum, Pka-C3, mamo, Dh31-R, rgn, trol, ETHR, stv, alph, Ac78C, msi, Snap25, ds, dysc, kek6, Doa, FER, S, nemy, PsGEF, CCHa1-R, CG5694, DAAM, CG43143, CG3726, HP1Lcsd, Prosap, Tao, DNApol-epsilon255, ed, opa, sima, f, Cda4, Rx, Tif-IA, CG12769, Dhc36C, wnd, Stat92E, Src64B, Btk29A, cdi, loco, NKCC, eIF5B, dpr12, mbl, DIP-zeta, dpr9, Grd, Gie, jbug, PRL-1, smog, cv-2, lncRNA:flam, Msp300, Sap47, CG42342, CG32758, lmd, ich, disco-r, Oaz, l(2)gl, Dr, beat-Ic, Poxm, Rph, Mbs, axo, htt, bru3, CG1815, trp, futsch, Cbp53E, CG11486, RunxB, gukh, Jupiter, cora, spir, beat-IIa, CG14669, BicD, EcR, Nrx-1, upSET, CG13197, Ca-alpha1T, ACC, Pura, dlg1, spri, eRF3, Hipk, slo, Lim3, Unc-115a, Pde8, Dad, sd, Dhc62B, Cht10, RecQ4, Gyf, fs(1)h, Rbfox1, CG30463, Tl, Gbs-70E, PpD5, dome, CG31183, ec, CG34393, kuz, jing, CG8177, Vps13D, Camta, dpy, eIF4EHP, luna, CG33144, CG12605, TyrR, Gnf1, crb, Mef2, ap, Nha2, jvl, Tlk, Gprk2, Spt3, CG42748, sano, Sox21a, crp, CG15160, unc-13-4A, CG30456, S6KL, dpr13, timeout, Tis11, Ncc69, CG43658, mtg, ft, nrv1, CAH1, Ser, Gem3, foxo, Tob, mew, toc, bgm, CG17514, app, Pka-R2, bun, Spt20, loh, wry, pdm3, mAChR-B, Trpm, PDZ-GEF, l(3)psg2, nerfin-1, 5-HT7,

						chinmo, Bsg, elB, pip, Cngl, l(1)G0196, Lim1, nkd, Sara, vkg, vvl, PVRAP, Dgk, Eip93F, dpr1, milt, PyK, Ccn, tefu, corto, Itl, Sb, dia, AdamTS-A, PH4alphaEFB, Fur1, Grip, ush, Rme-8, Asator, ko, beat-Va, ckn, cta, ken, kek5, zen, Dhc64C, Rok, ATP8B, chrb, CG31612, CG4629, SkpE, smg, CG11883, CG42319, CG7470, Tom40, en, sfl, Dop1R2, CG9932, Ttd14, vn, Pde11, Fas3, ogre, pyd, Gprk1, fz2, CG1724, Nlg1, Fs(2)Ket, nAChRalpha2, Sxl, CG5921, osa, CG9395, sns, Ten-a, cno, Sytbeta, Ptp99A, Mur89F, poe, Oct-TyrR, sra, snky, CG3339, cv-c, Snoo, rut, DIP-gamma, hng3, uif, Mmp2, amon, Blimp-1, Eip78C, Src42A, bbg, CG1090, lobo, hth, eIF4B, GluRIA, CPT2, sas, Ptp61F, Mctp, Hk, Scgdelta, beat-Vc, neur, per, tRNA:Ser-AGA-1-1, InR, nAChRbeta2, DIP-theta, snRNA:U2:14B, RyR, RhoBTB, bin3, Cht7, CG6701, Rchy1, fra, tral, MBD-R2, Smr, Gyc88E, Fs, Fife, dpr8, Duox, MED14, stl, bab2, egh, melt, Elk, rdgC, Pde6, hang, CG41099, esn, pot, Pde1c, CG7236, bol, ex, CG15611, Xrp1, Antp, Men, Nc73EF, beat-VI, wake, Tet, sff, Mur2B, Wdr62, Ten-m, RluA-1, e(y)3, Shab, Mmp1, ey, Oamb, step, SK, CG43897, par-1, heph, TrissinR, Proc-R, Abl, ps, gpp, cic, CCKLR-17D1, cac, lncRNA:acal, crol, RhoGEF64C, Ac3, Gr28b, ths, kirre, rl, Syt1, CG32683, cher, olf413, unk, sbb, DCX-EMAP, Sesn, Slc25A46, caup, CG42674, CG5142, Dys, alpha-Cat, Trim9, Cnx99A, Nlg3, CG7544, pyr, robo3, AGO3, inv, CG11030, CG10947, Usp10, dtr, CG33110, Awh, bdg, alpha-Man-Ia, Hacd1, Kank, Fancm, CG33298, CG42684, Nrg, CG34384, CG32447, beat-IIb, beat-Ib, stau, kl-2, CG13251, CG40178, nAChRalpha6, qua, thw, slgA, ara, pHCl-1, mam, shep, CG33639, LManIII, shn, grn, CadN2, COX4L, kay, CG31145, hppy, tinc, Cdep, Syt7, CG11247, CG12344, dpr2, CG31760, dsx, Sema2a, klu, SCAP, Parp, lola, boss, CG9674, TfAP-2, abd-A, nau, CG31523, CalpA, stai, Rev1, pnt, LRR, Hs6st, CG9003, Usp32, DIP-epsilon, Rbp6, rost, mei-P26, rhea, Debcl, hh, Rbp9, drl, wdb, KCNQ, csw, Drl-2, RasGAP1, spz3, X11Lbeta, CtBP, sNPF-R, tara, tyn, stan, RhoGAP18B, ced-6, IP3K2, CG4238, e, Hr4, nompC, kibra, Tie, al, CG34357, nej, ACXC, Maf1, Rpl135, Keap1, siz, KaiR1D, CG8405, hbs, Trxr-2, Rh7, Patronin, bru2, beat-IIIb, CG32085, tut, CG5758
actin filament bundle assembly	12 of 856 genes, 1.4%	34 of 16085 genes, 0.2%	0.00021	0.00%	0.00	Rok, DAAM, Src64B, CG43658, f, jvl, Src42A, FER, ed, if, RhoBTB, Sb
cell junction organization	19 of 856 genes, 2.2%	86 of 16085 genes, 0.5%	0.00023	0.00%	0.00	cora, l(2)gl, CG42748, PDZ-GEF, Rok, Src64B, Nrg, kirre, alpha-Cat, rl, Mmp2, crb, loco, sns, Src42A, Mmp1, CadN2, pyd, dlg1
negative regulation of multicellular organismal process	32 of 856 genes, 3.7%	213 of 16085 genes, 1.3%	0.00024	0.00%	0.00	ft, Doa, Ser, stl, shn, foxo, kibra, EcR, neur, Grd, osa, pnt, crb, Tao, ed, dlg1, InR, Abl, Dad, l(2)gl, CG12344, sd, Src64B, ex, Dop1R2, Sema2a, RasGAP1, cdi, cher, alph, Tl, Src42A
striated muscle cell differentiation	22 of 856 genes, 2.6%	113 of 16085 genes, 0.7%	0.00025	0.00%	0.00	rost, siz, rhea, C3G, lmd, hbs, nau, sd, foxo, Rok, CalpA, Scgdelta, DAAM, PyK, kirre, pnt, Bsg, cher, sns, if, Mef2, InR
muscle tissue	12 of 856	35 of 16085	0.00031	0.00%	0.00	Scgdelta, vkg, kirre, lmd, kuz, hbs, abd-A,

development	genes, 1.4%	genes, 0.2%				nau, sd, Mef2, Msp300, EcR
regulation of neuron differentiation	30 of 856 genes, 3.5%	194 of 16085 genes, 1.2%	0.00032	0.00%	0.00	hth, lola, tinc, vvl, Abl, kuz, PDZ-GEF, nerfin-1, cv-c, Dhc64C, csw, EcR, Rok, Src64B, DAAM, neur, Sema2a, Mbs, rl, pnt, RasGAP1, Trim9, cdi, Prosap, alph, Src42A, ed, fra, stan, InR
brain development	24 of 856 genes, 2.8%	135 of 16085 genes, 0.8%	0.00041	0.00%	0.00	lab, hth, ced-6, Sema5c, vvl, hh, drl, PsGEF, Dhc64C, Dr, EcR, Src64B, DAAM, en, Nrg, chinmo, vn, Prosap, bun, Tao, robo3, ey, Ten-a, stan
protein phosphorylation	50 of 856 genes, 5.8%	432 of 16085 genes, 2.7%	0.00046	0.00%	0.00	CG31183, Doa, sff, FER, Ptp61F, Tie, Rok, CG34357, per, CG43143, tefu, Pka-R2, Tao, CG4629, step, cno, InR, kay, par-1, hppy, CG31145, Hipk, Tlk, Gprk2, Abl, drl, l(2)gl, Dad, CG7094, Pde8, lncRNA:acal, PDZ-GEF, CG7236, wdb, wnd, Drl-2, Src64B, Btk29A, CG8312, chinmo, rl, Pka-C3, vn, Asator, cdi, alph, sNPF-R, Src42A, S6KL, Gprk1
response to drug	32 of 856 genes, 3.7%	220 of 16085 genes, 1.4%	0.00053	0.00%	0.00	hppy, Sh, mAChR-B, slo, Syt7, S, Pde8, alpha-Man-Ia, RyR, PsGEF, dpr9, EcR, rut, Dop1R2, per, pum, Syt1, rl, Nrx-1, tefu, trol, Orco, Ggamma30A, Pka-R2, Ac78C, cher, Gbs-70E, Tao, hang, dlg1, Sytbeta, InR
developmental process involved in reproduction	90 of 856 genes, 10.5%	980 of 16085 genes, 6.1%	0.00053	0.00%	0.00	fz2, Parp, lola, Smr, dome, TfAP-2, kuz, jing, abd-A, Fs(2)Ket, stl, Sxl, milt, bab2, stai, egh, tefu, robo3, sns, hid, if, Mef2, lncRNA:flam, Msp300, rhea, mei-P26, poe, dia, dsf, jvl, Gprk2, Debcl, Rbp9, hh, l(2)gl, bol, Eip75B, Dr, csw, ex, pum, Rme-8, msi, stau, bbg, qua, Tet, Doa, spir, ken, mam, S, BicD, kibra, Dhc64C, EcR, Tie, Rok, toc, neur, Sox100B, nej, bun, Tao, ed, dlg1, InR, kay, par-1, spri, heph, sima, Abl, cic, PDZ-GEF, Stat92E, Src64B, RecQ4, en, Btk29A, dsx, Rbfox1, chinmo, rl, Syt1, vn, tut, cher, loco, Fas3
developmental growth involved in morphogenesis	21 of 856 genes, 2.5%	109 of 16085 genes, 0.7%	0.00060	0.00%	0.00	fz2, spri, Sh, Abl, kuz, disco-r, FER, Dad, wnd, al, Rok, DAAM, rut, Nrg, Sema2a, Mbs, Mmp1, CadN2, stan, kay, cno
actin filament bundle organization	12 of 856 genes, 1.4%	37 of 16085 genes, 0.2%	0.00063	0.00%	0.00	Rok, DAAM, Src64B, CG43658, f, jvl, Src42A, FER, ed, if, RhoBTB, Sb
leg disc morphogenesis	18 of 856 genes, 2.1%	83 of 16085 genes, 0.5%	0.00068	0.00%	0.00	hth, CG43658, ap, TfAP-2, hh, Ser, CG42674, sd, l(3)psg2, al, RhoGEF64C, bab2, e(y)3, osa, CG30456, ds, Pura, Lim1
cyclic nucleotide metabolic process	11 of 856 genes, 1.3%	31 of 16085 genes, 0.2%	0.00071	0.00%	0.00	CG34357, Ac3, rut, Gyc88E, CG31183, ACXC, Pde11, Ac78C, Pde1c, Pde8, Pde6
negative regulation of developmental process	32 of 856 genes, 3.7%	223 of 16085 genes, 1.4%	0.00072	0.00%	0.00	ft, Ser, stl, shn, foxo, kibra, EcR, neur, osa, pnt, crb, bun, Tao, ed, dlg1, InR, Abl, Dad, l(2)gl, sd, Sox21a, Stat92E, Src64B, ex, ush, Sema2a, RasGAP1, cdi, cher, alph, Tl, Src42A
negative regulation of growth	20 of 856 genes, 2.3%	101 of 16085 genes, 0.6%	0.00073	0.00%	0.00	sima, ft, Sesn, Abl, caup, ara, l(2)gl, cic, Dad, foxo, kibra, ex, Src64B, Sema2a, chrb, crb, Tl, Src42A, Tao, dlg1
actin filament organization	25 of 856 genes, 2.9%	149 of 16085 genes, 0.9%	0.00074	0.00%	0.00	CG43658, f, dia, qua, jvl, Abl, spir, FER, PsGEF, RhoBTB, Kank, Rok, Src64B, DAAM, mew, Btk29A, chinmo, cdi, cher, tyn, Src42A, ed, if, Msp300, Sb
synapse assembly	27 of 856 genes, 3.2%	170 of 16085 genes, 1.1%	0.00079	0.00%	0.00	par-1, futsch, mtg, slo, Nlg1, Abl, Fife, drl, cac, Dad, Src64B, stai, Ten-m, pum, Gie, Nrx-1, nej, Prosap, Nlg3, Tl, Src42A, stau, Ten-a, stan, dysc, dlg1, kay
positive regulation of intracellular signal transduction	28 of 856 genes, 3.3%	181 of 16085 genes, 1.1%	0.00084	0.00%	0.00	hppy, CG43658, siz, ft, CG34393, Gprk2, PVRAP, Pde8, CG42674, PsGEF, wnd, kibra, RhoGEF64C, ex, Src64B, melt, vn, CG15611, sNPF-R, crb, CG30456,

						Src42A, Tao, ed, Pura, ds, step, InR
positive regulation of developmental process	37 of 856 genes, 4.3%	283 of 16085 genes, 1.8%	0.00096	0.00%	0.00	hth, ec, kuz, abd-A, nau, Hr4, eIF4EHP, Dhc64C, foxo, Tie, EcR, DAAM, pnt, Prosap, bun, pyr, hid, ey, step, dlg1, InR, kay, spri, Hipk, cac, nerfin-1, Src64B, rl, vn, RasGAP1, Rme-8, klu, sNPF-R, stau, Src42A, stan, tral
stem cell differentiation	20 of 856 genes, 2.3%	103 of 16085 genes, 0.6%	0.00102	0.00%	0.00	Sara, Rbp6, kuz, abd-A, S, Dr, bab2, Stat92E, EcR, en, osa, pnt, Sema2a, vn, Antp, klu, stau, pyr, inv, nkd
ameboidal-type cell migration	30 of 856 genes, 3.5%	205 of 16085 genes, 1.3%	0.00110	0.00%	0.00	par-1, spri, rhea, dome, kuz, jing, AdamTS-A, hh, Dhc64C, kibra, csw, Tie, Stat92E, EcR, stai, ex, ths, sfl, egh, Sema2a, pnt, vn, Rme-8, bun, pyr, bbg, smog, if, cta, kay
cAMP metabolic process	6 of 856 genes, 0.7%	8 of 16085 genes, 0.0%	0.00141	0.00%	0.00	Ac78C, Pde1c, Ac3, rut, Pde8, Pde11
cellular response to growth factor stimulus	19 of 856 genes, 2.2%	98 of 16085 genes, 0.6%	0.00203	0.00%	0.00	Fs, kek5, Dad, Ptp61F, shn, Snoo, csw, Src64B, ths, sfl, rl, pnt, RasGAP1, Mmp2, trol, pyr, Itl, fra, cv-2
negative regulation of nucleobase-containing compound metabolic process	50 of 856 genes, 5.8%	454 of 16085 genes, 2.8%	0.00209	0.00%	0.00	sbb, Smr, TfAP-2, jing, abd-A, Hr4, Sxl, shn, foxo, al, EcR, per, e(y)3, upSET, CG12605, tefu, Maf1, corto, dlg1, Oamb, Usp10, rhea, scrt, Imd, ich, Tlk, gpp, CG11247, Oaz, cic, crol, sd, nerfin-1, bol, Eip75B, bin3, Fancm, ush, en, fs(1)h, dsx, CG9932, pum, Blimp-1, Antp, klu, CtBP, Eip78C, timeout, Lim1
cell growth	25 of 856 genes, 2.9%	158 of 16085 genes, 1.0%	0.00230	0.00%	0.00	fz2, spri, sima, Sh, poe, Sesn, kuz, FER, cic, Dad, wnd, foxo, DAAM, rut, Nrg, Sema2a, Mbs, Tl, bun, ey, CadN2, stan, InR, cno, kay
negative regulation of RNA metabolic process	48 of 856 genes, 5.6%	430 of 16085 genes, 2.7%	0.00235	0.00%	0.00	sbb, Smr, TfAP-2, jing, abd-A, Hr4, Sxl, shn, foxo, al, EcR, per, e(y)3, upSET, CG12605, tefu, Maf1, corto, dlg1, Oamb, Usp10, rhea, scrt, Imd, ich, Tlk, gpp, CG11247, Oaz, cic, crol, sd, nerfin-1, bol, Eip75B, bin3, ush, en, fs(1)h, dsx, CG9932, pum, Blimp-1, Antp, klu, CtBP, Eip78C, Lim1
response to growth factor	19 of 856 genes, 2.2%	99 of 16085 genes, 0.6%	0.00239	0.00%	0.00	Fs, kek5, Dad, Ptp61F, shn, Snoo, csw, Src64B, ths, sfl, rl, pnt, RasGAP1, Mmp2, trol, pyr, Itl, fra, cv-2
regulation of circadian rhythm	18 of 856 genes, 2.1%	90 of 16085 genes, 0.6%	0.00244	0.00%	0.00	Sh, TfAP-2, wake, Rh7, CG12344, Hk, EcR, per, Grd, Dop1R2, Mbs, bgm, timeout, Shab, hid, Pura, Oamb, InR
response to oxygen-containing compound	42 of 856 genes, 4.9%	355 of 16085 genes, 2.2%	0.00249	0.00%	0.00	Sh, boss, Gyc88E, Sesn, S, Duox, Ptp61F, Hr4, foxo, dpr9, EcR, PyK, per, melt, Ggamma30A, Pka-R2, Tao, ACC, hid, hang, step, dlg1, InR, hppy, sima, mAChR-B, l(2)gl, Pde8, wdb, l(3)psg2, Eip75B, Stat92E, rut, Dop1R2, pum, rl, Blimp-1, Orco, Ac78C, cher, Tl, Gbs-70E
plasmatocyte differentiation	9 of 856 genes, 1.1%	23 of 16085 genes, 0.1%	0.00337	0.00%	0.00	ush, neur, ths, pnt, rl, cher, pyr, Ser, Stat92E
learning or memory	26 of 856 genes, 3.0%	172 of 16085 genes, 1.1%	0.00344	0.00%	0.00	trp, Sh, futsch, dome, Abl, sra, drl, lilli, nemy, wnd, EcR, Stat92E, Src64B, Tob, neur, Btk29A, rut, per, Rbfox1, pum, vn, Nrx-1, cher, stau, Oamb, Sap47
cognition	26 of 856 genes, 3.0%	172 of 16085 genes, 1.1%	0.00344	0.00%	0.00	trp, Sh, futsch, dome, Abl, sra, drl, lilli, nemy, wnd, EcR, Stat92E, Src64B, Tob, neur, Btk29A, rut, per, Rbfox1, pum, vn, Nrx-1, cher, stau, Oamb, Sap47
homeostatic process	53 of 856 genes, 6.2%	503 of 16085 genes, 3.1%	0.00383	0.00%	0.00	trp, Sara, boss, Cbp53E, CG40178, NKCC, nrv1, nAChRalpha6, CPT2, mbl, CG8177, rdgA, foxo, EcR, PyK, mew, per, Dys, Cnx99A, bgm, tefu, crb, bun, Tao, ey, if, Oamb, Drip, InR, Mvl, hppy, rhea, KaiR1D,

						Debcl, Trxr-2, Trpm, cac, CCKLR-17D1, RyR, cv-c, CG30377, ush, Gyf, Dop1R2, Ttd14, uif, amon, unc80, Src42A, CG1090, fra, dysc, Ncc69
regulation of behavior	19 of 856 genes, 2.2%	102 of 16085 genes, 0.6%	0.00384	0.00%	0.00	Sh, TfAP-2, wake, foxo, Hk, EcR, rut, Dop1R2, egh, per, Mbs, bgm, Trim9, sNPF-R, Shab, Pura, Oamb, InR, Drip
regulation of photoreceptor cell differentiation	12 of 856 genes, 1.4%	43 of 16085 genes, 0.3%	0.00392	0.00%	0.00	Src64B, neur, rl, Mbs, RasGAP1, tinc, cdi, alph, Src42A, PDZ-GEF, ed, csw
negative regulation of transcription, DNA-templated	46 of 856 genes, 5.4%	412 of 16085 genes, 2.6%	0.00394	0.00%	0.00	sbb, Smr, TfAP-2, jing, abd-A, Hr4, shn, foxo, al, EcR, per, e(y)3, upSET, CG12605, tefu, Maf1, corto, dlg1, Oamb, Usp10, rhea, scrt, lmd, ich, Tlk, gpp, CG11247, Oaz, cic, crol, sd, nerfin-1, Eip75B, bin3, ush, en, fs(1)h, dsx, CG9932, pum, Blimp-1, Antp, klu, CtBP, Eip78C, Lim1
negative regulation of RNA biosynthetic process	46 of 856 genes, 5.4%	412 of 16085 genes, 2.6%	0.00394	0.00%	0.00	sbb, Smr, TfAP-2, jing, abd-A, Hr4, shn, foxo, al, EcR, per, e(y)3, upSET, CG12605, tefu, Maf1, corto, dlg1, Oamb, Usp10, rhea, scrt, lmd, ich, Tlk, gpp, CG11247, Oaz, cic, crol, sd, nerfin-1, Eip75B, bin3, ush, en, fs(1)h, dsx, CG9932, pum, Blimp-1, Antp, klu, CtBP, Eip78C, Lim1
negative regulation of nucleic acid-templated transcription	46 of 856 genes, 5.4%	412 of 16085 genes, 2.6%	0.00394	0.00%	0.00	sbb, Smr, TfAP-2, jing, abd-A, Hr4, shn, foxo, al, EcR, per, e(y)3, upSET, CG12605, tefu, Maf1, corto, dlg1, Oamb, Usp10, rhea, scrt, lmd, ich, Tlk, gpp, CG11247, Oaz, cic, crol, sd, nerfin-1, Eip75B, bin3, ush, en, fs(1)h, dsx, CG9932, pum, Blimp-1, Antp, klu, CtBP, Eip78C, Lim1
wing disc pattern formation	17 of 856 genes, 2.0%	84 of 16085 genes, 0.5%	0.00410	0.00%	0.00	sbb, ap, CG8405, hh, lilli, Ser, cic, Dr, shn, en, CG5890, pnt, osa, crb, tara, inv, ds
organ growth	17 of 856 genes, 2.0%	84 of 16085 genes, 0.5%	0.00410	0.00%	0.00	sbb, Hipk, ft, hh, l(2)gl, Sxl, kibra, foxo, ex, sfl, Hs6st, crb, Tao, hid, DNApol-epsilon255, dlg1, InR
digestive tract development	20 of 856 genes, 2.3%	112 of 16085 genes, 0.7%	0.00411	0.00%	0.00	lab, vkg, dome, abd-A, hh, S, Dad, Sox21a, cv-c, shn, Stat92E, EcR, mew, pnt, vn, Antp, crb, if, Mef2, opa
digestive system development	20 of 856 genes, 2.3%	112 of 16085 genes, 0.7%	0.00411	0.00%	0.00	lab, vkg, dome, abd-A, hh, S, Dad, Sox21a, cv-c, shn, Stat92E, EcR, mew, pnt, vn, Antp, crb, if, Mef2, opa
positive regulation of hippo signaling	8 of 856 genes, 0.9%	18 of 16085 genes, 0.1%	0.00418	0.00%	0.00	ex, hppy, ft, crb, Tao, ed, ds, kibra
skeletal muscle organ development	13 of 856 genes, 1.5%	51 of 16085 genes, 0.3%	0.00449	0.00%	0.00	mew, vkg, rhea, C3G, lmd, kuz, Tsp, drl, nau, if, Mef2, Grip, Msp300
larval lymph gland hemocyte differentiation	9 of 856 genes, 1.1%	24 of 16085 genes, 0.1%	0.00515	0.00%	0.00	ush, neur, ths, pnt, rl, cher, pyr, Ser, Stat92E
cyclic-nucleotide-mediated signaling	15 of 856 genes, 1.8%	68 of 16085 genes, 0.4%	0.00534	0.00%	0.00	5-HT7, CG34357, Ac3, rut, Dop1R2, Gyc88E, CG31183, ACXC, TyrR, Oct-TyrR, Gprk2, Pka-R2, Ac78C, hang, Cngl
regulation of synaptic plasticity	11 of 856 genes, 1.3%	37 of 16085 genes, 0.2%	0.00537	0.00%	0.00	Src64B, kek6, Cbp53E, Dys, Fife, Prosap, Src42A, nemy, Snap25, Mctp, Sap47
R7 cell differentiation	14 of 856 genes, 1.6%	60 of 16085 genes, 0.4%	0.00571	0.00%	0.00	Src64B, lola, boss, Ten-m, pnt, rl, RasGAP1, nej, cdi, klu, alph, Src42A, CadN2, csw
dorsal/ventral pattern formation	24 of 856 genes, 2.8%	156 of 16085 genes, 1.0%	0.00633	0.00%	0.00	Parp, sbb, ap, jvl, CG8405, lilli, S, cic, BicD, Dhc64C, Dr, shn, csw, milt, CalpA, CG5890, osa, pnt, Tl, tara, crb, loco, pip, tral
epidermal growth factor receptor signaling pathway	15 of 856 genes, 1.8%	69 of 16085 genes, 0.4%	0.00650	0.00%	0.00	Src64B, hppy, pum, pnt, rl, RasGAP1, vn, Src42A, S, cic, Ptp61F, ed, dlg1, step, csw
ERBB signaling pathway	15 of 856 genes, 1.8%	69 of 16085 genes, 0.4%	0.00650	0.00%	0.00	Src64B, hppy, pum, pnt, rl, RasGAP1, vn, Src42A, S, cic, Ptp61F, ed, dlg1, step, csw

regulation of transport	35 of 856 genes, 4.1%	282 of 16085 genes, 1.8%	0.00671	0.00%	0.00	Fife, Fs(2)Ket, lilli, S, nemy, PsGEF, BicD, Mctp, CG2121, Hk, neur, Dys, Cnx99A, Nrx-1, Prosap, crb, Tao, Pde6, step, Sytbeta, InR, Mvl, spri, Apoltp, Gprk2, Abl, lt, hh, Syt7, cac, Ttd14, Syt1, pyd, dysc, tral
response to ammonium ion	8 of 856 genes, 0.9%	19 of 16085 genes, 0.1%	0.00689	0.00%	0.00	Pka-R2, Sh, Dop1R2, per, Tao, mAChR-B, InR, EcR
syncytium formation by plasma membrane fusion	13 of 856 genes, 1.5%	53 of 16085 genes, 0.3%	0.00718	0.00%	0.00	Rok, CalpA, PyK, rost, siz, kirre, lmd, hbs, nau, sns, Mef2, foxo, InR
syncytium formation	13 of 856 genes, 1.5%	53 of 16085 genes, 0.3%	0.00718	0.00%	0.00	Rok, CalpA, PyK, rost, siz, kirre, lmd, hbs, nau, sns, Mef2, foxo, InR
myoblast fusion	13 of 856 genes, 1.5%	53 of 16085 genes, 0.3%	0.00718	0.00%	0.00	Rok, CalpA, PyK, rost, siz, kirre, lmd, hbs, nau, sns, Mef2, foxo, InR
cell-cell fusion	13 of 856 genes, 1.5%	53 of 16085 genes, 0.3%	0.00718	0.00%	0.00	Rok, CalpA, PyK, rost, siz, kirre, lmd, hbs, nau, sns, Mef2, foxo, InR
photoreceptor cell development	20 of 856 genes, 2.3%	116 of 16085 genes, 0.7%	0.00724	0.00%	0.00	lola, boss, tinc, Doa, mbl, lilli, PDZ-GEF, CG5921, csw, EcR, neur, Ten-m, Mbs, pnt, Bsg, crb, CG13251, CadN2, stan, dysc
negative regulation of macromolecule metabolic process	76 of 856 genes, 8.9%	836 of 16085 genes, 5.2%	0.00735	0.00%	0.00	sbb, Smr, TfAP-2, jing, abd-A, eIF4EHP, Sxl, CG12605, tefu, crb, AGO3, corto, Usp10, rhea, scrt, lmd, ich, Tlk, hh, Oaz, l(2)gl, wdb, bol, Eip75B, Dr, Fancm, ush, CG8312, pum, Blimp-1, Antp, alph, CtBP, msi, Eip78C, bru3, timeout, Tis11, CG11486, ft, Ptp61F, Hr4, foxo, shn, EcR, al, per, e(y)3, upSET, Maf1, Oamb, dlg1, smg, heph, CG11247, gpp, cic, Dad, lncRNA:acal, nerfin-1, sd, Achl, crol, bin3, bru2, CG6701, fs(1)h, en, dsx, Rbfox1, chinmo, CG9932, tut, klu, elB, Lim1
imaginal disc-derived leg morphogenesis	16 of 856 genes, 1.9%	79 of 16085 genes, 0.5%	0.00817	0.00%	0.00	hth, CG43658, ap, TfAP-2, Ser, CG42674, sd, l(3)psg2, al, RhoGEF64C, e(y)3, osa, CG30456, ds, Pura, Lim1
somatic muscle development	18 of 856 genes, 2.1%	98 of 16085 genes, 0.6%	0.00889	0.00%	0.00	hth, lola, rhea, ap, C3G, lmd, cora, nau, sd, foxo, Stat92E, PyK, ths, crp, Poxm, Tl, pyr, InR
negative regulation of metabolic process	79 of 856 genes, 9.2%	885 of 16085 genes, 5.5%	0.00928	0.00%	0.00	sbb, Smr, TfAP-2, jing, abd-A, eIF4EHP, Sxl, CG12605, tefu, crb, AGO3, corto, Usp10, rhea, scrt, lmd, ich, Tlk, hh, Oaz, l(2)gl, wdb, bol, Eip75B, Dr, Fancm, ush, CG8312, pum, Blimp-1, Antp, alph, CtBP, msi, Eip78C, bru3, timeout, Tis11, CG11486, ft, e, Ptp61F, Hr4, foxo, shn, EcR, al, per, e(y)3, upSET, Maf1, Oamb, dlg1, InR, smg, heph, CG11247, gpp, cic, Dad, lncRNA:acal, crol, sd, nerfin-1, Achl, bin3, bru2, en, CG6701, fs(1)h, dsx, Rbfox1, CG9932, chinmo, rl, tut, klu, elB, Lim1
central complex development	6 of 856 genes, 0.7%	10 of 16085 genes, 0.1%	0.00965	0.00%	0.00	Nrg, robo3, Tao, Sema5c, ey, Ten-a
regulation of cytoskeleton organization	24 of 856 genes, 2.8%	160 of 16085 genes, 1.0%	0.00996	0.00%	0.00	CG43658, futsch, dia, Abl, Jupiter, spir, lilli, Patronin, PsGEF, Dhc64C, RhoBTB, Kank, Rok, stai, Src64B, DAAM, Btk29A, chinmo, cher, ed, smog, if, cta, step

Table S10. The results of RNA-Seq before (HS-) and after (HS+) heat shock treatment of S2 cells for the selected 588 rDNA-contacting genes. Excel file attached separately.

Table S11. The overlap between 274 4C-decreased genes, 913 4C-increased genes, and 42 downregulated genes. Related to the Venn diagram in Fig. 6B.

Names	total	elements
274 4C decreased padj down-42	7	CG3764 Sema1a Nipped-A pico CG18135 Frl PlexA
913-4C-increased padj down-42	5	Hers kto mrj sti MESR3
274 4C decreased	267	rdx Dhc93AB serp tou zfh1 CG34383 CG32459 CG2201 corn ct RhoGAP102A Crtc CG42340 CG13203 CG34370 bru1 BG642163 CG9005 lncRNA:CR34047 Egfr salr lncRNA:CR44544 lncRNA:CR44917 CG14115 CG31999 haf CG9926 BtbVII CG32264 CG30158 hdc mgl Toll-7 Pvf2 Calx lncRNA:CR45261 CG3906 nmo CG1888 Myo81F pros CG41434 Glut1 fru CG10175 eg plum Strn-Mlck lncRNA:CR44945 caps sdt Liprin-alpha lncRNA:CR45735 Dhc98D CG13912 Tgi CG10413 Atf3 lncRNA:CR45641 kek3 CrebA nrv2 mmy CR46160 Octbeta1R Tg Ddr Pkn CG13698 lncRNA:CR44780 GluRIIC Tom CG7140 CG15258 ssp5 lncRNA:CR44506 red CG5404 otk2 Frq1 Pka-C1 Atpalpha CG4213 apt sona LanB1 lncRNA:CR45148 Dap160 Nep1 CG43078 kl-5 stx Pkcdelta Ktl shakB CG43736 Samuel fz nolo pxb CG34347 lncRNA:CR44184 CG4229 Glut4EF nAChRalpha4 mir-280 mtt CG12164 pHCl-2 Lkr lncRNA:CR45439 smal CG1909 Osi8 Nlg4 CG14073 wech CG11319 CG7381 CG42750 klar wg RpS26 OtopLc CG7365 bnl lncRNA:CR45580 CG40439 betaTub97EF CG8180 lncRNA:CR31386 Pax spin Itp-r83A SNF4Agamma dac CG14370 Octbeta2R Shroom Abd-B CG2082 odd unc-5 tai igl trv Zasp52 CrzR lncRNA:CR43807 slow FoxL1 Skeletor Nedd4 psq CG6767 Ire1 oys DIP-beta fry Acsl hb Mid1 slou wun CG32006 Eph lncRNA:CR45362 lncRNA:CR44634 toy snRNA:U3 RunxA CG46301 CG7065 shot MsR2 Or43a kek2 RapGAP1 Con CR43360 Raf CG31869 CG46302 Nckx30C bma Atg17 cwo 5-HT1A Aps neo lncRNA:CR44938 CadN dpr17 Sobp CG1504 CG4074 ss TyrRII Oat Tollo px fipi CG16791 CG32395 CG17839 CG9837 lncRNA:CR44833 tgy 28SrRNA-Psi:CR40596 gus TpnC41C lncRNA:CR43304 CG12438 Appl Dnah3 CG32694 CG33669 RhoGAP100F nab NetA lncRNA:CR44500 nAChRalpha7 lncRNA:CR44602 Sdc side CG7031 CG31637 IA-2 CG13743 CG15820 svr yellow-e CG44956 CG15186 CG9400 CG42389 chp Gfrl pan CG10019 CG10000 lncRNA:CR45668 Cpr50Ca CG11453 lncRNA:CR44730 CG13954 lncRNA:CR45632 scrib MYPT-75D Dg Mical GEFmeso Gbp3 Syp flw Myo10A CG4168 Ac76E inaC CG43366 CG6051 CG1695 GluClalpha lncRNA:CR44344 lncRNA:CR45423 Svil CG30438
913-4C-increased	908	e(y)3 CG42674 DIP-theta Oamb ome SKIP Mmp2 Orco lncRNA:CR46197 CG42324 cic stv siz Sap47 dtr CG2258 axo Fer1HCH CG9003 lncRNA:CR44399 CG13197 CG10185 bru3 CG31176 Mctp kl-2 Shab lncRNA:CR44997 ft Tao trol CG4744 dpr2 cno cdc14 Fs CG6959 GluRIB stan CG43050 CG5142 AdamTS-A loh CG13251 Rpl135 CG43759 lncRNA:CR44530 CG42598 Mvl pdm3 lncRNA:CR43492 lncRNA:CR45543 C3G CG14892 RhoGEF64C KrT95D lncRNA:CR45123 egh CG32447 Gnf1 PRL-1 lncRNA:CR45741 Awh CG31324 dpr12 CLIP-190 RhoBTB Ir41a CG9395 CG7094 CG9664 Ten-m RunxB CG11865 dlg1 lncRNA:acal CG34251 dpr1 mmd Sema5c CG30463 CG34393 tna lncRNA:CR45519 CG33523 CG7720 CG14506 TrissinR Sodh-1 beat-IIa lncRNA:CR44603 Mmp1 CG4374 thw CG9572 CadN2 nAChRalpha3 nemy tinc bab2 CG45782 slo vn Prosap CG17646 ex CG33144 kek5 CG1724 lncRNA:CR32835 CG30389 CG14967 lncRNA:CR43769 hth CG8476 amon lncRNA:CR44315 CG34259 CG33923 sbb Dad Teh1 Dbp80 lncRNA:CR46215 CG9171 Hcf Rchy1 CG4393 polyph Fbp1 Blimp-1 jvl Sb Hs6st cv-c Elk CG44837 Kank Lmpt CG17716 Ir40a CG31145 CtBP beat-VII Or67d Cyp4e3 jbug CG4520 Ccp84Ac CG12769 fs(1)h sr CG16800 rut lncRNA:CR44269 Slc25A46 lncRNA:CR43453 CG6244 CG31050 eIF5B sd CG14692 OtopLb CG43897 DIP-gamma CG31294 abd-A CG13810 lncRNA:CR44755 Hk Sesn Snoo ens Usp32 dpr8 CG15803 CG15160 NKCC mew CG42851 Jon44E hng3 lncRNA:CR44633 28SrRNA-Psi:CR45851 ap lncRNA:CR45347 gukh mbl Stat92E eRF3 CR41320 htt CG14947 CG14082 MED14 lncRNA:CR44320 boss COX4L Scgdelta CG13405 wake ds lncRNA:CR44735 CG4341 sano RluA-1 CG7692 opa CG14960 CG41099 stau DCX-EMAP nw sra per CG42339 ckd lncRNA:CR43485 sns lncRNA:CR44110 nAChRbeta2 beat-Vc BicD loco lncRNA:CR44785 CCHa1-R jing EcR Su(Ste):CR42414 lncRNA:CR46113 CG11247 Ten-a CG17047 Gem3 Eip75B bgm CG40178 alpha-Est10 e inaE beat-Ib chinmo CG31808 Ac3 Pka-R2 frm CG4467 Ncc69 bchs Duox Ady43A CG40006 CG32700 uif unk CG12605 lilli beat-IIIb CG31475 CG8740 CG6398 CG33639 side-VIII CG11041 Nha2 Maf1 SerT CG12054 CG13833 Cda4 lmd CG41284 zen bdg PH4alphaEFB CG10126 inv CG17684 KCNQ Pde11 nAChRalpha6 Oct-TyrR T48 Dys S6KL CG31038 CG11284 Fas3 lncRNA:CR45245 CG15544 tty CG42684 timeout mam kibra Src64B CG40472 CG43778 lncRNA:CR44313 CG6701 dpy Tie CG31663 Osi17 crb CG5758 chas Sema2b beat-IIb DIP-epsilon msi CG11317 CG12541 l(1)G0196 CG15611 CG14070 InR lobo Syt7 CG42747 kay CG45080 hbs sens-2 CG6836 Tob ed Vps13D CG10947 Tet Tsp CG2162 smg lola fred CG30048 bin3 scrt sm CG33110 CG8405 CG11030 rgn nkd Xrp1 Neurochondrin lncRNA:CR44685 Tif-IA shep CG6040 lncRNA:CR44902 CG10839 Hacd1 Cdep ko nerfin-1 cta CG43915 CG13003 Gyf side-II Unc-115a Dhc62B upSET ara CG11486 CCKLR-17D1 Rme-8 CG14457 Mur2B CG9265 corto lncRNA:CR44197 DIP-zeta CG2121 CG34398 Ac78C pot Hasp lncRNA:CR45266 foxo wdb CG12589 CG32645 Irk2 lncRNA:CR45336 CG17574 toc Nrx-1 ATP8B milt CG33143 Spt20 Ccn Proc-R lncRNA:CR45411 Keap1 eIF4B CG4691 lncRNA:CR45276 CG4073 CG42319 nompC kirre PDZ-GEF CG17571 grn CG42404 CG5966 lncRNA:CR45689 CG42749 Corin E(spl)m6-BFM Gprk2 TyrR lncRNA:CR44750 futsch CG31183 lncRNA:CR45377 lncRNA:CR46006 CG14395 HP1Lcsd lncRNA:CR44339 CPT2 nej Obp56d Patronin Rev1 pip Camta CG3726 lncRNA:CR44980 lncRNA:CR44337 CG4238 CG9674 CG30456 Cpr50Cb CG12009 CG10348 CG18747 Rx Nc73EF bbg Trxr-2 Npc1b nahoda Eip78C lncRNA:CR45335 pre-rRNA:CR45847 cher lncRNA:CR44909 CG7470 sff CG3339 app wnd CG9492 DAAM alpha-Cat Mbs Sh KaiR1D lncRNA:CR43763 CG42335 CG42232 Pde8 Btk29A CG34357 Gr28b CR40068 side-III CG42524 lab CG32473 unc80 wry pyd Sytbeta CG6024 nAChRalpha2 Dgk vib side-VI hid Tom40 l(2)41Ab CG31998 mei-P26 CG34386 lncRNA:CR44494 lncRNA:CR46075 lncRNA:CR46004 lncRNA:CR43334 ORY Fancm scro Hr4 CG6163

		PpD5 CG42269 Dh31-R CG43373 lncRNA:CR44931 CG4502 spz3 l(3)psg2 trp CG42680 Cyp6a14 Ubx rl Rbp6 PsGEF Parp CG1090 CG5888 CG42673 LManIII TwdlQ CG42795 CG16965 Grd LRP1 CG34384 smog CG4650 lncRNA:CR13130 lncRNA:CR44786 Meltrin Apoltp MBD-R2 LRR step mesh CG15631 CG44475 Fs(2)Ket lncRNA:CR45308 pnt CG43143 CG7991 Grip CG44426 CG31693 CG14535 Gr23a bru2 jp CG5694 caup CG32085 RecQ4 CG31688 CG13898 ppk15 CG3635 Vha44 Drip lncRNA:CR45750 Ptp99A lncRNA:CR45228 CG32816 CG32206 SK elB S beat-IV CG31882 CG43658 dsx kuz asRNA:CR44894 asRNA:CR45909 PyK ths ps CG4297 CG8086 shn ACC lncRNA:CR44486 28SrRNA-Psi:CR45848 dpr9 mnd chrb CG43427 Gcn2 CG43729 CG7548 tefu CG18812 ey pyr slgA spir Rfx Abl nrv1 cnc CG1815 Dr Men lncRNA:CR46212 CG45781 CG15040 CG4662 Myo28B1 CG8854 Mur89F Trim9 snRNA:U2:14B Ttd14 SkpE spri TfAP-2 Dop1R2 tara unc-13-4A lncRNA:CR44350 tweek vvl Aldh-III Mef2 lncRNA:CR43848 fz2 lncRNA:CR45447 Pde1c Pka-C3 lncRNA:CR44349 FER lncRNA:CR45579 sas CG44838 CG40160 zormin Hipk Usp10 Sxl esn lncRNA:CR44231 pwn Gbs-70E RhoGAP18B CG7236 Syt1 CG32432 PVRAP Cnx99A Wdr62 RasGAP1 Tlk CG31206 tut robo3 Piezo ogre Gie CG32683 nau Achl klu CG4629 CG34351 CG6044 CG5921 Cbp53E CG6678 hh ltl fra DNApol-epsilon255 vkg cdi neur lncRNA:CR44776 Smr CG41378 CG33298 5-HT7 l(3)72Ab CG15594 ced-6 dome Dhc64C CG5910 CG17514 CG7530 dpr21 sima ckn lncRNA:CR43627 Pgant2 rdgA CG31612 beat-VI pHCl-1 CG16826 csw CG12344 CG33467 Drl-2 CG9815 CG42852 CG42748 lncRNA:CR44443 rdog Osi24 CG42663 ich rost chic ETHR l(2)gl CG7294 Tl Ref2 Pzl CG6231 Sema2a Snap25 beat-Ic Fur1 CalpA Cngl X11Lbeta CG11883 lncRNA:CR44525 lncRNA:CR44621 Pura sty CG5890 lncRNA:CR44737 tyn CG42326 CG13699 bun lncRNA:CR44686 sNPF-R CG5973 Oaz lncRNA:CR44801 Rim ken bol Bsg gpp CG32758 al Sara CG34353 nvy CG43861 lncRNA:CR42836 stai pum alpha-Man-Ia Traf4 beat-Va CG34354 Rbp9 dsf Prip CG14669 lbl CG5004 CG8177 Tsp96F dpr13 CG30377 Cht7 CG3376 sfl Ggamma30A CG7378 Gprk1 CG5892 Src42A osa CG9932 spen Spt3 Nlg1 Rph lncRNA:CR45622 lov en Dhc36C Lim3 Mdr50 lncRNA:CR44754 lncRNA:CR44609 crol dpr6 CG12994 Gyc88E hppy cora Rbfox1 melt CG31523 lncRNA:flam CG7251 cac CG15395 CR34311 Rh7 if CG42342 Sox100B CG4577 mtg lncRNA:CR44552 side-V CG43313 CG15498 MRP Msp300 CG4998 CG8312 par-1 dia stl CG45263 Debcl Jupiter CG10178 tral CG10919 Pino eIF4EHP CR1075 Atf6 alph GluRIA mAChR-B Lim1 Tis11 Fife Doa lncRNA:CR45267 CG43337 CG8945 hang Pde6 kek6 Rok IP3K2 Cht10 lncRNA:CR43270 snky CG18586 Trpm SCAP Eip93F qua luna ec CG31121 heph gish Bruce lncRNA:CR45576 tRNA:Ser-AGA-1-1 crp nvd osp ACXC Ser CG32198 Sox21a drl Nrg Asator olf413 ZnT63C poe CG31741 Ca-alpha1T CG13784 Gr22e rhea Mhcl Osi6 CG31760 ush disco-r CG7544 CG12814 CG17129 lt Antp AGO3 CG6142 Poxm Pld lncRNA:CR45334 Ptp61F RyR dysc lncRNA:CR45054 cpo f rdgC mamo Nlg3 cv-2 CAH1 CG12516
padj down-42	30	HERC2 ERp60 CG7289 Syx18 THADA Tango4 ste24a CG10623 lncRNA:CR45039 scra hay MED25 CG18273 BTBD9 CG15211 Tango1 CG5098 OstDelta gry CG16762 CG9663 Hph CG2186 RtcB Cdk2 PRAS40 Prp8 Got2 CG44085 CG13185

Table S12. GO associations with Biological Process (GO Profiler) of 42 rDNA-contacting genes shown in the Venn diagram in Fig. 6B. The genes were downregulated after heat shock treatment.

GO.ID	Description	padj	Genes
GO:2000305	semaphorin-plexin signaling pathway involved in regulation of photoreceptor cell axon guidance	0.008254721900982972	SEMA1A,PLEXA
GO:0009987	cellular process	0.025069109437893252	SEMA1A,NIPPED-A,PICO,CG18135,FRL,PLEXA,HERS,KTO,MRJ,STI,MESR3,HERC2,ERP60,SYX18,THADA,TANGO4,STE24A,CG10623,SCRA,HAY,MED25,BTBD9,TANGO1,CG5098,OSTDELTA,GRY,HPH,RTCB,CDK2,PRAS40,PRP8,GOT2,CG44085,CG13185

Table S13. The overlapping between 274 4C-decreased genes, 913 4C-increased genes, and 60 upregulated genes. Related to the Venn diagram in Fig. 6*C*.

Names	total	Elements

274 4C decreased 60 upregulated RNA-Seq	19	mgl Tgi Glut4EF wech OtopLc bnl spin tai Zasp52 Nedd4 CG6767 Mid1 slou cwo Aps Sdc lncRNA:CR45668 Mical Syp
913-4C-increased 60 upregulated RNA-Seq	5	CG9171 lncRNA:CR43334 CG43729 cnc chic
274 4C decreased	255	rdx Dhc93AB serp tou zfh1 CG34383 CG32459 CG2201 corn ct RhoGAP102A Crtc CG42340 CG13203 CG34370 bru1 BG642163 CG9005 lncRNA:CR34047 CG3764 Egfr salr lncRNA:CR44544 lncRNA:CR44917 CG14115 CG31999 haf CG9926 BtbVII CG32264 CG30158 hdc Toll-7 Pvf2 Calx lncRNA:CR45261 CG3906 nmo CG1888 Myo81F pros CG41434 Glut1 fru CG10175 eg plum Strn-Mlck lncRNA:CR44945 caps sdt Sema1a Liprin-alpha lncRNA:CR45735 Dhc98D CG13912 CG10413 Atf3 lncRNA:CR45641 kek3 CrebA nrv2 mmy CR46160 Octbeta1R Tg Ddr Pkn CG13698 lncRNA:CR44780 GluRIIC Nipped-A Tom CG7140 CG15258 ssp5 lncRNA:CR44506 red CG5404 otk2 Frq1 Pka-C1 Atpalpha CG4213 apt sona LanB1 lncRNA:CR45148 Dap160 Nep1 CG43078 kl-5 stx Pkcdelta Ktl shakB CG43736 Samuel fz nolo pxb CG34347 lncRNA:CR44184 CG4229 nAChRalpha4 mir-280 mtt CG12164 pHCl-2 Lkr lncRNA:CR45439 smal CG1909 Osi8 Nlg4 CG14073 CG11319 CG7381 CG42750 klar wg RpS26 CG7365 lncRNA:CR45580 CG40439 betaTub97EF CG8180 lncRNA:CR31386 Pax Itp-r83A SNF4Agamma dac CG14370 Octbeta2R Shroom Abd-B CG2082 odd unc-5 igl pico trv CrzR lncRNA:CR43807 slow FoxL1 Skeletor psq Ire1 oys CG18135 DIP-beta fry Acsl hb Frl wun CG32006 Eph lncRNA:CR45362 lncRNA:CR44634 toy snRNA:U3 RunxA CG46301 CG7065 shot MsR2 Or43a kek2 RapGAP1 Con CR43360 Raf CG31869 CG46302 Nckx30C bma Atg17 5-HT1A neo lncRNA:CR44938 CadN dpr17 PlexA Sobp CG1504 CG4074 ss TyrRII Oat Tollo px fipi CG16791 CG32395 CG17839 CG9837 lncRNA:CR44833 tgy 28SrRNA-Psi:CR40596 gus TpnC41C lncRNA:CR43304 CG12438 Appl Dnah3 CG32694 CG33669 RhoGAP100F nab NetA lncRNA:CR44500 nAChRalpha7 lncRNA:CR44602 side CG7031 CG31637 IA-2 CG13743 CG15820 svr yellow-e CG44956 CG15186 CG9400 CG42389 chp Gfrl pan CG10019 CG10000 Cpr50Ca CG11453 lncRNA:CR44730 CG13954 lncRNA:CR45632 scrib MYPT-75D Dg GEFmeso Gbp3 flw Myo10A CG4168 Ac76E inaC CG43366 CG6051 CG1695 GluClalpha lncRNA:CR44344 lncRNA:CR45423 Svil CG30438
913-4C-increased	908	e(y)3 CG42674 DIP-theta Oamb ome SKIP Mmp2 Orco lncRNA:CR46197 CG42324 cic stv siz Sap47 dtr CG2258 axo Fer1HCH CG9003 lncRNA:CR44399 CG13197 CG10185 bru3 CG31176 Mctp kl-2 Shab lncRNA:CR44997 ft Tao trol CG4744 dpr2 cno cdc14 Fs CG6959 GluRIB stan CG43050 CG5142 Hers AdamTS-A loh CG13251 Rpl135 CG43759 lncRNA:CR44530 CG42598 Mvl pdm3 lncRNA:CR43492 lncRNA:CR45543 C3G CG14892 RhoGEF64C KrT95D lncRNA:CR45123 egh CG32447 Gnf1 PRL-1 lncRNA:CR45741 Awh CG31324 dpr12 CLIP-190 RhoBTB Ir41a CG9395 CG7094 CG9664 Ten-m RunxB CG11865 dlg1 lncRNA:acal CG34251 dpr1 mmd Sema5c CG30463 CG34393 tna lncRNA:CR45519 CG33523 CG7720 CG14506 TrissinR Sodh-1 beat-IIa lncRNA:CR44603 Mmp1 CG4374 thw CG9572 CadN2 nAChRalpha3 nemy tinc bab2 CG45782 slo vn Prosap CG17646 ex CG33144 kek5 CG1724 lncRNA:CR32835 CG30389 CG14967 lncRNA:CR43769 hth CG8476 amon lncRNA:CR44315 CG34259 CG33923 sbb Dad Teh1 Dbp80 lncRNA:CR46215 Hcf Rchy1 CG4393 polyph Fbp1 Blimp-1 jvl Sb Hs6st cv-c Elk CG44837 Kank Lmpt CG17716 Ir40a CG31145 CtBP beat-VII Or67d Cyp4e3 jbug CG4520 Ccp84Ac CG12769 fs(1)h sr CG16800 rut lncRNA:CR44269 Slc25A46 lncRNA:CR43453 CG6244 CG31050 eIF5B sd CG14692 OtopLb CG43897 DIP-gamma CG31294 abd-A CG13810 lncRNA:CR44755 Hk Sesn Snoo ens Usp32 dpr8 CG15803 CG15160 NKCC mew CG42851 Jon44E hng3 lncRNA:CR44633 28SrRNA-Psi:CR45851 ap lncRNA:CR45347 gukh mbl Stat92E eRF3 CR41320 htt CG14947 CG14082 MED14 lncRNA:CR44320 boss COX4L Scgdelta CG13405 wake ds lncRNA:CR44735 CG4341 sano RluA-1 CG7692 opa CG14960 CG41099 stau DCX-EMAP nw sra per CG42339 ckd lncRNA:CR43485 sns lncRNA:CR44110 nAChRbeta2 beat-Vc BicD loco lncRNA:CR44785 CCHa1-R jing EcR Su(Ste):CR42414 lncRNA:CR46113 CG11247 Ten-a CG17047 Gem3 Eip75B bgm CG40178 alpha-Est10 e inaE beat-Ib chinmo CG31808 Ac3 Pka-R2 frm CG4467 Ncc69 bchs Duox Ady43A CG40006 CG32700 uif unk CG12605 lilli beat-IIIb CG31475 CG8740 CG6398 CG33639 side-VIII CG11041 Nha2 Maf1 SerT CG12054 CG13833 Cda4 lmd CG41284 zen bdg PH4alphaEFB CG10126 inv CG17684 KCNQ Pde11 nAChRalpha6 Oct-TyrR T48 Dys S6KL CG31038 CG11284 Fas3 lncRNA:CR45245 CG15544 tty CG42684 timeout mam kibra Src64B CG40472 CG43778 lncRNA:CR44313 CG6701 dpy Tie CG31663 Osi17 crb CG5758 chas Sema2b beat-IIb DIP-epsilon msi CG11317 CG12541 l(1)G0196 CG15611 CG14070 InR lobo Syt7 CG42747 kay CG45080 hbs sens-2 CG6836 Tob ed Vps13D CG10947 Tet Tsp CG2162 smg lola fred CG30048 bin3 scrt sm CG33110 CG8405 CG11030 rgn nkd Xrp1 Neurochondrin lncRNA:CR44685 kto Tif-IA shep CG6040 lncRNA:CR44902 CG10839 Hacd1 Cdep ko nerfin-1 cta CG43915 CG13003 Gyf side-II Unc-115a Dhc62B upSET ara CG11486 CCKLR-17D1 Rme-8 CG14457 Mur2B CG9265 corto lncRNA:CR44197 DIP-zeta CG2121 CG34398 Ac78C pot Hasp lncRNA:CR45266 foxo wdb CG12589 CG32645 Irk2 lncRNA:CR45336 CG17574 toc Nrx-1 ATP8B milt CG33143 Spt20 Ccn Proc-R lncRNA:CR45411 Keap1 eIF4B CG4691 lncRNA:CR45276 CG4073 CG42319 nompC kirre PDZ-GEF CG17571 grn CG42404 CG5966 lncRNA:CR45689 CG42749 Corin E(spl)m6-BFM Gprk2 TyrR lncRNA:CR44750 futsch CG31183 lncRNA:CR45377 lncRNA:CR46006 CG14395 HP1Lcsd lncRNA:CR44339 CPT2 nej Obp56d Patronin Rev1 pip Camta CG3726 lncRNA:CR44980 lncRNA:CR44337 CG4238 CG9674 CG30456 Cpr50Cb CG12009 CG10348 CG18747 Rx Nc73EF bbg Trxr-2 Npc1b nahoda Eip78C lncRNA:CR45335 pre-rRNA:CR45847 cher lncRNA:CR44909 CG7470 sff CG3339 app wnd CG9492 DAAM alpha-Cat Mbs Sh KaiR1D lncRNA:CR43763 CG42335 CG42232 Pde8 Btk29A CG34357 Gr28b CR40068 side-III CG42524 lab CG32473 unc80 wry pyd Sytbeta CG6024 nAChRalpha2 Dgk vib side-VI hid Tom40 l(2)41Ab CG31998 mei-P26 CG34386 lncRNA:CR44494 lncRNA:CR46075 lncRNA:CR46004 ORY Fancm scro Hr4 CG6163 PpD5 CG42269 Dh31-R CG43373 lncRNA:CR44931 CG4502 spz3 l(3)psg2 trp CG42680 Cyp6a14 Ubx rl Rbp6 PsGEF Parp CG1090 CG5888 CG42673 LManIII TwdlQ CG42795 CG16965 Grd LRP1 CG34384 smog CG4650 lncRNA:CR13130 lncRNA:CR44786 Meltrin Apoltp MBD-R2 LRR step mesh CG15631 CG44475 Fs(2)Ket lncRNA:CR45308 pnt CG43143 CG7991 Grip CG44426 CG31693 CG14535 Gr23a bru2 jp CG5694 caup CG32085 RecQ4 CG31688 CG13898 ppk15 CG3635 Vha44 Drip lncRNA:CR45750 Ptp99A

		lncRNA:CR45228 CG32816 CG32206 SK elB S beat-IV CG31882 CG43658 dsx kuz asRNA:CR44894 asRNA:CR45909 PyK ths ps CG4297 CG8086 shn ACC lncRNA:CR44486 28SrRNA-Psi:CR45848 dpr9 mnd chrb CG43427 Gcn2 CG7548 tefu CG18812 ey pyr slgA spir Rfx Abl nrv1 CG1815 Dr Men lncRNA:CR46212 CG45781 CG15040 CG4662 Myo28B1 CG8854 Mur89F Trim9 snRNA:U2:14B Ttd14 SkpE spri TfAP-2 Dop1R2 tara unc-13-4A lncRNA:CR44350 tweek vvl Aldh-III Mef2 lncRNA:CR43848 fz2 lncRNA:CR45447 Pde1c Pka-C3 lncRNA:CR44349 FER lncRNA:CR45579 sas CG44838 CG40160 zormin Hipk mrj Usp10 Sxl esn lncRNA:CR44231 pwn Gbs-70E RhoGAP18B CG7236 Syt1 CG32432 PVRAP Cnx99A Wdr62 RasGAP1 Tlk CG31206 tut robo3 Piezo sti ogre Gie CG32683 nau Achl klu CG4629 CG34351 CG6044 CG5921 Cbp53E CG6678 hh ltl fra DNApol-epsilon255 vkg cdi neur lncRNA:CR44776 Smr CG41378 CG33298 5-HT7 l(3)72Ab CG15594 ced-6 dome Dhc64C CG5910 CG17514 CG7530 dpr21 sima ckn lncRNA:CR43627 Pgant2 rdgA CG31612 beat-VI pHCl-1 CG16826 csw CG12344 CG33467 Drl-2 CG9815 CG42852 CG42748 lncRNA:CR44443 rdog Osi24 CG42663 ich rost ETHR l(2)gl CG7294 Tl Ref2 Pzl CG6231 Sema2a Snap25 beat-Ic Fur1 CalpA Cngl X11Lbeta CG11883 lncRNA:CR44525 lncRNA:CR44621 Pura sty CG5890 lncRNA:CR44737 tyn CG42326 CG13699 bun lncRNA:CR44686 sNPF-R CG5973 Oaz lncRNA:CR44801 Rim ken bol Bsg gpp CG32758 al Sara CG34353 nvy CG43861 lncRNA:CR42836 stai pum alpha-Man-Ia Traf4 beat-Va CG34354 Rbp9 dsf Prip CG14669 lbl CG5004 CG8177 Tsp96F dpr13 CG30377 Cht7 CG3376 sfl Ggamma30A CG7378 Gprk1 CG5892 Src42A osa CG9932 spen Spt3 Nlg1 Rph lncRNA:CR45622 lov en Dhc36C Lim3 Mdr50 lncRNA:CR44754 lncRNA:CR44609 crol dpr6 CG12994 Gyc88E hppy cora Rbfox1 melt CG31523 lncRNA:flam CG7251 cac CG15395 CR34311 Rh7 if CG42342 Sox100B CG4577 mtg lncRNA:CR44552 side-V CG43313 CG15498 MRP Msp300 CG4998 CG8312 par-1 dia stl CG45263 Debcl Jupiter CG10178 tral CG10919 Pino eIF4EHP CR1075 Atf6 alph GluRIA mAChR-B Lim1 Tis11 Fife Doa lncRNA:CR45267 CG43337 CG8945 hang Pde6 kek6 Rok IP3K2 Cht10 lncRNA:CR43270 snky CG18586 Trpm SCAP Eip93F qua luna ec CG31121 heph gish Bruce lncRNA:CR45576 tRNA:Ser-AGA-1-1 crp nvd osp ACXC Ser CG32198 Sox21a drl Nrg Asator olf413 ZnT63C poe CG31741 Ca-alpha1T CG13784 Gr22e rhea Mhcl Osi6 CG31760 ush disco-r CG7544 CG12814 CG17129 lt Antp AGO3 CG6142 Poxm Pld lncRNA:CR45334 Ptp61F RyR dysc lncRNA:CR45054 cpo f rdgC mamo Nlg3 cv-2 CAH1 CG12516 MESR3
60 upregulated RNA-Seq	36	sca CG31636 CG30484 TTLL6B E(spl)mbeta-HLH CG32277 CG33159 CG5151 CG31211 Ucp4C TTLL3B Cp18 CG13685 CG13992 CG9068 CG32369 CG32271 CG10170 Oda Irk1 Hrb27C Cad87A CG13177 Chd64 CG6511 CG15894 Clk asRNA:CR44047 CG10063 lncRNA:CR45038 lncRNA:CR32385 RpL32 Rab5 ssp cep290 CG34232

Table S14. GO associations with Biological Process (GO Profiler) of 60 rDNA-contacting genes shown in the Venn diagram in Fig. 6C. The genes were upregulated after heat shock treatment.

GO.ID	Description	padj	Genes
GO:2000026	regulation of multicellular organismal development	0.00005113410542148909	TAI,CNC,MICAL,MGL,NEDD4,SSP,HRB27C,SYP,SDC,E(SPL)MBETA-HLH,SPIN,BNL,CHIC,CWO
GO:0051239	regulation of multicellular organismal process	0.00019993280423244542	TAI,CNC,MICAL,MGL,NEDD4,SSP,HRB27C,SYP,SDC,E(SPL)MBETA-HLH,SPIN,BNL,CHIC,CLK,CWO
GO:0050793	regulation of developmental process	0.001241017442743205	TAI,CNC,MICAL,MGL,NEDD4,SSP,HRB27C,SYP,SDC,E(SPL)MBETA-HLH,SPIN,BNL,CHIC,CWO
GO:0060284	regulation of cell development	0.00957474084776453	TAI,CNC,MICAL,NEDD4,HRB27C,E(SPL)MBETA-HLH,SPIN,CHIC,CWO
GO:0120039	plasma membrane bounded cell projection morphogenesis	0.011709163963156922	TAI,CNC,MICAL,NEDD4,HRB27C,RAB5,SDC,BNL,CHIC,CWO
GO:0048858	cell projection morphogenesis	0.012159770305567247	TAI,CNC,MICAL,NEDD4,HRB27C,RAB5,SDC,BNL,CHIC,CWO
GO:0120036	plasma membrane bounded cell projection organization	0.014145865672356732	TAI,TTLL3B,CNC,MICAL,NEDD4,CEP290,HRB27C,RAB5,SDC,BNL,CHIC,CWO
GO:0032990	cell part morphogenesis	0.014378445417645378	TAI,CNC,MICAL,NEDD4,HRB27C,RAB5,SDC,BNL,CHIC,CWO
GO:0030030	cell projection organization	0.016791508307157078	TAI,TTLL3B,CNC,MICAL,NEDD4,CEP290,HRB27C,RAB5,SDC,BNL,CHIC,CWO
GO:0032989	cellular component morphogenesis	0.017765188169053803	TAI,CNC,MICAL,ZASP52,NEDD4,HRB27C,RAB5,SYP,SDC,BNL,CHIC,CWO
GO:0007275	multicellular organism development	0.032198137037670334	IRK1,TAI,TTLL3B,CNC,MICAL,MGL,NEDD4,SCA,SSP,HRB27C,RAB5,SYP,SLOU,SDC,E(SPL)MBETA-HLH,CP18,SPIN,BNL,CHIC,CLK,CWO,WECH,

			ODA
GO:0032879	regulation of localization	0.045667483174053426	IRK1,TAI,CNC,MGL,NEDD4,CG43729,HRB27C,RAB5,BNL,CHIC
GO:0022008	neurogenesis	0.049784720367310645	TAI,CNC,MICAL,NEDD4,SCA,HRB27C,RAB5,SDC,E(SPL)MBETA-HLH,SPIN,BNL,CHIC,CWO

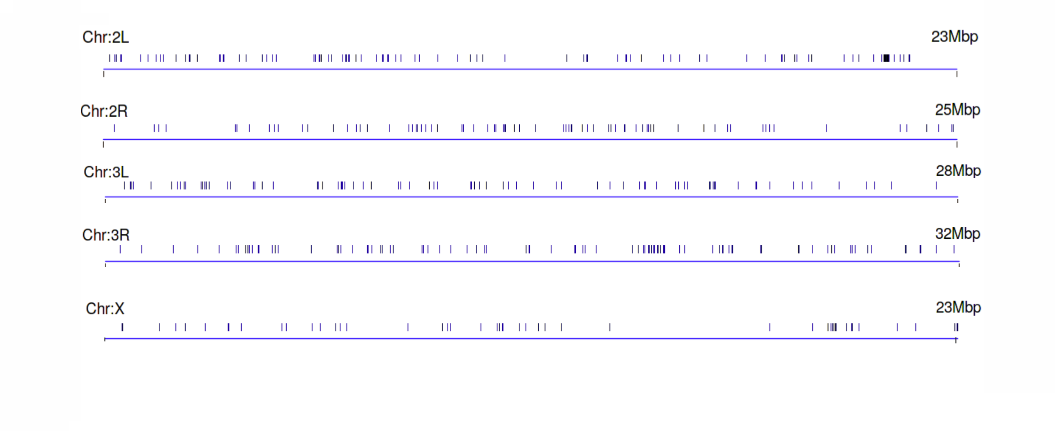

Figure S1. The distribution of 588 selected rDNA-contacting genes on *Drosophila* chromosomes. The search was performed using the http://bioinformatics.sdstate.edu/go/ resource. All chromosomes possess rDNA-contacting genes.

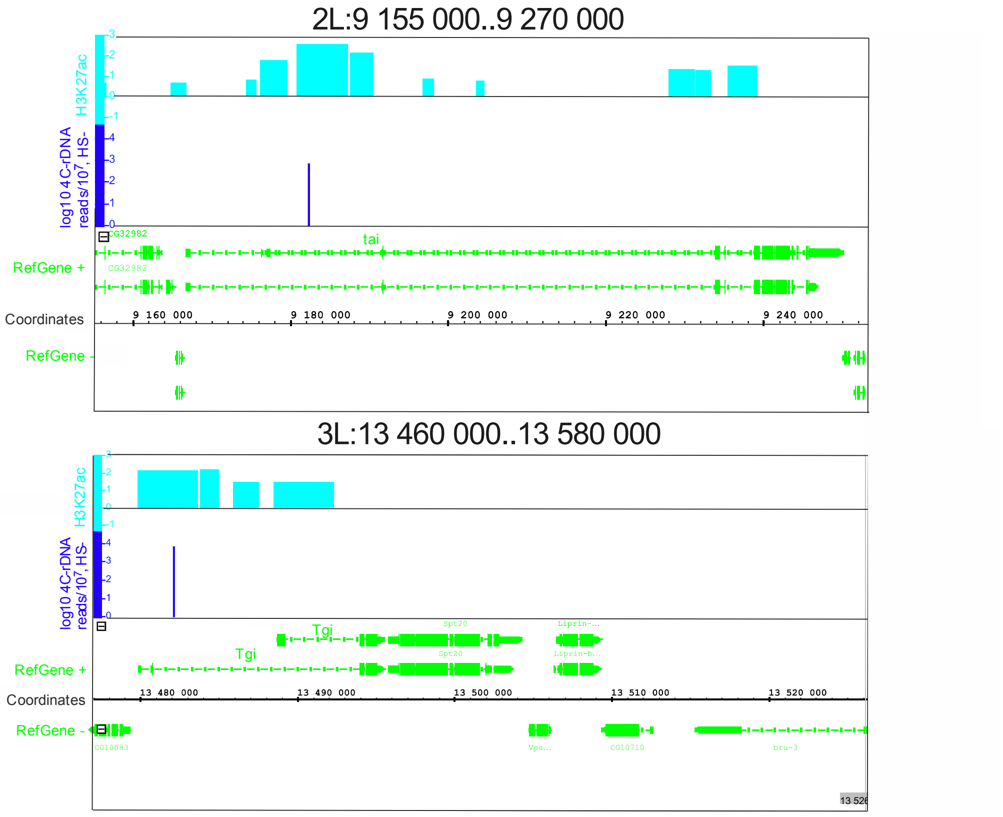

Figure S2. The rDNA-contacting sites often possess H3K27ac marks. Two examples are shown: a region of *Tai* genes in 2L (top) and a region of *Tgi* gene in 3L (bottom). Also, see H3K27ac profiles

in Fig. 6. Both genes specify transcription factors, are upregulated, and decrease the number of contacts with rDNA after heat shock treatment (see Fig. 6C).

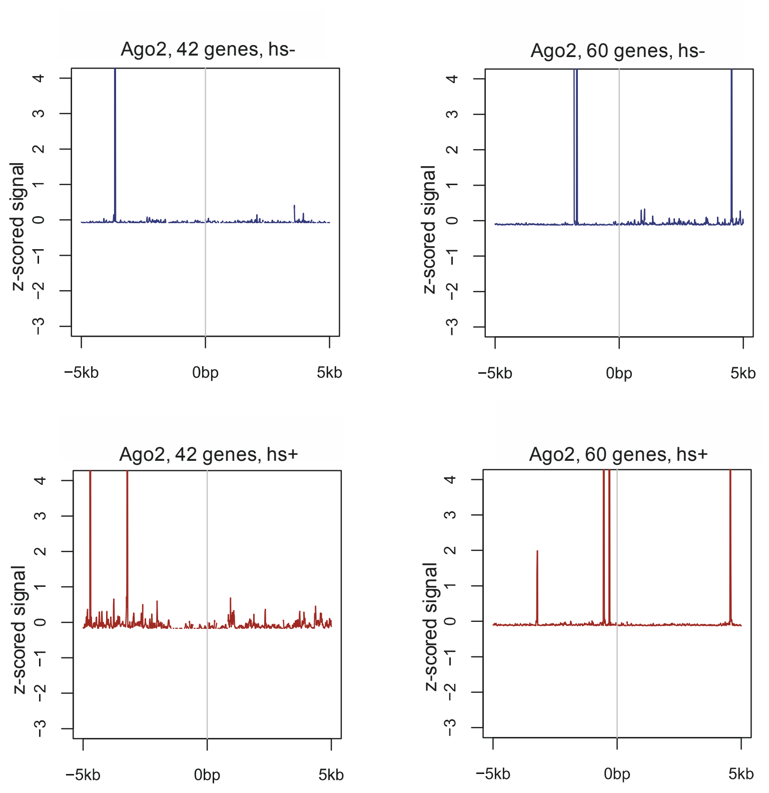

Figure S3. Profiles of Ago2 complexes around rDNA-contacting sites at 42 downregulated and 60 upregulated genes (see Figure 6 and Tables S13 and 14). The profiles shown in blue and red correspond to nucleoli contacts in normal and heat-shock-treated S2 cells, respectively.
